# Elucidating redox balance shift in *Scheffersomyces stipitis*’ fermentative metabolism using a modified genome-scale metabolic model

**DOI:** 10.1186/s12934-018-0983-y

**Published:** 2018-09-05

**Authors:** Matthew Hilliard, Andrew Damiani, Q. Peter He, Thomas Jeffries, Jin Wang

**Affiliations:** 1 Department of Chemical Engineering, Auburn University, Auburn, AL 36849 USA; 2 Xylome, Madison, WI 53719 USA; 3 Department of Bacteriology, University of Wisconsin at Madison, Madison, WI 53706 USA

**Keywords:** *Scheffersomyces stipitis*, Genome-scale metabolic network model (GEM), Redox balance, System identification, Phenotype phase-plane analysis

## Abstract

**Background:**

*Scheffersomyces stipitis* is an important yeast species in the field of biorenewables due to its desired capacity for xylose utilization. It has been recognized that redox balance plays a critical role in *S. stipitis* due to the different cofactor preferences in xylose assimilation pathway. However, there has not been any systems level understanding on how the shift in redox balance contributes to the overall metabolic shift in *S. stipitis* to cope with reduced oxygen uptake. Genome-scale metabolic network models (GEMs) offer the opportunity to gain such systems level understanding; however, currently the two published GEMs for *S. stipitis* cannot be used for this purpose, as neither of them is able to capture the strain’s fermentative metabolism reasonably well due to their poor prediction of xylitol production, a key by-product under oxygen limited conditions.

**Results:**

A system identification-based (SID-based) framework that we previously developed for GEM validation is expanded and applied to refine a published GEM for *S. stipitis*, iBB814. After the modified GEM, named iDH814, was validated using literature data, it is used to obtain genome-scale understanding on how redox cofactor shifts when cells respond to reduced oxygen supply. The SID-based framework for GEM analysis was applied to examine how the environmental perturbation (i.e., reduced oxygen supply) propagates through the metabolic network, and key reactions that contribute to the shifts of redox and metabolic state were identified. Finally, the findings obtained through GEM analysis were validated using transcriptomic data.

**Conclusions:**

iDH814, the modified model, was shown to offer significantly improved performance in terms of matching available experimental results and better capturing available knowledge on the organism. More importantly, our analysis based on iDH814 provides the first genome-scale understanding on how redox balance in *S. stipitis* was shifted as a result of reduced oxygen supply. The systems level analysis identified the key contributors to the overall metabolic state shift, which were validated using transcriptomic data. The analysis confirmed that *S. stipitis* uses a concerted approach to cope with the stress associated with reduced oxygen supply, and the shift of reducing power from NADPH to NADH seems to be the center theme that directs the overall shift in metabolic states.

**Electronic supplementary material:**

The online version of this article (10.1186/s12934-018-0983-y) contains supplementary material, which is available to authorized users.

## Background

*Scheffersomyces stipitis* has been recognized as an important yeast species in the field of biorenewables due to its desired capacity for utilizing xylose [1], the second most abundant sugar in lignocellulosic biomass. Its enzymes for xylose assimilation have been used to engineer *Saccharomyces cerevisiae* for both glucose and xylose conversion to produce ethanol [2, 3]. In addition, CRISPR-based genetic tools have recently been developed for *S. stipitis*, making it a potential platform stain for producing various compounds derived from the shikimate pathway [4].

For biological organisms, cofactor balances play critical roles in maintaining intracellular redox hemostasis, which has been recognized to be a prerequisite for robust growth and metabolism [5]. This is especially the case for *S. stipitis*, because the first two reactions in xylose assimilation pathway, i.e., xylose reductase (XR) and xylitol dehydrogenase (XDH), prefer different cofactors. XR prefers NADPH while XDH strictly depends on NAD^+^, which leads to redox imbalance. It has been suggested that a major cause for the limited growth performance and ethanol biosynthetic capacity of *S. stipitis* with xylose as substrate is the redox bottleneck, rather than enzyme activity deficiency that hinders specific metabolic pathways [6, 7]. It is well-recognized that the cellular redox balance is sustained through an intricate network with multiple redox reactions. However, currently there has not been a systems level analysis nor understanding on how different metabolic pathways involving production/consumption of cofactors shift in a coherent fashion in response to reduced oxygen supply to produce ethanol.

### Genome-scale metabolic models (GEMs) for *S. Stipitis*

To gain genome-wide understanding on *S. stipitis*’ cellular metabolism, which is the foundation of various applications, two genome-scale metabolic models (GEMs), iSS884 [8] and iBB814 [9], have been reconstructed based on the sequenced genome of *S. stipitis* [1]. A GEM is a comprehensive functional database of the organism’s cellular metabolism [10, 11], which consists of a set of metabolites, metabolic reactions (i.e., stoichiometric matrix), and constraints. GEMs can be used to conduct simulations/computations to answer various questions about the capabilities of the organism and its likely phenotypic states. GEMs, especially those of model organisms *Escherichia coli* and *Saccharomyces cerevisiae*, have been successfully utilized in many applications, including metabolic engineering, model-driven discovery, prediction of cellular phenotypes, and others [12, 13].

Similar to models developed in various science and engineering fields, the quality of a GEM determines the successfulness of its applications. Therefore, model validation plays an important role in GEM development. Besides assessing its size and connectivity, the current standard approach for GEM validation is to compare model predictions with experimental data under different conditions [11]. Most often the experimental data consist of measured cross-membrane fluxes, i.e., various substrate uptake rates, product excretion rates, and cell growth rate. With the recent advancement of “omics” technologies, gene-expression profiles, gene deletion data, and proteomic profiles are also used for model validations. Such a validation approach is deemed as the gold standard for evaluating the quality of a GEM. We term these approaches “point-matching” approaches because each experimental condition represents a single (although potentially high dimensional) point in the phenotypic space. For well-characterized organisms, point-matching approaches work well, because their metabolic network structures have been well-studied and well-defined. However, given the fact that a GEM, especially a less studied one, is severely underdetermined (i.e., large degree of freedom), matching numerical experimental data over a few limited conditions does not necessarily indicate a high-quality GEM and can result in very misleading conclusions. This was clearly demonstrated in our recent study on the evaluation of the two GEMs of *S. stipitis* [14]. In that study, although one model (iSS884) consistently showed much better agreement with experimental measurements than the other model (iBB814) across multiple data sets in terms of product secretion rates, its prediction for several mutant strains are incorrect. Such a lack of predictability suggests that iSS884 contains internal errors.

### System identification-based GEM validation

To address the shortcomings of point-matching validation approaches, we proposed a system identification (SID) based framework for GEM validation [14]. In the SID framework, biological knowledge embedded in a GEM is first extracted from a series of designed in silico experiments through multivariate analysis methods such as principal component analysis (PCA); next, the extracted knowledge, such as how cells respond under a given stimulus, is visualized and compared with the existing knowledge for model validation and analysis. We term the proposed approach “knowledge-matching” as the simulation results are not directly compared with experimental data; instead, the knowledge captured by the model is compared with available knowledge. Although rooted in simulations, the SID-based approach is more of a qualitative validation, instead of a quantitative approach, and offers additional robustness against measurement errors. In [14], through the knowledge-matching based validation, we have shown that although iSS884 has much better agreement with experimentally measured cross-membrane fluxes, it contains some significant errors. On the other hand, although iBB814 shows poorer performance in quantitative point-matching validations, it captures the knowledge that aligns better with existing knowledge on *S. stipitis* [14]. In this work, built upon our previous work on the GEM comparison and validation, we present iDH814, an improved GEM for *Scheffersomyces stipitis*.

### Need of an improved GEM for *S. stipitis*

For any microorganism, obtaining a high-quality GEM is challenging and time consuming due to the scale and complexity involved at genome-scale [13]. The still advancing GEMs of *Escherichia coli* (since 2000) and S*accharomyces cerevisiae* (since 2003) suggest that the development of the initial version of a GEM is a groundbreaking start; after which, significantly more efforts are needed to validate and refine the GEMs [13, 15]. Being the first GEMs of *S. stipitis*, iSS884 and iBB814 represent significant steps forward in gaining a genome-scale understanding of the cellular metabolism of *S. stipitis*; and at the same time, it is natural that both models have certain limitations. For example, the prediction of xylitol production from both GEMs are quite different from the experimental observations. For iSS884, xylitol is not produced under any condition; while for iBB814, xylitol is only produced under conditions that are almost completely anaerobic, where no cell growth is predicted. Since xylitol is one of the major by-products under oxygen-limited conditions as observed in wet lab experiments, it is essential that the modified GEM can predict the production of xylitol under oxygen-limited conditions, before it is utilized to gain systems level understanding of *S. stipitis*’ fermentative metabolism.

Currently, GEM refinement is typically accomplished through trial-and-error by modifying different reactions in the model and examining whether model predictions improve. This process relies heavily on the modeler’s knowledge and capability to sort out clues from various simulation results. Therefore, GEM refinement is usually labor intensive and time consuming [13, 15]. To help address this challenge, this work expands our proposed SID framework to guide GEM refinement and uses the development of iDH814 to illustrate how the SID framework can help identify the root causes of erroneous model behaviors; and thus, expedite the GEM refinement process.

## Materials and methods

### Two GEMs of *S. stipitis*: iBB814 and iDH814

iBB814 [9] was constructed manually by following a published protocol [16]. The improved model, iDH814, was developed by using iBB814 as the reference model, and the model refinement was guided by the SID-based framework. Table 1 lists these models’ basic information.

**Table 1:** Basic Information for two *P. stipitis* GEMs iBB814 and iDH814

Specification	iBB814	iDH814
Reactions	1371	1380
Cytosol	751	759
Mitochondria	125	126
Transport	495	495
Metabolites	971	972
genes	814	814
Percent of genome (%)	13.6	13.6
Compartment	Cytosol, exchange, mitochondria	Cytosol, exchange, mitochondria

### The SID-based framework for GEM refinement

**Fig. 1:**
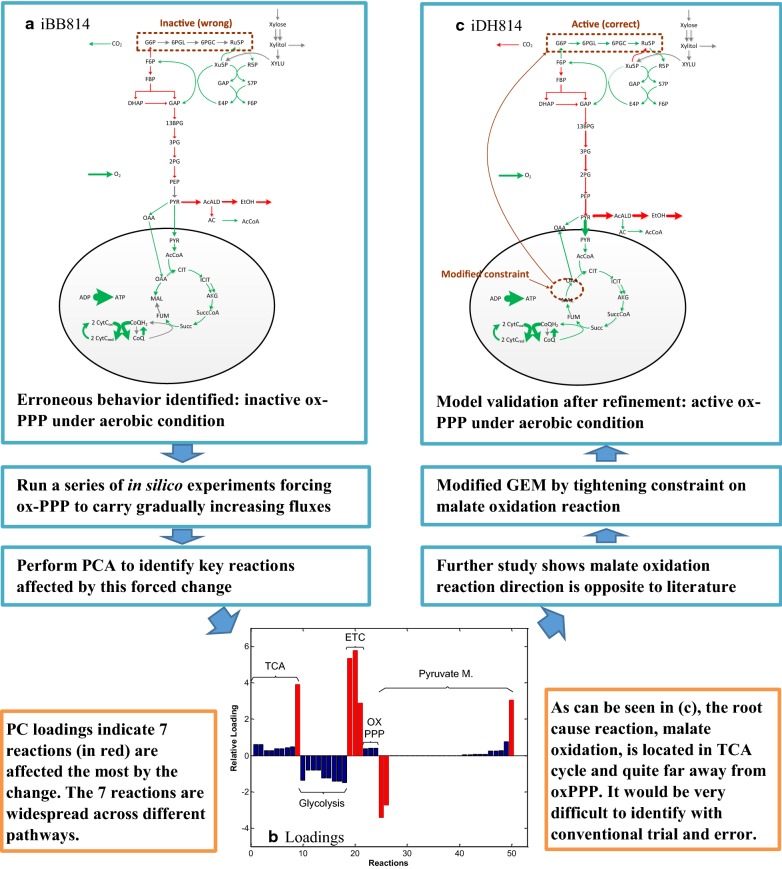
Demonstration of the SID based framework for GEM refinement

For GEM refinement, the biggest challenge is to identify the root cause of an erroneous model behavior. Because of the complex interconnectivity in a GEM, many times seemingly unrelated reactions located far away from the “problematic” reactions (i.e., reactions that are not carried out in the expected way) play a key role in changing model behavior, and the point-matching validation does not provide information on such “hidden” relations. In this section, we present how the SID-based framework can be applied to identify candidate root causes for a particular model faulty behavior, and in doing so, expedite GEM refinement. Specifically, we use the refinement of iBB814 for one particular error as an example to illustrate the following four steps in the proposed SID-guided GEM refinement as shown in Fig. 1.

1. Identify the erroneous model behavior: one of the identified errors in iBB814 is that the oxidative branch of pentose phosphate pathway (oxPPP) is off under aerobic conditions (Fig. 1a). This is contrary to the common knowledge for *S. stipitis* (and other microorganisms), as it is well accepted that oxPPP should be active under aerobic conditions to generate reducing equivalents needed for cell synthesis.
2. Conduct in silico experiments by forcing desired model behavior (through, e.g., applying additional constraints) to identify the key reactions that contribute to this erroneous model behavior: a series of in silico experiments were conducted by forcing proportionally increasing flux through oxPPP with increasing O_2_ uptake under aerobic conditions.
3. Apply SID to identify which reactions respond most significantly to the forced change: Principal component analysis (PCA) identifies key reactions based on the magnitude of principal component (PC) loadings (Fig. 1b). Seven reactions that had the largest loadings were identified as candidates for further examination. Figure 1b shows that these seven reactions spread across a wide range of reaction pathways, including tricarboxylic acid cycle (TCA), glycolysis, electron transport chain (ETC) and pyruvate metabolism, and may not be easily identified through a trial-and-error approach.
4. Determine which candidate reactions and/or their constraints should be modified and/or what new reactions should be added: careful examination of the seven reactions showed that one reaction, malate oxidation to oxaloacetate, was carried out in the opposite direction as suggested by the literature. By increasing the lower bound of the malate oxidation reaction from − 1000 mmol/gCDW/h to 0 mmol/gCDW/h, oxPPP becomes active under aerobic conditions (Fig. 1c).

This example shows that the malate oxidation reaction, the root cause of the erroneous model behavior, i.e. inactive oxPPP under aerobic condition, is located quite away from oxPPP pathway. Without the guidance of the SID framework, it could be very difficult to quickly identify this source of error. However, with the SID-based analysis, the overall network response provides key information to help quickly identify the root cause of the error.

### Transcriptomic data acquisition and analysis

*Scheffersomyces stipitis* CBS 5773 was obtained from ATCC. The frozen stock and culture media were the same as in Liang et al. [17]. All experiments were conducted in a BioFlo 115 with 2 L working volume. After inoculation, the cells were cultured under batch mode until biomass reached 6 g/L and switched to continuous mode. Once a controlled chemostat of aerobic growth was achieved and maintained, two samples were collected 10 min apart. Next, the oxygen supply was immediately and significantly reduced to induce oxygen-limited fermentation, and the dilution rate was simultaneously adjusted to maintain a relatively constant cell density. After the cells were maintained at the oxygen-limited chemostat for 24 h, two more samples were collected 10 min apart for the oxygen-limited chemostat. The experiment was repeated for two times.

Each cell sample was centrifuged for 5 min at 10,000 rpm at room temperature. After centrifugation, the samples were decanted and the remaining cell pellets were immediately submerged into liquid nitrogen. The frozen cell samples were stored in a − 80 °C freezer until all cell samples from both trials were collected. The frozen cell samples were shipped to the University of Wisconsin-Madison Biotechnology Center, which conducted the RNA extraction, and analysed the samples through Illumina Next-Generation Sequencing. The data set consists of raw count data, fragments per kilobase per million reads (FPKM), and transcripts per million (TPM). The raw reads were aligned to the CBS6054 genome using STAR aligner, and the aligned reads were quantified using RSEM [18, 19]. After alignment, each sample contains 5966 gene expression levels.

The gene-to-protein-to-reaction (GPR) rules provided in the GEM were used to calculate the reaction expression values (protein expression values) for different pathways. For reactions catalyzed by multiple isozymes, we use the sum of the expressions for the associated genes. If a protein consists of subunits, the protein expression is determined by considering the minimum expression of the subunits. These rules were also used in [20].

## Results and discussion

In this section, we first provide detailed information on the differences between iBB814 (the base model) and iDH814 (the modified model) and evaluate the quality of iDH814. Specifically, we first subject iDH814 to phenotype phase plane (PhPP) Analysis and provide a detailed analysis along the line of optimality where cell growth is optimal; then we evaluate the model through the conventional point-matching approach by comparing the model predictions with published experimental results. Once validated, we use published experimental data to guide the design of in silico experiments and apply the SID-based framework to understand how cellular metabolism shift in response to reduced oxygen supply. In particular, we thoroughly examined the cellular redox cofactor consumption and production to obtain genome-scale understanding on the redox shift that contributes to the overall metabolic shift. Finally, the findings obtained through GEM analysis were validated using transcriptomic data collected on *S. stipitis* under aerobic growth and oxygen-limited fermentation.

### The modified GEM iDH814

Following the procedure illustrated in Fig. 1, as well as integrating available knowledge on *S. stipitis*, we obtained a modified GEM, iDH814, based on iBB814. In summary, starting with iBB814, we modified 13 reactions, deleted 10 reactions and added 19 reactions to obtain iDH814. Additional file 1: Table S1, Additional file 2: Table S2, Additional file 3: Table S3 list the detailed reactions that have been modified, removed and added to iBB814 to obtain iDH814. The model files are provided as Additional file 4: Table S4 and Additional file 5: Table S5.

#### Overall model behavior through phenotype phase plane analysis

**Fig. 2:**
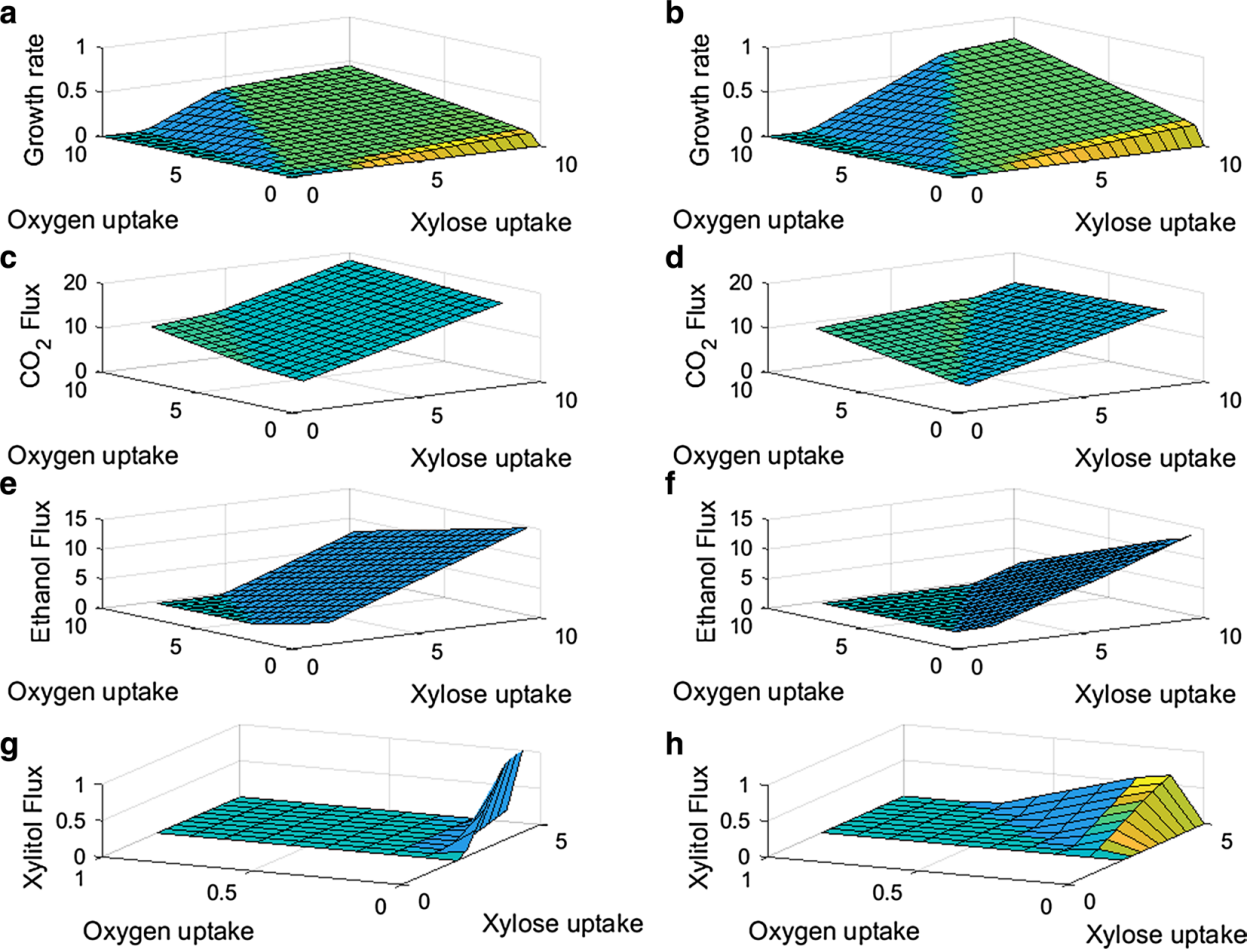
3D phenotype phase planes for the two GEMs. **a** Growth PhPP for iBB814, **b** growth PhPP for iDH814, **c** CO_2_ production PhPP for iBB814, **d** CO_2_ production PhPP for iDH814, **e** ethanol production PhPP for iBB814, **f** ethanol production PhPP for iDH814, **g** xylitol production PhPP for iBB814, **h** xylitol production PhPP for iDH814. Uptake and flux values given in mmol/gCDW/h. Growth rate given in h^−1^

One major application of GEMs is to predict different growth phenotypes (e.g., how fast cells grow, what products are excreted) under various genetic and environmental conditions. Developed by the Palsson lab, phenotype phase plane (PhPP) analysis is a powerful tool that utilizes flux balance analysis (FBA) with a GEM to provide a global perspective on the genotype–phenotype relationship, and to help characterize different metabolic phenotypes [21]. Here we compare iBB814 and iDH814 through PhPP analysis, where Fig. 2 compares the two models for their predictions on the cell growth, CO_2_, ethanol, and xylitol production over a wide range of xylose and oxygen uptake rates. Figure 2 indicates that, although the two models follow similar trends in terms of cell growth rate and various product secretion rates, when culture condition changes, they do contain some key differences. For example, iDH814 predicts higher growth rates and less CO_2_ production than iBB814, while ethanol secretion is predicted over a much narrower range of conditions compared to iBB814. More importantly, Fig. 2g, h clearly show that the xylitol production pattern from iDH814 agrees with experimental observations, i.e., the peak xylitol production occurs under oxygen limited condition, instead of anaerobic condition where the cell cannot grow at all; and xylitol is produced at much wider range where cell growth is feasible, which also agrees with experimental observations.

#### Model comparison through analysis along line of optimality

**Fig. 3:**
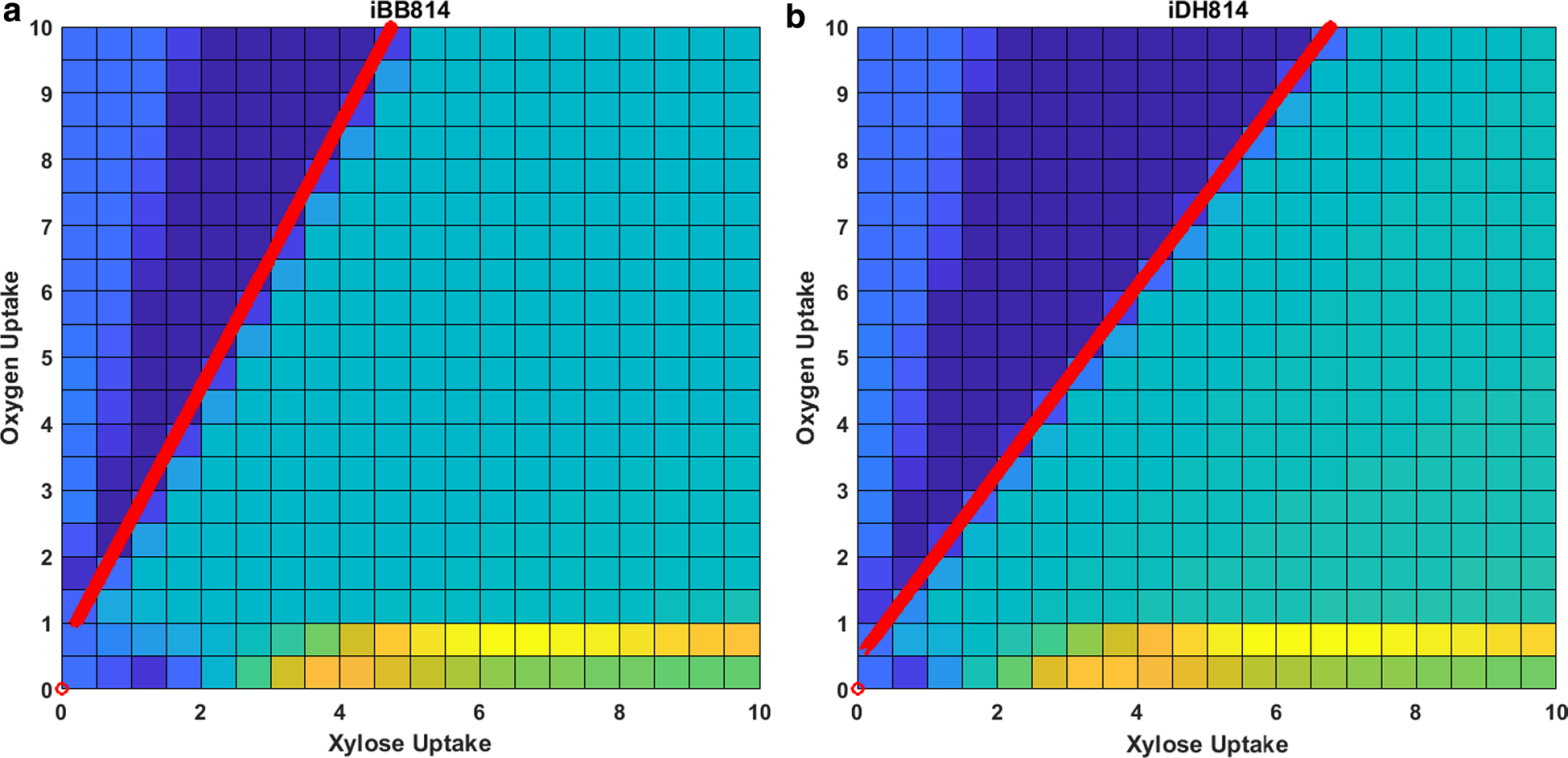
2D phenotype phase plots with LO indicated by red line for **a** iBB814 and **b** iDH814. Uptake values given in mmol/gCDW/h

Figure 3 shows the 2D PhPP plots of the two GEMs, which is obtained through projecting Fig. 2a, b onto the plane defined by the xylose and oxygen uptake rates. The different colors/shades indicate different phenotypes. The straight lines marked on the plots are the line of optimality (LO) determined by each model. The LO represents the optimal relationship between the carbon and oxygen uptake rates for maximum cell growth. It has been shown that for well-defined GEMs, the model predicted LO agrees very well with experimental results [21, 22]. Therefore, a necessary condition for a high-quality GEM would be that the model performs well along the LO. Hence, as one of the first steps for examining the two models in detail, we performed SID-based analysis along the LO, and compared the knowledge captured by the two models.

**Fig. 4:**
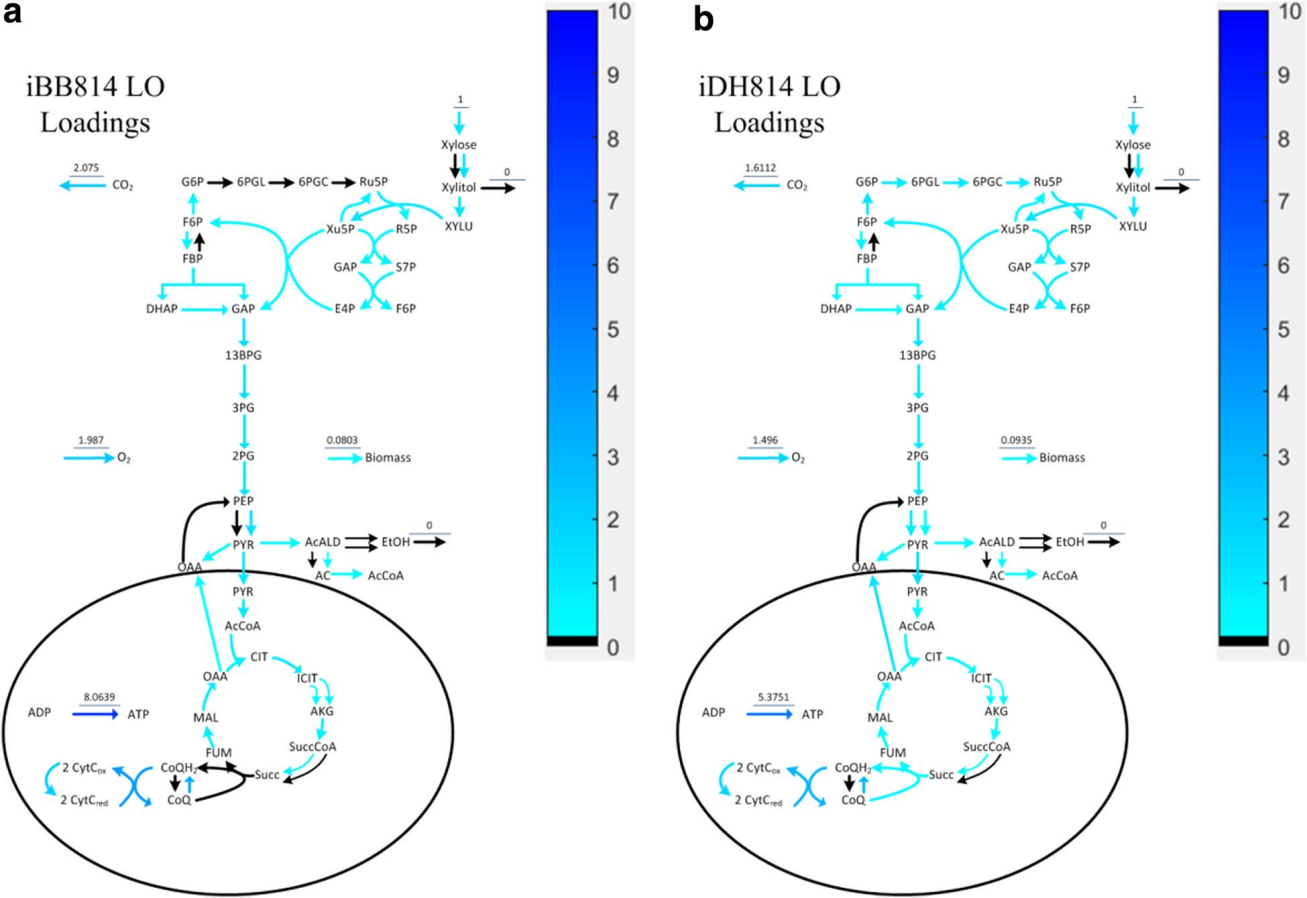
PCA loading values visualized on central carbon metabolic network maps for **a** iBB814 and **b** iDH814 along the LO

Along the LO, since carbon and oxygen uptake rates increase proportionally at the fixed optimal ratio, it is expected that all activated pathways should carry proportionally increasing fluxes to achieve increased maximum cell growth. Therefore, when SID-extracted information along the LO is visualized, only blue colored (upregulating flux) and black colored (inactive reaction) reaction arrows are expected. Figure 4 compares the knowledge captured by each model along their corresponding LO.

In this analysis, the LO is determined by changing the xylose uptake rate and allowing the oxygen uptake rate to be determined by FBA with maximizing cell growth as the objective. Figure 4a shows that the result from iBB814 is mostly as expected, except the oxidative branch of PPP is inactive. This indicates that the knowledge captured by iBB814 is mostly correct, at least for aerobic conditions.

Figure 4b indicates that along the LO, the knowledge captured by iDH814 fully agrees with the expected behavior. The loadings shown in Fig. 4 indicate how the flux through each reaction changes corresponding to the change in xylose uptake rate. The different loading values for the O_2_ uptake reaction indicate the different slopes of the corresponding LO, as shown in Fig. 3; more specifically, to assimilate 1 mol of xylose for optimal growth, iBB814 requires 1.987 mol of O_2_, whereas iDH814 only requires 1.496 mol of O_2_. The larger loading for the CO_2_ exchange reaction in iBB814 indicates that it produces more CO_2_ than iDH814, which agrees with what is revealed in PhPP analysis, i.e., Fig. 2c, d. Correspondingly, the smaller loading for cell growth rate in iBB814, indicates the smaller increase in cell growth rate when moving along the LO compared to iDH814, which is also confirmed by Fig. 2a, b.

#### Conventional point-matching with experimental data

It is worth noting that iDH814 was developed through the “knowledge-matching” approach only, without any attempt to fit available experimental data; in addition, the knowledge utilized to guide the model refinement is general to many different microorganisms, such as the example shown in Fig. 1. To further evaluate the modified model for its quantitative prediction performance, several published experimental results were used to evaluate the model through the “point-matching” approach. The results predicted by iDH814 were compared to the results by iSS884 and iBB814. Here we included the results from iSS884 because it performed much better than iBB814 in point-matching validations [14].

To provide a fair comparison, we used the same datasets that were used in Damiani et al. [14] for iSS884 and iBB814 evaluation. One data set was taken from Caspeta et al. [8], and the other from Li [23]. Table 2 lists the model prediction errors. For the three GEMs, the model input (i.e., xylose and oxygen pickup fluxes) were set to the experimental values, and model predictions (i.e., biomass, ethanol, and CO_2_ fluxes) were compared with experimental values at seven different conditions. Table 2 clearly shows that iDH814 provides the best quantitative validation performance, although the model was derived purely based on a knowledge-matching approach.

**Table 2:** Point-matching validation of three GEMs

Data source	Exp. cond.^a^	Product	err %^b^		
			iSS884	iBB814	iDH814
Caspeta et al. [8]	O: 0.24 C: 3.11	EtOH	29.9	39.1	*26.7*
		CO_2_	11.8	16	*7.8*
		Cell	32.1	66.1	*31.8*
	O: 0.35 C: 3.03	EtOH	2.3	6.6	*0.29*
		CO_2_	22.7	28.5	*21.4*
		Cell	*24*	63.7	*24*
	O: 0.75 C: 2.55	EtOH	*2.5*	16	3.6
		CO_2_	*26.6*	38.8	27.1
		Cell	*40.7*	75.7	49.9
Li [23]	O: 1.64 C: 4.10	EtOH	11.1	36.5	*1.0*
		CO_2_	37	55	*24.1*
		Cell	47	30.3	*24.2*
	O: 6.23 C: 4.69	EtOH	*0.17* ^1^	2.72^1^	0.8^1^
		CO_2_	12.2	18.9	*4.3*
		Cell	26.6	40.4	*2.5*
	O: 1.33 C: 3.28	EtOH	*6.5*	14.5	7.4
		CO_2_	6.2	6.1	*2.2*
		Cell	*18.2*	63.2	22.2
	O: 6.00 C: 4.32	EtOH	*0*	2.3^1^	*0*
		CO_2_	10.5	21.3	*9.7*
		Cell	*12.4*	47	*5.5*

^a^Units C/O flux: mmol/(gCDW/h). In [8], xylose is the substrate. In [23], xylose is the substrate for the first two conditions and glucose for the last two conditions. Since NADPH dependent glutamate dehydrogenase (GDH3) is repressed by glucose, the corresponding reaction was disabled for the two cases where glucose is the substrate

^b^$err\% = \left| {\frac{Pred. - Exp.}{Exp.}} \right|*100\%$. When the experimental value is zero, absolute error is given (marked with ^1^). Better prediction values (i.e., smaller errors) are shown in italics

### Using iDH814 to understand *S. stipitis’* fermentative metabolism

With iDH814 validated through both knowledge-matching and point-matching approaches, we apply the SID-based framework to study the fermentative phenotype predicted by the GEM. The goal is to obtain system-level understanding on the strain’s fermentative metabolism.

#### Further validation of iDH814 for xylitol production

Xylitol has been shown to be a major by-product of *S. stipitis* under fermentation. We have shown that iDH814 is able to predict xylitol production in a reasonable way, here we compared its prediction with experimental data available in literature on xylitol production [24].

As noted previously, because of the different redox cofactor preferences of XR and XDH, when xylose is converted to xylulose in *S. stipitis*, a redox imbalance is resulted and excess amount of NADH is produced. Since both routes of XR reactions that consume NADH and NADPH are included in the GEMs, without further constraints, the NADH-dependent route is always activated by FBA, as it will generate faster growth rate due to the balanced redox. To address this limitation, for the simulations conducted in this section, we added a constraint on the flux ratio between NADPH-dependent and NADH-dependent XR, to reflect the available knowledge on the XR cofactor preference [25].

Table 3 compares the model predictions with reported experimental values. Since in Farias et al. [24], the oxygen uptake rate was not reported for the experiments, the comparison was conducted by matching the model predicted ethanol yield with the reported value through tuning oxygen uptake flux and XR ratio; then, the model predicted xylitol yields were compared with the reported experimental value, which show excellent agreement.

**Table 3:** Comparison of iDH814 xylitol results to experimental data

Case	Y_E/S_ experiment	Xylitol experimental (mmol/gCDW/h)	Xylitol prediction (mmol/gCDW/h)	Ratio of XR NADPH/NADH	OUR
1	0.328	0.1408	0.1408	0.94	2.33
2	0.358	0.0934	0.0935	0.207	1.64
3	0.376	0.0115	0.0116	0.11	1.42
4	0.437	0.0172	0.0173	0.11	0.50

Y_E/S_ is the ethanol yield, where xylose is the substrate (S). Xylose reductase (XR) redox ratio and oxygen uptake rate (OUR) were varied. Minimized the error between ethanol yield of experiment and prediction from iDH814, then examined the xylitol productions from experiments and those predicted by iDH814

Table 3 shows that to match the experimental reported ethanol yield, which monotonically increases from condition 1 to condition 4, the model requires monotonically decreasing oxygen uptake rates and concurrently shifting the XR’s cofactor preference from NADPH to NADH. Such trends agree with available knowledge: when oxygen supply is abundant, the excess NADH produced through XR-XDH reaction pair can be shuttled into mitochondria where they enter ETC to produce ATP with oxygen as the electron acceptor; when less oxygen is available, then less NADH can be processed via ETC. Therefore, it is reasonable that XR cofactor preference shifts toward NADH to reduce the demand on oxygen for NADH consumption.

#### Analysis of the cellular metabolic shift with reduced oxygen at genome-scale

**Fig. 5:**
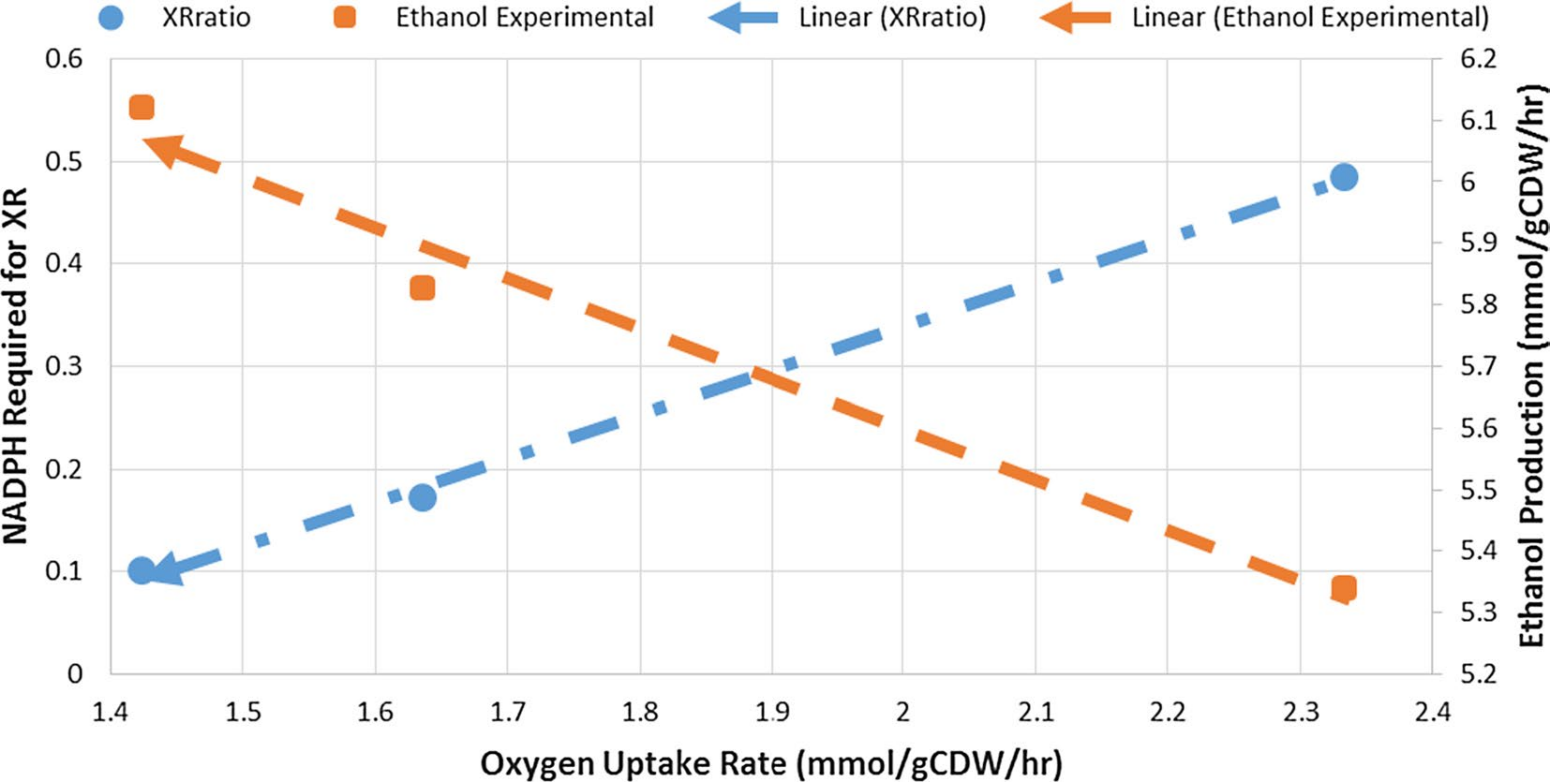
Experimental ethanol production and NADPH stoichiometric coefficient in XR vs OUR. As can be seen, the linear fits for XRratio and ethanol production indicate that the three points are in the same phenotypic phase

To gain a better understanding on how the cellular redox balance shifts at genome-scale when oxygen uptake rate (OUR) decreases, we applied the SID-based framework to investigate the cellular response to the change in OUR using iDH814. As mentioned before, first a series of in silico experiments should be designed and carried out to help reveal how the cellular redox balance shifts when OUR decreases. Because iDH814 does not contain the gene regulatory mechanism on cofactor utilization, in silico experiments were carefully designed to reflect what is going on within the cell by integrating experimental results. In Fig. 5, we plotted the ethanol yield and the model fitted XR cofactor preference corresponding to OUR for conditions 1–3. Figure 5 shows the linear correlations between the XR cofactor preference and OUR, as well as between ethanol yield and OUR. These linear relationships suggest that the three conditions are located within the same phenotype phase [26]. Based on Fig. 5, we designed a series of in silico experiments corresponding to conditions that are located along the straight line as indicated in Fig. 5. Specifically, for these in silico experiments, as OUR was reduced linearly from 2.33 to 1.42 mmol/gDCW/h, the XR cofactor preference ratio (NADPH:NADH) was decreased linearly from 0.93 to 0.10, while keeping a constant xylose uptake rate (5 mmol/gDCW/h).

**Fig. 6:**
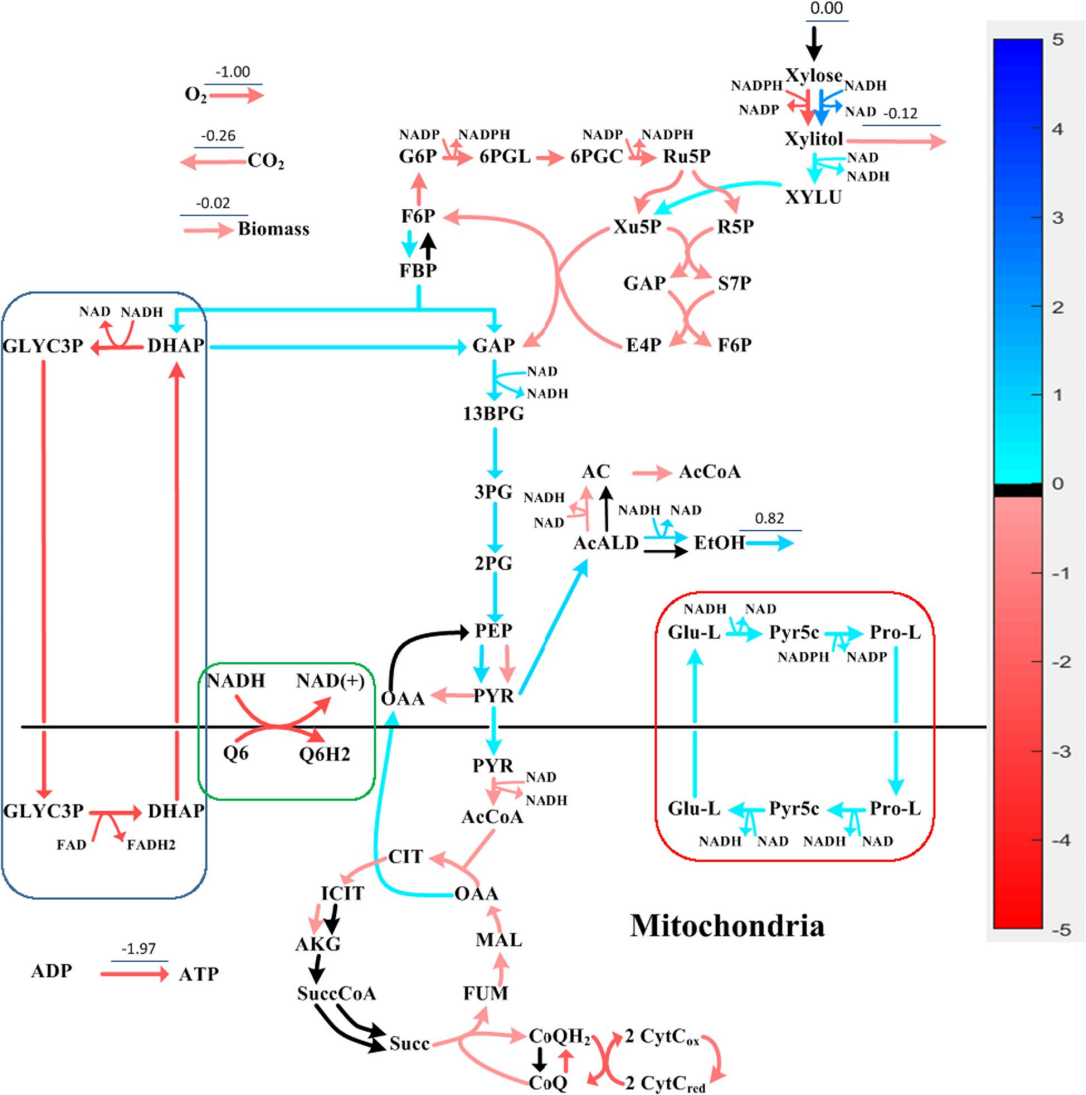
Visualization of the metabolic network response induced by the reduced oxygen uptake

Next, PCA was applied to analyze the resulting flux distribution matrix. One principal component (PC) captured 100% of the variance, which confirms that conditions along the linear segment shown in Fig. 5 indeed belong to the same phenotype. The PC loadings were then visualized against the metabolic network map to better understand the intracellular metabolic responses predicted by the model, as shown in Fig. 6. In Fig. 6, blue reaction arrows indicate that the reaction fluxes are upregulated, red down regulated, and black no change in flux through the reaction. Figure 6 shows that corresponding to the reduced OUR and shift in XR cofactor preference, PPP, TCA and ETC pathways are all down regulated, while glycolysis and ethanol production are upregulated. Such responses agree with available understanding: when XR cofactor preference shifts toward NADH, less NADPH is needed for xylose reduction, which explains the reduced flux through oxPPP, the major route for cytosolic NADPH production. As a result of the decrease in carbon flux through oxPPP, the carbon is rerouted through glycolysis and eventually converted to ethanol which is secreted by the cell. In addition to an increase in ethanol yield corresponding to reduced OUR, which is observed in experiments, Fig. 6 confirms that xylitol yield also decreases as noted by the negative loading value for the xylitol secretion reaction. Regarding NADH production and consumption, Fig. 6 shows that as OUR decreases, the ATP production through ETC decreases (due to reduced oxygen availability), indicating that less amount of NADH can be oxidized through this route. To compensate for this, as XR cofactor preference shifts toward NADH, less amount of excess NADH is produced through XR-XDH pair; hence, less amount of NADH should be shuttled into mitochondria for ATP production. Because the inner membrane of the mitochondria is impermeable to NADH [27], electron shuttles, metabolic pathways that facilitate the transfer of electrons (in the form of NADH) produced in the cytosol to the electron transport chain in mitochondria, are needed. It has been reported that in *Saccharomyces cerevisiae*, the two major electron shuttle pathways are external NADH dehydrogenase (NADHDH) and the glycerol-3-phosphate (GLYC3P) shuttles [28]. Our analysis results show that *S. stipitis* also primarily utilizes the GLYC3P shuttle and/or the NADHDH shuttle. As revealed by Fig. 6, such reduction in excess NADH was indeed reflected in the down regulation of the two electron shuttles: GLYC3P shuttle (highlighted in blue box) and NADHDH shuttle (highlighted in green box). It should be noted that the use of GLYC3P and NADHDH shuttles by iDH814 were obtained as alternative optimal solutions of FBA, indicating both shuttles could be in operation at the same time.

#### Analysis of the cellular redox shift with reduced oxygen at genome-scale

It has been well recognized that redox balance is one of the prerequisites for robust growth and metabolism, and the redox imbalance caused by xylose assimilation pathway in *S. stipitis* has been suggested as a major cause for the limited growth performance and ethanol biosynthesis capacity of *S. stipitis*. Since cellular redox balance is sustained through an intricate network with multiple redox reactions, a genome-scale understanding on how cellular redox shift with reduced OUR will provide valuable insights for mutant design, especially for redox cofactor engineering. In this section, we use the modified model iDH814 to gain system-level understanding on how the key contributing reactions to the redox shift respond in a coherent fashion to the reduced oxygen supply and produce ethanol.

Based on the in silico experimental results obtained in the previous section, we tabulated the total NADH, NADPH production/consumption in cytosol and the total Q6H2 production/consumption in mitochondria predicted by the GEM when the cells transition from condition 1 to condition 3. All reactions that contribute to the redox shift are listed in Additional file 6: Table S6 and Additional file 7: Table S7, for NADH and NADPH involved reactions, correspondingly. As shown in Table 4, as cells transition from condition 1 to condition 3 due to reduced OUR, the total production/consumption of cytosolic NADH slightly increased by 6% (0.67 mmol/gDCW/h), while the total production/consumption of cytosolic NADPH decreased significantly by 46% (1.138 mmol/gDCW/h).

**Table 4:** Summary of the balance of cytosolic electron carriers

Metabolite	Process	Cond 1	Cond 3	Cond 3 − Cond 1
NADH	Produced	11.77	12.44	0.67
	Consumed	11.77	12.44	0.67
NADPH	Produced	3.96	2.13	− 1.83
	Consumed	3.96	2.13	− 1.83
Q6H2	Produced	4.42	2.63	− 1.79
	Consumed	4.42	2.63	− 1.79

The detailed flux information provided in Additional file 6: Table S6 and Additional file 7: Table S7 indicates that there are only a few reactions that contribute significantly to the shift of redox balance, which are listed in Table 5. The major contributing reactions to redox balance are also shown in Fig. 6. Besides the reactions contained in central carbon metabolic network that consume/produce NADH/NADPH, the rest of the major contributors are all part of the three electron shuttle systems: two shuttle systems for NADH (i.e., NADHDH and GLYC3P, shown in green and blue boxes respectively), and one shuttle system for combined NADH/NADPH (termed glutamate (GLU) shuttle, shown in red box in Fig. 6).

**Table 5:** Reactions that contribute significantly to shift in redox balance

Role	Reaction	Reaction equation	Flux change^a^	Match TPM trend
NADH production	GAPD	−1 nad[c] +−1 pi[c] +−1 g3p[c] ⟷ 1 h[c]+1 nadh[c]+1 13dpg[c]	0.585	Yes
	XDH	−1 h[c] +−1 nadh[c] +−1 xylu-D[c] ⟷ 1 nad[c]+1 xylt[c]	0.112	Yes
NADH consumption	ALCDH	−1 etoh[c] +−1 nad[c] ⟷ 1 h[c]+1 acald[c]+1 nadh[c]	0.746	Yes
	NADHDH	−1 h[c] +−1 nadh[c] +−1 q6[m] ⟶ 1 nad[c]+1 q6h2[m]	− 2.294	Yes
	XYLR1	−1 h[c] +−1 nadh[c] +−1 xyl-D[c] ⟶ 1 nad[c]+1 xylt[c]	1.950	N/A^b^
	PYRC	−1 h[c] +−1 nadh[c] +−1 glu-L[c] ⟷ 2 h2o[c]+1 nad[c]+1 1pyr5c[c]	0.282	No
NADPH production	G6PDH	−1 nadp[c] +−1 g6p[c] ⟶ 1 h[c]+1 nadph[c]+1 6pgl[c]	− 0.892	Yes
	GND	−1 nadp[c] +−1 6pgc[c] ⟶ 1 nadph[c]+1 co2[c]+1 ru5p-D[c]	− 0.892	Yes
NADPH consumption	XYLR2	−1 h[c] +−1 nadph[c] +−1 xyl-D[c] ⟶ 1 nadp[c]+1 xylt[c]	− 1.950	N/A^b^
	P5CR	−2 h[c] +−1 nadph[c] +−1 1pyr5c[c] ⟶ 1 nadp[c]+1 pro-L[c]	0.282	No
Q6H2 production	NADHDH	−1 h[c] +−1 nadh[c] +−1 q6[m] ⟶ 1 nad[c]+1 q6h2[m]	− 2.294	Yes
Q6H2 consumption	CYOR_u6m	−2 h[m] +−1 q6h2[m] +−2 ficytc[m] ⟶ 4 h[c]+1 q6[m]+2 focytc[m]	− 1.788	Yes
ATP production	ATPSm	−1 adp[m] +−1 pi[m] +−4 h[c] ⟶ 1 atp[m]+1 h2o[m]+4 h[m]	− 1.793	Yes

^a^Flux change values given in mmol/gCDW/h

^b^The gene for xylose reductase is the same for both the NAD and NADP dependent routes

Figure 6 shows that due to the reduced amount of oxygen available as an electron acceptor in the mitochondria, the amount of NADH that is transported into the mitochondria was reduced significantly (65.7% decrease of shuttle flux compared to condition 1), which is expected. In addition, it was somewhat surprising to see the dramatic increase (126% increase compared to condition 1) of flux through the GLU shuttle. However, a closer look at the GLU shuttle reveals that the shuttle consumes 1 NADH and 1 NADPH in cytosol, but produces 2 NADH in the mitochondria, which suggests that the GLU-shuttle effectively serves as an NAD(P)^+^ transhydrogenase that converts cytosolic NADPH into mitochondrial NADH, which helps balance the significant shift from NADPH to NADH.

Regarding the redox shifts in the mitochondria, in Table 4 we have only listed the production and consumption of Q6H2, since the final output of each electron shuttle that is active in iDH814 is Q6H2, which subsequently enters the ETC for ATP production. As shown in Table 4, the decrease of Q6H2 production/consumption in the mitochondria is 1.79 mmol/gDCW/h, which accounts for 98% of the reduced oxygen supply (OUR decreased from 2.33 to 1.42 mmol/gDCW/h, representing decrease of 1.82 mmol/gDCW/h electron acceptors).

This analysis suggests that to cope with the stress caused by reduced oxygen supply, cells rely on increased production of NADH, as well as converting cytosolic NADPH to mitochondrial NADH to achieve the drastic shift from NADPH to NADH as the reducing factor.

#### Validation of the GEM-based analysis using transcriptomic data

In this section, we use transcriptomic data collected under aerobic growth and oxygen-limited conditions to validate the findings we obtained through GEM based analysis. Although the relationship between gene expression levels and the metabolic flux through the corresponding enzymatic reaction is not direct, transcriptomic data obtained through RNA-Seq analysis offers an opportunity to help gain genome-wide understanding on how an organism responds to environmental/genetic perturbations. It has been assumed that the trend of significant gene-regulation should align with flux changes, i.e., significant up-regulations would result in increased fluxes, while significant down-regulations would result in decreased fluxes [20]. Therefore, we compared the direction of changes in GEM predicted flux when oxygen uptake rate is reduced, with the direction of changes in measured gene expression levels to validate the findings obtained from the GEM-based analysis.

Table 6 lists the comparison result for the flux and gene expression level changes (from two trials) for the central carbon network (CCN), and the genome-wide comparison is given in Additional file 8: Table S8 and Additional file 9: Table S9. Table 6 only includes reactions that carry fluxes and there are 17 reactions in CCN that do not carry flux. In addition, Table 6 does not include the exchange or transport reactions. Finally, the xylose reductase reactions were not included, because the same enzyme can either utilized NADH or NADPH and there is no way to distinguish the two routes. In Table 6, the gene expression levels that showed larger than 10% of changes relative to their expression levels under aerobic growth are denoted by “+” or “−” for increase and decrease, respectively; while the ones show less than 10% of changes, either increase or decrease, are denoted by “o”.

**Table 6:** GEM predicted flux changes vs. experimental gene expression level changes

Reaction	Flux change	TPM change (trial I)	TPM change (trial II)	Reaction	Flux change	TPM change (trial I)	TPM change (trial II)
GAPD	+	+	+	TKT1	−	−	−
PGK	+	+	+	G6PI	−	−	−
PGM	+	+	+	G6PDH	−	−	−
ENO	+	+	+	PGL	−	−	−
PYK	+	+	+	GND	−	−	−
ATPSm	−	o	−	NADH2-u6t	+	+	o
ATPtm-H	−	+	+	GLUD2	−	−	−
PYRDC	+	+	+	RPE	+	−	−
ALCDH	+	+	+	ACS1	−	−	−
XYLUR	+	+	+	ALDDH1	−	−	−
XYLK	+	+	+	PDHm	−	−	−
CYOR_u6m	−	o	−	PC	−	o	−
SUCCDH1m	−	o	−	ASPGLU2 m	+	−	−
G3PD1*	−	−	−	PYRC	+	−	−
NADHDH*	−	−	−	P5CR	+	−	−
G3PDm	−	o	−	PRO1 m	+	−	−
RPI	−	−	−	PYRCm	+	−	−
FBA	+	−	+	ACONHm	−	−	−
PFK	+	+	+	CITSm	−	−	−
TPI	+	+	+	ICDH1m	−	−	−
TKT2	−	−	−	FUMm	−	−	−
TALA	−	+	+	MDHm	−	−	−
CYOOm	−	+	+	SUCCDHpm	−	−	−

From Table 6 it can be seen that 76% (Trial I) and 80% (Trial II) of reaction flux changes agree with transcriptomic level changes which include all key contributors identified by the SID-based analysis, indicating high quality prediction by iDH814. Specifically, iDH814 predicts reduced fluxes for both of the two NADH shuttles (i.e., the NADHDH and GLYC3P shuttles), together with increased ethanol production to cope with reduced oxygen supply. The reduced fluxes through both NADH shuttles were confirmed by the transcriptomic data as genes associated with both NADH shuttles show reduced expression levels. It is worth noting that it has been reported inhibiting NADHDH shuttle can increase ethanol production as suggested by the SID-based analysis; however, the inhibition only resulted in 18% of increase in ethanol production, and such less than expected increase can be explained by the operation of the GLYC3P shuttle, which was not considered in [29].

In addition, the last column of Table 6 indicates whether the change predicted by iDH814 agrees with gene expression data, which clearly shows that all key contributors identified by iDH814 based analysis are supported by transcriptomic data, except few related to GLU shuttle. Although one could argue that the discrepancy in the NADPH shuttle could be explained by translational control and/or post translational modification, we think it is more likely due to the potential error contained in the model and/or lack of the key regulatory mechanisms.

## Conclusion

In this work, we expanded our previously developed SID-based framework for GEM validation to GEM refinement and demonstrated its effectiveness through developing an improved GEM for *Scheffersomyces stipitis*. Through designed in silico experiments, the SID-based framework can identify candidate reactions to examine in order to correct erroneous prediction behaviors, and thus, expedite the GEM refinement process. Based on our previous results of GEM evaluation for *S. stipitis*, we applied SID-based framework on a previously published GEM model iBB814 to derive an improved GEM, iDH814. Through a series of examinations and comparison experiments, we showed that iDH814 offers improved performance in both traditional point-matching validations and the proposed knowledge-matching validations, although iDH814 was developed solely based on knowledge-matching.

With the modified GEM iDH814 being capable of producing xylitol, we designed and performed additional in silico experiments to gain a more in-depth understanding of how *S. stipitis* adjusts its cellular metabolism in response to a decrease in OUR through ethanol production. To compensate for the limitation that iDH814 (or all existing GEMs of *S. stipitis*) does not contain a gene-regulatory mechanism to adjust XR cofactor preference, we used experimental data to derive additional constraints in order to reflect what happens in cellular metabolism when OUR decreases. The systems level analysis obtained through the SID-based framework identified the key reactions contributed to the metabolic shift trigger by reduced oxygen supply. In addition, the intricate network of redox reactions involved in the metabolic shift were identified as well. These findings offer key insight to the mutant design for cofactor engineering. Finally, the iDH814 predicted flux changes were validated by transcriptomic data, which showed 75–80% agreement between predicted changes by iDH814 and transcriptomic data.

The analysis results confirmed that *S. stipitis* uses a concerted approach to cope with the stress associated with reduced oxygen supply, and the shift of reducing power from NADPH to NADH seems to be the center theme that organizes the overall shift in metabolic states. It is worth noting that although the SID-based framework can only reveal correlations amongst the changes that happen to different pathways, we could derive certain causal relationships among them by integrating available information (such as introduced perturbation) and existing knowledge to derive testable hypotheses on causal relationships.

## Additional files

**Additional file 1: Table S1.** iBB814 Reactions Modified to Produce iDH814.

**Additional file 2: Table S2.** iBB814 Reactions Deleted to Produce iDH814.

**Additional file 3: Table S3.** Reactions added to Produce iDH814.

**Additional file 4: Table S4.** GEM metabolites.

**Additional file 5: Table S5.** GEM reactions.

**Additional file 6: Table S6.** All cytosolic reactions that use NADH as a cofactor with non-zero flux.

**Additional file 7: Table S7.** All cytosolic reactions that use NADPH as a cofactor with non-zero flux.

**Additional file 8: Table S8.** Genome-wide comparison for Trial I.

**Additional file 9: Table S9.** Genome-wide comparison for Trial II.

## Abbreviations

ETC: electron transport chain
FBA: flux balance analysis
FADH2: flavin adenine dinucleotide reduced
GLYC3P: glycerol-3-phosphate
GEM: genome-scale metabolic model
gCDW: gram cell dry weight
LO: line of optimality (objective function is cell growth unless specified otherwise)
NAD(+): nicotinamide adenine dinucleotide
NADH: nicotinamide adenine dinucleotide-reduced
NADPH: nicotinamide adenine dinucleotide phosphate-reduced
OUR: oxygen uptake rate
oxPPP: oxidative pentose phosphate pathway
P5CR: pyrroline-5-carboxylate reductase
PC: principal component
PCA: principal component analysis
PhPP: phenotype phase plane analysis
PPP: pentose phosphate pathway
Q6H2: ubiquinol-6
SID: systems identification
TCA: tricarboxylic acid cycle
XR: xylose reductase
XDH: xylitol dehydrogenase
Y_P/S_: yield of metabolite P with respect to substrate S (g/g)
