# Supplementary material for: Elucidating redox balance shift in Scheffersomyces stipitis’ fermentative metabolism using a modified genome-scale metabolic model
Source: Microb Cell Fact. 2018 Sep 5;17:140. doi: 10.1186/s12934-018-0983-y (PMC6126012; doi:10.1186/s12934-018-0983-y)
Supplement: Supplementary file 1 — Additional file 1: Table S1. iBB814 Reactions Modified to Produce iDH814. [file 12934_2018_983_MOESM1_ESM.pdf]

Table S1: iBB814 Reactions Modified to Produce iDH814

Reaction Name	Reaction Equation	Change
NADH dehydrogenase, mitochondrial	1h[m] + 1nadh[m] + 1q6[m] -> 1nad[m]+1q6h2[m]	Proton Change
Ubiquinol-6 cytochrome C reductase	1q6h2[m] + 4h[m] + 2ficytc[m] -> q6[m] + 4h[c] + 2focytc[m]	Proton Change
Cytochrome C oxidase, mitochondrial	1O2[m] + 8h[m] + 4focytc[m] -> 2h2o[m] + 8h[c] + 2ficytc[m]	Proton Change
Succinate-CoA ligase (ATP-forming), mitochondrial	1adp[m] + 1pi[m] + 1succoa[m] <=> 1succ[m] + 1coa[m] + 1atp[m]	Reverse direction blocked
Succinate-CoA ligase (GDP-forming), mitochondrial	1gdp[m] + 1pi[m] + 1succoa[m] <=> 1succ[m] + 1coa[m] + 1gtp[m]	Reverse direction blocked
Malate dehydrogenase, mitochondrial	1nad[m] + 1mal-L[m] <=> 1h[m] + 1oaa[m] + 1nadh[m]	Reverse direction blocked
Malate dehydrogenase, cytosolic	1nad[c] + 1mal-L[c] <=> 1h[c] + 1oaa[c] + 1nadh[c]	Reverse direction blocked
Phosphoribosyl-pyrophosphate synthetase	1atp[c] + 1r5p[c] <=> 1h[c] + 1amp[c] + 1prpp[c]	Reverse direction blocked
Nucleoside-diphosphate kinase (ATP:GDP)	1atp[c] + 1gdp[c] <=> 1adp[c] + 1gtp[c]	Reverse direction blocked
Nucleoside-diphosphate kinase (ATP:dGDP)	1atp[c] + 1dgdp[c] <=>1adp[c] + 1dgtp[c]	Reverse direction blocked
Nucleoside-diphosphate kinase (ATP:dADP)	1atp[c] +1dadp[c] <=> 1adp[c]+1datp[c]	Reverse direction blocked
Isoleucine transaminase	1 akg[c] +1 ile-L[c] <=> 1glu-L[c] + 1 3mop[c]	Reverse direction blocked
Leucine transaminase, mitochondrial	1 akg[m] + 1 leu-L[m] <=> 1 glu-L[m] + 1 4mop[m]	Reverse direction blocked
