# Supplementary material for: Elucidating redox balance shift in Scheffersomyces stipitis’ fermentative metabolism using a modified genome-scale metabolic model
Source: Microb Cell Fact. 2018 Sep 5;17:140. doi: 10.1186/s12934-018-0983-y (PMC6126012; doi:10.1186/s12934-018-0983-y)
Supplement: Supplementary file 2 — Additional file 2: Table S2. iBB814 Reactions Deleted to Produce iDH814. [file 12934_2018_983_MOESM2_ESM.pdf]

Table S2: iBB814 Reactions Deleted to Produce iDH814

Reaction Name	Reaction Equation
D-arabinitol exchange	1 abt-D[e] -> 1 na
L-arabinitol exchange	1 abt-L[e] -> 1 na
Fumarate reductase	1 fum[c] + 1 fadh2[m] -> 1 succ[c] + 1 fad[m]
Fumarase	1 h2o[c] + 1 fum[c] -> 1 mal-L[c]
Isocitrate dehydrogenase (NADP+)	1 nadp[c] + 1 icit[c] -> 1 akg[c] + 1 nadph[c] + 1 co2[c]
D1-pyrroline-5-carboxylate dehydrogenase, mitochondrial	1 glu5sa[m] + 1 h2o[m] + 1 nadp[m] -> 1 glu-L[m] + 2 h[m] + 1 nadph[m]
Succinate Dehydrogenase (Ubiquinone) Mitochondrial	1 succ[m] + 1 q6[m] ó 1 fum[m] + 1 q6h2[m]
Pyruvate transport in via proton symport	1 h[e] + 1 pyr[e] -> 1 pyr[c]+1 h[c]
Spermine acetyltransferase	1 accoa[c] + 1 sprm[c] -> 1 h[c] + 1 coa[c] + 1 N1sprm[c]
Valine-pyruvate aminotransferase	1 ala-L[c] + 1 3mob[c] -> 1 pyr[c]+1 val-L[c]
