# Supplementary material for: Elucidating redox balance shift in Scheffersomyces stipitis’ fermentative metabolism using a modified genome-scale metabolic model
Source: Microb Cell Fact. 2018 Sep 5;17:140. doi: 10.1186/s12934-018-0983-y (PMC6126012; doi:10.1186/s12934-018-0983-y)
Supplement: Supplementary file 3 — Additional file 3: Table S3. Reactions added to Produce iDH814. [file 12934_2018_983_MOESM3_ESM.pdf]

## Table S3: Reactions added to Produce iDH813

| Reaction Name | Reaction Equation |
|---|---|
| GMP:diphosphate 5-phospho-alpha-D-ribosyltransferas | 1 prpp[c] + 1 gua[c] <=> 1 ppi[c] + 1 gmp[c] |
| Guanosine aminohydrolase | 1 h2o[c] + 1 gsn[c] <=> 1 nh4[c] + 1 xtsn[c] |
| CDP-diacylglycerol:L-serine 3-phosphatidyltransferase | 1 ser-L[c] + 0.01 cdpdag[c] <=> 0.01 ps[c] + 1 cmp[c] |
| L-Alanine:2-oxoglutarate aminotransferase | 1 ala-L[c] + 1 akg[c] <=> 1 pyr[c] + 1 glu-L[c] |
| Glutamate Decarboxylase | 1 glu-L[m] + 1 h[m] -> 1 co2[m] + 1 4abut[m] |
| 4-aminobutyrate aminotransferase | 1 akg[m] + 1 4abut[m] <=> 1 glu-L[m] + 1 succsal[m] |
| Succinate Semialdehyde Dehydrogenase | 1 h2o[m] + 1 nadp[m] + 1 succsal[m] -> 1 succ[m]+2 h[m]+1 nadph[m] |
| Malate-alpha-ketoglutarate shuttle | 1 akg[m] + 1 mal-L[c] <=> 1 akg[c] + 1 mal-L[m] |
| (S)-1-pyrroline-5-carboxylate:NAD+ oxidoreductase | 1 h[c] + 1 nadh[c] + 1 glu-L[c] <=> 2 h2o[c] + 1 nad[c] + 1 1pyr5c[c] |
| (S)-1-pyrroline-5-carboxylate:NAD+ oxidoreductase, Mitochondrial | 1 glu-L[m] + 1 h[m] + 1 nadh[m] <=> 2 h2o[m]+1 nad[m]+1 1pyr5c[m] |
| Mitochondrial 2-oxodicarboxylate transporter | 1 akg[c] + 1 2oxoadp[m] <=> 1 akg[m]+1 2oxoadp[c] |
| 1-Phosphatidyl-D-myo-inositol inositolphosphohydrolase | 1 h2o[c] + 1 ptd1ino[c] <=> 1 12dgr[c]+1 mi1p-D[c] |
| Choline sulfotransferase | 1 paps[c] + 1 chol[c] <=> 1 pap[c] + 1 cholso4[c] |
| 2-aceto-2-hydroxybutanoate synthase, mitochondrial | 1 accoa[c] + 1 spmd[c] -> 1 h[c] + 1 coa[c] + 1 N1aspmd[c] |
| Phospholipase D | 1 chol[c] + 1 cdpdag[c] <=> 1 pc[c]+1 cmp[c] |
| Guanosine ribohydrolase | 1 h2o[c] + 1 gsn[c] <=> 1 rib-D[c] + 1 gua[c] |
| Uridine ribohydrolase | 1 h2o[c] + 1 uri[c] <=> 1 rib-D[c] + 1 ura[c] |
| Hydrogen Transport | 1 h[c] -> 1 h[e] |
| Isocitrate dehydrogenase (NAD+), cytosol | 1 nad[c] + 1 icit[c] -> 1 nadh[c] + 1 co2[c] + 1 akg[c] |
