# Supplementary material for: Elucidating redox balance shift in Scheffersomyces stipitis’ fermentative metabolism using a modified genome-scale metabolic model
Source: Microb Cell Fact. 2018 Sep 5;17:140. doi: 10.1186/s12934-018-0983-y (PMC6126012; doi:10.1186/s12934-018-0983-y)
Supplement: Supplementary file 4 — Additional file 4: Table S4. GEM metabolites. [file 12934_2018_983_MOESM4_ESM.pdf]

Table S4: GEM metabolites

Metabolite name	Metabolite description	Metabolite neutral formula	Metabolite charged formula	Metabolite charge
succ[m]	Succinate		C4H4O4	-2
atp[m]	ATP		C10H12N5O13P3	-4
coa[m]	Coenzyme A		C21H32N7O16P3S	-4
adp[m]	ADP		C10H12N5O10P2	-3
pi[m]	Phosphate		HO4P	-2
succoa[m]	Succinyl-CoA		C25H35N7O19P3S	-5
gtp[m]	GTP		C10H12N5O14P3	-4
gdp[m]	GDP		C10H12N5O11P2	-3
abt-D[e]	D- Arabitol		C5H12O5	0
abt-L[e]	L-Arabitol		C5H12O5	0
fum[c]	Fumarate		C4H2O4	-2
fadh2[m]	Flavin adenine dinucleotide reduced		C27H33N9O15P2	-2
succ[c]	Succinate		C4H4O4	-2
fad[m]	Flavin adenine dinucleotide oxidized		C27H31N9O15P2	-2
nadp[c]	Nicotinamide adenine dinucleotide phosphate		C21H25N7O17P3	-3
etoh[c]	Ethanol		C2H6O	0
h[c]	H+		H	1
nadph[c]	Nicotinamide adenine dinucleotide phosphate - reduced		C21H26N7O17P3	-4
acald[c]	Acetaldehyde		C2H4O	0
ala-L[c]	L-Alanine		C3H7NO2	0
3mob[c]	3-Methyl-2-oxobutanoate		C5H7O3	-1
pyr[c]	Pyruvate		C3H3O3	-1
val-L[c]	L-Valine		C5H11NO2	0
paps[c]	3-Phosphoadenylyl sulfate		C10H11N5O13P2S	-4
chol[c]	Choline		C5H14NO	1

pap[c]	Adenosine 3,5-bisphosphate	C10H11N5O10P2		-4
cholso4[c]	Choline Sulfate	C5H13NO4S		0
h2o[c]	H2O	H2O		0
so4[c]	Sulfate	O4S		-2
nadh[c]	Nicotinamide adenine dinucleotide - reduced	C21H27N7O14P2		-2
abt-D[c]	D- Arabitol	C5H12O5		0
nad[c]	Nicotinamide adenine dinucleotide	C21H26N7O14P2		-1
rbl-D[c]	D-Ribulose	C5H10O5	[]	
atp[c]	ATP	C10H12N5O13P3		-4
adp[c]	ADP	C10H12N5O10P2		-3
pi[c]	Phosphate	HO4P		-2
asp-L[c]	L-Aspartate	C4H6NO4		-1
citr-L[c]	L-Citrulline	C6H13N3O3		0
amp[c]	AMP	C10H12N5O7P		-2
argsuc[c]	N(omega)-(L-Arginino)succinate	C10H17N4O6		-1
ppi[c]	Diphosphate	HO7P2		-3
glu-L[c]	L-Glutamate	C5H8NO4		-1
4abut[c]	4-Aminobutanoate	C4H9NO2		0
co2[c]	CO2	CO2		0
succsal[c]	Succinic-semialdehyde	C4H5O3	[]	
akg[c]	2-Oxoglutarate	C5H4O5		-2
akg[m]	2-Oxoglutarate	C5H4O5		-2
asp-L[m]	L-Aspartate	C4H6NO4		-1
glu-L[m]	L-Glutamate	C5H8NO4		-1
oaa[m]	Oxaloacetate	C4H2O5		-2
ala-D[c]	D-Alanine	C3H7NO2		0
o2[c]	O2	O2		0
nh4[c]	Ammonium	H4N		1
h2o2[c]	Hydrogen peroxide	H2O2		0
arg-L[c]	L-Arginine	C6H15N4O2		1

oaa[c]	Oxaloacetate	C4H2O5	-2
ala-L[m]	L-Alanine	C3H7NO2	0
pyr[m]	Pyruvate	C3H3O3	-1
gln-L[c]	L-Glutamine	C5H10N2O3	0
hco3[c]	Bicarbonate	CHO3	-1
cbp[c]	Carbamoyl phosphate	CH2NO5P	-2
asn-L[e]	L-Asparagine	C4H8N2O3	0
h2o[e]	H2O	H2O	0
asp-L[e]	L-Aspartate	C4H6NO4	-1
nh4[e]	Ammonium	H4N	1
asn-L[c]	L-Asparagine	C4H8N2O3	0
prpp[c]	5-Phospho-alpha-D-ribose 1-diphosphate	C5H8O14P3	-5
pram[c]	5-Phospho-beta-D-ribosylamine	C5H11NO7P	-1
acgam[c]	N-Acetyl-D-glucosamine	C8H15NO6	0
acgam6p[c]	N-Acetyl-D-glucosamine 6-phosphate	C8H14NO9P	-2
uacgam[c]	UDP-N-acetyl-D-glucosamine	C17H25N3O17P2	-2
chitin[c]	Chitin (monomer)	C8H13NO5	0
udp[c]	UDP	C9H11N2O12P2	-3
ac[c]	Acetate	C2H3O2	-1
chitos[c]	Chitosan	C6H11NO4	0
ficytb5[c]	Ferricytochrome b5		[]
focytb5[c]	Ferrocytochrome b5		[]
gam6p[c]	D-Glucosamine 6-phosphate	C6H13NO8P	-1
acgam1p[c]	N-Acetyl-D-glucosamine 1-phosphate	C8H14NO9P	-2
utp[c]	UTP	C9H11N2O15P3	-4

f6p[c]	D-Fructose 6-phosphate	C6H11O9P	-2
accoa[c]	Acetyl-CoA	C23H34N7O17P3S	-4
coa[c]	Coenzyme A	C21H32N7O16P3S	-4
2mcit[m]	2-Methylcitrate	C7H10O7	-3
micit[m]	methylisocitrate	C7H10O7	-3
allphn[c]	Allophanate	C2H3N2O3	-1
urea[c]	Urea	CH4N2O	0
glu5p[c]	L-Glutamate 5-phosphate	C5H8NO7P	-2
agm[c]	Agmatine	C5H16N4	2
ptrc[c]	Putrescine	C4H14N2	2
1pyr5c[c]	1-Pyrroline-5-carboxylate	C5H6NO2	-1
pro-L[c]	L-Proline	C5H9NO2	0
orn[c]	Ornithine	C5H13N2O2	1
acetm[c]	Acetamide	C2H5NO	0
pad[c]	2-Phenylacetamide	C8H9NO	0
pac[c]	Phenylacetic acid	C8H7O2	-1
acglu[c]	N-Acetyl-L-glutamate	C7H9NO5	-2
e4hglu[m]	L-erythro-4-Hydroxyglutamate	C5H8NO5	-1
4h2oglt[m]	4-Hydroxy-2-oxoglutarate	C5H4O6	-2
acg5sa[m]	N-Acetyl-L-glutamate 5-semialdehyde	C7H10NO4	-1
acorn[m]	N2-Acetyl-L-ornithine	C7H14N2O3	0
glu5sa[m]	L-Glutamate 5-semialdehyde	C5H9NO3	0
h2o[m]	H2O	H2O	0
nadp[m]	Nicotinamide adenine dinucleotide phosphate	C21H25N7O17P3	-3
h[m]	H+	H	1

nadph[m]	Nicotinamide adenine dinucleotide phosphate - reduced	C21H26N7O17P3	-4
acglu[m]	N-Acetyl-L-glutamate	C7H9NO5	-2
acg5p[m]	N-Acetyl-L-glutamyl 5-phosphate	C7H9NO8P	-3
accoa[m]	Acetyl-CoA	C23H34N7O17P3S	-4
orn[m]	Ornithine	C5H13N2O2	1
e4hglu[c]	L-erythro-4-Hydroxyglutamate	C5H8NO5	-1
4h2oglt[c]	4-Hydroxy-2-oxoglutarate	C5H4O6	-2
glu5sa[c]	L-Glutamate 5-semialdehyde	C5H9NO3	0
nad[m]	Nicotinamide adenine dinucleotide	C21H26N7O14P2	-1
pro-L[m]	L-Proline	C5H9NO2	0
1pyr5c[m]	1-Pyrroline-5-carboxylate	C5H6NO2	-1
nadh[m]	Nicotinamide adenine dinucleotide - reduced	C21H27N7O14P2	-2
aproa[c]	3-Aminopropanal	C3H8NO	0
ala-B[c]	beta-Alanine	C3H7NO2	0
dtbt[c]	Dethiobiotin	C10H17N2O3	-1
s[c]	Sulfur	S	0
btn[c]	Biotin	C10H15N2O3S	-1
8aonn[c]	8-Amino-7-oxononanoate	C9H17NO3	0
amet[c]	S-Adenosyl-L-methionine	C15H23N6O5S	1
amob[c]	S-Adenosyl-4-methylthio-2-oxobutanoate	C15H19N5O6S	0

dann[c]	7,8-Diaminononanoate	C9H21N2O2	1
btd-RR[c]	(R,R)-2,3-Butanediol	C4H10O2	0
actn-R[c]	(R)-Acetoin	C4H8O2	[]
fum[m]	Fumarate	C4H2O4	-2
mal-L[m]	L-Malate	C4H4O5	-2
icit[m]	Isocitrate	C6H5O7	-3
co2[m]	CO2	CO2	0
icit[c]	Isocitrate	C6H5O7	-3
cit[m]	Citrate	C6H5O7	-3
mal-L[c]	L-Malate	C4H4O5	-2
hcys-L[c]	L-Homocysteine	C4H9NO2S	0
ahcys[c]	S-Adenosyl-L-homocysteine	C14H20N6O5S	0
met-L[c]	L-Methionine	C5H11NO2S	0
cys-L[c]	L-Cysteine	C3H7NO2S	0
suchms[c]	O-Succinyl-L-homoserine	C8H13NO6	-1
cyst-L[c]	L-Cystathionine	C7H14N2O4S	0
2obut[c]	2-Oxobutanoate	C4H5O3	-1
dkmpp[c]	2,3-diketo-5-methylthio-1-phosphopentane	C6H9O6PS	-2
2kmb[c]	2-keto-4-methylthiobutyrate	C5H7O3S	-1
for[c]	Formate	CH1O2	-1
lcyst[c]	L-Cysteate	C3H6NO5S	-1
taur[c]	Taurine	C2H7NO3S	-1
5mdru1p[c]	5-Methylthio-5-deoxy-D-ribulose 1-phosphate	C6H11O7PS	-2
aspsa[c]	L-Aspartate 4-semialdehyde	C4H7NO3	0
hom-L[c]	L-Homoserine	C4H9NO3	0

5mta[c]	5-Methylthioadenosine	C11H15N5O3S	0
5mdr1p[c]	5-Methylthio-5-deoxy-D-ribose 1-phosphate	C6H11O7PS	-2
ade[c]	Adenine	C5H5N5	0
4pasp[c]	4-Phospho-L-aspartate	C4H6NO7P	-2
adn[c]	Adenosine	C10H13N5O4	0
5mthf[c]	5-Methyltetrahydrofolate	C20H24N7O6	-1
thf[c]	5,6,7,8-Tetrahydrofolate	C19H21N7O6	-2
mhpglu[c]	5-Methyltetrahydropteroyltri-L-glutamate	C25H36N8O12	0
hpglu[c]	Tetrahydropteroyltri-L-glutamate	C24H34N8O12	0
ametam[c]	S-Adenosylmethioninamine	C14H24N6O3S	2
spmd[c]	Spermidine	C7H22N3	3
sprm[c]	Spermine	C10H30N4	-4
ser-L[c]	L-Serine	C3H7NO3	0
acser[c]	O-Acetyl-L-serine	C5H9NO4	0
h2s[c]	Hydrogen sulfide	H2S	0
achms[c]	O-Acetyl-L-homoserine	C6H11NO4	0
ch4s[c]	Methanethiol	CH4S	0
alaasp[c]	L-Alanine-L-Aspartate	C7H11N2O5	-1
glyglu[c]	Glycyl Glutamate	C7H11N2O5	-1
gly[c]	Glycine	C2H5NO2	0
glymet[c]	Glycyl Methionine	C7H14N2O3S	0
metala[c]	Methionine-Alanine	C8H15N2O3S	-1
glyasp[c]	Glycyl Aspartate	C6H9N2O5	-1

glypro[c]	Glycyl Proline	C7H12N2O3		0
gluala[c]	5-L-Glutamyl-L-alanine	C8H13N2O5		-1
alagln[c]	L-Alanine -L-Glutamine	C8H15N3O4		0
alaglu[c]	L-alanine-L-glutamate	C8H13N2O5		-1
alagly[c]	L-alanine-Glycine	C5H10N2O3		0
alahis[c]	L-alanine-L-Histidine	C9H14N4O3		0
his-L[c]	L-Histidine	C6H9N3O2		0
alaleu[c]	L-alanine-L-Leucine	C9H18N2O3		0
alathr[c]	L-alanine-L-Threonine	C7H14N2O4		0
thr-L[c]	L-Threonine	C4H9NO3		0
glyasn[c]	Glycyl Asparagine	C6H11N3O4		0
glygln[c]	Glycyl Glutamine	C7H13N3O4		0
13BDglcn[e]	1,3-beta-D-Glucan	C6H10O5		0
2mbald[e]	2-Methylbutanal	C5H10O	[]	
2mbtoh[e]	2-Methyl Butanol	C5H11O	[]	
2mppal[e]	2-Methyl 1- Propanal	C4H9O	[]	
2obut[e]	2-Oxobutanoate	C4H5O3		-1
2pg[e]	D-Glycerate 2-phosphate	C3H4O7P		-3
2pglyc[e]	2-Phosphoglycolate	C2H2O6P		-3
2phetoh[e]	2-Phenylethanol	C8H10O	[]	
3c3hmp[e]	3-Carboxy-3-hydroxy-4-methylpentanoate	C7H10O5		-2
3mbald[e]	3-Methylbutanal	C5H10O	[]	
3mop[e]	(S)-3-Methyl-2-oxopentanoate	C6H9O3		-1
3pg[e]	3-Phospho-D-glycerate	C3H4O7P		-3
4abut[e]	4-Aminobutanoate	C4H9NO2		0
4abz[e]	4-Aminobenzoate	C7H6NO2		-1
4hpro-LT[e]	trans-4-Hydroxy-L-proline	C5H9NO3		0
5aop[e]	5-Amino-4-oxopentanoate	C5H9NO3		0

6pgc[e]	6-Phospho-D-gluconate	C6H10O10P		-3
8aonn[e]	8-Amino-7-oxononanoate	C9H17NO3		0
ac[e]	Acetate	C2H3O2		-1
acac[e]	Acetoacetate	C4H5O3		-1
acald[e]	Acetaldehyde	C2H4O		0
acetm[e]	Acetamide	C2H5NO		0
ade[e]	Adenine	C5H5N5		0
adn[e]	Adenosine	C10H13N5O4		0
agm[e]	Agmatine	C5H16N4		2
akg[e]	2-Oxoglutarate	C5H4O5		-2
alaasp[e]	L-Alanine-L-Aspartate	C7H11N2O5		-1
ala-D[e]	D-Alanine	C3H7NO2		0
alagln[e]	L-Alanine -L-Glutamine	C8H15N3O4		0
alaglu[e]	L-alanine-L-glutamate	C8H13N2O5		-1
alagly[e]	L-alanine-Glycine	C5H10N2O3		0
alahis[e]	L-alanine-L-Histidine	C9H14N4O3		0
ala-L[e]	L-Alanine	C3H7NO2		0
alaleu[e]	L-alanine-L-Leucine	C9H18N2O3		0
alltn[e]	Allantoin	C4H6N4O3		0
alathr[e]	L-alanine-L-Threonine	C7H14N2O4		0
amet[e]	S-Adenosyl-L-methionine	C15H23N6O5S		1
amp[e]	AMP	C10H12N5O7P		-2
arab-D[e]	D-Arabinose	C5H10O5		0
arab-L[e]	L-Arabinose	C5H10O5		0
arg-L[e]	L-Arginine	C6H15N4O2		1
btd-RR[e]	(R,R)-2,3-Butanediol	C4H10O2		0
btn[e]	Biotin	C10H15N2O3S		-1
cellb[e]	Cellobiose	C12H22O11	[]	
cgly[e]	Cys-Gly	C5H10N2O3S		0
ch4s[e]	Methanethiol	CH4S		0

chol[e]	Choline	C5H14NO		1
cit[e]	Citrate	C6H5O7		-3
citr-L[e]	L-Citrulline	C6H13N3O3		0
cmp[e]	CMP	C9H12N3O8P		-2
co2[e]	CO2	CO2		0
csn[e]	Cytosine	C4H5N3O		0
cys-L[e]	L-Cysteine	C3H7NO2S		0
cyst-L[e]	L-Cystathionine	C7H14N2O4S		0
cytd[e]	Cytidine	C9H13N3O5		0
dad-2[e]	Deoxyadenosine	C10H13N5O3		0
dann[e]	7,8-Diaminononanoate	C9H21N2O2		1
dca[e]	Decanoate (n-C10:0)	C10H19O2		-1
dcyt[e]	Deoxycytidine	C9H13N3O4		0
ddca[e]	Dodecanoate (n-C12:0)	C12H23O2		-1
dgsn[e]	Deoxyguanosine	C10H13N5O4		0
dha[e]	Dihydroxyacetone	C3H6O3		0
din[e]	Deoxyinosine	C10H12N4O4		0
docosa[e]	Docosanoic acid	C22H44O2	[]	
dtmp[e]	dTMP	C10H13N2O8P		-2
dttp[e]	dTTP	C10H13N2O14P3		-4
duri[e]	Deoxyuridine	C9H12N2O5		0
eicosapen[e]	Eicosapentaenoic acid	C20H30O2	[]	
epist[e]	episterol	C28H46O		0
ergst[e]	Ergosterol	C28H44O		0
etha[e]	Ethanolamine	C2H8NO		1
etoh[e]	Ethanol	C2H6O		0
f6p[e]	D-Fructose 6-phosphate	C6H11O9P		-2
fe2[e]	Fe2+	Fe		2
fecost[e]	fecosterol	C28H46O		0
fmn[e]	FMN	C17H19N4O9P		-2
for[e]	Formate	CH1O2		-1

frmd[e]	Formamide	CH3NO	[]
fru[e]	D-Fructose	C6H12O6	0
fum[e]	Fumarate	C4H2O4	-2
g1p[e]	D-Glucose 1-phosphate	C6H11O9P	-2
g3pc[e]	sn-Glycero-3-phosphocholine	C8H20NO6P	0
g3pi[e]	sn-Glycero-3-phospho-1-inositol		-1
g6p[e]	D-Glucose 6-phosphate	C6H11O9P	-2
gal[e]	D-Galactose	C6H12O6	0
gam6p[e]	D-Glucosamine 6-phosphate	C6H13NO8P	-1
gcald[e]	Glycolaldehyde	C2H4O2	0
glc-D[e]	D-Glucose	C6H12O6	0
glcn-D[e]	D-Gluconic acid	C6H12O7	[]
gln-L[e]	L-Glutamine	C5H10N2O3	0
gluala[e]	5-L-Glutamyl-L-alanine	C8H13N2O5	-1
glu-L[e]	L-Glutamate	C5H8NO4	-1
glx[e]	Glyoxylate	C2H1O3	-1
gly[e]	Glycine	C2H5NO2	0
glyasn[e]	Glycyl Asparagine	C6H11N3O4	0
glyasp[e]	Glycyl Aspartate	C6H9N2O5	-1
glyc[e]	Glycerol	C3H8O3	0
glyc3p[e]	Glycerol 3-phosphate	C3H7O6P	-2
glyclt[e]	Glycolate	C2H3O3	-1
glygln[e]	Glycyl Glutamine	C7H13N3O4	0
glyglu[e]	Glycyl Glutamate	C7H11N2O5	-1
glymet[e]	Glycyl Methionine	C7H14N2O3S	0
glypro[e]	Glycyl Proline	C7H12N2O3	0
gmp[e]	GMP	C10H12N5O8P	-2
gsn[e]	Guanosine	C10H13N5O5	0
gthox[e]	Oxidized glutathione	C20H30N6O12S2	-2

gthrd[e]	Reduced glutathione	C10H16N3O6S		-1
gua[e]	Guanine	C5H5N5O		0
h[e]	H+	H		1
hdca[e]	Hexadecanoate (n-C16:0)	C16H31O2		-1
hdcea[e]	Hexadecenoate (n-C16:1)	C16H29O2		-1
hepdcea[e]	Heptadecenoate		[]	
hexa[e]	Hexanoate	C6H11O2		-1
hexc[e]	hexacosanoate (n-C26:0)	C26H51O2		-1
his-L[e]	L-Histidine	C6H9N3O2		0
hom-L[e]	L-Homoserine	C4H9NO3		0
iamoh[e]	3-Methylbutanol	C5H12O	[]	
ibutoh[e]	Isobutyl alcohol	C4H10O	[]	
ile-L[e]	L-Isoleucine	C6H13NO2		0
ind3eth[e]	Indole-3-ethanol	C10H11NO		0
inost[e]	myo-Inositol	C6H12O6		0
ins[e]	Inosine	C10H12N4O5		0
k[e]	potassium	K		1
lac-D[e]	D-Lactate	C3H5O3		-1
lac-L[e]	L-Lactate	C3H5O3		-1
lanost[e]	Lanosterol	C30H50O		0
lcyst[e]	L-Cysteate	C3H6NO5S		-1
leu-L[e]	L-Leucine	C6H13NO2		0
lys-L[e]	L-Lysine	C6H15N2O2		1
madg[e]	alpha-Methyl-D-glucoside	C7H14O6		0
mal-L[e]	L-Malate	C4H4O5		-2
malt[e]	Maltose	C12H22O11		0
maltr[e]	Maltotriose	C18H32O16		0
man[e]	D-Mannose	C6H12O6		0
man1p[e]	D-Mannose 1-phosphate	C6H11O9P		-2

man6p[e]	D-Mannose 6-phosphate	C6H11O9P		-2
mnl[e]	D-Mannitol	C6H14O6		0
mbdg[e]	beta-Methylglucoside	C7H14O6		0
meoh[e]	Methanol	CH4O	[]	
metala[e]	Methionine-Alanine	C8H15N2O3S		-1
met-L[e]	L-Methionine	C5H11NO2S		0
Na[e]	Sodium	Na	[]	
nac[e]	Nicotinate	C6H4NO2		-1
acgam[e]	N-Acetyl-D-glucosamine	C8H15NO6		0
nadp[e]	Nicotinamide adenine dinucleotide phosphate	C21H25N7O17P3		-3
nmn[e]	NMN	C11H14N2O8P		-1
o2[e]	O2	O2		0
oaa[e]	Oxaloacetate	C4H2O5		-2
ocdca[e]	octadecanoate (n-C18:0)	C18H35O2		-1
ocdcea[e]	octadecenoate (n-C18:1)	C18H33O2		-1
ocdcta[e]	Octadecatrienoate		[]	
ocdcya[e]	octadecadienoate (n-C18:2)	C18H31O2		-1
opro-L[e]	5-Oxoproline	C5H7NO3	[]	
orn[e]	Ornithine	C5H13N2O2		1
pacald[e]	Phenylacetaldehyde	C8H8O		0
pap[e]	Adenosine 3,5-bisphosphate	C10H11N5O10P2		-4
pc[e]	Phosphatidylcholine	C4040H7844N100O800P100		0
pep[e]	Phosphoenolpyruvate	C3H2O6P		-3
phe-L[e]	L-Phenylalanine	C9H11NO2		0
pi[e]	Phosphate	HO4P		-2
pnto-R[e]	(R)-Pantothenate	C9H16NO5		-1

ppi[e]	Diphosphate	HO7P2		-3
pro-L[e]	L-Proline	C5H9NO2		0
pser-L[e]	O-Phospho-L-serine	C3H6NO6P		-2
ptd1ino[e]	phosphatidyl-1D-myo-inositol	C4140H7644O1300P100		-100
ptrc[e]	Putrescine	C4H14N2		2
pyr[e]	Pyruvate	C3H3O3		-1
rbt[e]	Ribitol	C5H12O5	[]	
rham-L[e]	L-Rhamnose	C6H12O5		0
rib-D[e]	D-Ribose	C5H10O5		0
ribflv[e]	Riboflavin	C17H20N4O6		0
sbt-D[e]	D-Sorbitol	C6H14O6		0
sbt-L[e]	L-Sorbitol	C6H14O6		0
ser-L[e]	L-Serine	C3H7NO3		0
so3[e]	Sulfite	O3S		-2
so4[e]	Sulfate	O4S		-2
spmd[e]	Spermidine	C7H22N3		3
sprm[e]	Spermine	C10H30N4		-4
srb-L[e]	L-Sorbose	C6H12O6		0
succ[e]	Succinate	C4H4O4		-2
sucr[e]	Sucrose	C12H22O11		0
taur[e]	Taurine	C2H7NO3S		-1
thm[e]	Thiamin	C12H17N4OS		1
thmmp[e]	Thiamin monophosphate	C12H16N4O4PS		-1
thmpp[e]	Thiamine diphosphate	C12H16N4O7P2S		-2
thr-L[e]	L-Threonine	C4H9NO3		0
thym[e]	Thymine	C5H6N2O2		0
thymd[e]	Thymidine	C10H14N2O5		0
tmp[e]	Trimetaphosphate	O9P3		-3
tre[e]	Trehalose	C12H22O11		0
trp-L[e]	L-Tryptophan	C11H12N2O2		0
ttdca[e]	tetradecanoate (n-C14:0)	C14H27O2		-1

tyr-L[e]	L-Tyrosine	C9H11NO3		0
ump[e]	UMP	C9H11N2O9P		-2
ura[e]	Uracil	C4H4N2O2		0
urate[e]	Uric acid	C5H4N4O3		0
urea[e]	Urea	CH4N2O		0
uri[e]	Uridine	C9H12N2O6		0
val-L[e]	L-Valine	C5H11NO2		0
xan[e]	Xanthine	C5H4N4O2		0
xtsn[e]	Xanthosine	C10H12N4O6		0
xyl-D[e]	D-Xylose			0
xylt[e]	Xylitol	C5H12O5		0
zymst[e]	zymosterol	C27H44O		0
hexacoa[e]	Hexanoyl-CoA		[]	
dca[c]	Decanoate (n-C10:0)	C10H19O2		-1
dcacoa[c]	Decanoyl-CoA (n-C10:0CoA)	C31H50N7O17P3S		-4
ttdca[c]	tetradecanoate (n-C14:0)	C14H27O2		-1
tdcoa[c]	Tetradecanoyl-CoA (n-C14:0CoA)	C35H58N7O17P3S		-4
hdca[c]	Hexadecanoate (n-C16:0)	C16H31O2		-1
pmtcoa[c]	Palmitoyl-CoA (n-C16:0CoA)	C37H62N7O17P3S		-4
hdcea[c]	Hexadecenoate (n-C16:1)	C16H29O2		-1
hdceacoa[c]	Hexadecenoyl coA		[]	
hepdca[c]	Heptadecanoate		[]	
hepdcacoa[c]	Heptadecanoyl-CoA		[]	
hepdcea[c]	Heptadecenoate		[]	
hepdceacoa[c]	Heptadecenoyl-CoA		[]	
ocdca[c]	octadecanoate (n-C18:0)	C18H35O2		-1

stcoa[c]	Stearoyl-CoA (n-C18:0CoA)	C39H66N7O17P3S		-4
ocdcea[c]	octadecenoate (n-C18:1)	C18H33O2		-1
ocdceacoa[c]	octadecenoyl-coA		[]	
ocdcya[c]	octadecadienoate (n-C18:2)	C18H31O2		-1
ocdcyacoa[c]	Octadecynoyl-CoA-n-C182CoA	C39H62N7O17P3S	[]	
ocdcta[c]	Octadecatrienoate		[]	
ocdctacoa[c]	Octadecatrienoyl-CoA		[]	
eicosapen[c]	Eicosapentaenoic acid	C20H30O2	[]	
eicosapencoa[c]	Icosapentaenoyl-CoA	C41H64N7O17P3S	[]	
hexa[c]	Hexanoate	C6H11O2		-1
hexacoa[c]	Hexanoyl-CoA		[]	
malACP[m]	Malonyl-[acyl-carrier protein]	C14H22N2O10PRS		-2
octaACP[m]	Octanoyl-ACP		[]	
ACP[m]	acyl carrier protein	C11H21N2O7PRS		-1
dcaACP[m]	Decanoyl-ACP (n-C10:0ACP)	C21H39N2O8PRS		-1
ddcaACP[m]	Dodecanoyl-ACP (n-C12:0ACP)	C23H43N2O8PRS		-1
myrsACP[m]	Myristoyl-ACP (n-C14:0ACP)	C25H47N2O8PRS		-1
palmACP[m]	Palmitoyl-ACP (n-C16:0ACP)	C27H51N2O8PRS		-1
o2[m]	O2	O2		0
hdceaACP[m]	Hexadecenoyl ACP		[]	
ppACP[m]	Propanoyl-ACP		[]	
hepdcaACP[m]	Heptadecanoyl-ACP		[]	
hepdceaACP[m]	Heptadecenoyl-ACP		[]	
ocdcaACP[m]	Octadecanoyl-ACP (n-C18:0ACP)	C29H55N2O8PRS		-1

ocdceaACP[m]	octadecenoyl-ACP		[]	
ocdcyaACP[m]	Octadecynoyl-ACP (n-C18:2ACP)	C29H51N2O8PRS		-1
ocdctaACP[m]	Octadecatrienoyl-ACP		[]	
eicosaACP[m]	Eicosanoyl - ACP		[]	
eicosapenACP[m]	Eicosapentanoyl - ACP		[]	
docosaACP[m]	Docosanoyl-ACP		[]	
ttcACP[m]	tetracosanoyl-ACP		[]	
hexcACP[m]	Hexacosanoyl-ACP		[]	
acACP[m]	Acetyl-ACP	C13H23N2O8PRS		-1
hexaACP[m]	Hexanoyl-ACP		[]	
malcoa[m]	Malonyl-CoA	C24H33N7O19P3S		-5
ACP[c]	acyl carrier protein	C11H21N2O7PRS		-1
acACP[c]	Acetyl-ACP	C13H23N2O8PRS		-1
dcaACP[c]	Decanoyl-ACP (n-C10:0ACP)	C21H39N2O8PRS		-1
ddcaACP[c]	Dodecanoyl-ACP (n-C12:0ACP)	C23H43N2O8PRS		-1
ddca[c]	Dodecanoate (n-C12:0)	C12H23O2		-1
myrsACP[c]	Myristoyl-ACP (n-C14:0ACP)	C25H47N2O8PRS		-1
palmACP[c]	Palmitoyl-ACP (n-C16:0ACP)	C27H51N2O8PRS		-1
hdceaACP[c]	Hexadecenoyl ACP		[]	
hepdcaACP[c]	Heptadecanoyl-ACP		[]	
hepdceaACP[c]	Heptadecenoyl-ACP		[]	
ocdcaACP[c]	Octadecanoyl-ACP (n-C18:0ACP)	C29H55N2O8PRS		-1
ocdceaACP[c]	octadecenoyl-ACP		[]	
ocdcyaACP[c]	Octadecynoyl-ACP (n-C18:2ACP)	C29H51N2O8PRS		-1
ocdctaACP[c]	Octadecatrienoyl-ACP		[]	
eicosaACP[c]	Eicosanoyl - ACP		[]	

eicosa[c]	Eicosanoic acid	$C_{20}H_{40}O_2$	[]	
eicosapenACP[c]	Eicosapentanoyl - ACP		[]	
docosaACP[c]	Docosanoyl-ACP		[]	
docosa[c]	Docosanoic acid	$C_{22}H_{44}O_2$	[]	
ttcACP[c]	tetracosanoyl-ACP		[]	
ttc[c]	tetracosanoate (n-C24:0)	$C_{24}H_{47}O_2$		-1
hexcACP[c]	Hexacosanoyl-ACP		[]	
hexc[c]	hexacosanoate (n-C26:0)	$C_{26}H_{51}O_2$		-1
hexaACP[c]	Hexanoyl-ACP		[]	
octaACP[c]	Octanoyl-ACP		[]	
octa[c]	octanoate (n-C8:0)	$C_8H_{15}O_2$		-1
malcoa[c]	Malonyl-CoA	$C_{24}H_{33}N_7O_{19}P_3S$		-5
octacoa[c]	Octanoyl-CoA	$C_{29}H_{46}N_7O_{17}P_3S$		-4
ddcacoa[c]	Dodecanoyl-CoA (n-C12:0CoA)	$C_{33}H_{54}N_7O_{17}P_3S$		-4
ppcoa[c]	Propanoyl-CoA	$C_{24}H_{36}N_7O_{17}P_3S$		-4
eicosacoa[c]	Eicosanoyl-CoA	$C_{41}H_{74}N_7O_{17}P_3S$	[]	
docosacoa[c]	Docosanoyl-CoA	$C_{43}H_{78}N_7O_{17}P_3S$	[]	
ttccoa[c]	tetracosanoyl-CoA (n-C24:0CoA)	$C_{45}H_{78}N_7O_{17}P_3S$		-4
hexccoa[c]	Hexacosanoyl-CoA (n-C26:0CoA)	$C_{47}H_{82}N_7O_{17}P_3S$		-4
ppACP[c]	Propanoyl-ACP		[]	
ppcoa[m]	Propanoyl-CoA	$C_{24}H_{36}N_7O_{17}P_3S$		-4
fad[c]	Flavin adenine dinucleotide oxidized	$C_{27}H_{31}N_9O_{15}P_2$		-2
fadh2[c]	Flavin adenine dinucleotide reduced	$C_{27}H_{33}N_9O_{15}P_2$		-2
gtp[c]	GTP	$C_{10}H_{12}N_5O_{14}P_3$		-4

ahdt[c]	2-Amino-4-hydroxy-6-(erythro-1,2,3-trihydroxypropyl)dihydropteridine triphosphate	C9H12N5O13P3	-4
5fthf[c]	5-Formyltetrahydrofolate	C20H21N7O7	-2
methf[c]	5,10-Methenyltetrahydrofolate	C20H20N7O6	-1
dhf[c]	7,8-Dihydrofolate	C19H19N7O6	-2
4adcho[c]	4-amino-4-deoxychorismate	C10H10NO5	-1
4abz[c]	4-Aminobenzoate	C7H6NO2	-1
dhnpt[c]	Dihydroneopterin	C9H13N5O4	0
thfglu[c]	Tetrahydrofolyl-[Glu](2)	C24H27N8O9	-3
2ahhmp[c]	2-Amino-4-hydroxy-6-hydroxymethyl-7,8-dihydropteridine	C7H9N5O2	0
gcald[c]	Glycolaldehyde	C2H4O2	0
dhpt[c]	Dihydropteroate	C14H13N6O3	-1
2ahhmd[c]	2-Amino-4-hydroxy-6-hydroxymethyl-7,8-dihydropteridine diphosphate	C7H8N5O8P2	-3
mlthf[c]	5,10-Methylenetetrahydrofolate	C20H21N7O6	-2
chor[c]	chorismate	C10H8O6	-2
for[m]	Formate	CH1O2	-1
thf[m]	5,6,7,8-Tetrahydrofolate	C19H21N7O6	-2

10fthf[m]	10-Formyltetrahydrofolate	C20H21N7O7		-2
methf[m]	5,10-Methenyltetrahydrofolate	C20H20N7O6		-1
mlthf[m]	5,10-Methylenetetrahydrofolate	C20H21N7O6		-2
5fthf[m]	5-Formyltetrahydrofolate	C20H21N7O7		-2
10fthf[c]	10-Formyltetrahydrofolate	C20H21N7O7		-2
iasp[c]	Iminoaspartate	C4H3NO4		-2
fuc1p[c]	L-Fuculose 1-phosphate	C6H13O8P	[]	
dhap[c]	Dihydroxyacetone phosphate	C3H5O6P		-2
lald-S[c]	(S)-Lactaldehyde	C3H6O2	[]	
man[c]	D-Mannose	C6H12O6		0
man6p[c]	D-Mannose 6-phosphate	C6H11O9P		-2
fru[c]	D-Fructose	C6H12O6		0
bf6p[c]	beta-D-Fructose 2,6-bisphosphate	C6H14O12P2	[]	
sbt-D[c]	D-Sorbitol	C6H14O6		0
sbt-L[c]	L-Sorbitol	C6H14O6		0
srb-L[c]	L-Sorbose	C6H12O6		0
bf26p[c]	beta-D-Fructose 6-phosphate	C6H13O9P	[]	
man1p[c]	D-Mannose 1-phosphate	C6H11O9P		-2

gdpmann[c]	GDP-D-mannose	C16H23N5O16P2	-2
23drhamn[c]	2-Dehydro-3-deoxy-L-rhamnonate	C6H10O5	[]
lac-L[c]	L-Lactate	C3H5O3	-1
mnl[c]	D-Mannitol	C6H14O6	0
rhamn[c]	L-Rhamnonate	C6H12O6	[]
rham-L[c]	L-Rhamnose	C6H12O5	0
rhamlac[c]	L-Rhamnono-1,4-lactone	C6H12O5	[]
glc-D[c]	D-Glucose	C6H12O6	0
lact[c]	Lactose	C12H22O11	0
gal[c]	D-Galactose	C6H12O6	0
gal1p[c]	alpha-D-Galactose 1-phosphate	C6H11O9P	-2
udpg[c]	UDPglucose	C15H22N2O17P2	-2
g1p[c]	D-Glucose 1-phosphate	C6H11O9P	-2
udpgal[c]	UDPgalactose	C15H22N2O17P2	-2
gal14lac[c]	D-Galactono-1,4-lactone	C6H10O6	[]
maltr[c]	Maltotriose	C18H32O16	0
malt[c]	Maltose	C12H22O11	0
gthrd[c]	Reduced glutathione	C10H16N3O6S	-1
gthox[c]	Oxidized glutathione	C20H30N6O12S2	-2
cgly[c]	Cys-Gly	C5H10N2O3S	0
glucys[c]	gamma-L-Glutamyl-L-cysteine	C8H13N2O5S	-1
opro-L[c]	5-Oxoproline	C5H7NO3	[]
dolmanp[c]	Dolichyl phosphate D-mannose		-1
dolp[c]	Dolichol phosphate	C15H27O4P	-2
mannan[c]	Mannan	C6H10O5	0
ctp[c]	CTP	C9H12N3O14P3	-4
dolichol[c]	Dolichol	C15H28O	0

cdp[c]	CDP	C9H12N3O11P2	-3
gdp[c]	GDP	C10H12N5O11P2	-3
triglyc[c]	triglyceride	C5160H9566O600	0
12dgr[c]	1,2-Diacylglycerol	C3540H6644O500	0
dha[c]	Dihydroxyacetone	C3H6O3	0
glyc3p[c]	Glycerol 3-phosphate	C3H7O6P	-2
glyc[c]	Glycerol	C3H8O3	0
1ag3p[c]	1-Acyl-sn-glycerol 3-phosphate	C1920H3622O700P100	-200
1agly3p[c]	1-Acyl-glycerone 3-phosphate	C1920H3422O700P100	-200
pa[c]	Phosphatidate	C3540H6544O800P100	-200
pc[c]	Phosphatidylcholine	C4040H7844N100O800P100	0
1agpc[c]	acyl-glycerophosphocholine	C1243H2524N50O350P50	0
glyc3p[m]	Glycerol 3-phosphate	C3H7O6P	-2
dhap[m]	Dihydroxyacetone phosphate	C3H5O6P	-2
ps[m]	phosphatidylserine	C3840H7144N100O1000P100	-100
pe[m]	phosphatidylethanolamine	C3740H7244N100O800P100	0
ps[c]	phosphatidylserine	C3840H7144N100O1000P100	-100
pe[c]	phosphatidylethanolamine	C3740H7244N100O800P100	0
cdpdag[m]	CDPdiacylglycerol	C4440H7744N300O1500P200	-200
ser-L[m]	L-Serine	C3H7NO3	0
cmp[m]	CMP	C9H12N3O8P	-2
etha[c]	Ethanolamine	C2H8NO	1
ethamp[c]	Ethanolamine phosphate	C2H7NO4P	-1
cdpea[c]	CDPethanolamine	C11H19N4O11P2	-1
ptdmeeta[c]	Phosphatidyl-N-methylethanolamine	C3840H7444N100O800P100	0

ptd2meeta[c]	Phosphatidyl-N-dimethylethanolamine			0
cholp[c]	Choline phosphate	C5H13NO4P		-1
cdpchol[c]	CDPcholine	C14H25N4O11P2		-1
pgp[m]	Phosphatidylglycerophosphate	C3840H7144O1300P200		-300
pg[m]	Phosphatidylglycerol	C3840H7244O1000P100		-100
3php[c]	3-Phosphohydroxypyruvate	C3H2O7P		-3
pser-L[c]	O-Phospho-L-serine	C3H6NO6P		-2
glx[c]	Glyoxylate	C2H1O3		-1
phom[c]	O-Phospho-L-homoserine	C4H8NO6P		-2
gly[m]	Glycine	C2H5NO2		0
lpam[m]	Lipoamide	C8H15NOS2		0
alpam[m]	S-aminomethyldihydrolipoamide	C9H21N2OS2		1
dhlam[m]	Dihydrolipoamide	C8H17NOS2		0
nh4[m]	Ammonium	H4N		1
3pg[c]	3-Phospho-D-glycerate	C3H4O7P		-3
5aop[m]	5-Amino-4-oxopentanoate	C5H9NO3		0
g6p[c]	D-Glucose 6-phosphate	C6H11O9P		-2
13dpg[c]	3-Phospho-D-glyceroyl phosphate	C3H4O10P2		-4
23dph[c]	2,3-Bisphospho-D-glycerate	C3H8O10P2	[]	
2pg[c]	D-Glycerate 2-phosphate	C3H4O7P		-3
fdp[c]	D-Fructose 1,6-bisphosphate	C6H10O12P2		-4

pep[c]	Phosphoenolpyruvate	C3H2O6P		-3
g3p[c]	Glyceraldehyde 3-phosphate	C3H5O6P		-2
frmd[c]	Formamide	CH3NO	[]	
2pglyc[c]	2-Phosphoglycolate	C2H2O6P		-3
glyclt[c]	Glycolate	C2H3O3		-1
hisp[c]	L-Histidinol phosphate	C6H11N3O4P		-1
histd[c]	L-Histidinol	C6H12N3O		1
prfp[c]	1-(5-Phosphoribosyl)-5-[(5-phosphoribosylamino)methylideneamino]imidazole-4-carboxamide	C15H21N5O15P2		-4
prlp[c]	5-[(5-phospho-1-deoxyribulos-1-ylamino)methylideneamino]-1-(5-phosphoribosyl)imidazole-4-carboxamide	C15H21N5O15P2		-4
aicar[c]	5-Amino-1-(5-Phospho-D-ribosyl)imidazole-4-carboxamide	C9H13N4O8P		-2
eig3p[c]	D-erythro-1-(Imidazol-4-yl)glycerol 3-phosphate	C6H9N2O6P		-2
prbatp[c]	1-(5-Phosphoribosyl)-ATP	C15H19N5O20P4		-6
prbamp[c]	1-(5-Phosphoribosyl)-AMP	C15H19N5O14P2		-4
imacp[c]	3-(Imidazol-4-yl)-2-oxopropyl phosphate	C6H7N2O5P		-2
cdpdag[c]	CDPdiacylglycerol	C4440H7744N300O1500P200		-200
inost[c]	myo-Inositol	C6H12O6		0
cmp[c]	CMP	C9H12N3O8P		-2

ptd1ino[c]	phosphatidyl-1D-myo-inositol	C4140H7644O1300P100		-100
mi1p-D[c]	1D-myo-Inositol 1-phosphate	C6H11O9P		-2
ptd3ino[c]	1-Phosphatidyl-1D-myo-inositol 3-phosphate	C4140H7544O1600P200		-300
ptd145bp[c]	1-Phosphatidyl-D-myo-inositol 4,5-bisphosphate	C4140H7444O1900P300		-500
mi145tp-D[c]	D-myo-Inositol 1,4,5-trisphosphate	C6H15O15P3	[]	
ptd4ino[c]	1-Phosphatidyl-1D-myo-inositol 4-phosphate	C4140H7544O1600P200		-300
mi14bp-D[c]	D-myo-Inositol 1,4-bisphosphate	C6H14O12P2	[]	
ptd134bp[c]	phosphatidyl-1D-myo-inositol 3,4-bisphosphate	C4140H7444O1900P300		-500
2ins[c]	2-Inosose	C6H10O6		0
hicit[m]	Homoisocitrate	C7H7O7		-3
oxag[m]	Oxaloglutarate	C7H5O7		-3
23dhdp[c]	2,3-Dihydrodipicolinate	C7H5NO4		-2
2oxoadp[c]	2-Oxoadipate	C6H6O5		-2
L2aadp[c]	L-2-Aminoadipate	C6H10NO4		-1
L2aadp6sa[c]	L-2-Aminoadipate 6-semialdehyde	C6H11NO3		0
saccrp-L[c]	L-Saccharopine	C11H19N2O6		-1
glucoa[m]	Glutaryl-CoA	C26H42N7O19P3S	[]	
lys-L[c]	L-Lysine	C6H15N2O2		1
b124tc[m]	But-1-ene-1,2,4-tricarboxylate	C7H5O6		-3

2oxoadp[m]	2-Oxoadipate	C6H6O5	-2
rnam[c]	N-Ribosylnicotinamide		1
nmn[c]	NMN	C11H14N2O8P	-1
adprib[c]	ADPribose	C15H23N5O14P2	-2
ncam[c]	Nicotinamide	C6H6N2O	0
adprib[m]	ADPribose	C15H23N5O14P2	-2
ncam[m]	Nicotinamide	C6H6N2O	0
nac[m]	Nicotinate	C6H4NO2	-1
r1p[c]	alpha-D-Ribose 1-phosphate	C5H9O8P	-2
quln[c]	Quinolinate	C7H3NO4	-2
nac[c]	Nicotinate	C6H4NO2	-1
nicrnt[c]	Nicotinate D-ribonucleotide	C11H12NO9P	-2
dnad[c]	Deamino-NAD+	C21H24N6O15P2	-2
aprop[c]	alpha-Aminopropiononitrile	C3H7N2	1
acybut[c]	gamma-Amino-gamma-cyanobutanoate	C5H8N2O2	0
trdrd[c]	Reduced thioredoxin	XH2	0
datp[c]	dATP	C10H12N5O12P3	-4
trdox[c]	Oxidized thioredoxin	X	0
dgtp[c]	dGTP	C10H12N5O13P3	-4
dctp[c]	dCTP	C9H12N3O13P3	-4
dutp[c]	dUTP	C9H11N2O14P3	-4
hxan[c]	Hypoxanthine	C5H4N4O	0
2dr1p[c]	2-Deoxy-D-ribose 1-phosphate	C5H9O7P	-2
2dr5p[c]	2-Deoxy-D-ribose 5-phosphate	C5H9O7P	-2
csn[c]	Cytosine	C4H5N3O	0
r5p[c]	alpha-D-Ribose 5-phosphate	C5H9O8P	-2

gsn[c]	Guanosine	C10H13N5O5		0
gmp[c]	GMP	C10H12N5O8P		-2
ins[c]	Inosine	C10H12N4O5		0
imp[c]	IMP	C10H11N4O8P		-2
thymd[c]	Thymidine	C10H14N2O5		0
dtmp[c]	dTMP	C10H13N2O8P		-2
thym[c]	Thymine	C5H6N2O2		0
q6[m]	Ubiquinone-6	C39H58O4		0
q6h2[m]	Ubiquinol-6	C39H60O4		0
ficytc[m]	Ferricytochrome c	C42H52FeN8O6S2		1
focytc[m]	Ferrocytochrome c	C42H53FeN8O6S2		1
ficytc[c]	Ferricytochrome c	C42H52FeN8O6S2		1
focytc[c]	Ferrocytochrome c	C42H53FeN8O6S2		1
ppi[m]	Diphosphate	HO7P2		-3
k[c]	potassium	K		1
Na[c]	Sodium	Na	[]	
pppi[c]	Inorganic triphosphate	HO10P3		-4
tmp[c]	Trimetaphosphate	O9P3		-3
N1aspmd[c]	N1-Acetylspermidine	C9H23N3O		-2
aprut[c]	N-Acetylputrescine	C6H15N2O		-1
N1sprm[c]	N1-Acetylspermine	C12H31N4O		-3
2dhp[c]	2-Dehydropantoate	C6H9O4		-1
dpcoa[c]	Dephospho-CoA	C21H33N7O13P2S		-2
pan4p[c]	Pantetheine 4-phosphate	C11H21N2O7PS		-2
4ppan[c]	D-4-Phosphopantothenate	C9H15NO8P		-3
4ppcys[c]	N-((R)-4-Phosphopantothenoyl)-L-cysteine	C12H20N2O9PS		-3
2dhp[m]	2-Dehydropantoate	C6H9O4		-1
pant-R[m]	(R)-Pantoate	C6H11O4		-1
pnto-R[c]	(R)-Pantothenate	C9H16NO5		-1
pant-R[c]	(R)-Pantoate	C6H11O4		-1

23dhmb[m]	(R)-2,3-Dihydroxy-3-methylbutanoate	C5H9O4		-1
3mob[m]	3-Methyl-2-oxobutanoate	C5H7O3		-1
alac-S[m]	(S)-2-Acetolactate	C5H7O4		-1
rbt[c]	Ribitol	C5H12O5	[]	
xylu-D[c]	D-Xylulose	C5H10O5		0
xu5p-D[c]	D-Xylulose 5-phosphate	C5H9O8P		-2
xylt[c]	Xylitol	C5H12O5		0
xyl-D[c]	D-Xylose			0
arab-D[c]	D-Arabinose	C5H10O5		0
arab-L[c]	L-Arabinose	C5H10O5		0
abt-L[c]	L-Arabitol	C5H12O5		0
rib-D[c]	D-Ribose	C5H10O5		0
xylu-L[c]	L-Xylulose	C5H10O5		0
ribu-D[c]	D-Ribulose	C5H10O5		0
ru5p-D[c]	D-Ribulose 5-phosphate	C5H9O8P		-2
g15lac[c]	D-Glucono-1,5-lactone	C6H10O6	[]	
glcn-D[c]	D-Gluconic acid	C6H12O7	[]	
2glcna[c]	2-Dehydro-D-gluconate	C6H10O7	[]	
e4p[c]	D-Erythrose 4-phosphate	C4H7O7P		-2
s7p[c]	Sedoheptulose 7-phosphate	C7H13O10P		-2
6pgl[c]	6-phospho-D-glucono-1,5-lactone	C6H9O9P		-2
6pgc[c]	6-Phospho-D-gluconate	C6H10O10P		-3
pacald[c]	Phenylacetaldehyde	C8H8O		0
indpyr[c]	Indolepyruvate	C11H8NO3		-1
id3acald[c]	Indole-3-acetaldehyde	C10H9NO		0

phpyr[c]	Phenylpyruvate	C9H7O3	-1
pphn[c]	Prephenate	C10H8O6	-2
anth[c]	Anthranilate	C7H6NO2	-1
pran[c]	N-(5-Phospho-D-ribosyl)anthranilate	C12H13NO9P	-3
2cpr5p[c]	1-(2-Carboxyphenylamino)-1-deoxy-D-ribulose 5-phosphate	C12H13NO9P	-3
tyr-L[m]	L-Tyrosine	C9H11NO3	0
34hpp[m]	3-(4-Hydroxyphenyl)pyruvate	C9H7O4	-1
34hpp[c]	3-(4-Hydroxyphenyl)pyruvate	C9H7O4	-1
3dhq[c]	3-Dehydroquinate	C7H9O6	-1
3dhsk[c]	3-Dehydroshikimate	C7H7O5	-1
tyr-L[c]	L-Tyrosine	C9H11NO3	0
phe-L[c]	L-Phenylalanine	C9H11NO2	0
trp-L[c]	L-Tryptophan	C11H12N2O2	0
3ig3p[c]	C-(3-Indolyl)-glycerol 3-phosphate	C11H12NO6P	-2
2dda7p[c]	2-Dehydro-3-deoxy-D-arabino-heptonate 7-phosphate	C7H10O10P	-3
skm5p[c]	Shikimate 5-phosphate	C7H8O8P	-3
3psme[c]	5-O-(1-Carboxyvinyl)-3-phosphoshikimate	C10H9O10P	-4
skm[c]	Shikimate	C7H9O5	-1
g3pc[c]	sn-Glycero-3-phosphocholine	C8H20NO6P	0
octa[e]	octanoate (n-C8:0)	C8H15O2	-1

clpn[m]	Cardiolipin	C7380H13688O1700P200		-200
ctp[m]	CTP	C9H12N3O14P3		-4
pa[m]	Phosphatidate	C3540H6544O800P100		-200
uppg3[c]	Uroporphyrinogen III	C40H36N4O16		-8
dscl[c]	dihydrosirohydrochlorin	C42H41N4O16		-7
fe2[m]	Fe2+	Fe		2
ppp9[m]	Protoporphyrin	C34H32N4O4		-2
pheme[m]	Protoheme	C34H30FeN4O4		-2
cpppg3[c]	Coproporphyrinogen III	C36H40N4O8		-4
ppbng[c]	Porphobilinogen	C10H13N2O4		-1
hmbil[c]	Hydroxymethylbilane	C40H38N4O17		-8
frdp[m]	Farnesyl diphosphate	C15H25O7P2		-3
hemeO[m]	Heme O	C49H56FeN4O5		-2
5aop[c]	5-Amino-4-oxopentanoate	C5H9NO3		0
pppg9[m]	Protoporphyrinogen IX	C34H38N4O4		-2
scl[c]	sirohydrochlorin	C42H39N4O16		-7
fe2[c]	Fe2+	Fe		2
sheme[c]	Siroheme	C42H36FeN4O16		-8
pppg9[c]	Protoporphyrinogen IX	C34H38N4O4		-2
hemeA[m]	Heme A	C49H54FeN4O6		-6
hppcoa[c]	3-Hydroxypropionyl-CoA;	C24H40N7O18P3S	[]	
ppecoa[c]	Propenoyl-CoA	C24H38N7O17P3S	[]	
msa[c]	Malonate semialdehyde	C3H3O3		-1
2mop[m]	2-Methyl-3-oxopropanoate	C4H5O3		-1
dadp[c]	dADP	C10H12N5O9P2		-3
dgdp[c]	dGDP	C10H12N5O10P2		-3
xmp[c]	Xanthosine 5-phosphate	C10H11N4O9P		-2

xtsn[c]	Xanthosine	C10H12N4O6		0
ump[c]	UMP	C9H11N2O9P		-2
uri[c]	Uridine	C9H12N2O6		0
dcmp[c]	dCMP	C9H12N3O7P		-2
dcyt[c]	Deoxycytidine	C9H13N3O4		0
damp[c]	dAMP	C10H12N5O6P		-2
dad-2[c]	Deoxyadenosine	C10H13N5O3		0
dgmp[c]	dGMP	C10H12N5O7P		-2
dgsn[c]	Deoxyguanosine	C10H13N5O4		0
gar[c]	N1-(5-Phospho-D-ribosyl)glycinamide	C7H14N2O8P		-1
fgam[c]	N2-Formyl-N1-(5-phospho-D-ribosyl)glycinamide	C8H13N2O9P		-2
alltn[c]	Allantoin	C4H6N4O3		0
alltt[c]	Allantoate	C4H7N4O4		-1
gua[c]	Guanine	C5H5N5O		0
xan[c]	Xanthine	C5H4N4O2		0
urate[c]	Uric acid	C5H4N4O3		0
ap4a[c]	P1,P4-Bis(5-adenosyl) tetraphosphate	C20H24N10O19P4		-4
gp4g[c]	P1,P4-Bis(5-guanosyl) tetraphosphate	C20H24N10O21P4		-4
up4u[c]	P1,P4-Bis(5-uridyl) tetraphosphate	C18H26N4O23P4	[]	
xp4x[c]	P1,P4-Bis(5-xanthosyl) tetraphosphate	C20H26N8O23P4	[]	
xtp[c]	XTP	C10H11N4O15P3		-4
urdglyc[c]	Ureidoglycolate	C3H5N2O4		-1
ap4g[c]	P1-(5-adenosyl),P4-(5-guanosyl) tetraphosphate	C20H24N10O20P4		-4

5aizc[c]	5-amino-1-(5-phospho-D-ribosyl)imidazole-4-carboxylate	C9H11N3O9P	-3
25aics[c]	(S)-2-[5-Amino-1-(5-phospho-D-ribosyl)imidazole-4-carboxamido]succinate	C13H15N4O12P	-4
amp[m]	AMP	C10H12N5O7P	-2
camp[c]	cAMP	C10H11N5O6P	-1
idp[c]	IDP	C10H11N4O11P2	-3
itp[c]	ITP	C10H11N4O14P3	-4
fprica[c]	5-Formamido-1-(5-phospho-D-ribosyl)imidazole-4-carboxamide	C10H13N4O9P	-2
fpram[c]	2-(Formamido)-N1-(5-phospho-D-ribosyl)acetamidine	C8H15N3O8P	-1
air[c]	5-amino-1-(5-phospho-D-ribosyl)imidazole	C8H12N3O7P	-2
dcamp[c]	N6-(1,2-Dicarboxyethyl)-AMP	C14H14N5O11P	-4
duri[c]	Deoxyuridine	C9H12N2O5	0
dump[c]	dUMP	C9H11N2O8P	-2
aps[c]	Adenosine 5-phosphosulfate	C10H12N5O10PS	-2
din[c]	Deoxyinosine	C10H12N4O4	0
cytd[c]	Cytidine	C9H13N3O5	0
dtdp[c]	dTDP	C10H13N2O11P2	-3
dttp[c]	dTTP	C10H13N2O14P3	-4
dudp[c]	dUDP	C9H11N2O11P2	-3
dcdp[c]	dCDP	C9H12N3O10P2	-3
ura[c]	Uracil	C4H4N2O2	0

dhor-S[c]	(S)-Dihydroorotate	C5H5N2O4		-1
cbasp[c]	N-Carbamoyl-L-aspartate	C5H6N2O5		-2
orot5p[c]	Orotidine 5-phosphate	C10H10N2O11P		-3
orot[c]	Orotate	C5H3N2O4		-1
mthgxl[c]	Methylglyoxal	C3H4O2		0
lgt-S[c]	(R)-S-Lactoylglutathione	C13H20N3O8S		-1
lac-D[c]	D-Lactate	C3H5O3		-1
aacoa[c]	Acetoacetyl-CoA	C25H36N7O18P3S		-4
acald[m]	Acetaldehyde	C2H4O		0
ac[m]	Acetate	C2H3O2		-1
lac-L[m]	L-Lactate	C3H5O3		-1
3c3hmp[m]	3-Carboxy-3-hydroxy-4-methylpentanoate	C7H10O5		-2
hco3[m]	Bicarbonate	CHO3		-1
lac-D[m]	D-Lactate	C3H5O3		-1
2hpmhmbq[m]	2-Hexaprenyl-3-methyl-5-hydroxy-6-methoxy-1,4-benzoquinone	C38H56O4	[]	
amet[m]	S-Adenosyl-L-methionine	C15H23N6O5S		1
ahcys[m]	S-Adenosyl-L-homocysteine	C14H20N6O5S		0
3dh5hpb[m]	3-Hexaprenyl-4,5-dihydroxybenzoate	C37H54O4	[]	
3hph5mb[m]	3-Hexaprenyl-4-hydroxy-5-methoxybenzoate	C38H56O4	[]	
2hp6mp[m]	2-Hexaprenyl-6-methoxyphenol	C37H56O2	[]	

2hp6mbq[m]	2-Hexaprenyl-6-methoxy-1,4-benzoquinone	C37H54O3	[]	
2hpmmbq[m]	2-Hexaprenyl-3-methyl-6-methoxy-1,4-benzoquinone	C38H56O3	[]	
ipdp[m]	Isopentenyl diphosphate	C5H9O7P2		-3
pendp[m]	all-trans-Pentaprenyl diphosphate	C25H41O7P2		-3
hexdp[m]	all-trans-Hexaprenyl diphosphate	C30H52O7P2	[]	
4hbz[m]	4-Hydroxybenzoate	C7H5O3		-1
3ophb_5[m]	3-Hexaprenyl-4-hydroxybenzoate	C37H53O3		-1
25dhpp[c]	2,5-Diamino-6-hydroxy-4-(5-phosphoribosylamino)-pyrimidine	C9H14N5O8P		-2
fmn[c]	FMN	C17H19N4O9P		-2
4r5au[c]	4-(1-D-Ribitylamino)-5-aminouracil	C9H16N4O6		0
db4p[c]	3,4-dihydroxy-2-butanone 4-phosphate	C4H7O6P		-2
dmlz[c]	6,7-Dimethyl-8-(1-D-ribityl)lumazine	C13H18N4O6		0
ribflv[c]	Riboflavin	C17H20N4O6		0
5apru[c]	5-Amino-6-(5-phosphoribosylamino)uracil	C9H13N4O9P		-2
5aprbu[c]	5-Amino-6-(5-phosphoribitylamino)uracil	C9H15N4O9P		-2

sph1p[c]	Sphinganine 1-phosphate	C18H39NO5P		-1
sphgn[c]	Sphinganine	C18H40NO2		1
psph1p[c]	Phytosphingosine 1-phosphate	C18H39NO6P		-1
psphings[c]	Phytosphingosine	C18H40NO3		1
gcylcer[c]	Glucosylceramide	C25H46NO8R	[]	
lactcer[c]	Lactosylceramide	C31H56NO13R	[]	
cer1_24[c]	Ceramide-1 (Sphinganine:n-C24:0)	C42H85NO3		0
cer1_26[c]	Ceramide-1 (Sphinganine:n-C26:0)	C44H89NO3		0
cer2_24[c]	Ceramide-2 (Phytosphingosine:n-C24:0)	C42H85NO4		0
cer2_26[c]	Ceramide-2 (Phytosphingosine:n-C26:0)	C44H89NO4		0
ipc124[c]	Inositol phosphorylceramide, ceramide-1 (24C)	C4800H9500N100O1100P100		-100
ipc126[c]	Inositol phosphorylceramide, ceramide-1 (26C)	C5000H9900N100O1100P100		-100
ipc224[c]	Inositol-phosphorylceramide-ceramide-2-24C	C4800H9500N100O1200P100	[]	
ipc226[c]	Inositol phosphorylceramide, ceramide-2 (26C)	C5000H9900N100O1200P100		-100
mip2c124[c]	mannose-(inositol-P)2-ceramide, ceramide-1 (24C)	C6000H11500N100O2400P200		-200

man2mi1p-D[c]	D-Mannose Inositol Phosphate 2		[]	
mip2c126[c]	mannose-(inositol-P)2-ceramide, ceramide-1 (26C)	C6200H11900N100O2400P200		-200
mip2c224[c]	mannose-(inositol-P)2-ceramide, ceramide-2 (24C)	C6000H11500N100O2500P200		-200
mip2c226[c]	mannose-(inositol-P)2-ceramide, ceramide-2 (26C)	C6200H11900N100O2500P200		-200
mipc124[c]	mannose-inositol phosphorylceramide, ceramide-1 (24C)	C5400H10500N100O1600P100		-100
manmi1p-D[c]	D-Mannose Inositol Phosphate		[]	
mipc126[c]	mannose-inositol phosphorylceramide, ceramide-1 (26C)	C5600H10900N100O1600P100		-100
mipc224[c]	mannose-inositol phosphorylceramide, ceramide-2 (24C)	C5400H10500N100O1700P100		-100
mipc226[c]	mannose-inositol phosphorylceramide, ceramide-2 (26C)	C5600H10900N100O1700P100		-100
cer3[c]	N-Acylsphingosine	C19H36NO3R	[]	
3dsphgn[c]	3-Dehydrosphinganine	C18H38NO2		1
spmylin[c]	Sphingomyelin	C24H49N2O6PR	[]	
cer3_24[c]	Ceramide-3 (Phytosphingosine:n-C24:0OH)	C42H85NO5		0
cer3_26[c]	Ceramide-3 (Phytosphingosine:n-C26:0OH)	C44H89NO5		0

2hhxdal[c]	2-Hydroxy-hexadecanal	C16H32O2	0
hxdcal[c]	Hexadecanal	C16H32O	0
epist[c]	episterol	C28H46O	0
ergtrol[c]	ergosta-5,7,24(28)-trienol	C28H44O	0
ergtetrol[c]	Ergosta-5,7,22,24,(28)-tetraen-3beta-ol	C28H42O	0
ergst[c]	Ergosterol	C28H44O	0
44mctr[c]	4,4-dimethylcholesta-8,14,24-trienol	C29H46O	0
44mzym[c]	4,4-dimethyllanosterol	C29H48O	0
4mzym_int2[c]	3-Keto-4-methylzymosterol	C28H44O	0
4mzym[c]	4-methylzymosterol	C28H46O	0
zym_int2[c]	zymosterol intermediate 2	C27H42O	0
zymst[c]	zymosterol	C27H44O	0
lanost[c]	Lanosterol	C30H50O	0
Ssq23epx[c]	(S)-Squalene-2,3-epoxide	C30H50O	0
4mzym_int1[c]	4alpha-Methylzymosterol-4-carboxylate	C29H46O3	0
zym_int1[c]	zymosterol intermediate 1	C28H44O3	0
fecost[c]	fecosterol	C28H46O	0
sql[c]	Squalene	C30H50	0
frdp[c]	Farnesyl diphosphate	C15H25O7P2	-3
glycogen[c]	glycogen	C6H10O5	0
13BDglcn[c]	1,3-beta-D-Glucan	C6H10O5	0
sucr[c]	Sucrose	C12H22O11	0
tre6p[c]	alpha,alpha-Trehalose 6-phosphate	C12H21O14P	-2

tre[c]	Trehalose	C12H22O11	0
14glun[c]	(1,4-alpha-D-Glucosyl)n	C6H12O6	0
so3[c]	Sulfite	O3S	-2
hmgcoa[c]	Hydroxymethylglutaryl-CoA	C27H39N7O20P3S	-5
amacald[c]	Aminoacetaldehyde	C2H5NO	0
ggdp[c]	Geranylgeranyl diphosphate	C20H33O7P2	-3
ipdp[c]	Isopentenyl diphosphate	C5H9O7P2	-3
pendp[c]	all-trans-Pentaprenyl diphosphate	C25H41O7P2	-3
mev-R[c]	(R)-Mevalonate	C6H11O4	-1
5pmev[c]	(R)-5-Phosphomevalonate	C6H10O7P	-3
dmpp[c]	Dimethylallyl diphosphate	C5H9O7P2	-3
grdp[c]	Geranyl diphosphate	C10H17O7P2	-3
5dpmev[c]	(R)-5-Diphosphomevalonate	C6H10O10P2	-4
thm[c]	Thiamin	C12H17N4OS	1
thmpp[c]	Thiamine diphosphate	C12H16N4O7P2S	-2
thmtp[c]	Thiamin triphosphate	C12H16N4O10P3S	-3
4mpetz[c]	4-Methyl-5-(2-phosphoethyl)-thiazole	C6H8NO4PS	-2
4ahmmp[c]	4-Amino-5-hydroxymethyl-2-methylpyrimidine	C6H9N3O	0
4ampm[c]	4-Amino-2-methyl-5-phosphomethylpyrimidine	C6H8N3O4P	-2

2mahmp[c]	2-Methyl-4-amino-5-hydroxymethylpyrimidine diphosphate	C6H8N3O7P2		-3
4mhetz[c]	4-Methyl-5-(2-hydroxyethyl)-thiazole	C6H9NOS		0
thmmp[c]	Thiamin monophosphate	C12H16N4O4PS		-1
cit[c]	Citrate	C6H5O7		-3
pep[m]	Phosphoenolpyruvate	C3H2O6P		-3
2obut[m]	2-Oxobutanoate	C4H5O3		-1
3c3hmp[c]	3-Carboxy-3-hydroxy-4-methylpentanoate	C7H10O5		-2
3c4mop[c]	3-Carboxy-4-methyl-2-oxopentanoate	C7H8O5		-2
3c4mop[m]	3-Carboxy-4-methyl-2-oxopentanoate	C7H8O5		-2
3mop[c]	(S)-3-Methyl-2-oxopentanoate	C6H9O3		-1
3mop[m]	(S)-3-Methyl-2-oxopentanoate	C6H9O3		-1
4hpro-LT[c]	trans-4-Hydroxy-L-proline	C5H9NO3		0
4hpro-LT[m]	trans-4-Hydroxy-L-proline	C5H9NO3		0
acac[c]	Acetoacetate	C4H5O3		-1
acac[m]	Acetoacetate	C4H5O3		-1
hibut[m]	(S)-3-Hydroxyisobutyrate	C4H8O3	[]	
hibut[c]	(S)-3-Hydroxyisobutyrate	C4H8O3	[]	
ibcoa[m]	Isobutyryl-CoA	C25H38N7O17P3S		-4
ibcoa[c]	Isobutyryl-CoA	C25H38N7O17P3S		-4
ile-L[m]	L-Isoleucine	C6H13NO2		0
ile-L[c]	L-Isoleucine	C6H13NO2		0

ivcoa[m]	Isovaleryl-CoA	C26H40N7O17P3S		-4
ivcoa[c]	Isovaleryl-CoA	C26H40N7O17P3S		-4
thr-L[m]	L-Threonine	C4H9NO3		0
val-L[m]	L-Valine	C5H11NO2		0
leu-L[c]	L-Leucine	C6H13NO2		0
lact[e]	Lactose	C12H22O11		0
2mbald[c]	2-Methylbutanal	C5H10O	[]	
2mbtoh[c]	2-Methyl Butanol	C5H11O	[]	
2mppal[c]	2-Methyl 1- Propanal	C4H9O	[]	
2phetoh[c]	2-Phenylethanol	C8H10O	[]	
3mbald[c]	3-Methylbutanal	C5H10O	[]	
g3pi[c]	sn-Glycero-3-phospho-1-inositol			-1
iamoh[c]	3-Methylbutanol	C5H12O	[]	
ibutoh[c]	Isobutyl alcohol	C4H10O	[]	
ind3eth[c]	Indole-3-ethanol	C10H11NO		0
Lkynr[c]	L-Kynurenine	C10H12N2O3		0
hLkynr[c]	3-Hydroxy-L-kynurenine	C10H12N2O4		0
iad[c]	Indole-3-acetamide	C10H10N2O		0
ind3ac[c]	Indole-3-acetate	C10H8NO2		-1
Lfmkynr[c]	L-Formylkynurenine	C11H12N2O4		0
3hanthrn[c]	3-Hydroxyanthranilate	C7H7NO3		0
cmusa[c]	2-Amino-3-carboxymuconate semialdehyde	C7H6NO5		-1
am6sa[c]	2-Aminomuconate 6-semialdehyde	C6H7NO3		0
amuco[c]	2-Aminomuconate	C6H7NO4		0
ind3acnl[c]	Indole-3-acetonitrile	C10H8N2		0
hmgth[c]	hydroxymethylglutathione	C11H18N3O7S		-1
Sfglutth[c]	S-Formylglutathione	C11H16N3O7S		-1
hgentis[c]	Homogentisate	C8H7O4		-1

4fumacac[c]	4-Fumarylacetoacetate	C8H6O6		-2
4mlacac[c]	4-Maleylacetoacetate	C8H6O6		-2
2ahbut[m]	(S)-2-Aceto-2-hydroxybutanoate	C6H9O4		-1
3c2hmp[c]	3-Carboxy-2-hydroxy-4-methylpentanoate	C7H10O5		-2
2ippm[c]	2-Isopropylmaleate	C7H8O4		-2
23dhmp[m]	(R)-2,3-Dihydroxy-3-methylpentanoate	C6H11O4		-1
mcrocoa[c]	3-Methylcrotonyl-CoA	C26H42N7O17P3S	[]	
mglutcoa[c]	3-Methylglutaconyl-CoA	C27H42N7O19P3S	[]	
hibcoa[c]	(S)-3-Hydroxyisobutyryl-CoA	C25H39N7O18P3S		-3
macrylcoa[c]	Methacrylyl-CoA	C25H40N7O17P3S	[]	
2mb2coa[c]	trans-2-Methylbut-2-enoyl-CoA	C26H38N7O17P3S		-4
3hmbcoa[c]	(S)-3-Hydroxy-2-methylbutyryl-CoA	C26H40N7O18P3S		-4
2maacoa[c]	2-Methyl-3-acetoacetyl-CoA	C26H38N7O18P3S		-4
2mbcoa[c]	2-Methylbutanoyl-CoA	C26H40N7O17P3S		-4
aacoa[m]	Acetoacetyl-CoA	C25H36N7O18P3S		-4
leu-L[m]	L-Leucine	C6H13NO2		0
4mop[m]	4-Methyl-2-oxopentanoate	C6H9O3		-1
4mop[c]	4-Methyl-2-oxopentanoate	C6H9O3		-1
pydam[c]	Pyridoxamine	C8H13N2O2		1
pyam5p[c]	Pyridoxamine 5-phosphate	C8H12N2O5P		-1
pydx[c]	Pyridoxal	C8H9NO3		0
pydx5p[c]	Pyridoxal 5-phosphate	C8H8NO6P		-2

pydxn[c]	Pyridoxine	C8H11NO3	0
pdx5p[c]	Pyridoxine 5-phosphate	C8H10NO6P	-2
4abut[m]	4-Aminobutanoate	C4H9NO2	0
succsal[m]	Succinic-semialdehyde	C4H5O3	0
