# Supplementary material for: Elucidating redox balance shift in Scheffersomyces stipitis’ fermentative metabolism using a modified genome-scale metabolic model
Source: Microb Cell Fact. 2018 Sep 5;17:140. doi: 10.1186/s12934-018-0983-y (PMC6126012; doi:10.1186/s12934-018-0983-y)
Supplement: Supplementary file 5 — Additional file 5: Table S5. GEM reactions. [file 12934_2018_983_MOESM5_ESM.pdf]

Table S5: GEM reactions

Rxn name	Gene-reaction	Genes	Proteins	Subsystem	Reversible	LB	UB	Objective
SUCOAS1m	(PICST_69303 and PICST_66961)	PICST_69303 PICST_66961	(LSC1 and LSC2)	Citrate cycle (TCA cycle)	1	-1000	0	0
SUCOASm	(PICST_69303 and PICST_66961)	PICST_69303 PICST_66961	(LSC1 and LSC2)	Citrate cycle (TCA cycle)	1	-1000	0	0
ALCDH1	(PICST_29079 or PICST_45137)	PICST_29079 PICST_45137	(ADH4 or ADH7)	Glycolysis/Gluconeogenesis	1	-1000	0	0
CHOT				Acyltransferases	1	0	1000	0
ATP1	(PICST_30157 or PICST_30672 or PICST_31786 or PICST_32083 or PICST_33561 or PICST_33873 or PICST_39246 or PICST_49179 or PICST_54293 or PICST_57110 or PICST_67560 or PICST_83848 or PICST_85147 or PICST_86369 or PICST_88824 or PICST_30460 or PICST_31021 or PICST_75361 or PICST_75213)	PICST_30157 PICST_30672 PICST_31786 PICST_32083 PICST_33561 PICST_33873 PICST_39246 PICST_49179 PICST_54293 PICST_57110 PICST_67560 PICST_83848 PICST_85147 PICST_86369 PICST_88824 PICST_30460 PICST_31021 PICST_75361 PICST_75213	(ISW2 or MDL2 or PEX6 or VCP1 or DRS2 or MSP1 or ISW1.2 or RAD16 or HSP78 or FUN30 or DNF3 or PEX1 or DNF1 or SAP11 or MOT1 or SMC5 or RHC18 or MYO2 or YND1)	Purine Metabolism	0	2.6	2.6	0
ARGSS	PICST_34492	PICST_34492	ARG1	Arginine and Proline Metabolism	1	-1000	1000	0
GLUDC	(PICST_40180 or PICST_55334 or PICST_87778)	PICST_40180 PICST_55334 PICST_87778	(DCE1 or GAD2 or DPL1)	Butanoate Metabolism	0	0	1000	0
SUCCSALDH	(PICST_40468 or PICST_57266)	PICST_40468 PICST_57266	(UGA2 or UGA22)	Butanoate Metabolism	0	0	1000	0

4ABUTAT	(PICST_46781 or PICST_54153)	PICST_46781 PICST_54153	(UGA1.2 or UGA1.1)	Butanoate Metabolism	1	-1000	1000	0
ASPTAm	(PICST_51039 or PICST_80440)	PICST_51039 PICST_80440	(AAT22 or AAT1)	Arginine and Proline Metabolism	1	-1000	1000	0
DALAOX	PICST_57912	PICST_57912	DAO1	Alanine, aspartate and glutamate metabolism	0	0	1000	0
ARGSL	PICST_62615	PICST_62615	ARG4	Arginine and Proline Metabolism	1	-1000	1000	0
ASPTA	PICST_66059	PICST_66059	AAT2	Alanine, aspartate and glutamate metabolism	1	-1000	1000	0
ALATA_L	PICST_70108	PICST_70108	ALA2	Alanine, aspartate and glutamate metabolism	1	-1000	1000	0
CBPS	(PICST_90563 or (PICST_50410 and PICST_28533))	PICST_90563 PICST_50410 PICST_28533	(URA2 or (CPA2 and CPA1))	Pyrimidine Metabolism	0	0	1000	0
ASNN	PICST_39982	PICST_39982	HYP	Nitrogen Metabolism	0	0	1000	0
GLNS	PICST_68474	PICST_68474	GLN1	Nitrogen Metabolism	0	0	1000	0
GLUD1	PICST_78004	PICST_78004	GDH2	Nitrogen Metabolism	1	0	0	0
ASNS1	PICST_83540	PICST_83540	ASN1	Nitrogen Metabolism	0	0	1000	0
GLUS1	PICST_87428	PICST_87428	GLT1	Nitrogen Metabolism	0	0	1000	0
GLUPRT	PICST_78788	PICST_78788	ADE4	Purine Metabolism	0	0	1000	0
ACGAMK	PICST_32526	PICST_32526	NAG5	Alternate Carbon metabolism	0	0	1000	0
CHTNS	(PICST_29105 or PICST_29807 or PICST_42812 or PICST_51281 or PICST_65597)	PICST_29105 PICST_29807 PICST_42812 PICST_51281 PICST_65597	(CHS3 or CHS8 ot CHS1 or CHS2.2 or CHS2)	Amino Sugar Metabolism	0	0	1000	0
CHIT	(PICST_31390 or PICST_48142 or PICST_68871 or PICST_91537 or PICST_77156 or PICST_32069)	PICST_31390 PICST_48142 PICST_68871 PICST_91537 PICST_77156 PICST_32069	(CHT4 or CHT2 or CHT3 or CHT1 or BHA1 or HEX1)	Amino Sugar Metabolism	0	0	1000	0
CHTNDA	PICST_43807	PICST_43807	CDA2	Amino Sugar Metabolism	0	0	1000	0

CYB5R	(PICST_44816 or PICST_45522 or PICST_68997)	PICST_44816 PICST_45522 PICST_68997	(CBR2 or CBR3 or CBR1)	Amino Sugar Metabolism	1	-1000	1000	0
ACGALAC	PICST_48829	PICST_48829	NAG2	Amino Sugar Metabolism	0	0	1000	0
ACGAMPP	PICST_61206	PICST_61206	UAP1	Amino Sugar Metabolism	1	-1000	1000	0
G6PDA	PICST_62706	PICST_62706	NAG1	Amino Sugar Metabolism	0	0	1000	0
GF6PTA	PICST_67692	PICST_67692	GFA1	Amino Sugar Metabolism	0	0	1000	0
ACGAM6PS	PICST_77083	PICST_77083	GNA1	Amino Sugar Metabolism	1	-1000	1000	0
ACGAMPM	PICST_81149	PICST_81149	PCM1	Amino Sugar Metabolism	1	-1000	1000	0
ACONT3m	PICST_43263	PICST_43263	HYP	Anaplerotic reactions	1	-1000	1000	0
MCITL2m	PICST_62080	PICST_62080	ICL1	Anaplerotic reactions	0	0	1000	0
ALPHNH	PICST_28452	PICST_28452	DUR1	Arginine and Proline Metabolism	0	0	1000	0
UREASE	PICST_28452	PICST_28452	DUR1	Arginine and Proline Metabolism	1	-1000	1000	0
GLU5K	PICST_29301	PICST_29301	PRO2	Arginine and Proline Metabolism	0	0	1000	0
AGMT	(PICST_32515 or PICST_55948)	PICST_32515 PICST_55948	(SPB2 or CAR12)	Arginine and Proline Metabolism	0	0	1000	0
P5CR	PICST_35750	PICST_35750	PRO3	Arginine and Proline Metabolism	0	0	1000	0
OCBT	PICST_35868	PICST_35868	ARG3	Arginine and Proline Metabolism	0	0	1000	0
AMID1	(PICST_43525 or PICST_47832 or PICST_48268 or PICST_87495)	PICST_43525 PICST_47832 PICST_48268 PICST_87495	(AMI1 or AMD2 or AMD4 or GTA2)	Arginine and Proline Metabolism	0	0	1000	0
AMID2	(PICST_43525 or PICST_47832 or PICST_48268 or PICST_87495)	PICST_43525 PICST_47832 PICST_48268 PICST_87495	(AMI1 or AMD2 or AMD4 or GTA2)	Arginine and Proline Metabolism	0	0	1000	0
ACGS	(PICST_51037 or PICST_76181)	PICST_51037 PICST_76181	(ARG2 or NAT3)	Arginine and Proline Metabolism	0	0	1000	0

EHGLATm	(PICST_51039 or PICST_80440)	PICST_51039 PICST_80440	(AAT22 or AAT1)	Arginine and Proline Metabolism	0	0	1000	0
ACOTAm	PICST_52948	PICST_52948	ARG8	Arginine and Proline Metabolism	0	0	1000	0
ACGKm	PICST_55623	PICST_55623	HYP	Arginine and Proline Metabolism	0	0	1000	0
AGPRm	PICST_55623	PICST_55623	HYP	Arginine and Proline Metabolism	0	0	1000	0
ACGSm	PICST_60752	PICST_60752	ECM40	Arginine and Proline Metabolism	0	0	1000	0
ORNTACim	PICST_60752	PICST_60752	ECM40	Arginine and Proline Metabolism	0	0	1000	0
EHGLAT	PICST_66059	PICST_66059	AAT2	Arginine and Proline Metabolism	0	0	1000	0
ORNTA	PICST_77538	PICST_77538	CAR2	Arginine and Proline Metabolism	0	0	1000	0
ARGN	PICST_78364	PICST_78364	CAR1	Arginine and Proline Metabolism	0	0	1000	0
PRO1m	PICST_84147	PICST_84147	PUT1	Arginine and Proline Metabolism	0	0	1000	0
ORNDC	PICST_90612	PICST_90612	SPE1	Arginine and Proline Metabolism	0	0	1000	0
G5SD1	PICST_90997	PICST_90997	HYP	Arginine and Proline Metabolism	0	0	1000	0
G5SD2	PICST_90997	PICST_90997	HYP	Arginine and Proline Metabolism	0	0	1000	0
G5SAD				Arginine and Proline Metabolism	1	-1000	1000	0
G5SADm				Arginine and Proline Metabolism	1	-1000	1000	0
ALDD22	(PICST_27980 or PICST_68558)	PICST_27980 PICST_68558	(ADH1 or ADH2)	Pantothenate and CoA Biosynthesis	0	0	1000	0
ASP1DC				Pantothenate and CoA Biosynthesis	0	0	1000	0

BTS	PICST_30955	PICST_30955	BIO2	Biotin Metabolism	1	-1000	1000	0
AMAOT	(PICST_38281 or PICST_38592)	PICST_38281 PICST_38592	(YOD2 or BIO3)	Biotin Metabolism	1	-1000	1000	0
DBTS	PICST_85593	PICST_85593	BIO4	Biotin Metabolism	1	-1000	1000	0
BTDD-RR	(PICST_74116 or PICST_89701)	PICST_74116 PICST_89701	(SOR5 or SOR1)	Butanoate Metabolism	1	-1000	1000	0
SUCCDHpm	(PICST_66251 and PICST_50416 and PICST_35527 and PICST_90476)	PICST_66251 PICST_50416 PICST_35527 PICST_90476	(SDH1 and SDH2 and SDH6 and SDH4)	Citrate cycle (TCA cycle)	1	0	1000	0
MDHm	(PICST_40132 or PICST_66451 or PICST_78343)	PICST_40132 PICST_66451 PICST_78343	(MDHM or MDH1 or MDH2)	Citrate cycle (TCA cycle)	1	0	1000	0
ICDHm	(PICST_42313 or PICST_91057)	PICST_42313 PICST_91057	(IDH2 or IDH1)	Citrate cycle (TCA cycle)	0	0	1000	0
ICDH1m	(PICST_43870 or PICST_72104)	PICST_43870 PICST_72104	(IDP2 or IDP1)	Citrate cycle (TCA cycle)	0	0	1000	0
OXGDHm	(PICST_79721 and PICST_68297 and PICST_69303)	PICST_79721 PICST_68297 PICST_69303	(KGD1 and KGD2 and LPD1)	Citrate cycle (TCA cycle)	0	0	1000	0
ACONHm	(PICST_85181 or PICST_90724)	PICST_85181 PICST_90724	(ACO1 or ACO2)	Citrate cycle (TCA cycle)	1	-1000	1000	0
CITSm	PICST_85554	PICST_85554	CIT1	Citrate cycle (TCA cycle)	0	0	1000	0
FUMm	(PICST_85745 or PICST_74587)	PICST_85745 PICST_74587	(FUM1 or FUM2)	Citrate cycle (TCA cycle)	0	0	1000	0
HCYSMT	PICST_30270	PICST_30270	SAM4	Cysteine and Methionine Metabolism	0	0	1000	0
SHSL1	(PICST_36608 or PICST_59680)	PICST_36608 PICST_59680	(HYP or HYP)	Cysteine and Methionine Metabolism	0	0	1000	0
SHSL4	(PICST_36608 or PICST_59680)	PICST_36608 PICST_59680	(HYP or HYP)	Cysteine and Methionine Metabolism	1	-1000	1000	0
DKMPPD2	PICST_37619	PICST_37619	HYP	Cysteine and Methionine Metabolism	0	0	1000	0

SALACLYASE	PICST_40180	PICST_40180	DCE1	Cysteine and Methionine Metabolism	0	0	1000	0
MDRPD	PICST_44113	PICST_44113	HYP	Cysteine and Methionine Metabolism	0	0	1000	0
HSD1	PICST_57619	PICST_57619	HOM6	Glycine, serine and threonine metabolism	0	0	1000	0
METAT	PICST_65158	PICST_65158	SAM2	Cysteine and Methionine Metabolism	0	0	1000	0
MTAP	PICST_65182	PICST_65182	HYP	Cysteine and Methionine Metabolism	0	0	1000	0
UNK3	(PICST_66767 or PICST_34099)	PICST_66767 PICST_34099	(ARO8 or ARO9.1)	Cysteine and Methionine Metabolism	0	0	1000	0
ASPK	PICST_69242	PICST_69242	HOM3	Glycine, serine and threonine metabolism	0	0	1000	0
AHC	PICST_76463	PICST_76463	SAH1	Cysteine and Methionine Metabolism	0	0	1000	0
METS	PICST_78051	PICST_78051	MET6	Cysteine and Methionine Metabolism	0	0	1000	0
MHPGLUT	PICST_78051	PICST_78051	MET6	Cysteine and Methionine Metabolism	0	0	1000	0
SPMS	(PICST_84352 or PICST_40259)	PICST_84352 PICST_40259	(HYP or HYP)	Arginine and Proline Metabolism	0	0	1000	0
SPMS1	(PICST_84352 or PICST_40259)	PICST_84352 PICST_40259	(HYP or HYP)	Arginine and Proline Metabolism	0	0	1000	0
ADMDC	PICST_85160	PICST_85160	SPE2	Arginine and Proline Metabolism	0	0	1000	0
ASAD	PICST_89195	PICST_89195	HOM2	Glycine, serine and threonine metabolism	0	0	1000	0
MTRI	PICST_89976	PICST_89976	MTN2	Cysteine and Methionine Metabolism	1	-1000	1000	0
SERAT				Cysteine and Methionine Metabolism	0	0	1000	0
CYSS	(PICST_30734 or PICST_59891)	PICST_30734 PICST_59891	(CYK2 or CYK1)	Sulphur Metabolism	0	0	1000	0

HSERTA	(PICST_30795 or PICST_76901)	PICST_30795 PICST_76901	(MET23 or MET2)	Sulphur Metabolism	1	-1000	1000	0
AHSERL	PICST_31162	PICST_31162	HYP	Sulphur Metabolism	0	0	1000	0
CYSTL	PICST_54505	PICST_54505	STR3	Nitrogen Metabolism	0	0	1000	0
CYSTGL	PICST_77247	PICST_77247	CYS3	Nitrogen Metabolism	0	0	1000	0
DPEPDASE1	(PICST_28659 or PICST_34371 or PICST_28404 or PICST_51091 or PICST_89481 or PICST_78234)	PICST_28659 PICST_34371 PICST_28404 PICST_51091 PICST_89481 PICST_78234	(HYP or CPS1 or PRD1.2 or PRD1.1 or OCT1 or CPGL)	Dipeptide metabolism	0	0	1000	0
DPEPDASE10	(PICST_28659 or PICST_34371 or PICST_28404 or PICST_51091 or PICST_89481 or PICST_78234)	PICST_28659 PICST_34371 PICST_28404 PICST_51091 PICST_89481 PICST_78234	(HYP or CPS1 or PRD1.2 or PRD1.1 or OCT1 or CPGL)	Dipeptide metabolism	0	0	1000	0
DPEPDASE11	(PICST_28659 or PICST_34371 or PICST_28404 or PICST_51091 or PICST_89481 or PICST_78234)	PICST_28659 PICST_34371 PICST_28404 PICST_51091 PICST_89481 PICST_78234	(HYP or CPS1 or PRD1.2 or PRD1.1 or OCT1 or CPGL)	Dipeptide metabolism	0	0	1000	0
DPEPDASE12	(PICST_28659 or PICST_34371 or PICST_28404 or PICST_51091 or PICST_89481 or PICST_78234)	PICST_28659 PICST_34371 PICST_28404 PICST_51091 PICST_89481 PICST_78234	(HYP or CPS1 or PRD1.2 or PRD1.1 or OCT1 or CPGL)	Dipeptide metabolism	0	0	1000	0

DPEPDASE13	(PICST_28659 or PICST_34371 or PICST_28404 or PICST_51091 or PICST_89481 or PICST_78234)	PICST_28659 PICST_34371 PICST_28404 PICST_51091 PICST_89481 PICST_78234	(HYP or CPS1 or PRD1.2 or PRD1.1 or OCT1 or CPGL)	Dipeptide metabolism	0	0	1000	0
DPEPDASE14	(PICST_28659 or PICST_34371 or PICST_28404 or PICST_51091 or PICST_89481 or PICST_78234)	PICST_28659 PICST_34371 PICST_28404 PICST_51091 PICST_89481 PICST_78234	(HYP or CPS1 or PRD1.2 or PRD1.1 or OCT1 or CPGL)	Dipeptide metabolism	0	0	1000	0
DPEPDASE16	(PICST_28659 or PICST_34371 or PICST_28404 or PICST_51091 or PICST_89481 or PICST_78234)	PICST_28659 PICST_34371 PICST_28404 PICST_51091 PICST_89481 PICST_78234	(HYP or CPS1 or PRD1.2 or PRD1.1 or OCT1 or CPGL)	Dipeptide metabolism	0	0	1000	0
DPEPDASE2	(PICST_28659 or PICST_34371 or PICST_28404 or PICST_51091 or PICST_89481 or PICST_78234)	PICST_28659 PICST_34371 PICST_28404 PICST_51091 PICST_89481 PICST_78234	(HYP or CPS1 or PRD1.2 or PRD1.1 or OCT1 or CPGL)	Dipeptide metabolism	0	0	1000	0
DPEPDASE3	(PICST_28659 or PICST_34371 or PICST_28404 or PICST_51091 or PICST_89481 or PICST_78234)	PICST_28659 PICST_34371 PICST_28404 PICST_51091 PICST_89481 PICST_78234	(HYP or CPS1 or PRD1.2 or PRD1.1 or OCT1 or CPGL)	Dipeptide metabolism	0	0	1000	0

DPEPDASE4	(PICST_28659 or PICST_34371 or PICST_28404 or PICST_51091 or PICST_89481 or PICST_78234)	PICST_28659 PICST_34371 PICST_28404 PICST_51091 PICST_89481 PICST_78234	(HYP or CPS1 or PRD1.2 or PRD1.1 or OCT1 or CPGL)	Dipeptide metabolism	0	0	1000	0
DPEPDASE5	(PICST_28659 or PICST_34371 or PICST_28404 or PICST_51091 or PICST_89481 or PICST_78234)	PICST_28659 PICST_34371 PICST_28404 PICST_51091 PICST_89481 PICST_78234	(HYP or CPS1 or PRD1.2 or PRD1.1 or OCT1 or CPGL)	Dipeptide metabolism	0	0	1000	0
DPEPDASE6	(PICST_28659 or PICST_34371 or PICST_28404 or PICST_51091 or PICST_89481 or PICST_78234)	PICST_28659 PICST_34371 PICST_28404 PICST_51091 PICST_89481 PICST_78234	(HYP or CPS1 or PRD1.2 or PRD1.1 or OCT1 or CPGL)	Dipeptide metabolism	0	0	1000	0
DPEPDASE7	(PICST_28659 or PICST_34371 or PICST_28404 or PICST_51091 or PICST_89481 or PICST_78234)	PICST_28659 PICST_34371 PICST_28404 PICST_51091 PICST_89481 PICST_78234	(HYP or CPS1 or PRD1.2 or PRD1.1 or OCT1 or CPGL)	Dipeptide metabolism	0	0	1000	0
DPEPDASE8	(PICST_28659 or PICST_34371 or PICST_28404 or PICST_51091 or PICST_89481 or PICST_78234)	PICST_28659 PICST_34371 PICST_28404 PICST_51091 PICST_89481 PICST_78234	(HYP or CPS1 or PRD1.2 or PRD1.1 or OCT1 or CPGL)	Dipeptide metabolism	0	0	1000	0

DPEPDASE9	(PICST_28659 or PICST_34371 or PICST_28404 or PICST_51091 or PICST_89481 or PICST_78234)	PICST_28659 PICST_34371 PICST_28404 PICST_51091 PICST_89481 PICST_78234	(HYP or CPS1 or PRD1.2 or PRD1.1 or OCT1 or CPGL)	Dipeptide metabolism	0	0	1000	0
EX_13BDglcn(e)				Exchange Reaction	1	0	1000	0
EX_2mbald(e)				Exchange Reaction	1	0	1000	0
EX_2mbtoh(e)				Exchange Reaction	1	0	1000	0
EX_2mppal(e)				Exchange Reaction	1	0	1000	0
EX_2obut(e)				Exchange Reaction	0	0	1000	0
EX_2pg(e)				Exchange Reaction	1	0	1000	0
EX_2pglyc(e)				Exchange Reaction	1	0	1000	0
EX_2phetoh(e)				Exchange Reaction	1	0	1000	0
EX_3c3hmp(e)				Exchange Reaction	1	0	1000	0
EX_3mbald(e)				Exchange Reaction	1	0	1000	0
EX_3mop(e)				Exchange Reaction	1	0	1000	0
EX_3pg(e)				Exchange Reaction	1	0	1000	0
EX_4abut(e)				Exchange Reaction	1	0	1000	0
EX_4abz(e)				Exchange Reaction	1	0	1000	0
EX_4hpro-LT(e)				Exchange Reaction	0	0	1000	0
EX_5aop(e)				Exchange Reaction	1	0	1000	0
EX_6pgc(e)				Exchange Reaction	1	0	1000	0
EX_8aonn(e)				Exchange Reaction	1	0	1000	0
EX_ac(e)				Exchange Reaction	1	0	1000	0
EX_acac(e)				Exchange Reaction	0	0	1000	0
EX_acald(e)				Exchange Reaction	1	0	1000	0
EX_acetm(e)				Exchange Reaction	0	0	1000	0
EX_ade(e)				Exchange Reaction	1	0	1000	0
EX_adn(e)				Exchange Reaction	1	0	1000	0
EX_agm(e)				Exchange Reaction	0	0	1000	0
EX_akg(e)				Exchange Reaction	1	0	1000	0
EX_alaasp(e)				Exchange Reaction	0	0	1000	0
EX_ala-D(e)				Exchange Reaction	0	0	1000	0

EX_alagln(e)	Exchange Reaction	0	0	1000	0
EX_alaglu(e)	Exchange Reaction	0	0	1000	0
EX_alagly(e)	Exchange Reaction	0	0	1000	0
EX_alahis(e)	Exchange Reaction	0	0	1000	0
EX_ala-L(e)	Exchange Reaction	1	0	1000	0
EX_alaleu(e)	Exchange Reaction	0	0	1000	0
EX_alan(e)	Exchange Reaction	0	0	1000	0
EX_alathr(e)	Exchange Reaction	0	0	1000	0
EX_amet(e)	Exchange Reaction	1	0	1000	0
EX_amp(e)	Exchange Reaction	0	0	1000	0
EX_arab-D(e)	Exchange Reaction	0	0	1000	0
EX_arab-L(e)	Exchange Reaction	0	0	1000	0
EX_arg-L(e)	Exchange Reaction	1	0	1000	0
EX_asn-L(e)	Exchange Reaction	1	0	1000	0
EX_asp-L(e)	Exchange Reaction	1	0	1000	0
EX_btd-RR(e)	Exchange Reaction	1	0	1000	0
EX_btn(e)	Exchange Reaction	1	0	1000	0
EX_cellb(e)	Exchange Reaction	1	0	1000	0
EX_cgly(e)	Exchange Reaction	1	0	1000	0
EX_ch4s(e)	Exchange Reaction	1	0	1000	0
EX_chol(e)	Exchange Reaction	1	0	1000	0
EX_cit(e)	Exchange Reaction	1	0	1000	0
EX_citr(e)	Exchange Reaction	0	0	1000	0
EX_cmp(e)	Exchange Reaction	1	0	1000	0
EX_co2(e)	Exchange Reaction	1	0	1000	0
EX_csn(e)	Exchange Reaction	1	0	1000	0
EX_cys-L(e)	Exchange Reaction	1	0	1000	0
EX_cyst(e)	Exchange Reaction	1	0	1000	0
EX_cytd(e)	Exchange Reaction	1	0	1000	0
EX_dad-2(e)	Exchange Reaction	1	0	1000	0
EX_dann(e)	Exchange Reaction	1	0	1000	0
EX_dca(e)	Exchange Reaction	1	0	1000	0
EX_dcyt(e)	Exchange Reaction	1	0	1000	0
EX_ddca(e)	Exchange Reaction	1	0	1000	0

EX_dgsn(e)	Exchange Reaction	1	0	1000	0
EX_dha(e)	Exchange Reaction	0	0	1000	0
EX_din(e)	Exchange Reaction	1	0	1000	0
EX_docosa(e)	Exchange Reaction	1	0	1000	0
EX_dtmp(e)	Exchange Reaction	1	0	1000	0
EX_dttp(e)	Exchange Reaction	1	0	1000	0
EX_duri(e)	Exchange Reaction	1	0	1000	0
EX_eicosapen(e)	Exchange Reaction	1	0	1000	0
EX_epist(e)	Exchange Reaction	1	0	1000	0
EX_ergst(e)	Exchange Reaction	1	0	1000	0
EX_etha(e)	Exchange Reaction	1	0	1000	0
EX_etoh(e)	Exchange Reaction	1	0	1000	0
EX_f6p(e)	Exchange Reaction	0	0	1000	0
EX_fe2(e)	Exchange Reaction	1	-1000	1000	0
EX_fecost(e)	Exchange Reaction	1	0	1000	0
EX_fmn(e)	Exchange Reaction	1	0	1000	0
EX_for(e)	Exchange Reaction	1	0	1000	0
EX_frmd(e)	Exchange Reaction	0	0	1000	0
EX_fru(e)	Exchange Reaction	1	0	1000	0
EX_fum(e)	Exchange Reaction	1	0	1000	0
EX_g1p(e)	Exchange Reaction	0	0	1000	0
EX_g3pc(e)	Exchange Reaction	1	0	1000	0
EX_g3pi(e)	Exchange Reaction	1	0	1000	0
EX_g6p(e)	Exchange Reaction	0	0	1000	0
EX_gal(e)	Exchange Reaction	1	0	1000	0
EX_gam6p(e)	Exchange Reaction	1	0	1000	0
EX_gcald(e)	Exchange Reaction	1	0	1000	0
EX_glc-D(e)	Exchange Reaction	1	0	0	0
EX_glcn-D(e)	Exchange Reaction	0	0	1000	0
EX_gln-L(e)	Exchange Reaction	1	0	1000	0
EX_gluala(e)	Exchange Reaction	1	0	1000	0
EX_glu-L(e)	Exchange Reaction	1	0	1000	0
EX_glx(e)	Exchange Reaction	1	0	1000	0
EX_gly(e)	Exchange Reaction	1	0	1000	0

EX_glyasn(e)	Exchange Reaction	0	0	1000	0
EX_glyasp(e)	Exchange Reaction	0	0	1000	0
EX_glyc(e)	Exchange Reaction	1	0	1000	0
EX_glyc3p(e)	Exchange Reaction	1	0	1000	0
EX_glyclt(e)	Exchange Reaction	1	0	1000	0
EX_glygln(e)	Exchange Reaction	0	0	1000	0
EX_glyglu(e)	Exchange Reaction	0	0	1000	0
EX_glymet(e)	Exchange Reaction	0	0	1000	0
EX_glypro(e)	Exchange Reaction	0	0	1000	0
EX_gmp(e)	Exchange Reaction	1	0	1000	0
EX_gsn(e)	Exchange Reaction	1	0	1000	0
EX_gthox(e)	Exchange Reaction	1	0	1000	0
EX_gthrd(e)	Exchange Reaction	1	0	1000	0
EX_gua(e)	Exchange Reaction	1	0	1000	0
EX_h(e)	Exchange Reaction	1	-1000	1000	0
EX_h2o(e)	Exchange Reaction	1	-1000	1000	0
EX_hdca(e)	Exchange Reaction	1	0	1000	0
EX_hdcea(e)	Exchange Reaction	1	0	1000	0
EX_hepdcea(e)	Exchange Reaction	1	0	1000	0
EX_hexa(e)	Exchange Reaction	1	0	1000	0
EX_hexc(e)	Exchange Reaction	1	0	1000	0
EX_his-L(e)	Exchange Reaction	1	0	1000	0
EX_hom-L(e)	Exchange Reaction	0	0	1000	0
EX_iamoh(e)	Exchange Reaction	1	0	1000	0
EX_ibutoh(e)	Exchange Reaction	1	0	1000	0
EX_ile-L(e)	Exchange Reaction	1	0	1000	0
EX_ind3eth(e)	Exchange Reaction	0	0	1000	0
EX_inost(e)	Exchange Reaction	1	0	1000	0
EX_ins(e)	Exchange Reaction	1	0	1000	0
EX_k(e)	Exchange Reaction	1	-1000	1000	0
EX_lac-D(e)	Exchange Reaction	1	0	1000	0
EX_lac-L(e)	Exchange Reaction	1	0	1000	0
EX_lanost(e)	Exchange Reaction	1	0	1000	0
EX_lcyst(e)	Exchange Reaction	1	0	1000	0

EX_leu-L(e)	Exchange Reaction	1	0	1000	0
EX_lys-L(e)	Exchange Reaction	1	0	1000	0
EX_madg(e)	Exchange Reaction	0	0	1000	0
EX_mal-L(e)	Exchange Reaction	1	0	1000	0
EX_malt(e)	Exchange Reaction	1	0	1000	0
EX_maltr(e)	Exchange Reaction	0	0	1000	0
EX_man(e)	Exchange Reaction	1	0	1000	0
EX_man1p(e)	Exchange Reaction	1	0	1000	0
EX_man6p(e)	Exchange Reaction	1	0	1000	0
EX_mann-D(e)	Exchange Reaction	0	0	1000	0
EX_mbdg(e)	Exchange Reaction	0	0	1000	0
EX_meoh(e)	Exchange Reaction	0	0	1000	0
EX_metala(e)	Exchange Reaction	0	0	1000	0
EX_met-L(e)	Exchange Reaction	1	0	1000	0
EX_Na(e)	Exchange Reaction	1	-1000	1000	0
EX_nac(e)	Exchange Reaction	1	0	1000	0
EX_NACGAM(e)	Exchange Reaction	0	0	1000	0
EX_nadp(e)	Exchange Reaction	1	0	1000	0
EX_nh4(e)	Exchange Reaction	1	-1000	1000	0
EX_nmn(e)	Exchange Reaction	1	0	1000	0
EX_o2(e)	Exchange Reaction	1	-1	-1	0
EX_oaa(e)	Exchange Reaction	1	0	1000	0
EX_ocdca(e)	Exchange Reaction	1	0	1000	0
EX_ocdcea(e)	Exchange Reaction	1	0	1000	0
EX_ocdcta(e)	Exchange Reaction	1	0	1000	0
EX_ocdcya(e)	Exchange Reaction	1	0	1000	0
EX_opro(e)	Exchange Reaction	0	0	1000	0
EX_orn(e)	Exchange Reaction	1	0	1000	0
EX_pacald(e)	Exchange Reaction	1	0	1000	0
EX_pap(e)	Exchange Reaction	1	0	1000	0
EX_pc(e)	Exchange Reaction	1	0	1000	0
EX_pep(e)	Exchange Reaction	1	0	1000	0
EX_phe-L(e)	Exchange Reaction	1	0	1000	0
EX_pi(e)	Exchange Reaction	1	-1000	1000	0

EX_pnto-R(e)	Exchange Reaction	1	0	1000	0
EX_ppi(e)	Exchange Reaction	0	0	1000	0
EX_pro-L(e)	Exchange Reaction	1	0	1000	0
EX_pser-L(e)	Exchange Reaction	1	0	1000	0
EX_ptd1ino(e)	Exchange Reaction	1	0	1000	0
EX_ptrc(e)	Exchange Reaction	1	0	1000	0
EX_pyr(e)	Exchange Reaction	1	0	1000	0
EX_rbt(e)	Exchange Reaction	0	0	1000	0
EX_rham-L(e)	Exchange Reaction	0	0	1000	0
EX_rib-D(e)	Exchange Reaction	1	0	1000	0
EX_ribflv(e)	Exchange Reaction	1	0	1000	0
EX_sbt-D(e)	Exchange Reaction	1	0	1000	0
EX_sbt-L(e)	Exchange Reaction	1	0	1000	0
EX_ser-L(e)	Exchange Reaction	1	0	1000	0
EX_so3(e)	Exchange Reaction	1	0	1000	0
EX_so4(e)	Exchange Reaction	1	-1000	1000	0
EX_spmd(e)	Exchange Reaction	1	0	1000	0
EX_sprm(e)	Exchange Reaction	1	0	1000	0
EX_srb-L(e)	Exchange Reaction	1	0	1000	0
EX_succ(e)	Exchange Reaction	1	0	1000	0
EX_sucr(e)	Exchange Reaction	1	0	1000	0
EX_taur(e)	Exchange Reaction	1	0	1000	0
EX_thm(e)	Exchange Reaction	1	0	1000	0
EX_thmmp(e)	Exchange Reaction	1	0	1000	0
EX_thmpp(e)	Exchange Reaction	1	0	1000	0
EX_thr-L(e)	Exchange Reaction	1	0	1000	0
EX_thym(e)	Exchange Reaction	1	0	1000	0
EX_thymd(e)	Exchange Reaction	1	0	1000	0
EX_tmp(e)	Exchange Reaction	0	0	1000	0
EX_tre(e)	Exchange Reaction	1	0	1000	0
EX_trp-L(e)	Exchange Reaction	1	0	1000	0
EX_ttdca(e)	Exchange Reaction	1	0	1000	0
EX_tyr-L(e)	Exchange Reaction	1	0	1000	0
EX_ump(e)	Exchange Reaction	1	0	1000	0

EX_ura(e)				Exchange Reaction	1	0	1000	0
EX_urate(e)				Exchange Reaction	0	0	1000	0
EX_urea(e)				Exchange Reaction	1	0	1000	0
EX_uri(e)				Exchange Reaction	1	0	1000	0
EX_val-L(e)				Exchange Reaction	1	0	1000	0
EX_xan(e)				Exchange Reaction	1	0	1000	0
EX_xtsn(e)				Exchange Reaction	1	0	1000	0
EX_xyl-D(e)				Exchange Reaction	1	-5	0	0
EX_xylt(e)				Exchange Reaction	1	0	1000	0
EX_zymst(e)				Exchange Reaction	1	0	1000	0
Hexacoa_ex				Exchange Reaction	0	0	1000	0
FACOAL100	(PICST_35069 or PICST_55002 or PICST_65071 or PICST_89450)	PICST_35069 PICST_55002 PICST_65071 PICST_89450	(FAA23 or FAA24 or FAA4 or FAA22)	Fatty acid Biosynthesis	1	-1000	1000	0
FACOAL140	(PICST_35069 or PICST_55002 or PICST_65071 or PICST_89450)	PICST_35069 PICST_55002 PICST_65071 PICST_89450	(FAA23 or FAA24 or FAA4 or FAA22)	Fatty acid Biosynthesis	1	-1000	1000	0
FACOAL160	(PICST_35069 or PICST_55002 or PICST_65071 or PICST_89450)	PICST_35069 PICST_55002 PICST_65071 PICST_89450	(FAA23 or FAA24 or FAA4 or FAA22)	Fatty acid Biosynthesis	1	-1000	1000	0
FACOAL161	(PICST_35069 or PICST_55002 or PICST_65071 or PICST_89450)	PICST_35069 PICST_55002 PICST_65071 PICST_89450	(FAA23 or FAA24 or FAA4 or FAA22)	Fatty acid Biosynthesis	1	-1000	1000	0
FACOAL170	(PICST_35069 or PICST_55002 or PICST_65071 or PICST_89450)	PICST_35069 PICST_55002 PICST_65071 PICST_89450	(FAA23 or FAA24 or FAA4 or FAA22)	Fatty acid Biosynthesis	1	-1000	1000	0

FACOAL171	(PICST_35069 or PICST_55002 or PICST_65071 or PICST_89450)	PICST_35069 PICST_55002 PICST_65071 PICST_89450	(FAA23 or FAA24 or FAA4 or FAA22)	Fatty acid Biosynthesis	1	-1000	1000	0
FACOAL180	(PICST_35069 or PICST_55002 or PICST_65071 or PICST_89450)	PICST_35069 PICST_55002 PICST_65071 PICST_89450	(FAA23 or FAA24 or FAA4 or FAA22)	Fatty acid Biosynthesis	1	-1000	1000	0
FACOAL181	(PICST_35069 or PICST_55002 or PICST_65071 or PICST_89450)	PICST_35069 PICST_55002 PICST_65071 PICST_89450	(FAA23 or FAA24 or FAA4 or FAA22)	Fatty acid Biosynthesis	1	-1000	1000	0
FACOAL182	(PICST_35069 or PICST_55002 or PICST_65071 or PICST_89450)	PICST_35069 PICST_55002 PICST_65071 PICST_89450	(FAA23 or FAA24 or FAA4 or FAA22)	Fatty acid Biosynthesis	1	-1000	1000	0
FACOAL183	(PICST_35069 or PICST_55002 or PICST_65071 or PICST_89450)	PICST_35069 PICST_55002 PICST_65071 PICST_89450	(FAA23 or FAA24 or FAA4 or FAA22)	Fatty acid Biosynthesis	1	-1000	1000	0
FACOAL205	(PICST_35069 or PICST_55002 or PICST_65071 or PICST_89450)	PICST_35069 PICST_55002 PICST_65071 PICST_89450	(FAA23 or FAA24 or FAA4 or FAA22)	Fatty acid Biosynthesis	1	-1000	1000	0
FACOAL60	(PICST_35069 or PICST_55002 or PICST_65071 or PICST_89450)	PICST_35069 PICST_55002 PICST_65071 PICST_89450	(FAA23 or FAA24 or FAA4 or FAA22)	Fatty acid Biosynthesis	1	-1000	1000	0
FAS100ACPm	PICST_64208	PICST_64208	CEM1	Fatty acid Biosynthesis	0	0	1000	0
FAS120ACPm	PICST_64208	PICST_64208	CEM1	Fatty acid Biosynthesis	0	0	1000	0
FAS140ACPm	PICST_64208	PICST_64208	CEM1	Fatty acid Biosynthesis	0	0	1000	0
FAS160ACPm	PICST_64208	PICST_64208	CEM1	Fatty acid Biosynthesis	0	0	1000	0
FAS161ACPm	PICST_64208	PICST_64208	CEM1	Fatty acid Biosynthesis	0	0	1000	0
FAS170ACPm_L	PICST_64208	PICST_64208	CEM1	Fatty acid Biosynthesis	0	0	1000	0

FAS171ACPm	PICST_64208	PICST_64208	CEM1	Fatty acid Biosynthesis	0	0	1000	0
FAS171ACPm_L	PICST_64208	PICST_64208	CEM1	Fatty acid Biosynthesis	0	0	1000	0
FAS180ACPm	PICST_64208	PICST_64208	CEM1	Fatty acid Biosynthesis	0	0	1000	0
FAS181ACPm	PICST_64208	PICST_64208	CEM1	Fatty acid Biosynthesis	0	0	1000	0
FAS182ACPm	PICST_64208	PICST_64208	CEM1	Fatty acid Biosynthesis	0	0	1000	0
FAS183ACPm	PICST_64208	PICST_64208	CEM1	Fatty acid Biosynthesis	0	0	1000	0
FAS200ACPm	PICST_64208	PICST_64208	CEM1	Fatty acid Biosynthesis	0	0	1000	0
FAS205ACPm	PICST_64208	PICST_64208	CEM1	Fatty acid Biosynthesis	0	0	1000	0
FAS220ACPm	PICST_64208	PICST_64208	CEM1	Fatty acid Biosynthesis	0	0	1000	0
FAS240ACPm	PICST_64208	PICST_64208	CEM1	Fatty acid Biosynthesis	0	0	1000	0
FAS260ACPm	PICST_64208	PICST_64208	CEM1	Fatty acid Biosynthesis	0	0	1000	0
FAS60ACPm_L	PICST_64208	PICST_64208	CEM1	Fatty acid Biosynthesis	0	0	1000	0
FAS80ACPm_L	PICST_64208	PICST_64208	CEM1	Fatty acid Biosynthesis	0	0	1000	0
MCOATAm	PICST_65214	PICST_65214	MCT1	Fatty acid Biosynthesis	1	-1000	1000	0
DESAT16	PICST_70693	PICST_70693	OLE1	Fatty acid Biosynthesis	0	0	1000	0
DESAT17	PICST_70693	PICST_70693	OLE1	Fatty acid Biosynthesis	0	0	1000	0
DESAT18	PICST_70693	PICST_70693	OLE1	Fatty acid Biosynthesis	0	0	1000	0
ACOATA	(PICST_85125 and PICST_80966)	PICST_85125 PICST_80966	(FAS1 and FAS2)	Fatty acid Biosynthesis	1	-1000	1000	0
ACOATAm	(PICST_85125 and PICST_80966)	PICST_85125 PICST_80966	(FAS1 and FAS2)	Fatty acid Biosynthesis	1	-1000	1000	0
FA100ACPH	(PICST_85125 and PICST_80966)	PICST_85125 PICST_80966	(FAS1 and FAS2)	Fatty acid Biosynthesis	1	-1000	1000	0
FA120ACPH	(PICST_85125 and PICST_80966)	PICST_85125 PICST_80966	(FAS1 and FAS2)	Fatty acid Biosynthesis	1	-1000	1000	0
FA140ACPH	(PICST_85125 and PICST_80966)	PICST_85125 PICST_80966	(FAS1 and FAS2)	Fatty acid Biosynthesis	1	-1000	1000	0
FA160ACPH	(PICST_85125 and PICST_80966)	PICST_85125 PICST_80966	(FAS1 and FAS2)	Fatty acid Biosynthesis	1	-1000	1000	0
FA161ACPH	(PICST_85125 and PICST_80966)	PICST_85125 PICST_80966	(FAS1 and FAS2)	Fatty acid Biosynthesis	1	-1000	1000	0
FA170ACPH	(PICST_85125 and PICST_80966)	PICST_85125 PICST_80966	(FAS1 and FAS2)	Fatty acid Biosynthesis	1	-1000	1000	0

FA171ACPH	(PICST_85125 and PICST_80966)	PICST_85125 PICST_80966	(FAS1 and FAS2)	Fatty acid Biosynthesis	1	-1000	1000	0
FA180ACPH	(PICST_85125 and PICST_80966)	PICST_85125 PICST_80966	(FAS1 and FAS2)	Fatty acid Biosynthesis	1	-1000	1000	0
FA181ACPH	(PICST_85125 and PICST_80966)	PICST_85125 PICST_80966	(FAS1 and FAS2)	Fatty acid Biosynthesis	1	-1000	1000	0
FA182ACPH	(PICST_85125 and PICST_80966)	PICST_85125 PICST_80966	(FAS1 and FAS2)	Fatty acid Biosynthesis	1	-1000	1000	0
FA183ACPH	(PICST_85125 and PICST_80966)	PICST_85125 PICST_80966	(FAS1 and FAS2)	Fatty acid Biosynthesis	1	-1000	1000	0
FA200ACPH	(PICST_85125 and PICST_80966)	PICST_85125 PICST_80966	(FAS1 and FAS2)	Fatty acid Biosynthesis	1	-1000	1000	0
FA205ACPH	(PICST_85125 and PICST_80966)	PICST_85125 PICST_80966	(FAS1 and FAS2)	Fatty acid Biosynthesis	1	-1000	1000	0
FA220ACPH	(PICST_85125 and PICST_80966)	PICST_85125 PICST_80966	(FAS1 and FAS2)	Fatty acid Biosynthesis	1	-1000	1000	0
FA240ACPH	(PICST_85125 and PICST_80966)	PICST_85125 PICST_80966	(FAS1 and FAS2)	Fatty acid Biosynthesis	1	-1000	1000	0
FA260ACPH	(PICST_85125 and PICST_80966)	PICST_85125 PICST_80966	(FAS1 and FAS2)	Fatty acid Biosynthesis	1	-1000	1000	0
FA60ACPH	(PICST_85125 and PICST_80966)	PICST_85125 PICST_80966	(FAS1 and FAS2)	Fatty acid Biosynthesis	1	-1000	1000	0
FA80ACPH	(PICST_85125 and PICST_80966)	PICST_85125 PICST_80966	(FAS1 and FAS2)	Fatty acid Biosynthesis	1	-1000	1000	0
FAS100	(PICST_85125 and PICST_80966)	PICST_85125 PICST_80966	(FAS1 and FAS2)	Fatty acid Biosynthesis	0	0	1000	0
FAS100COA	(PICST_85125 and PICST_80966)	PICST_85125 PICST_80966	(FAS1 and FAS2)	Fatty acid Biosynthesis	0	0	1000	0
FAS120	(PICST_85125 and PICST_80966)	PICST_85125 PICST_80966	(FAS1 and FAS2)	Fatty acid Biosynthesis	0	0	1000	0
FAS120COA	(PICST_85125 and PICST_80966)	PICST_85125 PICST_80966	(FAS1 and FAS2)	Fatty acid Biosynthesis	0	0	1000	0
FAS140	(PICST_85125 and PICST_80966)	PICST_85125 PICST_80966	(FAS1 and FAS2)	Fatty acid Biosynthesis	0	0	1000	0

FAS140COA	(PICST_85125 and PICST_80966)	PICST_85125 PICST_80966	(FAS1 and FAS2)	Fatty acid Biosynthesis	0	0	1000	0
FAS160	(PICST_85125 and PICST_80966)	PICST_85125 PICST_80966	(FAS1 and FAS2)	Fatty acid Biosynthesis	0	0	1000	0
FAS160COA	(PICST_85125 and PICST_80966)	PICST_85125 PICST_80966	(FAS1 and FAS2)	Fatty acid Biosynthesis	0	0	1000	0
FAS161	(PICST_85125 and PICST_80966)	PICST_85125 PICST_80966	(FAS1 and FAS2)	Fatty acid Biosynthesis	0	0	1000	0
FAS170_L	(PICST_85125 and PICST_80966)	PICST_85125 PICST_80966	(FAS1 and FAS2)	Fatty acid Biosynthesis	0	0	1000	0
FAS170COA_L	(PICST_85125 and PICST_80966)	PICST_85125 PICST_80966	(FAS1 and FAS2)	Fatty acid Biosynthesis	0	0	1000	0
FAS171_L	(PICST_85125 and PICST_80966)	PICST_85125 PICST_80966	(FAS1 and FAS2)	Fatty acid Biosynthesis	0	0	1000	0
FAS171COA_L	(PICST_85125 and PICST_80966)	PICST_85125 PICST_80966	(FAS1 and FAS2)	Fatty acid Biosynthesis	0	0	1000	0
FAS180	(PICST_85125 and PICST_80966)	PICST_85125 PICST_80966	(FAS1 and FAS2)	Fatty acid Biosynthesis	0	0	1000	0
FAS180COA	(PICST_85125 and PICST_80966)	PICST_85125 PICST_80966	(FAS1 and FAS2)	Fatty acid Biosynthesis	0	0	1000	0
FAS181	(PICST_85125 and PICST_80966)	PICST_85125 PICST_80966	(FAS1 and FAS2)	Fatty acid Biosynthesis	0	0	1000	0
FAS182	(PICST_85125 and PICST_80966)	PICST_85125 PICST_80966	(FAS1 and FAS2)	Fatty acid Biosynthesis	0	0	1000	0
FAS183	(PICST_85125 and PICST_80966)	PICST_85125 PICST_80966	(FAS1 and FAS2)	Fatty acid Biosynthesis	0	0	1000	0
FAS200	(PICST_85125 and PICST_80966)	PICST_85125 PICST_80966	(FAS1 and FAS2)	Fatty acid Biosynthesis	0	0	1000	0
FAS200coA	(PICST_85125 and PICST_80966)	PICST_85125 PICST_80966	(FAS1 and FAS2)	Fatty acid Biosynthesis	0	0	1000	0
FAS205	(PICST_85125 and PICST_80966)	PICST_85125 PICST_80966	(FAS1 and FAS2)	Fatty acid Biosynthesis	0	0	1000	0
FAS220	(PICST_85125 and PICST_80966)	PICST_85125 PICST_80966	(FAS1 and FAS2)	Fatty acid Biosynthesis	0	0	1000	0

FAS220coA	(PICST_85125 and PICST_80966)	PICST_85125 PICST_80966	(FAS1 and FAS2)	Fatty acid Biosynthesis	0	0	1000	0
FAS240	(PICST_85125 and PICST_80966)	PICST_85125 PICST_80966	(FAS1 and FAS2)	Fatty acid Biosynthesis	0	0	1000	0
FAS240coA	(PICST_85125 and PICST_80966)	PICST_85125 PICST_80966	(FAS1 and FAS2)	Fatty acid Biosynthesis	0	0	1000	0
FAS260	(PICST_85125 and PICST_80966)	PICST_85125 PICST_80966	(FAS1 and FAS2)	Fatty acid Biosynthesis	0	0	1000	0
FAS260coA	(PICST_85125 and PICST_80966)	PICST_85125 PICST_80966	(FAS1 and FAS2)	Fatty acid Biosynthesis	0	0	1000	0
FAS60_L	(PICST_85125 and PICST_80966)	PICST_85125 PICST_80966	(FAS1 and FAS2)	Fatty acid Biosynthesis	0	0	1000	0
FAS60COA_L	(PICST_85125 and PICST_80966)	PICST_85125 PICST_80966	(FAS1 and FAS2)	Fatty acid Biosynthesis	0	0	1000	0
FAS80_L	(PICST_85125 and PICST_80966)	PICST_85125 PICST_80966	(FAS1 and FAS2)	Fatty acid Biosynthesis	0	0	1000	0
FAS80COA_L	(PICST_85125 and PICST_80966)	PICST_85125 PICST_80966	(FAS1 and FAS2)	Fatty acid Biosynthesis	0	0	1000	0
PCOATA	(PICST_85125 and PICST_80966)	PICST_85125 PICST_80966	(FAS1 and FAS2)	Fatty acid Biosynthesis	1	-1000	1000	0
PCOATAm	(PICST_85125 and PICST_80966)	PICST_85125 PICST_80966	(FAS1 and FAS2)	Fatty acid Biosynthesis	1	-1000	1000	0
DESAT18_3				Fatty acid Biosynthesis	0	0	1000	0
DESAT20_5				Fatty acid Biosynthesis	0	0	1000	0
FAO100_1	(PICST_54590 and PICST_55180 and PICST_79854 and PICST_30193 and PICST_32846 and PICST_33103)	PICST_54590 PICST_55180 PICST_79854 PICST_30193 PICST_32846 PICST_33103	(FOXp and POT13 and POT11 and ECH1 and EHO3 and ACD99)	Fatty Acid Metabolism	0	0	1000	0

FAO120_1	(PICST_54590 and PICST_55180 and PICST_79854 and PICST_30193 and PICST_32846 and PICST_33103)	PICST_54590 PICST_55180 PICST_79854 PICST_30193 PICST_32846 PICST_33103	(FOXp and POT13 and POT11 and ECH1 and EHO3 and ACD99)	Fatty Acid Metabolism	0	0	1000	0
FAO140_1	(PICST_54590 and PICST_55180 and PICST_79854 and PICST_30193 and PICST_32846 and PICST_33103)	PICST_54590 PICST_55180 PICST_79854 PICST_30193 PICST_32846 PICST_33103	(FOXp and POT13 and POT11 and ECH1 and EHO3 and ACD99)	Fatty Acid Metabolism	0	0	1000	0
FAO160_1	(PICST_54590 and PICST_55180 and PICST_79854 and PICST_30193 and PICST_32846 and PICST_33103)	PICST_54590 PICST_55180 PICST_79854 PICST_30193 PICST_32846 PICST_33103	(FOXp and POT13 and POT11 and ECH1 and EHO3 and ACD99)	Fatty Acid Metabolism	0	0	1000	0
FAO170_1	(PICST_54590 and PICST_55180 and PICST_79854 and PICST_30193 and PICST_32846 and PICST_33103)	PICST_54590 PICST_55180 PICST_79854 PICST_30193 PICST_32846 PICST_33103	(FOXp and POT13 and POT11 and ECH1 and EHO3 and ACD99)	Fatty Acid Metabolism	0	0	1000	0
FAO180_1	(PICST_54590 and PICST_55180 and PICST_79854 and PICST_30193 and PICST_32846 and PICST_33103)	PICST_54590 PICST_55180 PICST_79854 PICST_30193 PICST_32846 PICST_33103	(FOXp and POT13 and POT11 and ECH1 and EHO3 and ACD99)	Fatty Acid Metabolism	0	0	1000	0

FAO200_1	(PICST_54590 and PICST_55180 and PICST_79854 and PICST_30193 and PICST_32846 and PICST_33103)	PICST_54590 PICST_55180 PICST_79854 PICST_30193 PICST_32846 PICST_33103	(FOXp and POT13 and POT11 and ECH1 and EHO3 and ACD99)	Fatty Acid Metabolism	0	0	1000	0
FAO220_1	(PICST_54590 and PICST_55180 and PICST_79854 and PICST_30193 and PICST_32846 and PICST_33103)	PICST_54590 PICST_55180 PICST_79854 PICST_30193 PICST_32846 PICST_33103	(FOXp and POT13 and POT11 and ECH1 and EHO3 and ACD99)	Fatty Acid Metabolism	0	0	1000	0
FAO240_L2	(PICST_54590 and PICST_55180 and PICST_79854 and PICST_30193 and PICST_32846 and PICST_33103)	PICST_54590 PICST_55180 PICST_79854 PICST_30193 PICST_32846 PICST_33103	(FOXp and POT13 and POT11 and ECH1 and EHO3 and ACD99)	Fatty Acid Metabolism	0	0	1000	0
FAO260_L2	(PICST_54590 and PICST_55180 and PICST_79854 and PICST_30193 and PICST_32846 and PICST_33103)	PICST_54590 PICST_55180 PICST_79854 PICST_30193 PICST_32846 PICST_33103	(FOXp and POT13 and POT11 and ECH1 and EHO3 and ACD99)	Fatty Acid Metabolism	0	0	1000	0
FAO60_L2	(PICST_54590 and PICST_55180 and PICST_79854 and PICST_30193 and PICST_32846 and PICST_33103)	PICST_54590 PICST_55180 PICST_79854 PICST_30193 PICST_32846 PICST_33103	(FOXp and POT13 and POT11 and ECH1 and EHO3 and ACD99)	Fatty Acid Metabolism	0	0	1000	0

FAO80_1	(PICST_54590 and PICST_55180 and PICST_79854 and PICST_30193 and PICST_32846 and PICST_33103)	PICST_54590 PICST_55180 PICST_79854 PICST_30193 PICST_32846 PICST_33103	(FOXp and POT13 and POT11 and ECH1 and EHO3 and ACD99)	Fatty Acid Metabolism	0	0	1000	0
FAO100	(PICST_54590 and PICST_55180 and PICST_79854 and PICST_30193 and PICST_32846 and PICST_75424)	PICST_54590 PICST_55180 PICST_79854 PICST_30193 PICST_32846 PICST_75424	(FOXp and POT13 and POT11 and ECH1 and EHO3 and POX1)	Fatty Acid Metabolism	0	0	1000	0
FAO120	(PICST_54590 and PICST_55180 and PICST_79854 and PICST_30193 and PICST_32846 and PICST_75424)	PICST_54590 PICST_55180 PICST_79854 PICST_30193 PICST_32846 PICST_75424	(FOXp and POT13 and POT11 and ECH1 and EHO3 and POX1)	Fatty Acid Metabolism	0	0	1000	0
FAO140	(PICST_54590 and PICST_55180 and PICST_79854 and PICST_30193 and PICST_32846 and PICST_75424)	PICST_54590 PICST_55180 PICST_79854 PICST_30193 PICST_32846 PICST_75424	(FOXp and POT13 and POT11 and ECH1 and EHO3 and POX1)	Fatty Acid Metabolism	0	0	1000	0
FAO160	(PICST_54590 and PICST_55180 and PICST_79854 and PICST_30193 and PICST_32846 and PICST_75424)	PICST_54590 PICST_55180 PICST_79854 PICST_30193 PICST_32846 PICST_75424	(FOXp and POT13 and POT11 and ECH1 and EHO3 and POX1)	Fatty Acid Metabolism	0	0	1000	0

FAO161p_even	(PICST_54590 and PICST_55180 and PICST_79854 and PICST_30193 and PICST_32846 and PICST_75424)	PICST_54590 PICST_55180 PICST_79854 PICST_30193 PICST_32846 PICST_75424	(FOXp and POT13 and POT11 and ECH1 and EHO3 and POX1)	Fatty Acid Metabolism	0	0	1000	0
FAO161p_odd	(PICST_54590 and PICST_55180 and PICST_79854 and PICST_30193 and PICST_32846 and PICST_75424)	PICST_54590 PICST_55180 PICST_79854 PICST_30193 PICST_32846 PICST_75424	(FOXp and POT13 and POT11 and ECH1 and EHO3 and POX1)	Fatty Acid Metabolism	0	0	1000	0
FAO170	(PICST_54590 and PICST_55180 and PICST_79854 and PICST_30193 and PICST_32846 and PICST_75424)	PICST_54590 PICST_55180 PICST_79854 PICST_30193 PICST_32846 PICST_75424	(FOXp and POT13 and POT11 and ECH1 and EHO3 and POX1)	Fatty Acid Metabolism	0	0	1000	0
FAO171p_even	(PICST_54590 and PICST_55180 and PICST_79854 and PICST_30193 and PICST_32846 and PICST_75424)	PICST_54590 PICST_55180 PICST_79854 PICST_30193 PICST_32846 PICST_75424	(FOXp and POT13 and POT11 and ECH1 and EHO3 and POX1)	Fatty Acid Metabolism	0	0	1000	0
FAO171p_odd	(PICST_54590 and PICST_55180 and PICST_79854 and PICST_30193 and PICST_32846 and PICST_75424)	PICST_54590 PICST_55180 PICST_79854 PICST_30193 PICST_32846 PICST_75424	(FOXp and POT13 and POT11 and ECH1 and EHO3 and POX1)	Fatty Acid Metabolism	0	0	1000	0

FAO180	(PICST_54590 and PICST_55180 and PICST_79854 and PICST_30193 and PICST_32846 and PICST_75424)	PICST_54590 PICST_55180 PICST_79854 PICST_30193 PICST_32846 PICST_75424	(FOXp and POT13 and POT11 and ECH1 and EHO3 and POX1)	Fatty Acid Metabolism	0	0	1000	0
FAO181p_even	(PICST_54590 and PICST_55180 and PICST_79854 and PICST_30193 and PICST_32846 and PICST_75424)	PICST_54590 PICST_55180 PICST_79854 PICST_30193 PICST_32846 PICST_75424	(FOXp and POT13 and POT11 and ECH1 and EHO3 and POX1)	Fatty Acid Metabolism	0	0	1000	0
FAO181p_odd	(PICST_54590 and PICST_55180 and PICST_79854 and PICST_30193 and PICST_32846 and PICST_75424)	PICST_54590 PICST_55180 PICST_79854 PICST_30193 PICST_32846 PICST_75424	(FOXp and POT13 and POT11 and ECH1 and EHO3 and POX1)	Fatty Acid Metabolism	0	0	1000	0
FAO182p_even/even	(PICST_54590 and PICST_55180 and PICST_79854 and PICST_30193 and PICST_32846 and PICST_75424)	PICST_54590 PICST_55180 PICST_79854 PICST_30193 PICST_32846 PICST_75424	(FOXp and POT13 and POT11 and ECH1 and EHO3 and POX1)	Fatty Acid Metabolism	0	0	1000	0
FAO182p_even/odd	(PICST_54590 and PICST_55180 and PICST_79854 and PICST_30193 and PICST_32846 and PICST_75424)	PICST_54590 PICST_55180 PICST_79854 PICST_30193 PICST_32846 PICST_75424	(FOXp and POT13 and POT11 and ECH1 and EHO3 and POX1)	Fatty Acid Metabolism	0	0	1000	0

FAO182p_odd/odd	(PICST_54590 and PICST_55180 and PICST_79854 and PICST_30193 and PICST_32846 and PICST_75424)	PICST_54590 PICST_55180 PICST_79854 PICST_30193 PICST_32846 PICST_75424	(FOXp and POT13 and POT11 and ECH1 and EHO3 and POX1)	Fatty Acid Metabolism	0	0	1000	0
FAO183p	(PICST_54590 and PICST_55180 and PICST_79854 and PICST_30193 and PICST_32846 and PICST_75424)	PICST_54590 PICST_55180 PICST_79854 PICST_30193 PICST_32846 PICST_75424	(FOXp and POT13 and POT11 and ECH1 and EHO3 and POX1)	Fatty Acid Metabolism	0	0	1000	0
FAO200	(PICST_54590 and PICST_55180 and PICST_79854 and PICST_30193 and PICST_32846 and PICST_75424)	PICST_54590 PICST_55180 PICST_79854 PICST_30193 PICST_32846 PICST_75424	(FOXp and POT13 and POT11 and ECH1 and EHO3 and POX1)	Fatty Acid Metabolism	0	0	1000	0
FAO205p	(PICST_54590 and PICST_55180 and PICST_79854 and PICST_30193 and PICST_32846 and PICST_75424)	PICST_54590 PICST_55180 PICST_79854 PICST_30193 PICST_32846 PICST_75424	(FOXp and POT13 and POT11 and ECH1 and EHO3 and POX1)	Fatty Acid Metabolism	0	0	1000	0
FAO220	(PICST_54590 and PICST_55180 and PICST_79854 and PICST_30193 and PICST_32846 and PICST_75424)	PICST_54590 PICST_55180 PICST_79854 PICST_30193 PICST_32846 PICST_75424	(FOXp and POT13 and POT11 and ECH1 and EHO3 and POX1)	Fatty Acid Metabolism	0	0	1000	0

FAO240_L1	(PICST_54590 and PICST_55180 and PICST_79854 and PICST_30193 and PICST_32846 and PICST_75424)	PICST_54590 PICST_55180 PICST_79854 PICST_30193 PICST_32846 PICST_75424	(FOXp and POT13 and POT11 and ECH1 and EHO3 and POX1)	Fatty Acid Metabolism	0	0	1000	0
FAO260_L1	(PICST_54590 and PICST_55180 and PICST_79854 and PICST_30193 and PICST_32846 and PICST_75424)	PICST_54590 PICST_55180 PICST_79854 PICST_30193 PICST_32846 PICST_75424	(FOXp and POT13 and POT11 and ECH1 and EHO3 and POX1)	Fatty Acid Metabolism	0	0	1000	0
FAO60_L1	(PICST_54590 and PICST_55180 and PICST_79854 and PICST_30193 and PICST_32846 and PICST_75424)	PICST_54590 PICST_55180 PICST_79854 PICST_30193 PICST_32846 PICST_75424	(FOXp and POT13 and POT11 and ECH1 and EHO3 and POX1)	Fatty Acid Metabolism	0	0	1000	0
FAO80	(PICST_54590 and PICST_55180 and PICST_79854 and PICST_30193 and PICST_32846 and PICST_75424)	PICST_54590 PICST_55180 PICST_79854 PICST_30193 PICST_32846 PICST_75424	(FOXp and POT13 and POT11 and ECH1 and EHO3 and POX1)	Fatty Acid Metabolism	0	0	1000	0
GTPCI	PICST_34960	PICST_34960	GHC1	Folate Metabolism	0	0	1000	0
FTHFCL	PICST_36098	PICST_36098	FAU1	Folate Metabolism	0	0	1000	0
DHFRi	(PICST_38512 or PICST_60284 or PICST_82622)	PICST_38512 PICST_60284 PICST_82622	(FSH1 or FSH3 or DFR1)	Folate Metabolism	0	0	1000	0
ADCL	PICST_42028	PICST_42028	HYP	Folate Metabolism	0	0	1000	0
AKP1	(PICST_43722 or PICST_82654)	PICST_43722 PICST_82654	(PHO82 or PHO8)	Folate Metabolism	0	0	1000	0
THFGLUS	(PICST_47181 or PICST_71737)	PICST_47181 PICST_71737	(FOL3 or HYP)	Folate Metabolism	1	-1000	1000	0

DHNPA	PICST_66087	PICST_66087	HYP	Folate Metabolism	0	0	1000	0
DHPS	PICST_66087	PICST_66087	HYP	Folate Metabolism	0	0	1000	0
FOLD3	PICST_66087	PICST_66087	HYP	Folate Metabolism	0	0	1000	0
HPPK	PICST_66087	PICST_66087	HYP	Folate Metabolism	0	0	1000	0
MTHFR2	(PICST_71674 or PICST_83803)	PICST_71674 PICST_83803	(HYP or HYP)	Folate Metabolism	0	0	1000	0
MTHFD2	PICST_73792	PICST_73792	HYP	Folate Metabolism	0	0	1000	0
ADCS	PICST_74393	PICST_74393	HYP	Folate Metabolism	0	0	1000	0
FTHFLm	PICST_80013	PICST_80013	MIS1	Folate Metabolism	0	0	1000	0
MTHFCm	PICST_80013	PICST_80013	MIS1	Folate Metabolism	1	-1000	1000	0
MTHFD1m	PICST_80013	PICST_80013	MIS1	Folate Metabolism	1	-1000	1000	0
MTHFD	(PICST_80013 or PICST_88119)	PICST_80013 PICST_88119	(MIS1 or ADE3)	Glyoxalate and Dicarboxylate Metabolism	1	-1000	1000	0
THFATm	PICST_83560	PICST_83560	GCV1	Folate Metabolism	0	0	1000	0
FTHFL	PICST_88119	PICST_88119	ADE3	Folate Metabolism	0	0	1000	0
MTHFC	PICST_88119	PICST_88119	ADE3	Folate Metabolism	1	-1000	1000	0
ASPOcm				Folate Metabolism	0	0	1000	0
DHFS				Folate Metabolism	0	0	1000	0
FTHFCLm				Folate Metabolism	0	0	1000	0
FUCPA	PICST_32146	PICST_32146	ALD1	Fructose and Mannose Metabolism	1	-1000	1000	0
HEX4	(PICST_32526 or PICST_73701 or PICST_85453)	PICST_32526 PICST_73701 PICST_85453	(NAG5 or GLK1 or HXK1)	Glycolysis/Gluconeogenesis	0	0	1000	0
HEX7	(PICST_32526 or PICST_73701 or PICST_85453)	PICST_32526 PICST_73701 PICST_85453	(NAG5 or GLK1 or HXK1)	Glycolysis/Gluconeogenesis	0	0	1000	0
HEX8	(PICST_32526 or PICST_73701 or PICST_85453)	PICST_32526 PICST_73701 PICST_85453	(NAG5 or GLK1 or HXK1)	Glycolysis/Gluconeogenesis	0	0	1000	0
SBTD_D	PICST_40172	PICST_40172	SOR3	Fructose and Mannose Metabolism	0	0	1000	0
SBTD_L	PICST_40172	PICST_40172	SOR3	Fructose and Mannose Metabolism	0	0	1000	0

FBP26	PICST_68318	PICST_68318	FBP26	Glycolysis/Gluconeogenesis	0	0	1000	0
FBP262	PICST_68318	PICST_68318	FBP26	Glycolysis/Gluconeogenesis	0	0	1000	0
BPFK	(PICST_68318 or PICST_57978 or PICST_66347 or PICST_85029)	PICST_68318 PICST_57978 PICST_66347 PICST_85029	(FBP26 or FRK26.2 or PFK26 or FRK26.1)	Fructose and Mannose Metabolism	1	0	1000	0
MAN6PI	PICST_72090	PICST_72090	PMI1	Fructose and Mannose Metabolism	1	-1000	1000	0
MAN1PGT	PICST_74665	PICST_74665	MPG1	Fructose and Mannose Metabolism	0	0	1000	0
PMANM	PICST_77262	PICST_77262	PMM1	Fructose and Mannose Metabolism	1	-1000	1000	0
KDRA				Fructose and Mannose Metabolism	0	0	1000	0
LALDH				Fructose and Mannose Metabolism	1	-1000	1000	0
MANNDH				Fructose and Mannose Metabolism	0	0	1000	0
RHAMDEH				Fructose and Mannose Metabolism	0	0	1000	0
RHAMDH				Fructose and Mannose Metabolism	0	0	1000	0
RHAMLAC				Fructose and Mannose Metabolism	0	0	1000	0
SBTR	PICST_89614	PICST_89614	XYL1	Alternate Carbon metabolism	0	0	1000	0
LACZ	PICST_30036	PICST_30036	LAC4	Galactose Metabolism	0	0	1000	0
UGLT	PICST_57493	PICST_57493	GAL7	Galactose Metabolism	1	-1000	1000	0
GALK	PICST_82625	PICST_82625	GAL1	Galactose Metabolism	0	0	1000	0
GALDH	PICST_58212	PICST_58212	YMT1	Alternate Carbon metabolism	1	-1000	1000	0
MALTASE	PICST_62666	PICST_62666	AGL1	Alternate Carbon metabolism	0	0	1000	0
UDPG4E	PICST_76750	PICST_76750	GAL10	Glycolysis/Gluconeogenesis	1	-1000	1000	0
GTHP	(PICST_52875 or PICST_78456)	PICST_52875 PICST_78456	(HYR1 or GPX2)	Glutathione Metabolism	1	-1000	1000	0

DPEPDASE15	(PICST_28659 or PICST_34371 or PICST_28404 or PICST_51091 or PICST_89481 or PICST_78234)	PICST_28659 PICST_34371 PICST_28404 PICST_51091 PICST_89481 PICST_78234	(HYP or CPS1 or PRD1.2 or PRD1.1 or OCT1 or CPGL)	Dipeptide metabolism	0	0	1000	0
GTHO	(PICST_40145 or PICST_42900)	PICST_40145 PICST_42900	(GLR2 or GLR1)	Glutathione Metabolism	0	0	1000	0
GTHS	PICST_43206	PICST_43206	GSH2	Glutathione Metabolism	0	0	1000	0
GTMLT	(PICST_58626 or PICST_78698)	PICST_58626 PICST_78698	(EMC37 or EMC38)	Glutathione Metabolism	0	0	1000	0
GLUCYS	PICST_62151	PICST_62151	GSH1	Glutathione Metabolism	0	0	1000	0
OPRONASE	(PICST_81320 or PICST_29736 or PICST_86944)	PICST_81320 PICST_29736 PICST_86944	(HYU1.1 or HYU1.2 or HYU1.3)	Glutathione Metabolism	1	-1000	1000	0
DOLPMMer	PICST_53466	PICST_53466	HYP	Sucrose and Starch Metabolism	0	0	1000	0
DOLK	PICST_58284	PICST_58284	SEC59	Sucrose and Starch Metabolism	0	0	1000	0
DOLPMTcer				Sucrose and Starch Metabolism	0	0	1000	0
TAGL	(PICST_60390 or PICST_78871 or PICST_80311)	PICST_60390 PICST_78871 PICST_80311	(LIP11 or HYP or SEC31)	Glycerolipid Metabolism	0	0	1000	0
DHAK	(PICST_31117 or PICST_53027 or PICST_57462)	PICST_31117 PICST_53027 PICST_57462	(DAK1.1 or DAK1.2 or DAK2)	Glycerolipid Metabolism	0	0	1000	0
G3PT	PICST_42440	PICST_42440	GPP1	Glycerolipid Metabolism	0	0	1000	0
GAT1	(PICST_45343 or PICST_81897)	PICST_45343 PICST_81897	(HYP or SCT1)	Glycerolipid Metabolism	0	0	1000	0
GAT2	(PICST_45343 or PICST_81897)	PICST_45343 PICST_81897	(HYP or SCT1)	Glycerolipid Metabolism	0	0	1000	0
AGAT	(PICST_61839 or PICST_77657)	PICST_61839 PICST_77657	(HYP or HYP)	Glycerolipid Metabolism	0	0	1000	0
GLYK	PICST_66162	PICST_66162	GUT1	Glycerolipid Metabolism	0	0	1000	0
DAGPYP	PICST_67964	PICST_67964	HYP	Glycerolipid Metabolism	0	0	1000	0
PCDAGAT	PICST_72946	PICST_72946	LRO1	Glycerolipid Metabolism	0	0	1000	0

PLD	PICST_81277	PICST_81277	SPO14	Glycerolipid Metabolism	0	0	1000	0
TRIGS				Glycerolipid Metabolism	0	0	1000	0
G3PD1	(PICST_83071 or PICST_90446)	PICST_83071 PICST_90446	(GPD2 or GPD1)	Glycerolipid Metabolism	0	0	1000	0
G3PDm	PICST_68764	PICST_68764	GUT2	Glycerolipid Metabolism	0	0	1000	0
PSERDm	PICST_32693	PICST_32693	PSD1	Phospholipid Metabolism	0	0	1000	0
PSERDc	PICST_40695	PICST_40695	PSD2	Phospholipid Metabolism	0	0	1000	0
PSERSm	PICST_51913	PICST_51913	CHO1	Glycine, serine and threonine metabolism	1	-1000	1000	0
ETHAK	PICST_59919	PICST_59919	EKI1	Phospholipid Metabolism	0	0	1000	0
PETHCT	PICST_76493	PICST_76493	MUQ1	Phospholipid Metabolism	0	0	1000	0
MFAPS				Phospholipid Biosynthesis	0	0	1000	0
CHOLK	PICST_28965	PICST_28965	CKT1	Phospholipid Metabolism	0	0	1000	0
CHLPCTD	PICST_31198	PICST_31198	PCT1	Phospholipid Metabolism	0	0	1000	0
CDPDGPm	PICST_63914	PICST_63914	PGS1	Phospholipid Metabolism	1	-1000	1000	0
PGPPAm				Phospholipid Biosynthesis	0	0	1000	0
PMETM				Phospholipid Biosynthesis	0	0	1000	0
PSERT	PICST_36392	PICST_36392	SER1	Glycine, serine and threonine metabolism	0	0	1000	0
PSPL	PICST_40365	PICST_40365	PSP1	Glycine, serine and threonine metabolism	0	0	1000	0
AGT	PICST_44075	PICST_44075	HYP	Glycine, serine and threonine metabolism	0	0	1000	0
HSD2	PICST_57619	PICST_57619	HOM6	Glycine, serine and threonine metabolism	0	0	1000	0
THRS	PICST_65754	PICST_65754	THR4	Glycine, serine and threonine metabolism	0	0	1000	0
THRA	(PICST_71203 or PICST_74130)	PICST_71203 PICST_74130	(GLY2 or GLY1)	Glycine, serine and threonine metabolism	0	0	1000	0
GCC21m	(PICST_75631 and PICST_83560 and PICST_36951)	PICST_75631 PICST_83560 PICST_36951	(GCV2 and GCV1 and LPD1)	Glycine, serine and threonine metabolism	1	-1000	1000	0

GCC22m	(PICST_75631 and PICST_83560 and PICST_36951)	PICST_75631 PICST_83560 PICST_36951	(GCV2 and GCV1 and LPD1)	Glycine, serine and threonine metabolism	0	0	1000	0
GCC23m	(PICST_75631 and PICST_83560 and PICST_36951)	PICST_75631 PICST_83560 PICST_36951	(GCV2 and GCV1 and LPD1)	Glycine, serine and threonine metabolism	1	-1000	1000	0
CYSTS	PICST_77227	PICST_77227	CYS4	Glycine, serine and threonine metabolism	0	0	1000	0
THRD	PICST_83915	PICST_83915	ILV1	Glycine, serine and threonine metabolism	0	0	1000	0
HSK	PICST_85224	PICST_85224	THR1	Glycine, serine and threonine metabolism	0	0	1000	0
PGCD	PICST_87754	PICST_87754	SER3	Glycine, serine and threonine metabolism	0	0	1000	0
ALASm	PICST_89623	PICST_89623	HEM1	Glycine, serine and threonine metabolism	0	0	1000	0
HEX1	(PICST_32526 or PICST_73701 or PICST_85453)	PICST_32526 PICST_73701 PICST_85453	(NAG5 or GLK1 or HXK1)	Glycolysis/Gluconeogenesis	0	0	1000	0
DPGM	(PICST_34127 or PICST_48292 or PICST_70394)	PICST_34127 PICST_48292 PICST_70394	(GPM1.1 or GPM1.2 or GPM1.3)	Glycolysis/Gluconeogenesis	1	-1000	1000	0
PGM	(PICST_34127 or PICST_48292 or PICST_70394 or PICST_89865)	PICST_34127 PICST_48292 PICST_70394 PICST_89865	(GPM1.1 or GPM1.2 or GPM1.3 or GPM2)	Glycolysis/Gluconeogenesis	1	-1000	1000	0
FBP	PICST_51462	PICST_51462	FBP1	Glycolysis/Gluconeogenesis	0	0	1000	0
PFK	(PICST_66973 and PICST_70563)	PICST_66973 PICST_70563	(PFK1 and PFK2)	Glycolysis/Gluconeogenesis	0	0	1000	0
ENO	PICST_70720	PICST_70720	ENO1	Glycolysis/Gluconeogenesis	1	-1000	1000	0
GAPD	(PICST_76518 or PICST_85794 or PICST_87252)	PICST_76518 PICST_85794 PICST_87252	(TDH1 or TDH2 or TDH3)	Glycolysis/Gluconeogenesis	1	-1000	1000	0
PGK	PICST_78267	PICST_78267	PGK1	Glycolysis/Gluconeogenesis	1	-1000	1000	0

PYK	PICST_83166	PICST_83166	PYK1	Glycolysis/Gluconeogenesis	0	0	1000	0
G6PI	PICST_84923	PICST_84923	PGI1	Glycolysis/Gluconeogenesis	1	-1000	1000	0
FBA	PICST_85951	PICST_85951	FBA1	Glycolysis/Gluconeogenesis	1	-1000	1000	0
TPI	PICST_91105	PICST_91105	TPI1	Glycolysis/Gluconeogenesis	1	-1000	1000	0
FORAMD	PICST_28914	PICST_28914	FRM1	Glyoxalate and Dicarboxylate Metabolism	0	0	1000	0
FDH	(PICST_33765 or PICST_47526 or PICST_91526)	PICST_33765 PICST_47526 PICST_91526	(FDH1.2 or FOC1 or FDH1.1)	Glyoxalate and Dicarboxylate Metabolism	0	0	1000	0
ICL	PICST_62080	PICST_62080	ICL1	Glyoxalate and Dicarboxylate Metabolism	0	0	1000	0
PGLYCP	PICST_84214	PICST_84214	SDT1	Glyoxalate and Dicarboxylate Metabolism	0	0	1000	0
HISTP	PICST_33066	PICST_33066	HIS9	Histidine Metabolism	0	0	1000	0
PRMICI	PICST_34105	PICST_34105	HYP	Histidine Metabolism	0	0	1000	0
IG3PS	PICST_65481	PICST_65481	HIS7	Histidine Metabolism	0	0	1000	0
ATPPRT	PICST_67165	PICST_67165	HIS1	Histidine Metabolism	0	0	1000	0
HISTD	PICST_89228	PICST_89228	HIS2	Histidine Metabolism	0	0	1000	0
PRAMPC	PICST_89228	PICST_89228	HIS2	Histidine Metabolism	0	0	1000	0
PRATPP	PICST_89228	PICST_89228	HIS2	Histidine Metabolism	0	0	1000	0
IGPDH				Histidine Metabolism	0	0	1000	0
HSTPT	PICST_44612	PICST_44612	HIS5	Phenylalanine, Tyrosine and Trytophan Biosynthesis	0	0	1000	0
PINOS	PICST_28132	PICST_28132	PIS1	Inositol Phosphate Metabolism	0	0	1000	0
MI1PS	PICST_29246	PICST_29246	INO1	Inositol Phosphate Metabolism	0	0	1000	0
PIN3K	PICST_42745	PICST_42745	VPS34	Inositol Phosphate Metabolism	0	0	1000	0
PI45BPP	PICST_42966	PICST_42966	PLC1	Inositol Phosphate Metabolism	0	0	1000	0
PIN4K	(PICST_49430 or PICST_79239)	PICST_49430 PICST_79239	(PIK1 or STT4)	Inositol Phosphate Metabolism	0	0	1000	0
I145PH	(PICST_58872 or PICST_82710)	PICST_58872 PICST_82710	(INP51 or INP52)	Inositol Phosphate Metabolism	0	0	1000	0
PI3P4K	PICST_59054	PICST_59054	FAB1	Inositol Phosphate Metabolism	0	0	1000	0
INS2D	(PICST_64256 or PICST_78306)	PICST_64256 PICST_78306		Inositol Phosphate Metabolism	0	0	1000	0

PI4P5K	PICST_64487	PICST_64487	MSS4	Inositol Phosphate Metabolism	0	0	1000	0
HICITDm	PICST_50442	PICST_50442	LYS12	Lysine Biosynthesis	1	-1000	1000	0
DHDPS	PICST_64442	PICST_64442	LRA4	Lysine Biosynthesis	0	0	1000	0
AATA	PICST_66767	PICST_66767	ARO8	Lysine Biosynthesis	1	-1000	1000	0
AASAD1	PICST_68020	PICST_68020	LYS2	Lysine Biosynthesis	0	0	1000	0
AASAD2	PICST_68020	PICST_68020	LYS2	Lysine Biosynthesis	0	0	1000	0
SACCD1	PICST_70210	PICST_70210	LYS9	Lysine Biosynthesis	1	-1000	1000	0
OXOADH	(PICST_79721 and PICST_68297 and PICST_69303)	PICST_79721 PICST_68297 PICST_69303	(KGD1 and KGD2 and LPD1)	Lysine Biosynthesis	1	-1000	1000	0
SACCD2	PICST_86170	PICST_86170	LYS1	Lysine Biosynthesis	1	-1000	1000	0
HACNHm	PICST_88705	PICST_88705	LYS4	Lysine Biosynthesis	1	-1000	1000	0
OXAGm				Lysine Biosynthesis	1	-1000	1000	0
RNMK	PICST_68496	PICST_68496	HYP	NAD Biosynthesis	0	0	1000	0
NADN				NAD Biosynthesis	0	0	1000	0
NADNm				NAD Biosynthesis	0	0	1000	0
NNAMrm				NAD Biosynthesis	1	-1000	1000	0
PNP				NAD Biosynthesis	1	-1000	1000	0
QULNS				Folate Metabolism	0	0	1000	0
NNAM	PICST_33075	PICST_33075	PNC1	Nicotinate and nicotinamide metabolism	1	-1000	1000	0
NNDPR	PICST_36541	PICST_36541	BNA6	Nicotinate and nicotinamide metabolism	0	0	1000	0
NADS1	PICST_40885	PICST_40885	QNS1	Nicotinate and nicotinamide metabolism	0	0	1000	0
NADS2	PICST_40885	PICST_40885	QNS1	Nicotinate and nicotinamide metabolism	0	0	1000	0
NADK	(PICST_53028 or PICST_87580)	PICST_53028 PICST_87580	(POS3 or UTR1)	Nicotinate and nicotinamide metabolism	0	0	1000	0
NNAT	PICST_81173	PICST_81173	HYP	Nicotinate and nicotinamide metabolism	0	0	1000	0
NMNATm	PICST_86133	PICST_86133	NMA1	Nicotinate and nicotinamide metabolism	0	0	1000	0

NAPRT	PICST_87669	PICST_87669	NPT1	Nicotinate and nicotinamide metabolism	0	0	1000	0
NTRLASE2	PICST_36697	PICST_36697	NIT1	Nitrogen Metabolism	0	0	1000	0
NTRLASE3	PICST_36697	PICST_36697	NIT1	Nitrogen Metabolism	0	0	1000	0
RNTR1	PICST_39568	PICST_39568	HYP	Nucleotide Salvage Pathway	0	0	1000	0
RNTR2	PICST_39568	PICST_39568	HYP	Nucleotide Salvage Pathway	0	0	1000	0
RNTR3	PICST_39568	PICST_39568	HYP	Nucleotide Salvage Pathway	0	0	1000	0
RNTR4	PICST_39568	PICST_39568	HYP	Nucleotide Salvage Pathway	0	0	1000	0
ADDAMSE	PICST_59156	PICST_59156	AAH1	Nucleotide Salvage Pathway	0	0	1000	0
PPM2	(PICST_77218 or PICST_91072)	PICST_77218 PICST_91072	(PGM2 or PGM3)	Nucleotide Salvage Pathway	1	-1000	1000	0
CMPN				Nucleotide Salvage Pathway	0	0	1000	0
DRPA				Nucleotide Salvage Pathway	1	-1000	1000	0
GSNK				Nucleotide Salvage Pathway	0	0	1000	0
INSK				Nucleotide Salvage Pathway	0	0	1000	0
TMDK1				Nucleotide Salvage Pathway	0	0	1000	0
TMDPP				Nucleotide Salvage Pathway	1	-1000	1000	0
GHMTm	PICST_78806	PICST_78806	SHM1	Glycine, serine and threonine metabolism	1	-1000	1000	0
GHMT	PICST_67292	PICST_67292	SHM2	Glycine, serine and threonine metabolism	1	-1000	1000	0
NADPHDH	(PICST_31957 or PICST_31958 or PICST_43720 or PICST_52325 or PICST_57042 or PICST_89722 or PICST_55621 or PICST_44614 or PICST_44456 or PICST_31316 or PICST_44613)	PICST_31957 PICST_31958 PICST_43720 PICST_52325 PICST_57042 PICST_89722 PICST_55621 PICST_44614 PICST_44456 PICST_31316 PICST_44613	(OYE2.9 or OYE2.4 or OYE2.2 or OYE3.3 or OPR1 or OYE2.1 or OYE2.3 or OYE2.6 or OYE2.5 or OYE2.8 or OYE2.7)	Oxidative Phosphorylation	0	0	1000	0

CYOR_u6m	(PICST_39828 and PICST_86147 and PICST_81500 and PICST_78316 and PICST_75355 and PICST_73333 and PICST_77955 and PICST_52082)	PICST_39828 PICST_86147 PICST_81500 PICST_78316 PICST_75355 PICST_73333 PICST_77955 PICST_52082	(UCR1 and CYT1 and COR1 and UQC2 and QCR6 and UCR7 and QCR8 and QCR10)	Oxidative Phosphorylation	0	0	1000	0
NADPHR	(PICST_44171 or PICST_53084 or PICST_74654 or PICST_84928)	PICST_44171 PICST_53084 PICST_74654 PICST_84928	(IFR1 or ETR1 or QOR1 or ZTA1)	Oxidative Phosphorylation	0	0	1000	0

NADH2-u6t	(PICST_44890 and PICST_72822 and PICST_75097 and PICST_76226 and PICST_85822 and PICST_68160 and PICST_74163 and PICST_62206 and PICST_58506 and PICST_75518 and PICST_88630 and PICST_31688 and PICST_76559 and PICST_65836 and PICST_46630 and PICST_84570 and PICST_82538 and PICST_76018 and PICST_71972 and PICST_80821 and PICST_69376 and PICST_90638 and PICST_79236)	PICST_44890 PICST_72822 PICST_75097 PICST_76226 PICST_85822 PICST_68160 PICST_74163 PICST_62206 PICST_58506 PICST_75518 PICST_88630 PICST_31688 PICST_76559 PICST_65836 PICST_46630 PICST_84570 PICST_82538 PICST_76018 PICST_71972 PICST_80821 PICST_69376 PICST_90638 PICST_79236	(NUO17 and NUO21.3 and NUO21.1 and HYP and NUO78 and NUC1 and NUO30 and NUO21.2 and NUO13 and NUO20 and NUO1 and NUO51 and NUO24 and NUO10 and NUFM and NBM4 and HYP and NUE1 and ACP1 and ACP2 and NBM8 and HYP and HYP)	Oxidative Phosphorylation	0	0	1000	0
NADPHR1	(PICST_55602 or PICST_77786)	PICST_55602 PICST_77786	(THA13 or HYP)	Oxidative Phosphorylation	0	0	1000	0
PPAm	PICST_55811	PICST_55811	PMA2	Oxidative Phosphorylation	0	0	1000	0
NADHDH	PICST_58800	PICST_58800	NDI1	Oxidative Phosphorylation	0	0	1000	0
NaKATPS1	PICST_60178	PICST_60178	PMR2	Oxidative Phosphorylation	0	0	1000	0
NaKATPS2	PICST_60178	PICST_60178	PMR2	Oxidative Phosphorylation	0	0	1000	0
NaKATPS3	PICST_60178	PICST_60178	PMR2	Oxidative Phosphorylation	0	0	1000	0
SUCCDH1m	(PICST_66251 and PICST_50416 and PICST_35527 and PICST_90476)	PICST_66251 PICST_50416 PICST_35527 PICST_90476	(SDH1 and SDH2 and SDH6 and SDH4)	Citrate cycle (TCA cycle)	0	0	1000	0

NADH2-u6m	PICST_66598	PICST_66598	NDE1	Oxidative Phosphorylation	0	0	1000	0
CCP	(PICST_67176 or PICST_85478)	PICST_67176 PICST_85478	(CCP2 or HYP)	Oxidative Phosphorylation	0	0	1000	0
AOXIDASE	PICST_67332	PICST_67332	STO1	Oxidative Phosphorylation	0	0	1000	0
ATPSm	((PICST_69779 and PICST_77492 and PICST_91482 and PICST_86570 and PICST_81978 and PICST_78422 and PICST_79036 and PICST_30875 and PICST_32314 and PICST_81568 and PICST_65041) or (PICST_71868 and PICST_35827 and PICST_58102 and PICST_75615 and PICST_48891 and PICST_60924 and PICST_69588 and PICST_87235 and PICST_86847 and PICST_52299 and PICST_67737 and PICST_80851 and PICST_61950 and PICST_66160))	PICST_69779 PICST_77492 PICST_91482 PICST_86570 PICST_81978 PICST_78422 PICST_79036 PICST_30875 PICST_32314 PICST_81568 PICST_65041 PICST_71868 PICST_35827 PICST_58102 PICST_75615 PICST_48891 PICST_60924 PICST_69588 PICST_87235 PICST_86847 PICST_52299 PICST_67737 PICST_80851 PICST_61950 PICST_66160	((ATP16 and ATP2 and ATP3 and ATP1 and ATP5 and ATP4 and ATP7 and ATP17 and ATP14 and ATP18 and ATP20) or (VMA6 and VMA7 and VMA5 and TFP1 and VMA2 and VMA4 and CUP5 and TFP3 and PPA1 and VPH1 and VPH3 and VMA8 and VMA10 and VMA13))	Oxidative Phosphorylation	0	0	1000	0

CYOOm	(PICST_77110 and PICST_82299 and PICST_88762 and PICST_67314 and PICST_71543 and PICST_85465 and PICST_82827 and PICST_84097 and PICST_70893)	PICST_77110 PICST_82299 PICST_88762 PICST_67314 PICST_71543 PICST_85465 PICST_82827 PICST_84097 PICST_70893	(COX5a and COX6 and COX4 and COX13 and COX12 and COX7 and COX11 and COX15 and COX17)	Oxidative Phosphorylation	0	0	1000	0
PPA	PICST_83097	PICST_83097	IPP1	Oxidative Phosphorylation	0	0	1000	0
ATPS	PICST_87366	PICST_87366	PMA1	Oxidative Phosphorylation	0	0	1000	0
ADK2				Oxidative Phosphorylation	1	-1000	1000	0
TMPHi				Oxidative Phosphorylation	1	-1000	1000	0
POLYAO	PICST_28515	PICST_28515	HYP	Pantothenate and CoA Biosynthesis	0	0	1000	0
POLYAO2	PICST_28515	PICST_28515	HYP	Pantothenate and CoA Biosynthesis	0	0	1000	0
POLYAO3	PICST_28515	PICST_28515	HYP	Pantothenate and CoA Biosynthesis	0	0	1000	0
MOHMT	PICST_38073	PICST_38073	ECM31	Pantotheonate and CoA metabolism	0	0	1000	0
DPCOAK	PICST_41000	PICST_41000	COA5	Pantotheonate and CoA metabolism	0	0	1000	0
PTPAT	PICST_46142	PICST_46142	CAB4	Pantotheonate and CoA metabolism	0	0	1000	0
PPNCL2	PICST_54534	PICST_54534	CAB2	Pantotheonate and CoA metabolism	0	0	1000	0
DPRm	PICST_54699	PICST_54699	PAN5	Pantotheonate and CoA metabolism	0	0	1000	0
PNTK	PICST_57761	PICST_57761	HYP	Pantotheonate and CoA metabolism	0	0	1000	0
PANTS	PICST_57860	PICST_57860	PAN4	Pantotheonate and CoA metabolism	0	0	1000	0

PPCDC	PICST_62046	PICST_62046	CAB3	Pantotheonate and CoA metabolism	0	0	1000	0
DHAD1m	PICST_75802	PICST_75802	ILV3	Pantotheonate and CoA metabolism	0	0	1000	0
KARA1m	PICST_78299	PICST_78299	ILV5	Pantotheonate and CoA metabolism	0	0	1000	0
RBTDH	(PICST_36953 or PICST_53846 or PICST_80053)	PICST_36953 PICST_53846 PICST_80053	(TSC10 or AYR3 or YIM4)	Pentose and glucuronate interconversions	1	-1000	1000	0
XYLK	PICST_68734	PICST_68734	XKS1	Pentose and glucuronate interconversions	0	0	1000	0
XYLUR	PICST_86924	PICST_86924	XYL2	Pentose and glucuronate interconversions	1	-1000	1000	0
XYLR	PICST_89614	PICST_89614	XYL1	Alternate Carbon metabolism	0	0	1000	0
XYLR1	PICST_89614	PICST_89614	XYL1	Alternate Carbon metabolism	0	0	1000	0
XYLR2	PICST_89614	PICST_89614	XYL1	Alternate Carbon metabolism	0	0	0	0
ARABDR				Alternate Carbon metabolism	0	0	1000	0
ARABLR				Alternate Carbon metabolism	0	0	1000	0
DRIBOSER				Alternate Carbon metabolism	1	0	1000	0
LXYLULOSER				Alternate Carbon metabolism	1	-1000	1000	0
DARABITOLDHD				Pentose and glucuronate interconversions	1	-1000	1000	0
DRIBITOLDHD				Pentose and glucuronate interconversions	1	-1000	1000	0
LARABITOLDHD				Pentose and glucuronate interconversions	1	-1000	1000	0
RBLK				Pentose and glucuronate interconversions	0	0	1000	0
RIBUK				Pentose Phosphate Pathway	0	0	1000	0
GLULACT	PICST_28583	PICST_28583	CGR1	Pentose Phosphate Pathway	1	-1000	1000	0
PRPPS	(PICST_34307 or PICST_65580 or PICST_66107 or PICST_78248)	PICST_34307 PICST_65580 PICST_66107 PICST_78248	(KPR4 or PRS1 or PRS2 or PRS5)	Pentose Phosphate Pathway	1	0	1000	0

GLUCDH	(PICST_37336 or PICST_31562 or PICST_43416)	PICST_37336 PICST_31562 PICST_43416	(MDH98 or MDH97 or MDH99)	Pentose Phosphate Pathway	1	-1000	1000	0
RPE	PICST_50761	PICST_50761	RPE1	Pentose Phosphate Pathway	1	-1000	1000	0
TKT1	(PICST_53327 or PICST_67105)	PICST_53327 PICST_67105	(DHA1 or TKT1)	Pentose Phosphate Pathway	1	-1000	1000	0
TKT2	(PICST_53327 or PICST_67105)	PICST_53327 PICST_67105	(DHA1 or TKT1)	Pentose Phosphate Pathway	1	-1000	1000	0
PGL	(PICST_60598 or PICST_82104)	PICST_60598 PICST_82104	(SOL1 or SOL2)	Pentose Phosphate Pathway	0	0	1000	0
GND	PICST_69500	PICST_69500	GND1	Pentose Phosphate Pathway	0	0	1000	0
TALA	PICST_74289	PICST_74289	TAL1	Pentose Phosphate Pathway	1	-1000	1000	0
RBK	PICST_75489	PICST_75489	RBK1	Pentose Phosphate Pathway	0	0	1000	0
RPI	(PICST_76151 or PICST_57049)	PICST_76151 PICST_57049	(RKI1 or RPI1)	Pentose Phosphate Pathway	1	-1000	1000	0
GNK	PICST_83065	PICST_83065	GLK2	Pentose Phosphate Pathway	1	-1000	1000	0
G6PDH	PICST_85065	PICST_85065	ZWF1	Pentose Phosphate Pathway	0	0	1000	0
PPM	(PICST_77218 or PICST_91072)	PICST_77218 PICST_91072	(PGM2 or PGM3)	Glycolysis/Gluconeogenesis	1	-1000	1000	0
FACOAL120	(PICST_35069 or PICST_55002 or PICST_65071 or PICST_89450)	PICST_35069 PICST_55002 PICST_65071 PICST_89450	(FAA23 or FAA24 or FAA4 or FAA22)	Fatty acid Biosynthesis	1	-1000	1000	0
FACOAL200	(PICST_35069 or PICST_55002 or PICST_65071 or PICST_89450)	PICST_35069 PICST_55002 PICST_65071 PICST_89450	(FAA23 or FAA24 or FAA4 or FAA22)	Fatty acid Biosynthesis	1	-1000	1000	0
FACOAL220	(PICST_35069 or PICST_55002 or PICST_65071 or PICST_89450)	PICST_35069 PICST_55002 PICST_65071 PICST_89450	(FAA23 or FAA24 or FAA4 or FAA22)	Fatty acid Biosynthesis	1	-1000	1000	0

FACOAL240	(PICST_35069 or PICST_55002 or PICST_65071 or PICST_89450)	PICST_35069 PICST_55002 PICST_65071 PICST_89450	(FAA23 or FAA24 or FAA4 or FAA22)	Fatty acid Biosynthesis	1	-1000	1000	0
FACOAL260	(PICST_35069 or PICST_55002 or PICST_65071 or PICST_89450)	PICST_35069 PICST_55002 PICST_65071 PICST_89450	(FAA23 or FAA24 or FAA4 or FAA22)	Fatty acid Biosynthesis	1	-1000	1000	0
FACOAL80	(PICST_35069 or PICST_55002 or PICST_65071 or PICST_89450)	PICST_35069 PICST_55002 PICST_65071 PICST_89450	(FAA23 or FAA24 or FAA4 or FAA22)	Fatty acid Biosynthesis	1	-1000	1000	0
DESAT18_2	(PICST_72518 or PICST_81126)	PICST_72518 PICST_81126	(ODD1 or HYP)	Fatty acid Biosynthesis	0	0	1000	0
ALDHPAC1	PICST_28221	PICST_28221	ALD7	Phenylalanine Metabolism	0	0	1000	0
INDPYRDC	PICST_62095	PICST_62095	ARO10	Phenylalanine Metabolism	0	0	1000	0
PPYRDC	PICST_62095	PICST_62095	ARO10	Phenylalanine Metabolism	0	0	1000	0
PPNDH	PICST_33690	PICST_33690	HYP	Phenylalanine, Tyrosine and Trytophan Biosynthesis	0	0	1000	0
ANPRT	PICST_34016	PICST_34016	TRP4	Phenylalanine, Tyrosine and Trytophan Biosynthesis	0	0	1000	0
PRAI	PICST_38245	PICST_38245	TRP1	Phenylalanine, Tyrosine and Trytophan Biosynthesis	0	0	1000	0
TYRTAm	(PICST_51039 or PICST_80440)	PICST_51039 PICST_80440	(AAT22 or AAT1)	Phenylalanine, Tyrosine and Trytophan Biosynthesis	1	-1000	1000	0
PPND	PICST_52223	PICST_52223	TYR1	Phenylalanine, Tyrosine and Trytophan Biosynthesis	0	0	1000	0
DHQT	PICST_62322	PICST_62322	DQD1	Phenylalanine, Tyrosine and Trytophan Biosynthesis	0	0	1000	0
TYRTA	PICST_66059	PICST_66059	AAT2	Phenylalanine, Tyrosine and Trytophan Biosynthesis	1	-1000	0	0
PHETA1	(PICST_66767 or PICST_34099)	PICST_66767 PICST_34099	(ARO8 or ARO9.1)	Phenylalanine, Tyrosine and Trytophan Biosynthesis	1	-1000	1000	0

TRPTA	(PICST_66767 or PICST_34099)	PICST_66767 PICST_34099	(ARO8 or ARO9.1)	Phenylalanine, Tyrosine and Trytophan Biosynthesis	1	-1000	1000	0
TYRTA1	(PICST_66767 or PICST_34099)	PICST_66767 PICST_34099	(ARO8 or ARO9.1)	Phenylalanine, Tyrosine and Trytophan Biosynthesis	0	0	1000	0
TRPS1	PICST_69669	PICST_69669	TRP5	Phenylalanine, Tyrosine and Trytophan Biosynthesis	0	0	1000	0
CHORM	PICST_73224	PICST_73224	ARO7	Phenylalanine, Tyrosine and Trytophan Biosynthesis	0	0	1000	0
ANS	((PICST_74393 and PICST_91065) or (PICST_85710 and PICST_91065))	PICST_74393 PICST_91065 PICST_85710 PICST_91065	((ABZ1 and TRP6) or (TRP2 and TRP6))	Phenylalanine, Tyrosine and Trytophan Biosynthesis	0	0	1000	0
DDPA	(PICST_80434 or PICST_83093)	PICST_80434 PICST_83093	(ARO4 or ARO3)	Phenylalanine, Tyrosine and Trytophan Biosynthesis	0	0	1000	0
DHQS	PICST_89141	PICST_89141	HYP	Phenylalanine, Tyrosine and Trytophan Biosynthesis	0	0	1000	0
PSCVT	PICST_89141	PICST_89141	HYP	Phenylalanine, Tyrosine and Trytophan Biosynthesis	0	0	1000	0
SHK3D	PICST_89141	PICST_89141	HYP	Phenylalanine, Tyrosine and Trytophan Biosynthesis	0	0	1000	0
SHKK	PICST_89141	PICST_89141	HYP	Phenylalanine, Tyrosine and Trytophan Biosynthesis	0	0	1000	0
CHORS	(PICST_90939 or PICST_89141)	PICST_90939 PICST_89141	(AROC or HYP)	Phenylalanine, Tyrosine and Trytophan Biosynthesis	0	0	1000	0
IGPS	PICST_91065	PICST_91065	TRP6	Phenylalanine, Tyrosine and Trytophan Biosynthesis	0	0	1000	0
DAGCPT	PICST_40464	PICST_40464	EPT1	Phospholipid Biosynthesis	0	0	1000	0
MI1PP	PICST_63274	PICST_63274	HYP	Phospholipid Biosynthesis	0	0	1000	0
LPCAT	PICST_89421	PICST_89421	HYP	Phospholipid Biosynthesis	0	0	1000	0
PLBPC	PICST_33358	PICST_33358	HYP	Phospholipid Metabolism	0	0	1000	0
GPDDA1	PICST_35844	PICST_35844	GLP1	Phospholipid Metabolism	0	0	1000	0
ETHAPT	PICST_40464	PICST_40464	EPT1	Phospholipid Metabolism	1	-1000	1000	0

PLBP1Ie	(PICST_48846 or PICST_81360 or PICST_82807)	PICST_48846 PICST_81360 PICST_82807	PLB6 or PLB4 or PLB1	Phospholipid Metabolism	0	0	1000	0
PLBPCe	(PICST_48846 or PICST_81360 or PICST_82807)	PICST_48846 PICST_81360 PICST_82807	PLB6 or PLB4 or PLB1	Phospholipid Metabolism	0	0	1000	0
CLPNSm	PICST_52642	PICST_52642	CRD2	Phospholipid Metabolism	0	0	1000	0
PETOHM	PICST_57055	PICST_57055	CHO2	Phospholipid Metabolism	0	0	1000	0
DASYN	PICST_78259	PICST_78259	HYP	Phospholipid Metabolism	1	-1000	1000	0
DASYNm	PICST_78259	PICST_78259	HYP	Phospholipid Metabolism	1	-1000	1000	0
UPP3MT	PICST_31820	PICST_31820	MET1	Porphyrin and chlorophyll metabolism	0	0	1000	0
FCLTm	PICST_36859	PICST_36859	HEM15	Porphyrin and chlorophyll metabolism	0	0	1000	0
UPPDC1	PICST_46716	PICST_46716	HEM12	Porphyrin and chlorophyll metabolism	0	0	1000	0
HMBS	PICST_47071	PICST_47071	HYP	Porphyrin and chlorophyll metabolism	0	0	1000	0
HEMEOSm	PICST_47531	PICST_47531	COX10	Porphyrin and chlorophyll metabolism	0	0	1000	0
PPBNGS	PICST_54024	PICST_54024	HYP	Porphyrin and chlorophyll metabolism	0	0	1000	0
PPPGOm	PICST_55152	PICST_55152	HEM14	Porphyrin and chlorophyll metabolism	0	0	1000	0
UPP3S	PICST_59732	PICST_59732	HEM4	Porphyrin and chlorophyll metabolism	0	0	1000	0
SHCHD	PICST_63116	PICST_63116	MET8	Porphyrin and chlorophyll metabolism	0	0	1000	0
SHCHF	PICST_63116	PICST_63116	MET8	Porphyrin and chlorophyll metabolism	0	0	1000	0
CPPPGO	PICST_64368	PICST_64368	HEM13	Porphyrin and chlorophyll metabolism	0	0	1000	0
HEMEOMOm	PICST_84097	PICST_84097	COX15	Porphyrin and chlorophyll metabolism	0	0	1000	0

MCITSm	PICST_85554	PICST_85554	CIT1	Anaplerotic reactions	0	0	1000	0
3HPPCOAHL	(PICST_30193 or PICST_32846)	PICST_30193 PICST_32846	(ECH1 or EHO3)	Propanoate Metabolism	1	-1000	1000	0
ACCOADH	PICST_33103	PICST_33103	ACD99	Propanoate Metabolism	1	-1000	1000	0
ALABAKGAT	(PICST_46781 or PICST_54153)	PICST_46781 PICST_54153	(UGA1.2 or UGA1.1)	Propanoate Metabolism	1	-1000	1000	0
MMSAD1	PICST_80168	PICST_80168	ALD6	Propanoate Metabolism	0	0	1000	0
ADPT	PICST_29461	PICST_29461	APT1	Purine Metabolism	0	0	1000	0
RNDR1	(((PICST_29856 and PICST_70259) and PICST_39568) or ((PICST_75662 and PICST_70259) and PICST_39568))	PICST_29856 PICST_70259 PICST_39568 PICST_75662 PICST_70259 PICST_39568	(((RNR1.1 and RNR2) and TRX1) or ((RNR1.2 and RNR2) and TRX1))	Purine Metabolism	0	0	1000	0
RNDR2	(((PICST_29856 and PICST_70259) and PICST_39568) or ((PICST_75662 and PICST_70259) and PICST_39568))	PICST_29856 PICST_70259 PICST_39568 PICST_75662 PICST_70259 PICST_39568	(((RNR1.1 and RNR2) and TRX1) or ((RNR1.2 and RNR2) and TRX1))	Purine Metabolism	0	0	1000	0
NTD10	(PICST_32593 or PICST_47650)	PICST_32593 PICST_47650	(PHO3 or PHO3.2)	Purine Metabolism	0	0	1000	0
NTD2	(PICST_32593 or PICST_47650)	PICST_32593 PICST_47650	(PHO3 or PHO3.2)	Purine Metabolism	0	0	1000	0
NTD3	(PICST_32593 or PICST_47650)	PICST_32593 PICST_47650	(PHO3 or PHO3.2)	Purine Metabolism	0	0	1000	0
NTD5	(PICST_32593 or PICST_47650)	PICST_32593 PICST_47650	(PHO3 or PHO3.2)	Purine Metabolism	0	0	1000	0
NTD6	(PICST_32593 or PICST_47650)	PICST_32593 PICST_47650	(PHO3 or PHO3.2)	Purine Metabolism	0	0	1000	0
NTD7	(PICST_32593 or PICST_47650)	PICST_32593 PICST_47650	(PHO3 or PHO3.2)	Purine Metabolism	0	0	1000	0
NTD8	(PICST_32593 or PICST_47650)	PICST_32593 PICST_47650	(PHO3 or PHO3.2)	Purine Metabolism	0	0	1000	0

NTD9	(PICST_32593 or PICST_47650)	PICST_32593 PICST_47650	(PHO3 or PHO3.2)	Purine Metabolism	0	0	1000	0
GARFT	PICST_34864	PICST_34864	PUR3	Purine Metabolism	0	0	1000	0
ALLTNASE	PICST_34891	PICST_34891	ALN1	Purine Metabolism	0	0	1000	0
GUAD	PICST_36448	PICST_36448	GAH1	Purine Metabolism	0	0	1000	0
URIC	PICST_40781	PICST_40781	URO1	Purine Metabolism	0	0	1000	0
AMPDA	PICST_40926	PICST_40926	AMD1	Purine Metabolism	0	0	1000	0
NTETPA	(PICST_42119 or PICST_56209)	PICST_42119 PICST_56209	(HNT1 or HNT2)	Purine Metabolism	0	0	1000	0
NTETPG	(PICST_42119 or PICST_56209)	PICST_42119 PICST_56209	(HNT1 or HNT2)	Purine Metabolism	0	0	1000	0
NTETPU	(PICST_42119 or PICST_56209)	PICST_42119 PICST_56209	(HNT1 or HNT2)	Purine Metabolism	0	0	1000	0
NTETPX	(PICST_42119 or PICST_56209)	PICST_42119 PICST_56209	(HNT1 or HNT2)	Purine Metabolism	0	0	1000	0
UDCH	PICST_43701	PICST_43701	DAL3	Purine Metabolism	0	0	1000	0
ATPATF2	PICST_48616	PICST_48616	APA2	Purine Metabolism	0	0	1000	0
ATPATF3	PICST_48616	PICST_48616	APA2	Purine Metabolism	0	0	1000	0
PRASCS	PICST_49451	PICST_49451	PUR7	Purine Metabolism	1	-1000	1000	0
ALLTAH	PICST_55822	PICST_55822	DAL2	Purine Metabolism	1	-1000	1000	0
ADNK1	PICST_57331	PICST_57331	ADO1	Purine Metabolism	0	0	1000	0
ADA	PICST_59156	PICST_59156	AAH1	Purine Metabolism	0	0	1000	0
AP4AH	PICST_62390	PICST_62390	APA4	Purine Metabolism	0	0	1000	0
ADK1m	PICST_63834	PICST_63834	ADK2	Purine Metabolism	1	-1000	1000	0
ADK1	PICST_65737	PICST_65737	ADK1	Purine Metabolism	1	-1000	1000	0
IMPD	PICST_65804	PICST_65804	HYP	Purine Metabolism	0	0	1000	0
ADNCYC	(PICST_67440 and PICST_67704)	PICST_67440 PICST_67704	(CDC35 and SDS2)	Purine Metabolism	0	0	1000	0
NDPK1	PICST_67856	PICST_67856	YNK1	Purine Metabolism	1	0	1000	0
NDPK5	PICST_67856	PICST_67856	YNK1	Purine Metabolism	1	0	1000	0
NDPK8	PICST_67856	PICST_67856	YNK1	Purine Metabolism	1	0	1000	0
NDPK9	PICST_67856	PICST_67856	YNK1	Purine Metabolism	1	-1000	1000	0
AICART	PICST_68505	PICST_68505	PUR92	Purine Metabolism	1	-1000	1000	0
IMPC	PICST_68505	PICST_68505	PUR92	Purine Metabolism	1	-1000	1000	0

GMPS	PICST_70121	PICST_70121	GUA1	Purine Metabolism	0	0	1000	0
GK1	PICST_72675	PICST_72675	GUK1	Purine Metabolism	1	-1000	1000	0
GK2	PICST_72675	PICST_72675	GUK1	Purine Metabolism	1	-1000	1000	0
GK3	PICST_72675	PICST_72675	GUK1	Purine Metabolism	1	-1000	1000	0
NDPADP	PICST_75213	PICST_75213	YND1	Purine Metabolism	0	0	1000	0
NDPCDP	PICST_75213	PICST_75213	YND1	Purine Metabolism	0	0	1000	0
NTPCTP	PICST_75213	PICST_75213	YND1	Purine Metabolism	0	0	1000	0
NTPGTP	PICST_75213	PICST_75213	YND1	Purine Metabolism	0	0	1000	0
NDPIDP	(PICST_75213 or PICST_72696)	PICST_75213 PICST_72696	(YND1 or HAM1)	Purine Metabolism	0	0	1000	0
NTPITP	(PICST_75213 or PICST_72696)	PICST_75213 PICST_72696	(YND1 or HAM1)	Purine Metabolism	0	0	1000	0
PRFGS	PICST_75569	PICST_75569	ADE6	Purine Metabolism	0	0	1000	0
PRAGS	PICST_78660	PICST_78660	HYP	Purine Metabolism	1	-1000	1000	0
PRAIS	PICST_78660	PICST_78660	HYP	Purine Metabolism	0	0	1000	0
PDE1	PICST_78746	PICST_78746	PDE2	Purine Metabolism	0	0	1000	0
AIRC	PICST_81265	PICST_81265	ADE2	Purine Metabolism	1	-1000	1000	0
NDPGDP	(PICST_82228 or PICST_75213)	PICST_82228 PICST_75213	(GDA1 or YND1)	Purine Metabolism	0	0	1000	0
NDPUDP	(PICST_82228 or PICST_75213)	PICST_82228 PICST_75213	(GDA1 or YND1)	Purine Metabolism	0	0	1000	0
PYK1	PICST_83166	PICST_83166	PYK1	Purine Metabolism	0	0	1000	0
ADSL1	PICST_84718	PICST_84718	ADE13	Purine Metabolism	1	-1000	1000	0
ADSL2	PICST_84718	PICST_84718	ADE13	Purine Metabolism	1	-1000	1000	0
ADSS	PICST_85387	PICST_85387	HYP	Purine Metabolism	0	0	1000	0
DURIK1				Purine Metabolism	0	0	1000	0
PUNP7				Purine Metabolism	1	-1000	1000	0
XANDa				Purine Metabolism	0	0	1000	0
XANDb				Purine Metabolism	0	0	1000	0
ADSK	PICST_90976	PICST_90976	KAP1	Sulphur Metabolism	0	0	1000	0
DADA	PICST_59156	PICST_59156	AAH1	Nucleotide Salvage Pathway	0	0	1000	0
DADK				Nucleotide Salvage Pathway	1	-1000	1000	0
PUNP1				Nucleotide Salvage Pathway	1	-1000	1000	0
PUNP2				Nucleotide Salvage Pathway	1	-1000	1000	0

PUNP3				Nucleotide Salvage Pathway	1	-1000	1000	0
PUNP4				Nucleotide Salvage Pathway	1	-1000	1000	0
PUNP5				Nucleotide Salvage Pathway	1	-1000	1000	0
PUNP6				Nucleotide Salvage Pathway	1	-1000	1000	0
NTD4	(PICST_32593 or PICST_47650)	PICST_32593 PICST_47650	(PHO3 or PHO3.2)	Purine Metabolism	0	0	1000	0
NDPK4	PICST_67856	PICST_67856	YNK1	Purine Metabolism	1	-1000	1000	0
NDPK6	PICST_67856	PICST_67856	YNK1	Purine Metabolism	1	-1000	1000	0
NDPK7	PICST_67856	PICST_67856	YNK1	Purine Metabolism	1	-1000	1000	0
RNDR3	(((PICST_29856 and PICST_70259) and PICST_39568) or ((PICST_75662 and PICST_70259) and PICST_39568))	PICST_29856 PICST_70259 PICST_39568 PICST_75662 PICST_70259 PICST_39568	(((RNR1.1 and RNR2) and TRX1) or ((RNR1.2 and RNR2) and TRX1))	Purine Metabolism	0	0	1000	0
RNDR4	(((PICST_29856 and PICST_70259) and PICST_39568) or ((PICST_75662 and PICST_70259) and PICST_39568))	PICST_29856 PICST_70259 PICST_39568 PICST_75662 PICST_70259 PICST_39568	(((RNR1.1 and RNR2) and TRX1) or ((RNR1.2 and RNR2) and TRX1))	Purine Metabolism	0	0	1000	0
CSND	(PICST_30000 and PICST_78481)	PICST_30000 PICST_78481	(TAD3 and FCY1)	Pyrimidine Metabolism	0	0	1000	0
DHORTS	PICST_32048	PICST_32048	URA4	Pyrimidine Metabolism	1	-1000	1000	0
DCMPDA	PICST_34441	PICST_34441	DCD1	Pyrimidine Metabolism	1	-1000	1000	0
TMDS	PICST_37780	PICST_37780	TMP1	Pyrimidine Metabolism	0	0	1000	0
CYTD	PICST_39066	PICST_39066	HYP	Pyrimidine Metabolism	0	0	1000	0
DCYTD	PICST_39066	PICST_39066	HYP	Pyrimidine Metabolism	0	0	1000	0
UPPRT	PICST_47473	PICST_47473	FUR1	Pyrimidine Metabolism	0	0	1000	0
OMPDC	PICST_50417	PICST_50417	URA3	Pyrimidine Metabolism	0	0	1000	0
DTMPK	PICST_53211	PICST_53211	CDC8	Pyrimidine Metabolism	1	-1000	1000	0
DHORD	PICST_55711	PICST_55711	URA1	Pyrimidine Metabolism	0	0	1000	0
TRDR	PICST_59792	PICST_59792	TRR1	Pyrimidine Metabolism	0	0	1000	0
DUTPDP	PICST_69362	PICST_69362	DUT1	Pyrimidine Metabolism	0	0	1000	0

CTPS1	PICST_78143	PICST_78143	URA7	Pyrimidine Metabolism	0	0	1000	0
CTPS2	PICST_78143	PICST_78143	URA7	Pyrimidine Metabolism	0	0	1000	0
URIK1	PICST_81319	PICST_81319	URK1	Pyrimidine Metabolism	0	0	1000	0
URIK2	PICST_81319	PICST_81319	URK1	Pyrimidine Metabolism	0	0	1000	0
ASPCT	(PICST_90563 or (PICST_50410 and PICST_28533))	PICST_90563 PICST_50410 PICST_28533	(URA2 or (CPA2 and CPA1))	Pyrimidine Metabolism	0	0	1000	0
CYTK1	PICST_90609	PICST_90609	URA6	Pyrimidine Metabolism	1	-1000	1000	0
CYTK2	PICST_90609	PICST_90609	URA6	Pyrimidine Metabolism	1	-1000	1000	0
UMPK	PICST_90609	PICST_90609	URA6	Pyrimidine Metabolism	1	-1000	1000	0
URIDK	PICST_90609	PICST_90609	URA6	Pyrimidine Metabolism	1	-1000	1000	0
ORPT	PICST_90848	PICST_90848	URA5	Pyrimidine Metabolism	1	-1000	1000	0
DURIPP				Nucleotide Salvage Pathway	1	-1000	1000	0
NTD1	(PICST_32593 or PICST_47650)	PICST_32593 PICST_47650	(PHO3 or PHO3.2)	Purine Metabolism	0	0	1000	0
NTD11	(PICST_32593 or PICST_47650)	PICST_32593 PICST_47650	(PHO3 or PHO3.2)	Purine Metabolism	0	0	1000	0
NDPK2	PICST_67856	PICST_67856	YNK1	Purine Metabolism	1	-1000	1000	0
NDPK3	PICST_67856	PICST_67856	YNK1	Purine Metabolism	1	-1000	1000	0
NTPUTP	PICST_75213	PICST_75213	YND1	Purine Metabolism	0	0	1000	0
MGSA				Alternate Carbon metabolism	0	0	1000	0
ALCDH	(PICST_27980 or PICST_68558)	PICST_27980 PICST_68558	(ADH1 or ADH2)	Glycolysis/Gluconeogenesis	1	-1000	0	0
ALDDH	PICST_28221	PICST_28221	ALD7	Glycolysis/Gluconeogenesis	0	0	1000	0
HCITSm	(PICST_28701 or PICST_89386)	PICST_28701 PICST_89386	(LYS21 or LYS22)	Pyruvate Metabolism	0	0	1000	0
GLYOX	PICST_31656	PICST_31656	GLO2	Pyruvate Metabolism	0	0	1000	0
ACACT1	PICST_31707	PICST_31707	ERG10	Pyruvate Metabolism	0	0	1000	0
ME1m	PICST_89732	PICST_89732	(MDHM or MDH1 or MDH2)	Citrate cycle (TCA cycle)	0	0	1000	0
ALDDHm	(PICST_44169 or PICST_60847)	PICST_44169 PICST_60847	(ALD4 or ALD3)	Glycolysis/Gluconeogenesis	0	0	1000	0
ACLSm	(PICST_44973 and PICST_80460)	PICST_44973 PICST_80460	(ILV2 and ILV6)	Butanoate Metabolism	0	0	1000	0

L-LACDm	PICST_53263	PICST_53263	CYB3	Pyruvate Metabolism	0	0	1000	0
MALS	(PICST_53620 or PICST_90814)	PICST_53620 PICST_90814	(MLS1.1 or MLS1.2)	Pyruvate Metabolism	0	0	1000	0
ALDDH1	PICST_63844	PICST_63844	ALD2	Glycolysis/Gluconeogenesis	0	0	1000	0
LGTHL	PICST_64811	PICST_64811	GLO1	Pyruvate Metabolism	0	0	1000	0
PYRDC	(PICST_64926 or PICST_86443)	PICST_64926 PICST_86443	(PDC1 or PDC2)	Pyruvate Metabolism	0	0	1000	0
IPPS	PICST_68770	PICST_68770	LEU4	Pyruvate Metabolism	0	0	1000	0
ACCOAH	PICST_78208	PICST_78208	ACH1	Pyruvate Metabolism	0	0	1000	0
ACS1m	(PICST_79135 or PICST_89873)	PICST_79135 PICST_89873	(ACS2 or ACS1)	Glycolysis/Gluconeogenesis	0	0	1000	0
ACS1	(PICST_79135 or PICST_89873)	PICST_79135 PICST_89873	(ACS2 or ACS1)	Glycolysis/Gluconeogenesis	0	0	1000	0
PDHm	((PICST_80322 and PICST_68238) and (PICST_81177 or PICST_82614) and PICST_36951)	PICST_80322 PICST_68238 PICST_81177 PICST_82614 PICST_36951	((PDA1 and PDB1) and (PDX1 or LAT1) and LPD1)	Glycolysis/Gluconeogenesis	0	0	1000	0
ACCOAC	PICST_81158	PICST_81158	ACC1	Pyruvate Metabolism	1	-1000	1000	0
ACCOACrm	PICST_81158	PICST_81158	ACC1	Pyruvate Metabolism	1	-1000	1000	0
D-LACDm	(PICST_86747 or PICST_89565 or PICST_31583 or PICST_52647)	PICST_86747 PICST_89565 PICST_31583 PICST_52647	(DLD1 or DLD2 or DLD3 or DLD4)	Pyruvate Metabolism	0	0	1000	0
MDH	(PICST_40132 or PICST_66451 or PICST_78343)	PICST_40132 PICST_66451 PICST_78343	MAE1	Pyruvate Metabolism	1	0	1000	0
PC	PICST_90975	PICST_90975	PYC1	Citrate cycle (TCA cycle)	0	0	1000	0
PPCK	PICST_71471	PICST_71471	PCK1	Glycolysis/Gluconeogenesis	0	0	1000	0
2HMHMBQMTm	PICST_31991	PICST_31991	COQ3	Quinone Synthesis	0	0	1000	0
3DH5HPBMTm	PICST_31991	PICST_31991	COQ3	Quinone Synthesis	0	0	1000	0
2HP6MPMOm	(PICST_32861 or PICST_73817)	PICST_32861 PICST_73817	(COQ6 or CAT5)	Quinone Synthesis	0	0	1000	0

2HPMMBQMOm	(PICST_32861 or PICST_73817)	PICST_32861 PICST_73817	(COQ6 or CAT5)	Quinone Synthesis	0	0	1000	0
2HPMBQMTm	PICST_71892	PICST_71892	COQ5	Quinone Synthesis	0	0	1000	0
PPTTm	PICST_86778	PICST_86778	COQ1	Quinone Synthesis	0	0	1000	0
HBZOPT6m	PICST_87025	PICST_87025	COQ2	Quinone Synthesis	0	0	1000	0
3HPH5MBDCm				Quinone Synthesis	0	0	1000	0
3OPHB5Hm				Quinone Synthesis	0	0	1000	0
GTPCII	PICST_30471	PICST_30471	RIB1	Riboflavin Metabolism	0	0	1000	0
FMNAT	PICST_40011	PICST_40011	HYP	Riboflavin Metabolism	0	0	1000	0
RBFSa	PICST_40556	PICST_40556	RIB5	Riboflavin Metabolism	0	0	1000	0
ACP1	(PICST_46121 or PICST_46975 or PICST_61096 or PICST_83142)	PICST_46121 PICST_46975 PICST_61096 PICST_83142	(PHO12 or PHO5 or PHO6 or PHO3.3)	Riboflavin Metabolism	0	0	1000	0
RBFK	PICST_50354	PICST_50354	FMN1	Riboflavin Metabolism	0	0	1000	0
RBFSb	PICST_51333	PICST_51333	RIB4	Riboflavin Metabolism	0	0	1000	0
APRAUR	PICST_53707	PICST_53707	HYP	Riboflavin Metabolism	0	0	1000	0
DHPPDA	PICST_53707	PICST_53707	HYP	Riboflavin Metabolism	0	0	1000	0
DB4PS	PICST_65046	PICST_65046	RIB3	Riboflavin Metabolism	0	0	1000	0
PMDPHT	(PICST_74933 or PICST_82137)	PICST_74933 PICST_82137	(YUB4 or YMR1)	Riboflavin Metabolism	0	0	1000	0
SBPP1er	PICST_28253	PICST_28253	HYP	Sphingolipid Metabolism	0	0	1000	0
SBPP2er	PICST_28253	PICST_28253	HYP	Sphingolipid Metabolism	0	0	1000	0
BGALGLUC	PICST_30036	PICST_30036	LAC4	Sphingolipid Metabolism	0	0	1000	0
CERS124	(PICST_32904 or PICST_45900)	PICST_32904 PICST_45900	(LAG1.1 or LAG1.3)	Sphingolipid Metabolism	0	0	1000	0
CERS126	(PICST_32904 or PICST_45900)	PICST_32904 PICST_45900	(LAG1.1 or LAG1.3)	Sphingolipid Metabolism	0	0	1000	0
CERS224	(PICST_32904 or PICST_45900)	PICST_32904 PICST_45900	(LAG1.1 or LAG1.3)	Sphingolipid Metabolism	0	0	1000	0
CERS226	(PICST_32904 or PICST_45900)	PICST_32904 PICST_45900	(LAG1.1 or LAG1.3)	Sphingolipid Metabolism	0	0	1000	0
IPC124PLC	PICST_33012	PICST_33012	HYP	Sphingolipid Metabolism	0	0	1000	0
IPC126PLC	PICST_33012	PICST_33012	HYP	Sphingolipid Metabolism	0	0	1000	0

IPC224PLC	PICST_33012	PICST_33012	HYP	Sphingolipid Metabolism	0	0	1000	0
IPC226PLC	PICST_33012	PICST_33012	HYP	Sphingolipid Metabolism	0	0	1000	0
MIP2C124PLC	PICST_33012	PICST_33012	HYP	Sphingolipid Metabolism	0	0	1000	0
MIP2C126PLC	PICST_33012	PICST_33012	HYP	Sphingolipid Metabolism	0	0	1000	0
MIP2C224PLC	PICST_33012	PICST_33012	HYP	Sphingolipid Metabolism	0	0	1000	0
MIP2C226PLC	PICST_33012	PICST_33012	HYP	Sphingolipid Metabolism	0	0	1000	0
MIPC124PLC	PICST_33012	PICST_33012	HYP	Sphingolipid Metabolism	0	0	1000	0
MIPC126PLC	PICST_33012	PICST_33012	HYP	Sphingolipid Metabolism	0	0	1000	0
MIPC224PLC	PICST_33012	PICST_33012	HYP	Sphingolipid Metabolism	0	0	1000	0
MIPC226PLC	PICST_33012	PICST_33012	HYP	Sphingolipid Metabolism	0	0	1000	0
CERGLUT	PICST_34303	PICST_34303	CGT1	Sphingolipid Metabolism	0	0	1000	0
3DSPHR	PICST_36953	PICST_36953	TSC10	Sphingolipid Metabolism	0	0	1000	0
MIP2CS124	PICST_49558	PICST_49558	HYP	Sphingolipid Metabolism	0	0	1000	0
MIP2CS126	PICST_49558	PICST_49558	HYP	Sphingolipid Metabolism	0	0	1000	0
MIP2CS224	PICST_49558	PICST_49558	HYP	Sphingolipid Metabolism	0	0	1000	0
MIP2CS226	PICST_49558	PICST_49558	HYP	Sphingolipid Metabolism	0	0	1000	0
SPMPDE	PICST_51970	PICST_51970	IFP3	Sphingolipid Metabolism	0	0	1000	0
SLCBK1	PICST_54239	PICST_54239	SPH1	Sphingolipid Metabolism	0	0	1000	0
SLCBK2	PICST_54239	PICST_54239	SPH1	Sphingolipid Metabolism	0	0	1000	0
FADESAT	PICST_54788	PICST_54788	FAD3	Sphingolipid Metabolism	0	0	1000	0
FADESAT1	PICST_54788	PICST_54788	FAD3	Sphingolipid Metabolism	0	0	1000	0
CERASE124er	PICST_63351	PICST_63351	HYP	Sphingolipid Metabolism	0	0	1000	0
CERASE126er	PICST_63351	PICST_63351	HYP	Sphingolipid Metabolism	0	0	1000	0
CERASE224er	PICST_63351	PICST_63351	HYP	Sphingolipid Metabolism	0	0	1000	0
CERASE226er	PICST_63351	PICST_63351	HYP	Sphingolipid Metabolism	0	0	1000	0
CERH124	PICST_77904	PICST_77904	SUR2	Sphingolipid Metabolism	0	0	1000	0
CERH126	PICST_77904	PICST_77904	SUR2	Sphingolipid Metabolism	0	0	1000	0
PSPHS	PICST_77904	PICST_77904	SUR2	Sphingolipid Metabolism	0	0	1000	0
MIPCS124	PICST_83423	PICST_83423	HYP	Sphingolipid Metabolism	0	0	1000	0
MIPCS126	PICST_83423	PICST_83423	HYP	Sphingolipid Metabolism	0	0	1000	0
MIPCS224	PICST_83423	PICST_83423	HYP	Sphingolipid Metabolism	0	0	1000	0
MIPCS226	PICST_83423	PICST_83423	HYP	Sphingolipid Metabolism	0	0	1000	0
SERPT	(PICST_84331 and PICST_90811)	PICST_84331 PICST_90811	(LCB2 and LCB1)	Sphingolipid Metabolism	0	0	1000	0

PSPHPL	PICST_87778	PICST_87778	HYP	Sphingolipid Metabolism	0	0	1000	0
SPHPL	PICST_87778	PICST_87778	HYP	Sphingolipid Metabolism	0	0	1000	0
IPCS124	PICST_91106	PICST_91106	HYP	Sphingolipid Metabolism	0	0	1000	0
IPCS126	PICST_91106	PICST_91106	HYP	Sphingolipid Metabolism	0	0	1000	0
IPCS224	PICST_91106	PICST_91106	HYP	Sphingolipid Metabolism	0	0	1000	0
IPCS226	PICST_91106	PICST_91106	HYP	Sphingolipid Metabolism	0	0	1000	0
CERSYN				Sphingolipid Metabolism	1	-1000	1000	0
MALT	(PICST_29292 or PICST_42120 or PICST_56703 or PICST_85073)	PICST_29292 PICST_42120 PICST_56703 PICST_85073	(MAL9 or MAL6 or CGA1 or ROT2)	Galactose Metabolism	0	0	1000	0
PGMT	(PICST_77218 or PICST_91072)	PICST_77218 PICST_91072	(PGM2 or PGM3)	Glycolysis/Gluconeogenesis	1	-1000	1000	0
GALU	PICST_40866	PICST_40866	UGP1	Pentose and glucuronate interconversions	1	-1000	1000	0
C5STDS	PICST_28450	PICST_28450	ERG3	Sterol Biosynthesis	0	0	1000	0
C24STR	PICST_29811	PICST_29811	ERG4	Sterol Biosynthesis	0	0	1000	0
C14STR	PICST_36016	PICST_36016	HYP	Sterol Biosynthesis	0	0	1000	0
C3STKR1	PICST_61325	PICST_61325	ERG27	Sterol Biosynthesis	0	0	1000	0
C3STKR2	PICST_61325	PICST_61325	ERG27	Sterol Biosynthesis	0	0	1000	0
LNS14DM	PICST_63421	PICST_63421	CP51	Sterol Biosynthesis	0	0	1000	0
LNSTLS	PICST_63421	PICST_63421	ERG7	Sterol Biosynthesis	0	0	1000	0
C3STDH1	PICST_71224	PICST_71224	ERG26	Sterol Biosynthesis	0	0	1000	0
C3STDH2	PICST_71224	PICST_71224	ERG26	Sterol Biosynthesis	0	0	1000	0
C22STDS	PICST_73043	PICST_73043	ERG5	Sterol Biosynthesis	0	0	1000	0
C22STDS1	PICST_73043	PICST_73043	ERG5	Sterol Biosynthesis	0	0	1000	0
C8STI	PICST_73274	PICST_73274	ERG2	Sterol Biosynthesis	0	0	1000	0
C4STMO1	PICST_74706	PICST_74706	ERG25	Sterol Biosynthesis	0	0	1000	0
C4STMO2	PICST_74706	PICST_74706	ERG25	Sterol Biosynthesis	0	0	1000	0
SQLE	PICST_75910	PICST_75910	ERG1	Sterol Biosynthesis	0	0	1000	0
SAM24MT	PICST_80971	PICST_80971	HYP	Sterol Biosynthesis	0	0	1000	0
SQLS	PICST_83429	PICST_83429	ERG9	Sterol Biosynthesis	0	0	1000	0

BGLe	(PICST_1541 or PICST_34123 or PICST_41452 or PICST_51227 or PICST_61725 or PICST_89784 or PICST_37797 or PICST_65767)	PICST_1541 PICST_34123 PICST_41452 PICST_51227 PICST_61725 PICST_89784 PICST_37797 PICST_65767	(BGL1 or BGL5 or BGL2 or BGL6 or BGL3 or SUN4 or BGL4 or BGL7)	Sucrose and Starch Metabolism	0	0	1000	0
BGLMADG	(PICST_1541 or PICST_34123 or PICST_41452 or PICST_51227 or PICST_61725 or PICST_89784 or PICST_37797 or PICST_65767)	PICST_1541 PICST_34123 PICST_41452 PICST_51227 PICST_61725 PICST_89784 PICST_37797 PICST_65767	(BGL1 or BGL5 or BGL2 or BGL6 or BGL3 or SUN4 or BGL4 or BGL7)	Sucrose and Starch Metabolism	0	0	1000	0
BGLMBDG	(PICST_1541 or PICST_34123 or PICST_41452 or PICST_51227 or PICST_61725 or PICST_89784 or PICST_37797 or PICST_65767)	PICST_1541 PICST_34123 PICST_41452 PICST_51227 PICST_61725 PICST_89784 PICST_37797 PICST_65767	(BGL1 or BGL5 or BGL2 or BGL6 or BGL3 or SUN4 or BGL4 or BGL7)	Sucrose and Starch Metabolism	0	0	1000	0
GLCGSD	(PICST_28187 or PICST_30325 or PICST_33726 or PICST_74319)	PICST_28187 PICST_30325 PICST_33726 PICST_74319	(WSC3 or MUC1.11 or SGA1 or GDB1)	Sucrose and Starch Metabolism	0	0	1000	0

13BGH	(PICST_28554 or PICST_57025 or PICST_57399 or PICST_61452 or PICST_74722 or PICST_78873 or PICST_89695)	PICST_28554 PICST_57025 PICST_57399 PICST_61452 PICST_74722 PICST_78873 PICST_89695	(BOT2 or SCW4.2 or EXG1 or EXG3 or SCW4 or EXG2 or SCW4.1)	Sucrose and Starch Metabolism	0	0	1000	0
13BGHe	(PICST_28554 or PICST_57025 or PICST_57399 or PICST_61452 or PICST_74722 or PICST_78873 or PICST_89695)	PICST_28554 PICST_57025 PICST_57399 PICST_61452 PICST_74722 PICST_78873 PICST_89695	(BOT2 or SCW4.2 or EXG1 or EXG3 or SCW4 or EXG2 or SCW4.1)	Sucrose and Starch Metabolism	0	0	1000	0
SUCH	(PICST_29292 or PICST_42120 or PICST_56703 or PICST_85073)	PICST_29292 PICST_42120 PICST_56703 PICST_85073	(MAL9 or MAL6 or CGA1 or ROT2)	Sucrose and Starch Metabolism	0	0	1000	0
13GS	(PICST_38184 or PICST_43247 or (PICST_67249 and PICST_86938))	PICST_38184 PICST_43247 PICST_67249 PICST_86938	(FKS3 or SMI1.1 or (GSL2 and GSC2))	Sucrose and Starch Metabolism	0	0	1000	0
TRE6PS	(PICST_41330 and PICST_60441)	PICST_41330 PICST_60441	(TPS1 and TPS3)	Sucrose and Starch Metabolism	0	0	1000	0
TREH	(PICST_64968 or PICST_80169)	PICST_64968 PICST_80169	(NTH2 or ATH1)	Sucrose and Starch Metabolism	0	0	1000	0
GLYGP	PICST_72279	PICST_72279	GPH1	Sucrose and Starch Metabolism	0	0	1000	0
TRE6PP	PICST_79118	PICST_79118	TPS2	Sucrose and Starch Metabolism	0	0	1000	0
GLCS2	PICST_81231	PICST_81231	GSY1	Sucrose and Starch Metabolism	0	0	1000	0
GLYGS	PICST_81231	PICST_81231	GSY1	Sucrose and Starch Metabolism	0	0	1000	0
14GBEZ	PICST_88710	PICST_88710	GLC3	Sucrose and Starch Metabolism	0	0	1000	0
BPNT	PICST_47423	PICST_47423	MET22	Sulphur Metabolism	0	0	1000	0
PAPSR	PICST_49723	PICST_49723	HYP	Sulphur Metabolism	0	0	1000	0
SADT	PICST_75451	PICST_75451	MET3	Sulphur Metabolism	0	0	1000	0

SULRy	PICST_83457	PICST_83457	HYP	Sulphur Metabolism	1	-1000	1000	0
HMGCOAS	PICST_83020	PICST_83020	HYP-ERG	Butanoate Metabolism	1	-1000	1000	0
TAUDIO	(PICST_34605 or PICST_39554)	PICST_34605 PICST_39554	(TCD4 or IFH2)	Cysteine and Methionine Metabolism	0	0	1000	0
AMACALDDH				Cysteine and Methionine Metabolism	0	0	1000	0
GGTT	(PICST_29164 or PICST_41956)	PICST_29164 PICST_41956	(SRT1 or HYP)	Terpenoid Backbone Synthesis	0	0	1000	0
MEVK1	PICST_40742	PICST_40742	ERG12	Terpenoid Backbone Synthesis	0	0	1000	0
MEVK2	PICST_40742	PICST_40742	ERG12	Terpenoid Backbone Synthesis	0	0	1000	0
MEVK3	PICST_40742	PICST_40742	ERG12	Terpenoid Backbone Synthesis	0	0	1000	0
MEVK4	PICST_40742	PICST_40742	ERG12	Terpenoid Backbone Synthesis	0	0	1000	0
FRTT	(PICST_46658 or (PICST_32799 and PICST_55783))	PICST_46658 PICST_32799 PICST_55783	(BTS1or (BET4 and RAM2))	Terpenoid Backbone Synthesis	0	0	1000	0
DMATT	(PICST_46658 or PICST_89338)	PICST_46658 PICST_89338	(BTS1 or ERG20)	Terpenoid Backbone Synthesis	0	0	1000	0
GRTT	(PICST_46658 or PICST_89338)	PICST_46658 PICST_89338	(BTS1 or ERG20)	Terpenoid Backbone Synthesis	0	0	1000	0
PMEVK	PICST_52257	PICST_52257	ERG8	Terpenoid Backbone Synthesis	0	0	1000	0
IPDDI	PICST_68990	PICST_68990	IDI1	Terpenoid Backbone Synthesis	1	-1000	1000	0
HMGCOAR	PICST_72872	PICST_72872	HMG1	Terpenoid Backbone Synthesis	1	-1000	1000	0
DPMVD	PICST_90752	PICST_90752	HYP	Terpenoid Backbone Synthesis	0	0	1000	0
TMDPK	(PICST_46967 or PICST_59866)	PICST_46967 PICST_59866	(TNR3 or THI80)	Thiamine Metabolism	0	0	1000	0
TMDPPK	(PICST_46967 or PICST_59866)	PICST_46967 PICST_59866	(TNR3 or THI80)	Thiamine Metabolism	0	0	1000	0
THZPSN1	PICST_50942	PICST_50942	HYP	Thiamine Metabolism	0	0	1000	0
THZPSN2	PICST_50942	PICST_50942	HYP	Thiamine Metabolism	0	0	1000	0
HMPK1	PICST_70077	PICST_70077	THI20	Thiamine Metabolism	0	0	1000	0
PMPK	PICST_70077	PICST_70077	THI20	Thiamine Metabolism	0	0	1000	0
TMN	PICST_70077	PICST_70077	THI20	Thiamine Metabolism	0	0	1000	0
THMP	(PICST_74933 or PICST_82137)	PICST_74933 PICST_82137	(YUB4 or YMR1)	Thiamine Metabolism	0	0	1000	0

THMDP	(PICST_74933 or PICST_82137 or PICST_72696)	PICST_74933 PICST_82137 PICST_72696	(YUB4 or YMR1or HAM1)	Thiamine Metabolism	0	0	1000	0
HETZK	PICST_77822	PICST_77822	HYP	Thiamine Metabolism	0	0	1000	0
TMPPP	PICST_77822	PICST_77822	HYP	Thiamine Metabolism	0	0	1000	0
AHMMPS				Thiamine Metabolism	0	0	1000	0
TMPK				Thiamine Metabolism	1	-1000	1000	0
CITtam	PICST_29031	PICST_29031	CTP1	Transport Mitochondrial	1	-1000	1000	0
CITtcm	PICST_29031	PICST_29031	CTP1	Transport Mitochondrial	1	-1000	1000	0
CITtpm	PICST_29031	PICST_29031	CTP1	Transport Mitochondrial	1	-1000	1000	0
ATPtm-H	(PICST_45104 or PICST_85436)	PICST_45104 PICST_85436	(PET9 or ADT1)	Transport Mitochondrial	0	0	1000	0
SFC1	PICST_60344	PICST_60344	SFC1	Transport Mitochondrial	1	-1000	1000	0
ASPGLU2m	PICST_66704	PICST_66704	CMC1	Transport Mitochondrial	0	-1000	1000	0
DICtm	PICST_78946	PICST_78946	DIC1	Transport Mitochondrial	1	-1000	1000	0
MALtm	PICST_78946	PICST_78946	DIC1	Transport Mitochondrial	1	-1000	1000	0
SUCCtm	PICST_78946	PICST_78946	DIC1	Transport Mitochondrial	1	-1000	1000	0
CTPtm	PICST_83067	PICST_83067	HYP	Transport Mitochondrial	0	0	1000	0
2DHPtm				Transport Mitochondrial	1	-1000	1000	0
2OBUTtm				Transport Mitochondrial	1	-1000	1000	0
2OXOADPtm				Transport Mitochondrial	0	0	1000	0
34HPPt2m				Transport Mitochondrial	1	-1000	1000	0
3C3HMPtm				Transport Mitochondrial	1	-1000	1000	0
3C4MOPtm				Transport Mitochondrial	1	-1000	1000	0
3MOBtm				Transport Mitochondrial	0	0	1000	0
3MOPtm				Transport Mitochondrial	1	-1000	1000	0
4HPRO-LTtm				Transport Mitochondrial	1	-1000	1000	0
5AOPtm				Transport Mitochondrial	1	-1000	1000	0
ACACtm				Transport Mitochondrial	1	-1000	1000	0
ACALDtm				Transport Mitochondrial	1	-1000	1000	0
ACPtm				Transport Mitochondrial	1	-1000	1000	0
ACtm				Transport Mitochondrial	1	-1000	1000	0
AHCYStm				Transport Mitochondrial	1	-1000	1000	0
ALAtm				Transport Mitochondrial	0	-1000	1000	0

AMETtm	Transport Mitochondrial	1	-1000	1000	0
CO2tm	Transport Mitochondrial	1	-1000	1000	0
COAtm	Transport Mitochondrial	0	0	1000	0
DHAPtm	Transport Mitochondrial	0	0	1000	0
D-LACtm	Transport Mitochondrial	1	-1000	1000	0
E4HGLUtm	Transport Mitochondrial	1	-1000	1000	0
FA100ACPtm	Transport Mitochondrial	0	0	1000	0
FA120ACPtm	Transport Mitochondrial	0	0	1000	0
FA140ACPtm	Transport Mitochondrial	0	0	1000	0
FA160ACPtm	Transport Mitochondrial	0	0	1000	0
FA161ACPtm	Transport Mitochondrial	0	0	1000	0
FA170ACPtm	Transport Mitochondrial	0	0	1000	0
FA171ACPtm	Transport Mitochondrial	0	0	1000	0
FA180ACPtm	Transport Mitochondrial	0	0	1000	0
FA181ACPtm	Transport Mitochondrial	0	0	1000	0
FA182ACPtm	Transport Mitochondrial	0	0	1000	0
FA183ACPtm	Transport Mitochondrial	0	0	1000	0
FA200ACPtm	Transport Mitochondrial	0	0	1000	0
FA205ACPtm	Transport Mitochondrial	0	0	1000	0
FA220ACPtm	Transport Mitochondrial	0	0	1000	0
FA240ACPtm	Transport Mitochondrial	0	0	1000	0
FA260ACPtm	Transport Mitochondrial	0	0	1000	0
FA60ACPtm	Transport Mitochondrial	0	0	1000	0
FA80ACPtm	Transport Mitochondrial	0	0	1000	0
FE2utm	Transport Mitochondrial	0	0	1000	0
FEtm	Transport Mitochondrial	0	0	1000	0
FORtm	Transport Mitochondrial	0	0	1000	0
FRDPtm	Transport Mitochondrial	1	-1000	1000	0
GLYC3Ptm	Transport Mitochondrial	0	0	1000	0
GLYt2m	Transport Mitochondrial	1	-1000	1000	0
H2Otm	Transport Mitochondrial	1	-1000	1000	0
HIBUTtm	Transport Mitochondrial	1	-1000	1000	0
IBCOAtm	Transport Mitochondrial	1	-1000	1000	0
ILEtm	Transport Mitochondrial	0	0	1000	0

IPDPtm	Transport Mitochondrial	1	-1000	1000	0
IVCOAtm	Transport Mitochondrial	1	-1000	1000	0
L-LACtm	Transport Mitochondrial	1	-1000	1000	0
NH4tm	Transport Mitochondrial	1	-1000	1000	0
O2tm	Transport Mitochondrial	1	-1000	1000	0
OAAtm	Transport Mitochondrial	1	-1000	1000	0
ORNtm	Transport Mitochondrial	1	-1000	1000	0
PANTtm	Transport Mitochondrial	1	-1000	1000	0
Patm	Transport Mitochondrial	1	-1000	1000	0
PENDPtm	Transport Mitochondrial	1	-1000	1000	0
Petm	Transport Mitochondrial	1	-1000	1000	0
PPCOAM	Transport Mitochondrial	1	-1000	1000	0
PPPG9tm	Transport Mitochondrial	1	-1000	1000	0
PROtm	Transport Mitochondrial	1	-1000	1000	0
PStm	Transport Mitochondrial	1	-1000	1000	0
PYRtm	Transport Mitochondrial	1	-1000	1000	0
SERtm	Transport Mitochondrial	1	-1000	1000	0
THRt2m	Transport Mitochondrial	1	-1000	1000	0
TYRt2m	Transport Mitochondrial	1	-1000	1000	0
VALtm	Transport Mitochondrial	1	-1000	1000	0

GLNt	(PICST_28779 or PICST_89811 or PICST_32180 or PICST_68755 or PICST_11039 or PICST_78721 or PICST_37009 or PICST_41966 or PICST_67826 or PICST_39044 or PICST_44246 or PICST_51094 or PICST_82860 or PICST_52358 or PICST_28502)	PICST_28779 PICST_89811 PICST_32180 PICST_68755 PICST_11039 PICST_78721 PICST_37009 PICST_41966 PICST_67826 PICST_39044 PICST_44246 PICST_51094 PICST_82860 PICST_52358 PICST_28502	(GNP1 or GNP2 or GAP1.1 or GAP1.2 or GAP1.8 or GAP1.6 or GAP1.4 or GAP1.7 or GAP1.5 or DIP5.1 or DIP5.2 or DIP5.4 or DIP5.3 or AGP3 or AGP2)	Transport Reaction	1	-1000	1000	0
HISt2r	(PICST_29107 or PICST_32180 or PICST_68755 or PICST_11039 or PICST_78721 or PICST_37009 or PICST_41966 or PICST_67826 or PICST_52358 or PICST_28502)	PICST_29107 PICST_32180 PICST_68755 PICST_11039 PICST_78721 PICST_37009 PICST_41966 PICST_67826 PICST_52358 PICST_28502	(HIP1 or GAP1.1 or GAP1.2 or GAP1.8 or GAP1.6 or GAP1.4 or GAP1.7 or GAP1.5 or AGP3 or AGP2)	Transport Reaction	1	-1000	1000	0
THMt	PICST_29465	PICST_29465	THI7	Transport Reaction	0	0	1000	0

ALAt2	(PICST_32180 or<br>PICST_68755 or<br>PICST_11039 or<br>PICST_78721 or<br>PICST_37009 or<br>PICST_41966 or<br>PICST_67826 or<br>PICST_39044 or<br>PICST_44246 or<br>PICST_51094 or<br>PICST_82860 or<br>PICST_52358 or<br>PICST_28502)	PICST_32180<br>PICST_68755<br>PICST_11039<br>PICST_78721<br>PICST_37009<br>PICST_41966<br>PICST_67826<br>PICST_39044<br>PICST_44246<br>PICST_51094<br>PICST_82860<br>PICST_52358<br>PICST_28502	(GAP1.1 or<br>GAP1.2 or<br>GAP1.8 or<br>GAP1.6 or<br>GAP1.4 or<br>GAP1.7 or<br>GAP1.5 or<br>DIP5.1 or DIP5.2<br>or DIP5.4 or<br>DIP5.3 or AGP3<br>or AGP2)	Transport Reaction	1	-1000	1000	0
ASNt2	(PICST_32180 or<br>PICST_68755 or<br>PICST_11039 or<br>PICST_78721 or<br>PICST_37009 or<br>PICST_41966 or<br>PICST_67826 or<br>PICST_39044 or<br>PICST_44246 or<br>PICST_51094 or<br>PICST_82860 or<br>PICST_52358 or<br>PICST_28502)	PICST_32180<br>PICST_68755<br>PICST_11039<br>PICST_78721<br>PICST_37009<br>PICST_41966<br>PICST_67826<br>PICST_39044<br>PICST_44246<br>PICST_51094<br>PICST_82860<br>PICST_52358<br>PICST_28502	(GAP1.1 or<br>GAP1.2 or<br>GAP1.8 or<br>GAP1.6 or<br>GAP1.4 or<br>GAP1.7 or<br>GAP1.5 or<br>DIP5.1 or DIP5.2<br>or DIP5.4 or<br>DIP5.3 or AGP3<br>or AGP2)	Transport Reaction	1	-1000	1000	0

ASPt2	(PICST_32180 or PICST_68755 or PICST_11039 or PICST_78721 or PICST_37009 or PICST_41966 or PICST_67826 or PICST_39044 or PICST_44246 or PICST_51094 or PICST_82860 or PICST_52358 or PICST_28502)	PICST_32180 PICST_68755 PICST_11039 PICST_78721 PICST_37009 PICST_41966 PICST_67826 PICST_39044 PICST_44246 PICST_51094 PICST_82860 PICST_52358 PICST_28502	(GAP1.1 or GAP1.2 or GAP1.8 or GAP1.6 or GAP1.4 or GAP1.7 or GAP1.5 or DIP5.1 or DIP5.2 or DIP5.4 or DIP5.3 or AGP3 or AGP2)	Transport Reaction	1	-1000	1000	0
GLUt	(PICST_32180 or PICST_68755 or PICST_11039 or PICST_78721 or PICST_37009 or PICST_41966 or PICST_67826 or PICST_39044 or PICST_44246 or PICST_51094 or PICST_82860 or PICST_52358 or PICST_28502)	PICST_32180 PICST_68755 PICST_11039 PICST_78721 PICST_37009 PICST_41966 PICST_67826 PICST_39044 PICST_44246 PICST_51094 PICST_82860 PICST_52358 PICST_28502	(GAP1.1 or GAP1.2 or GAP1.8 or GAP1.6 or GAP1.4 or GAP1.7 or GAP1.5 or DIP5.1 or DIP5.2 or DIP5.4 or DIP5.3 or AGP3 or AGP2)	Transport Reaction	1	-1000	1000	0

GLYt	(PICST_32180 or PICST_68755 or PICST_11039 or PICST_78721 or PICST_37009 or PICST_41966 or PICST_67826 or PICST_39044 or PICST_44246 or PICST_51094 or PICST_82860 or PICST_52358 or PICST_28502)	PICST_32180 PICST_68755 PICST_11039 PICST_78721 PICST_37009 PICST_41966 PICST_67826 PICST_39044 PICST_44246 PICST_51094 PICST_82860 PICST_52358 PICST_28502	(GAP1.1 or GAP1.2 or GAP1.8 or GAP1.6 or GAP1.4 or GAP1.7 or GAP1.5 or DIP5.1 or DIP5.2 or DIP5.4 or DIP5.3 or AGP3 or AGP2)	Transport Reaction	1	-1000	1000	0
SERt	(PICST_32180 or PICST_68755 or PICST_11039 or PICST_78721 or PICST_37009 or PICST_41966 or PICST_67826 or PICST_39044 or PICST_44246 or PICST_51094 or PICST_82860 or PICST_52358 or PICST_28502)	PICST_32180 PICST_68755 PICST_11039 PICST_78721 PICST_37009 PICST_41966 PICST_67826 PICST_39044 PICST_44246 PICST_51094 PICST_82860 PICST_52358 PICST_28502	(GAP1.1 or GAP1.2 or GAP1.8 or GAP1.6 or GAP1.4 or GAP1.7 or GAP1.5 or DIP5.1 or DIP5.2 or DIP5.4 or DIP5.3 or or AGP3 or AGP2)	Transport Reaction	1	-1000	1000	0
CYSt	(PICST_32180 or PICST_68755 or PICST_11039 or PICST_78721 or PICST_37009 or PICST_41966 or PICST_67826)	PICST_32180 PICST_68755 PICST_11039 PICST_78721 PICST_37009 PICST_41966 PICST_67826	(GAP1.1 or GAP1.2 or GAP1.8 or GAP1.6 or GAP1.4 or GAP1.7 or GAP1.5)	Transport Reaction	1	-1000	1000	0

ILEt2r	(PICST_32180 or PICST_68755 or PICST_11039 or PICST_78721 or PICST_37009 or PICST_41966 or PICST_67826 or PICST_52358 or PICST_28502)	PICST_32180 PICST_68755 PICST_11039 PICST_78721 PICST_37009 PICST_41966 PICST_67826 PICST_52358 PICST_28502	(GAP1.1 or GAP1.2 or GAP1.8 or GAP1.6 or GAP1.4 or GAP1.7 or GAP1.5 or AGP3 or AGP2)	Transport Reaction	1	-1000	1000	0
LEUt2r	(PICST_32180 or PICST_68755 or PICST_11039 or PICST_78721 or PICST_37009 or PICST_41966 or PICST_67826 or PICST_52358 or PICST_28502)	PICST_32180 PICST_68755 PICST_11039 PICST_78721 PICST_37009 PICST_41966 PICST_67826 PICST_52358 PICST_28502	(GAP1.1 or GAP1.2 or GAP1.8 or GAP1.6 or GAP1.4 or GAP1.7 or GAP1.5 or AGP3 or AGP2)	Transport Reaction	1	-1000	1000	0
ORNt	(PICST_32180 or PICST_68755 or PICST_11039 or PICST_78721 or PICST_37009 or PICST_41966 or PICST_67826 or PICST_52358 or PICST_28502)	PICST_32180 PICST_68755 PICST_11039 PICST_78721 PICST_37009 PICST_41966 PICST_67826 PICST_52358 PICST_28502	(GAP1.1 or GAP1.2 or GAP1.8 or GAP1.6 or GAP1.4 or GAP1.7 or GAP1.5 or or AGP3 or AGP2)	Transport Reaction	1	-1000	1000	0

PHEt2r	(PICST_32180 or<br>PICST_68755 or<br>PICST_11039 or<br>PICST_78721 or<br>PICST_37009 or<br>PICST_41966 or<br>PICST_67826 or<br>PICST_52358 or<br>PICST_28502)	PICST_32180<br>PICST_68755<br>PICST_11039<br>PICST_78721<br>PICST_37009<br>PICST_41966<br>PICST_67826<br>PICST_52358<br>PICST_28502	(GAP1.1 or<br>GAP1.2 or<br>GAP1.8 or<br>GAP1.6 or<br>GAP1.4 or<br>GAP1.7 or<br>GAP1.5 or or<br>AGP3 or AGP2)	Transport Reaction	1	-1000	1000	0
THRt2r	(PICST_32180 or<br>PICST_68755 or<br>PICST_11039 or<br>PICST_78721 or<br>PICST_37009 or<br>PICST_41966 or<br>PICST_67826)	PICST_32180<br>PICST_68755<br>PICST_11039<br>PICST_78721<br>PICST_37009<br>PICST_41966<br>PICST_67826	(GAP1.1 or<br>GAP1.2 or<br>GAP1.8 or<br>GAP1.6 or<br>GAP1.4 or<br>GAP1.7 or<br>GAP1.5 or AGP3<br>or AGP2)	Transport Reaction	1	-1000	1000	0
TRPt	(PICST_32180 or<br>PICST_68755 or<br>PICST_11039 or<br>PICST_78721 or<br>PICST_37009 or<br>PICST_41966 or<br>PICST_67826 or<br>PICST_52358 or<br>PICST_28502)	PICST_32180<br>PICST_68755<br>PICST_11039<br>PICST_78721<br>PICST_37009<br>PICST_41966<br>PICST_67826<br>PICST_52358<br>PICST_28502	(GAP1.1 or<br>GAP1.2 or<br>GAP1.8 or<br>GAP1.6 or<br>GAP1.4 or<br>GAP1.7 or<br>GAP1.5 or or<br>AGP3 or AGP2)	Transport Reaction	1	-1000	1000	0

TYRt2r	(PICST_32180 or PICST_68755 or PICST_11039 or PICST_78721 or PICST_37009 or PICST_41966 or PICST_67826 or PICST_52358 or PICST_28502)	PICST_32180 PICST_68755 PICST_11039 PICST_78721 PICST_37009 PICST_41966 PICST_67826 PICST_52358 PICST_28502	(GAP1.1 or GAP1.2 or GAP1.8 or GAP1.6 or GAP1.4 or GAP1.7 or GAP1.5 or AGP3 or AGP2)	Transport Reaction	1	-1000	1000	0
VALtx	(PICST_32180 or PICST_68755 or PICST_11039 or PICST_78721 or PICST_37009 or PICST_41966 or PICST_67826 or PICST_52358 or PICST_28502)	PICST_32180 PICST_68755 PICST_11039 PICST_78721 PICST_37009 PICST_41966 PICST_67826 PICST_52358 PICST_28502	(GAP1.1 or GAP1.2 or GAP1.8 or GAP1.6 or GAP1.4 or GAP1.7 or GAP1.5 or AGP3 or AGP2)	Transport Reaction	1	-1000	1000	0
ALANt	(PICST_33930 or PICST_62778 or PICST_34542 or PICST_34027 or PICST_29740 or PICST_65827 or PICST_31639 or PICST_31034 or PICST_48474 or PICST_30685 or PICST_42384 or PICST_72069 or PICST_33169 or PICST_46242 or PICST_33997)	PICST_33930 PICST_62778 PICST_34542 PICST_34027 PICST_29740 PICST_65827 PICST_31639 PICST_31034 PICST_48474 PICST_30685 PICST_42384 PICST_72069 PICST_33169 PICST_46242 PICST_33997	(DAL12 or DAL1 or DAL6 or DAL5 or DAL4 or ALT1 or DAL8 or DAL10 or DAL9 or DAL4.1 or DAL4.3 or DAL7 or DAL4.2 or ALT2 or DAL11)	Transport Reaction	1	-1000	1000	0
URATEt	PICST_34046	PICST_34046	UAP3	Transport Reaction	1	-1000	1000	0

XANt	PICST_34046	PICST_34046	UAP3	Transport Reaction	1	-1000	1000	0
PNTOt	PICST_35835	PICST_35835	FEN3	Transport Reaction	1	-1000	1000	0
Ala-Aspt	(PICST_36233 or PICST_66905 or PICST_70645)	PICST_36233 PICST_66905 PICST_70645	(PTR2 or HYP or HYP)	Transport Reaction	0	0	1000	0
Ala-Glnt	(PICST_36233 or PICST_66905 or PICST_70645)	PICST_36233 PICST_66905 PICST_70645	(PTR2 or HYP or HYP)	Transport Reaction	0	0	1000	0
Ala-Glut.	(PICST_36233 or PICST_66905 or PICST_70645)	PICST_36233 PICST_66905 PICST_70645	(PTR2 or HYP or HYP)	Transport Reaction	0	0	1000	0
Ala-Glyt.	(PICST_36233 or PICST_66905 or PICST_70645)	PICST_36233 PICST_66905 PICST_70645	(PTR2 or HYP or HYP)	Transport Reaction	0	0	1000	0
Ala-Hist.	(PICST_36233 or PICST_66905 or PICST_70645)	PICST_36233 PICST_66905 PICST_70645	(PTR2 or HYP or HYP)	Transport Reaction	0	0	1000	0
Ala-Leut	(PICST_36233 or PICST_66905 or PICST_70645)	PICST_36233 PICST_66905 PICST_70645	(PTR2 or HYP or HYP)	Transport Reaction	0	0	1000	0
Ala-Thrt	(PICST_36233 or PICST_66905 or PICST_70645)	PICST_36233 PICST_66905 PICST_70645	(PTR2 or HYP or HYP)	Transport Reaction	0	0	1000	0
CGLYt	(PICST_36233 or PICST_66905 or PICST_70645)	PICST_36233 PICST_66905 PICST_70645	(PTR2 or HYP or HYP)	Transport Reaction	1	0	1000	0
Glu-alat	(PICST_36233 or PICST_66905 or PICST_70645)	PICST_36233 PICST_66905 PICST_70645	(PTR2 or HYP or HYP)	Transport Reaction	1	0	1000	0
Gly-Asnt	(PICST_36233 or PICST_66905 or PICST_70645)	PICST_36233 PICST_66905 PICST_70645	(PTR2 or HYP or HYP)	Transport Reaction	0	0	1000	0

Gly-Aspt	(PICST_36233 or PICST_66905 or PICST_70645)	PICST_36233 PICST_66905 PICST_70645	(PTR2 or HYP or HYP)	Transport Reaction	0	0	1000	0
Gly-Glnt	(PICST_36233 or PICST_66905 or PICST_70645)	PICST_36233 PICST_66905 PICST_70645	(PTR2 or HYP or HYP)	Transport Reaction	0	0	1000	0
Gly-Glut	(PICST_36233 or PICST_66905 or PICST_70645)	PICST_36233 PICST_66905 PICST_70645	(PTR2 or HYP or HYP)	Transport Reaction	0	0	1000	0
Gly-Mett	(PICST_36233 or PICST_66905 or PICST_70645)	PICST_36233 PICST_66905 PICST_70645	(PTR2 or HYP or HYP)	Transport Reaction	0	0	1000	0
Gly-Prot	(PICST_36233 or PICST_66905 or PICST_70645)	PICST_36233 PICST_66905 PICST_70645	(PTR2 or HYP or HYP)	Transport Reaction	0	0	1000	0
Met-Alat	(PICST_36233 or PICST_66905 or PICST_70645)	PICST_36233 PICST_66905 PICST_70645	(PTR2 or HYP or HYP)	Transport Reaction	0	0	1000	0

GLCt1	(PICST_36948 or PICST_27983 or PICST_66414 or PICST_64741 or PICST_55800 or PICST_91253 or PICST_85501 or PICST_11314 or PICST_54022 or PICST_65135 or PICST_29041 or PICST_80517 or PICST_35662 or PICST_58946 or PICST_59900 or PICST_45095 or PICST_31239)	PICST_36948<br>PICST_27983<br>PICST_66414<br>PICST_64741<br>PICST_55800<br>PICST_91253<br>PICST_85501<br>PICST_11314<br>PICST_54022<br>PICST_65135<br>PICST_29041<br>PICST_80517<br>PICST_35662<br>PICST_58946<br>PICST_59900<br>PICST_45095<br>PICST_31239	(QUP2 or SUT2 or SUT1 or SUT4 or HGT1 or RGT2 or SNF3 or HXT2.5 or HXT2.3 or YBR2 or HXT2.1 or HXT2.4 or MFS5 or HXT2.6 or HYP or HYP or HYP)	Transport Reaction	0	0	1000	0
LACTOSEt2	(PICST_38981 or PICST_29297 or PICST_57768 or PICST_30035)	PICST_38981<br>PICST_29297<br>PICST_57768<br>PICST_30035	(HXT2.2 or LAC2 or LAC3 or LAC1)	Transport Reaction	0	0	1000	0
ABUTt	(PICST_39042 or PICST_64090)	PICST_39042<br>PICST_64090	(UGA4 or HYP)	Transport Reaction	1	-1000	1000	0
XYLt	(PICST_39517 or PICST_16856 or PICST_33078)	PICST_39517<br>PICST_16856<br>PICST_33078	(XUT7 or XUT4 or XUT5)	Transport Reaction	1	-1000	1000	0
MALTt2	(PICST_48369 or PICST_47584 or PICST_29639 or PICST_31838 or PICST_32531)	PICST_48369<br>PICST_47584<br>PICST_29639<br>PICST_31838<br>PICST_32531	(MAL4 or MAL3 or MAL1 or MAL2 or MAL5)	Transport Reaction	0	0	1000	0
FRUt	PICST_48420	PICST_48420	HXT4	Transport Reaction	0	0	1000	0
ADEt	PICST_54260	PICST_54260	ANT1	Transport Reaction	0	0	1000	0

PROt2r	(PICST_57189 or<br>PICST_52197or<br>PICST_32180 or<br>PICST_68755 or<br>PICST_11039 or<br>PICST_78721 or<br>PICST_37009 or<br>PICST_41966 or<br>PICST_67826 or<br>PICST_52358 or<br>PICST_28502)	PICST_57189<br>PICST_52197<br>PICST_32180<br>PICST_68755<br>PICST_11039<br>PICST_78721<br>PICST_37009<br>PICST_41966<br>PICST_67826<br>PICST_52358<br>PICST_28502	(PUT4.1 or<br>DIP5.5 or<br>GAP1.1 or<br>GAP1.2 or<br>GAP1.8 or<br>GAP1.6 or<br>GAP1.4 or<br>GAP1.7 or<br>GAP1.5 or or<br>AGP3 or AGP2)	Transport Reaction	1	-1000	1000	0
UREA2t	(PICST_57505 or<br>PICST_29213 or<br>PICST_32520 or<br>PICST_60304 or<br>PICST_55023 or<br>PICST_52351 or<br>PICST_49064)	PICST_57505<br>PICST_29213<br>PICST_32520<br>PICST_60304<br>PICST_55023<br>PICST_52351<br>PICST_49064	(DUR2.3 or<br>DUR5.2 or<br>DUR3.2 or DUR8<br>or DUR4 or<br>DUR3.1 or<br>DUR5.1)	Transport Reaction	1	-1000	1000	0
GALt2	PICST_58479	PICST_58479	FUC1	Transport Reaction	0	0	1000	0
CYTDt	PICST_59290	PICST_59290	HYP	Transport Reaction	0	0	1000	0
GUAt2r	PICST_60316	PICST_60316	YHM1	Transport Reaction	0	0	1000	0
NACt	(PICST_61879 or<br>PICST_43167 or<br>PICST_57264 or<br>PICST_60854)	PICST_61879<br>PICST_43167<br>PICST_57264<br>PICST_60854	(TNA13 or TNA1<br>or TNA11 or<br>TNA12)	Transport Reaction	1	-1000	1000	0
PIt2r	(PICST_66490 or<br>PICST_86239 or<br>PICST_81069 or<br>PICST_83361 or<br>PICST_82453)	PICST_66490<br>PICST_86239<br>PICST_81069<br>PICST_83361<br>PICST_82453	(PHO89 or<br>PHO87 or<br>PHO81 or<br>PHO91 or<br>PHO84)	Transport Reaction	1	-1000	1000	0
GLYCt1	PICST_66725	PICST_66725	GUP1	Transport Reaction	0	0	0	0

METt	(PICST_69488 or PICST_28954 or PICST_45036 or PICST_32180 or PICST_68755 or PICST_11039 or PICST_78721 or PICST_37009 or PICST_41966 or PICST_67826 or PICST_52358 or PICST_28502)	PICST_69488 PICST_28954 PICST_45036 PICST_32180 PICST_68755 PICST_11039 PICST_78721 PICST_37009 PICST_41966 PICST_67826 PICST_52358 PICST_28502	(MUP1.1 or MUP3 or MUP1.2 or GAP1.1 or GAP1.2 or GAP1.8 or GAP1.6 or GAP1.4 or GAP1.7 or GAP1.5 or AGP3 or AGP2)	Transport Reaction	1	-1000	1000	0
CSNt2	(PICST_75870 or PICST_72073)	PICST_75870 PICST_72073	(HYP or HYP)	Transport Reaction	0	0	1000	0
INSTt	PICST_80454	PICST_80454	ITR2	Transport Reaction	0	0	1000	0
LYSt2r	(PICST_81292 or PICST_51006 or PICST_32180 or PICST_68755 or PICST_11039 or PICST_78721 or PICST_37009 or PICST_41966 or PICST_67826 or PICST_52358 or PICST_28502)	PICST_81292 PICST_51006 PICST_32180 PICST_68755 PICST_11039 PICST_78721 PICST_37009 PICST_41966 PICST_67826 PICST_52358 PICST_28502	(ALP1 or LYP1 or GAP1.1 or GAP1.2 or GAP1.8 or GAP1.6 or GAP1.4 or GAP1.7 or GAP1.5 or AGP3 or AGP2)	Transport Reaction	1	-1000	1000	0
NH4t	(PICST_83334 or PICST_88919 or PICST_47780)	PICST_83334 PICST_88919 PICST_47780	(MEP1 or MEP2 or MEP3)	Transport Reaction	1	-1000	1000	0

ARGt2	(PICST_84246 or PICST_30939 or PICST_32180 or PICST_68755 or PICST_11039 or PICST_78721 or PICST_37009 or PICST_41966 or PICST_67826 or PICST_39044 or PICST_44246 or PICST_51094 or PICST_82860 or PICST_52358 or PICST_28502)	PICST_84246 PICST_30939 PICST_32180 PICST_68755 PICST_11039 PICST_78721 PICST_37009 PICST_41966 PICST_67826 PICST_39044 PICST_44246 PICST_51094 PICST_82860 PICST_52358 PICST_28502	(CAN2 or CAN1 or GAP1.1 or GAP1.2 or GAP1.8 or GAP1.6 or GAP1.4 or GAP1.7 or GAP1.5 or DIP5.1 or DIP5.2 or DIP5.4 or DIP5.3 or AGP3 or AGP2)	Transport Reaction	1	-1000	1000	0
SO4t	(PICST_85698 or PICST_51416 or PICST_32535 or PICST_80830)	PICST_85698 PICST_51416 PICST_32535 PICST_80830	(SUL1 or SUL3 or SUL4 or SUL2)	Transport Reaction	0	0	1000	0
ARABDt	PICST_87108	PICST_87108	AUT1	Transport Reaction	1	-1000	1000	0
ARABLt	PICST_87108	PICST_87108	AUT1	Transport Reaction	1	-1000	1000	0
2MBALDt				Transport Reaction	1	-1000	1000	0
2MBTOHt				Transport Reaction	1	-1000	1000	0
2MPPALt				Transport Reaction	1	-1000	1000	0
2OBUTt				Transport Reaction	1	-1000	1000	0
2PGLYCt				Transport Reaction	1	-1000	1000	0
2PGt				Transport Reaction	0	0	1000	0
2PHETOHt				Transport Reaction	1	-1000	1000	0
3C3HMPt				Transport Reaction	1	-1000	1000	0
3MBALDt				Transport Reaction	1	-1000	1000	0
3MOPt				Transport Reaction	1	-1000	1000	0
3PGt				Transport Reaction	0	0	1000	0
4ABZt				Transport Reaction	1	-1000	1000	0
4HPRO-LTt				Transport Reaction	1	-1000	1000	0

5AOPt	Transport Reaction	0	0	1000	0
6PGCt	Transport Reaction	1	-1000	1000	0
8AONNt2	Transport Reaction	1	-1000	1000	0
ACACt	Transport Reaction	1	-1000	1000	0
ACALDt	Transport Reaction	1	-1000	1000	0
ACETMt	Transport Reaction	1	-1000	1000	0
ACGAMT	Transport Reaction	1	-1000	1000	0
ACtr	Transport Reaction	1	-1000	1000	0
ADNt	Transport Reaction	0	0	1000	0
AGMt	Transport Reaction	1	-1000	1000	0
AKGt	Transport Reaction	1	-1000	1000	0
ALADt	Transport Reaction	1	-1000	1000	0
AMETt	Transport Reaction	0	0	1000	0
AMPt6	Transport Reaction	1	-1000	1000	0
ARABITOLDt	Transport Reaction	1	-1000	1000	0
ARABITOLLt	Transport Reaction	1	-1000	1000	0
BTD-RRt	Transport Reaction	1	-1000	1000	0
BTNt2	Transport Reaction	0	0	1000	0
CH4St	Transport Reaction	1	-1000	1000	0
CHLt2	Transport Reaction	1	-1000	1000	0
CITRULt	Transport Reaction	1	-1000	1000	0
CITt2	Transport Reaction	1	-1000	1000	0
CMPt	Transport Reaction	1	-1000	1000	0
CO2t	Transport Reaction	1	-1000	1000	0
CYSTt	Transport Reaction	1	-1000	1000	0
DADNt2	Transport Reaction	0	0	1000	0
DANNt	Transport Reaction	1	-1000	1000	0
DCAt	Transport Reaction	1	-1000	1000	0
DCYTt	Transport Reaction	0	0	1000	0
DDCAt	Transport Reaction	1	-1000	1000	0
DGSNt2	Transport Reaction	0	0	1000	0
DHAT	Transport Reaction	1	-1000	1000	0
DINSt	Transport Reaction	0	0	1000	0
D-LACt2	Transport Reaction	1	-1000	1000	0

DOCOSAt	Transport Reaction	1	-1000	1000	0
DTMPt	Transport Reaction	1	-1000	1000	0
DTTPt	Transport Reaction	1	-1000	1000	0
DURIt2	Transport Reaction	0	0	1000	0
EICOSAPENt	Transport Reaction	1	-1000	1000	0
EPISTt	Transport Reaction	1	-1000	1000	0
ERGSTt	Transport Reaction	1	-1000	1000	0
ETHAt	Transport Reaction	1	-1000	1000	0
ETOHt	Transport Reaction	1	-1000	1000	0
EX_lact(e)	Transport Reaction	1	0	1000	0
F6Pt6_2	Transport Reaction	0	0	1000	0
FE2t	Transport Reaction	0	0	1000	0
FECOSTt	Transport Reaction	1	-1000	1000	0
FORt	Transport Reaction	1	-1000	1000	0
FRMDt	Transport Reaction	1	-1000	1000	0
FUMt2r	Transport Reaction	0	0	1000	0
G1Pt6_2	Transport Reaction	0	0	1000	0
G3PCt	Transport Reaction	1	-1000	1000	0
G3PIt	Transport Reaction	1	-1000	1000	0
G6Pt6_2	Transport Reaction	0	0	1000	0
GAM6Pt	Transport Reaction	0	0	1000	0
GCALDt	Transport Reaction	1	-1000	1000	0
GLCNt	Transport Reaction	1	-1000	1000	0
GLXt	Transport Reaction	1	-1000	1000	0
GLY3Pt	Transport Reaction	0	0	1000	0
GLYCLTt	Transport Reaction	1	-1000	1000	0
GLYCt	Transport Reaction	1	-1000	1000	0
GMPt	Transport Reaction	1	-1000	1000	0
GSNt	Transport Reaction	0	0	1000	0
GTHOXt	Transport Reaction	0	0	1000	0
GTHRDt	Transport Reaction	0	0	1000	0
H2Ot	Transport Reaction	1	-1000	1000	0
HCO3E	Transport Reaction	1	-1000	1000	0
HCO3Em	Transport Reaction	1	-1000	1000	0

HDCAt	Transport Reaction	1	-1000	1000	0
HDCEAt	Transport Reaction	1	-1000	1000	0
HEPDCEAt	Transport Reaction	1	-1000	1000	0
HEXAt	Transport Reaction	1	-1000	1000	0
HEXCOAt	Transport Reaction	1	-1000	1000	0
HEXct	Transport Reaction	1	-1000	1000	0
HOMt	Transport Reaction	1	-1000	1000	0
IAMOHt	Transport Reaction	1	-1000	1000	0
IBUTOHt	Transport Reaction	1	-1000	1000	0
IND3ETHt	Transport Reaction	1	-1000	1000	0
INSt2	Transport Reaction	0	0	1000	0
Kt2r	Transport Reaction	0	0	1000	0
LANOSTt	Transport Reaction	1	-1000	1000	0
LCYSt	Transport Reaction	0	0	1000	0
L-LACt2r	Transport Reaction	1	-1000	1000	0
LRHAMt	Transport Reaction	1	-1000	1000	0
MALt	Transport Reaction	1	-1000	1000	0
MALTRt	Transport Reaction	1	-1000	1000	0
MAN1Pt	Transport Reaction	0	0	1000	0
MAN6Pt	Transport Reaction	0	0	1000	0
MANNt	Transport Reaction	1	-1000	1000	0
MANt2	Transport Reaction	0	0	1000	0
NADPt	Transport Reaction	1	-1000	1000	0
NMNTP	Transport Reaction	0	0	1000	0
O2t	Transport Reaction	1	-1000	1000	0
OAAt	Transport Reaction	1	-1000	1000	0
OCDCAt	Transport Reaction	1	-1000	1000	0
OCDCEAt	Transport Reaction	1	-1000	1000	0
OCDCTAt	Transport Reaction	1	-1000	1000	0
OCDCYAt	Transport Reaction	1	-1000	1000	0
OCTAt	Transport Reaction	1	-1000	1000	0
OPROt	Transport Reaction	1	-1000	1000	0
PACALDt	Transport Reaction	1	-1000	1000	0
PAPt	Transport Reaction	1	-1000	1000	0

PEPt				Transport Reaction	0	0	1000	0
PPIabc				Transport Reaction	0	0	1000	0
PSER_Lt				Transport Reaction	1	-1000	1000	0
PTRCt				Transport Reaction	0	0	1000	0
PYRt				Transport Reaction	0	0	1000	0
RIBFLVt2				Transport Reaction	0	0	1000	0
Ribitolt				Transport Reaction	1	-1000	1000	0
RIBt				Transport Reaction	0	0	1000	0
SBT_Dt				Transport Reaction	1	-1000	1000	0
SBT_Lt				Transport Reaction	1	-1000	1000	0
SO3t				Transport Reaction	0	0	1000	0
SPMDt				Transport Reaction	0	0	1000	0
SPRMt				Transport Reaction	0	0	1000	0
SRB_Lt				Transport Reaction	1	-1000	1000	0
SUCCt				Transport Reaction	1	-1000	1000	0
SUCRt2				Transport Reaction	0	0	1000	0
TAURt				Transport Reaction	1	-1000	1000	0
THMDPt				Transport Reaction	0	0	1000	0
THMDt2				Transport Reaction	1	-1000	1000	0
THMPt				Transport Reaction	0	0	1000	0
THYMt				Transport Reaction	1	-1000	1000	0
TMPt				Transport Reaction	1	-1000	1000	0
TREt				Transport Reaction	0	0	1000	0
TTDCAt				Transport Reaction	1	-1000	1000	0
UMPt				Transport Reaction	1	-1000	1000	0
URAt				Transport Reaction	1	-1000	1000	0
URIt				Transport Reaction	0	0	1000	0
XTSNt2				Transport Reaction	0	0	1000	0
XYLTt				Transport Reaction	1	-1000	1000	0
ZYMSTt				Transport Reaction	1	-1000	1000	0
CAT	PICST_40324	PICST_40324	CAT1	Tryptophan Metabolism	0	0	1000	0
KYN3OX	PICST_40545	PICST_40545	BNA4	Tryptophan Metabolism	0	0	1000	0

AMID3	(PICST_43525 or PICST_47832 or PICST_48268 or PICST_87495)	PICST_43525 PICST_47832 PICST_48268 PICST_87495	(AMI1 or AMD2 or AMD4 or GTA2)	Arginine and Proline Metabolism	0	0	1000	0
FKYNH	PICST_45065	PICST_45065	HYP	Tryptophan Metabolism	0	0	1000	0
HKYNH	PICST_55542	PICST_55542	BNA5	Tryptophan Metabolism	0	0	1000	0
KYN	PICST_55542	PICST_55542	BNA5	Tryptophan Metabolism	0	0	1000	0
3HAO	PICST_76205	PICST_76205	BNA1	Tryptophan Metabolism	0	0	1000	0
TRPO2	PICST_87443	PICST_87443	BNA2	Tryptophan Metabolism	0	0	1000	0
AM6SAD				Tryptophan Metabolism	0	0	1000	0
PCLAD				Tryptophan Metabolism	0	0	1000	0
QUILSYN				Tryptophan Metabolism	0	0	1000	0
NTRLASE1	PICST_36697	PICST_36697	NIT1	Nitrogen Metabolism	0	0	1000	0
FALDH2	PICST_29252	PICST_29252	FDH1	Tyrosine Metabolism	1	-1000	1000	0
34HPPOR	PICST_46345	PICST_46345	HPD1	Tyrosine Metabolism	0	0	1000	0
FUMAC				Tyrosine Metabolism	0	0	1000	0
HGNTOR				Tyrosine Metabolism	0	0	1000	0
MACACI				Tyrosine Metabolism	0	0	1000	0
SPMDAT1				Tyrosine Metabolism	0	0	1000	0
spmdAT1				Purine Metabolism	0	0	1000	0
ACHBSm	(PICST_44973 and PICST_80460)	PICST_44973 PICST_80460	(ILV2 and ILV6)	Valine, leucine and isoleucine Biosynthesis	0	0	1000	0
IPMD	PICST_68561	PICST_68561	LEU2	Valine, leucine and isoleucine Biosynthesis	0	0	1000	0
IPPMIa	PICST_68659	PICST_68659	LEU1	Valine, leucine and isoleucine Biosynthesis	1	-1000	1000	0
IPPMIb	PICST_68659	PICST_68659	LEU1	Valine, leucine and isoleucine Biosynthesis	1	-1000	1000	0
DHAD2m	PICST_75802	PICST_75802	ILV3	Valine, leucine and isoleucine Biosynthesis	0	0	1000	0
KARA2m	PICST_78299	PICST_78299	ILV5	Valine, leucine and isoleucine Biosynthesis	0	0	1000	0
THRDm	PICST_83915	PICST_83915	ILV1	Valine, leucine and isoleucine Biosynthesis	0	0	1000	0

ALCDHTP1	(PICST_27980 or PICST_68558)	PICST_27980 PICST_68558	(ADH1 or ADH2)	Valine, leucine and isoleucine Metabolism	0	0	1000	0
ALCDH2MB1	(PICST_27980 or PICST_68558 or PICST_59150 or PICST_67803)	PICST_27980 PICST_68558 PICST_59150 PICST_67803	(ADH1 or ADH2 or SAD1 or SAD2)	Valine, leucine and isoleucine Metabolism	0	0	1000	0
ALCDHIA1	(PICST_27980 or PICST_68558 or PICST_59150 or PICST_67803)	PICST_27980 PICST_68558 PICST_59150 PICST_67803	(ADH1 or ADH2 or SAD1 or SAD2)	Valine, leucine and isoleucine Metabolism	0	0	1000	0
ALCDHIB1	(PICST_27980 or PICST_68558 or PICST_59150 or PICST_67803)	PICST_27980 PICST_68558 PICST_59150 PICST_67803	(ADH1 or ADH2 or SAD1 or SAD2)	Valine, leucine and isoleucine Metabolism	0	0	1000	0
ALCDHPE1	(PICST_27980 or PICST_68558 or PICST_59150 or PICST_67803)	PICST_27980 PICST_68558 PICST_59150 PICST_67803	(ADH1 or ADH2 or SAD1 or SAD2)	Valine, leucine and isoleucine Metabolism	0	0	1000	0
URAMID	PICST_28452	PICST_28452	DUR1	Valine, leucine and isoleucine Metabolism	1	-1000	1000	0
ALCDH2MB2	(PICST_29079 or PICST_45137)	PICST_29079 PICST_45137	(ADH4 or ADH7)	Valine, leucine and isoleucine Metabolism	0	0	1000	0
ALCDHIA2	(PICST_29079 or PICST_45137)	PICST_29079 PICST_45137	(ADH4 or ADH7)	Valine, leucine and isoleucine Metabolism	0	0	1000	0
ALCDHIB2	(PICST_29079 or PICST_45137)	PICST_29079 PICST_45137	(ADH4 or ADH7)	Valine, leucine and isoleucine Metabolism	0	0	1000	0
ALCDHPE2	(PICST_29079 or PICST_45137)	PICST_29079 PICST_45137	(ADH4 or ADH7)	Valine, leucine and isoleucine Metabolism	0	0	1000	0
ALCDHTP2	(PICST_29079 or PICST_45137)	PICST_29079 PICST_45137	(ADH4 or ADH7)	Valine, leucine and isoleucine Metabolism	0	0	1000	0
HBUTHYD	(PICST_30193 or PICST_32846)	PICST_30193 PICST_32846	(ECH1 or EHO3)	Valine, leucine and isoleucine Metabolism	1	-1000	1000	0
MACRYLCOAH	(PICST_30193 or PICST_32846)	PICST_30193 PICST_32846	(ECH1 or EHO3)	Valine, leucine and isoleucine Metabolism	1	-1000	1000	0

MBUT2COAH	(PICST_30193 or PICST_32846)	PICST_30193 PICST_32846	(ECH1 or EHO3)	Valine, leucine and isoleucine Metabolism	1	-1000	1000	0
MGLUTCOAH	(PICST_30193 or PICST_32846)	PICST_30193 PICST_32846	(ECH1 or EHO3)	Valine, leucine and isoleucine Metabolism	1	-1000	1000	0
HACD8	PICST_31190	PICST_31190	HYP	Valine, leucine and isoleucine Metabolism	1	-1000	1000	0
ACACT2	PICST_31707	PICST_31707	ERG10	Valine, leucine and isoleucine Metabolism	1	-1000	1000	0
BACDH	PICST_33103	PICST_33103	ACD99	Valine, leucine and isoleucine Metabolism	1	-1000	1000	0
BACDH2	PICST_33103	PICST_33103	ACD99	Valine, leucine and isoleucine Metabolism	1	-1000	1000	0
MBUTCOAH	PICST_33103	PICST_33103	ACD99	Valine, leucine and isoleucine Metabolism	1	-1000	1000	0
OXOACYT	PICST_33142	PICST_33142	OCA3	Valine, leucine and isoleucine Metabolism	1	-1000	1000	0
ILETAm	PICST_34985	PICST_34985	HYP	Valine, leucine and isoleucine Metabolism	1	0	1000	0
LEUTAm	PICST_34985	PICST_34985	HYP	Valine, leucine and isoleucine Metabolism	1	0	1000	0
OMCDCm	PICST_34985	PICST_34985	HYP	Valine, leucine and isoleucine Metabolism	0	0	1000	0
VALTAm	PICST_34985	PICST_34985	HYP	Valine, leucine and isoleucine Metabolism	1	0	1000	0
HIBUTDH	PICST_35865	PICST_35865	HIB1	Valine, leucine and isoleucine Metabolism	1	-1000	1000	0
3MOBDC	(PICST_64926 or PICST_86443)	PICST_64926 PICST_86443	(PDC1 or PDC2)	Valine, leucine and isoleucine Metabolism	0	0	1000	0
3MOPDC	(PICST_64926 or PICST_86443)	PICST_64926 PICST_86443	(PDC1 or PDC2)	Valine, leucine and isoleucine Metabolism	0	0	1000	0
4MOPDC	(PICST_64926 or PICST_86443)	PICST_64926 PICST_86443	(PDC1 or PDC2)	Valine, leucine and isoleucine Metabolism	0	0	1000	0
OIVALDH	PICST_70031	PICST_70031	HYP	Valine, leucine and isoleucine Metabolism	1	-1000	1000	0

KICAPDH	(PICST_79721 and PICST_68297 and PICST_69303)	PICST_79721 PICST_68297 PICST_69303	(KGD1 and KGD2 and LPD1)	Valine, leucine and isoleucine Metabolism	1	-1000	1000	0
OVALDH	(PICST_79721 and PICST_68297 and PICST_69303)	PICST_79721 PICST_68297 PICST_69303	(KGD1 and KGD2 and LPD1)	Valine, leucine and isoleucine Metabolism	1	-1000	1000	0
ILETA	PICST_84005	PICST_84005	BAT2	Valine, leucine and isoleucine Metabolism	1	-1000	1000	0
LEUTA	PICST_84005	PICST_84005	BAT2	Valine, leucine and isoleucine Metabolism	1	-1000	1000	0
OMCDC	PICST_84005	PICST_84005	BAT2	Valine, leucine and isoleucine Metabolism	0	0	1000	0
VALTA	PICST_84005	PICST_84005	BAT2	Valine, leucine and isoleucine Metabolism	1	-1000	1000	0
PYDAMK	(PICST_31981 or PICST_34206)	PICST_31981 PICST_34206	(BUD17 or BUD16)	Vitamin B6 Metabolism	0	0	1000	0
PYDXK	(PICST_31981 or PICST_34206)	PICST_31981 PICST_34206	(BUD17 or BUD16)	Vitamin B6 Metabolism	0	0	1000	0
PYDXNK	(PICST_31981 or PICST_34206)	PICST_31981 PICST_34206	(BUD17 or BUD16)	Vitamin B6 Metabolism	0	0	1000	0
PDX5PO	PICST_50793	PICST_50793	PDX3	Vitamin B6 Metabolism	1	-1000	1000	0
PYAM5PO	PICST_50793	PICST_50793	PDX3	Vitamin B6 Metabolism	0	0	1000	0
PYDXNO	PICST_50793	PICST_50793	PDX3	Vitamin B6 Metabolism	0	0	1000	0
PYDXO	PICST_50793	PICST_50793	PDX3	Vitamin B6 Metabolism	1	-1000	1000	0
PYD5PS	(PICST_57750 or PICST_57121)	PICST_57750 PICST_57121	(HYP1 or HYP2)	Vitamin B6 Metabolism	0	0	1000	0
PYDDH	PICST_59193	PICST_59193	PLR1	Vitamin B6 Metabolism	1	-1000	1000	0
Biomass				Purine Metabolism	0	0	1000	1
GMPPRPP	PICST_66841	PICST_66841		[]	1	-1000	1000	0
PIPLC	PICST_70308	PICST_70308		Arginine biosynthesis	1	-1000	1000	0
ALT	PICST_70108	PICST_70108		Glycerophospholipid metabolism	1	0	1000	0
PD	PICST_81277	PICST_81277		Alanine, aspartate and glutamate metabolism	1	-1000	1000	0

GC	(PICST_40180 or PICST_55334)	PICST_40180 PICST_55334		Alanine, aspartate and glutamate metabolism	0	0	1000	0
4AB	(PICST_46781 or PICST_54153)	PICST_46781 PICST_54153		Alanine, aspartate and glutamate metabolism	1	-1000	1000	0
ALDH	PICST_40468	PICST_40468		[]	0	0	1000	0
GUA				Purine metabolism	1	0	1000	0
GUR				Glycine, serine and threonine metabolism	1	-1000	0	0
CDPSPT	PICST_51913	PICST_51913		Pyrimidine metabolism	1	-1000	1000	0
URH	PICST_60105	PICST_60105		Transport	1	-1000	1000	0
MAKG				Transport	1	0	1000	0
Ht				Citrate cycle (TCA cycle)	0	0	1000	0
ICDH	(PICST_42313 or PICST_91057)	PICST_42313 PICST_91057		Alanine, aspartate and glutamate metabolism	0	0	1000	0
PYRC	PICST_55391	PICST_55391		Alanine, aspartate and glutamate metabolism	1	-1000	1000	0
PYRCm	PICST_55391	PICST_55391		Transport	1	-1000	1000	0
MOC	PICST_70693	PICST_70693		[]	1	-1000	1000	0
GLUD2	PICST_82969	PICST_82969	GDH3	Nitrogen Metabolism	1	-1000	1000	0
