# Supplementary material for: Elucidating redox balance shift in Scheffersomyces stipitis’ fermentative metabolism using a modified genome-scale metabolic model
Source: Microb Cell Fact. 2018 Sep 5;17:140. doi: 10.1186/s12934-018-0983-y (PMC6126012; doi:10.1186/s12934-018-0983-y)
Supplement: Supplementary file 6 — Additional file 6: Table S6. All cytosolic reactions that use NADH as a cofactor with non-zero flux. [file 12934_2018_983_MOESM6_ESM.pdf]

Table S6: All cytosolic reactions that use NADH as a cofactor with non-zero flux

Rxns	Reaction Equation
GAPD	-1 nad[c] +-1 pi[c] +-1 g3p[c] <-> 1 h[c]+1 nadh[c]+1 13dpg[c]
XDH	-1 h[c] +-1 nadh[c] +-1 xylu-D[c] <-> 1 nad[c]+1 xylt[c]
PGCD	-1 nad[c] +-1 3pg[c] -> 1 h[c]+1 nadh[c]+1 3php[c]
SACCD2	-1 h2o[c] +-1 nad[c] +-1 saccrp-L[c] <-> 1 h[c]+1 nadh[c]+1 akg[c]+1 lys-L[c]
IPMD	-1 nad[c] +-1 3c2hmp[c] -> 1 h[c]+1 nadh[c]+1 3c4mop[c]
HISTD	-1 h2o[c] +-2 nad[c] +-1 histd[c] -> 3 h[c]+2 nadh[c]+1 his-L[c]
C3STDH2	-1 nad[c] +-1 zym_int1[c] -> 1 h[c]+1 nadh[c]+1 co2[c]+1 zym_int2[c]
C3STDH1	-1 nad[c] +-1 4mzym_int1[c] -> 1 h[c]+1 nadh[c]+1 co2[c]+1 4mzym_int2[c]
IMPD	-1 h2o[c] +-1 nad[c] +-1 imp[c] -> 1 h[c]+1 nadh[c]+1 xmp[c]
ALCDH	-1 etoh[c] +-1 nad[c] <-> 1 h[c]+1 acald[c]+1 nadh[c]
NADHDH	-1 h[c] +-1 nadh[c] +-1 q6[m] -> 1 nad[c]+1 q6h2[m]
XYLR1	-1 h[c] +-1 nadh[c] +-1 xyl-D[c] -> 1 nad[c]+1 xylt[c]
PYRC	-1 h[c] +-1 nadh[c] +-1 glu-L[c] <-> 2 h2o[c]+1 nad[c]+1 1pyr5c[c]
AASAD2	-1 h[c] +-1 nadh[c] +-1 atp[c] +-1 L2aadp[c] -> 1 nad[c]+1 amp[c]+1 ppi[c]+1 L2aadp6sa[c]
HSD1	-1 h[c] +-1 nadh[c] +-1 aspsa[c] -> 1 nad[c]+1 hom-L[c]
HACD8	-1 nad[c] +-1 3hmbcoa[c] <-> 1 h[c]+1 nadh[c]+1 2maacoa[c]
OIVALDH	-1 nad[c] +-1 coa[c] +-1 3mop[c] <-> 1 nadh[c]+1 co2[c]+1 2mbcoa[c]
C22STDS1	-1 h[c] +-1 nadh[c] +-1 o2[c] +-1 ergtrol[c] -> 2 h2o[c]+1 nad[c]+1 ergtetrol[c]
G3PD1	-1 h[c] +-1 nadh[c] +-1 dhap[c] -> 1 nad[c]+1 glyc3p[c]
CYB5R	-1 nadh[c] +-2 ficytb5[c] <-> 1 h[c]+1 nad[c]+2 focytb5[c]

	Solution 1						Solution 2					
	NADH Point 1		NADH Point 3		NADH Point 3-1		NADH Point 1		NADH Point 3		NADH Point 3-1	
**Rxns**	**Produced**	**Consumed**	**Produced**	**Consumed**	**Produced**	**Consumed**	**Produced**	**Consumed**	**Produced**	**Consumed**	**Produced**	**Consumed**
GAPD	6.657		7.242		0.585		6.657		7.242		0.585	
XDH	4.852		4.964		0.112		4.852		4.964		0.112	
PGCD	0.124		0.109		-0.015		0.124		0.109		-0.015	
SACCD2	0.041		0.036		-0.005		0.041		0.036		-0.005	
IPMD	0.038		0.034		-0.005		0.038		0.034		-0.005	
HISTD	0.031		0.028		-0.004		0.031		0.028		-0.004	
C3STDH2	0.009		0.008		-0.001		0.009		0.008		-0.001	
C3STDH1	0.009		0.008		-0.001		0.009		0.008		-0.001	
IMPD	0.007		0.006		-0.001		0.007		0.006		-0.001	
ALCDH		5.324		6.070		0.746		5.324		6.070		0.746
NADHDH		3.490		1.196		-2.294		0.000		0.000		0.000
XYLR1		2.590		4.539		1.950		2.590		4.539		1.950
PYRC		0.223		0.505		0.282		0.223		0.505		0.282
AASAD2		0.041		0.036		-0.005		0.041		0.036		-0.005
HSD1		0.037		0.033		-0.004		0.037		0.033		-0.004
HACD8		0.023		0.020		-0.003		0.023		0.020		-0.003
OIVALDH		0.023		0.020		-0.003		0.023		0.020		-0.003
C22STDS1		0.009		0.008		-0.001		0.009		0.008		-0.001
G3PD1		0.005		0.004		-0.001		3.495		1.201		-2.295
CYB5R		0.005		0.004		-0.001		0.005		0.004		-0.001
	**Produced**	**11.770**	**Produced**	**12.435**	**Produced**	**0.666**	**Produced**	**11.770**	**Produced**	**12.435**	**Produced**	**0.666**
	**Consumed**	**11.770**	**Consumed**	**12.435**	**Consumed**	**0.666**	**Consumed**	**11.770**	**Consumed**	**12.435**	**Consumed**	**0.666**
