# Supplementary material for: Elucidating redox balance shift in Scheffersomyces stipitis’ fermentative metabolism using a modified genome-scale metabolic model
Source: Microb Cell Fact. 2018 Sep 5;17:140. doi: 10.1186/s12934-018-0983-y (PMC6126012; doi:10.1186/s12934-018-0983-y)
Supplement: Supplementary file 7 — Additional file 7: Table S7. All cytosolic reactions that use NADPH as a cofactor with non-zero flux. [file 12934_2018_983_MOESM7_ESM.pdf]

Table S7: All cytosolic reactions that use NADPH as a cofactor with non-zero flux

Rxns	Reaction Equation
G6PDH	-1 nadp[c] +-1 g6p[c] -> 1 h[c]+1 nadph[c]+1 6pgl[c]
GND	-1 nadp[c] +-1 6pgc[c] -> 1 nadph[c]+1 co2[c]+1 ru5p-D[c]
ALDDH1	-1 nadp[c] +-1 acald[c] +-1 h2o[c] -> 2 h[c]+1 nadph[c]+1 ac[c]
MTHFD	-1 nadp[c] +-1 mlthf[c] <-> 1 nadph[c]+1 methf[c]
PPND	-1 nadp[c] +-1 pphn[c] -> 1 nadph[c]+1 co2[c]+1 34hpp[c]
XYLR	-1 h[c] +-1 nadph[c] +-1 xyl-D[c] -> 1 nadp[c]+1 xylt[c]
GLUD2	-1 h2o[c] +-1 nadp[c] +-1 glu-L[c] <-> 1 h[c]+1 nadph[c]+1 akg[c]+1 nh4[c]
P5CR	-2 h[c] +-1 nadph[c] +-1 1pyr5c[c] -> 1 nadp[c]+1 pro-L[c]
HMGCOAR	-2 nadp[c] +-1 coa[c] +-1 mev-R[c] <-> 2 h[c]+2 nadph[c]+1 hmgcoa[c]
SULRy	-3 nadp[c] +-3 h2o[c] +-1 h2s[c] <-> 5 h[c]+3 nadph[c]+1 so3[c]
SACCD1	-1 h[c] +-1 nadph[c] +-1 glu-L[c] +-1 L2aadp6sa[c] <-> 1 nadp[c]+1 h2o[c]+1 saccrp-L[c]
FAS80COA_L	-9 h[c] +-6 nadph[c] +-1 accoa[c] +-3 malcoa[c] -> 6 nadp[c]+3 h2o[c]+3 co2[c]+3 coa[c]+1 octacoa[c]
ASAD	-1 h[c] +-1 nadph[c] +-1 4pasp[c] -> 1 nadp[c]+1 pi[c]+1 aspsa[c]
SHK3D	-1 h[c] +-1 nadph[c] +-1 3dhsk[c] -> 1 nadp[c]+1 skm[c]
C4STMO2	-3 h[c] +-3 nadph[c] +-3 o2[c] +-1 4mzym[c] -> 3 nadp[c]+4 h2o[c]+1 zym_int1[c]
C4STMO1	-3 h[c] +-3 nadph[c] +-3 o2[c] +-1 44mzym[c] -> 3 nadp[c]+4 h2o[c]+1 4mzym_int1[c]
LNS14DM	-2 h[c] +-3 nadph[c] +-3 o2[c] +-1 lanost[c] -> 3 nadp[c]+4 h2o[c]+1 for[c]+1 44mctr[c]
TRDR	-1 h[c] +-1 nadph[c] +-1 trdox[c] -> 1 nadp[c]+1 trdrd[c]
MTHFR2	-2 h[c] +-1 nadph[c] +-1 mlthf[c] -> 1 nadp[c]+1 5mthf[c]
FAS100	-3 h[c] +-2 nadph[c] +-1 octa[c] +-1 malcoa[c] -> 2 nadp[c]+1 h2o[c]+1 co2[c]+1 coa[c]+1 dca[c]
FAS120	-3 h[c] +-2 nadph[c] +-1 dca[c] +-1 malcoa[c] -> 2 nadp[c]+1 h2o[c]+1 co2[c]+1 coa[c]+1 ddca[c]
FAS120COA	-3 h[c] +-2 nadph[c] +-1 dcacoa[c] +-1 malcoa[c] -> 2 nadp[c]+1 h2o[c]+1 co2[c]+1 coa[c]+1 ddcacoa[c]
FAS140COA	-3 h[c] +-2 nadph[c] +-1 malcoa[c] +-1 ddcacoa[c] -> 2 nadp[c]+1 h2o[c]+1 co2[c]+1 coa[c]+1 tdcoa[c]
FAS140	-3 h[c] +-2 nadph[c] +-1 ddca[c] +-1 malcoa[c] -> 2 nadp[c]+1 h2o[c]+1 co2[c]+1 coa[c]+1 ttdca[c]
SQLS	-1 h[c] +-1 nadph[c] +-2 frdp[c] -> 1 nadp[c]+2 ppi[c]+1 sql[c]
SQLE	-1 h[c] +-1 nadph[c] +-1 o2[c] +-1 sql[c] -> 1 nadp[c]+1 h2o[c]+1 Ssq23epx[c]
C3STKR2	-1 h[c] +-1 nadph[c] +-1 zym_int2[c] -> 1 nadp[c]+1 zymst[c]
C3STKR1	-1 h[c] +-1 nadph[c] +-1 4mzym_int2[c] -> 1 nadp[c]+1 4mzym[c]
C14STR	-1 h[c] +-1 nadph[c] +-1 44mctr[c] -> 1 nadp[c]+1 44mzym[c]

C24STR	-1 h[c] +-1 nadph[c] +-1 ergtetrol[c] -> 1 nadp[c]+1 ergst[c]
C5STDS	-1 h[c] +-1 nadph[c] +-1 o2[c] +-1 epist[c] -> 1 nadp[c]+2 h2o[c]+1 ergtrol[c]
FAS160COA	-3 h[c] +-2 nadph[c] +-1 tdcoa[c] +-1 malcoa[c] -> 2 nadp[c]+1 h2o[c]+1 co2[c]+1 coa[c]+1 pmtcoa[c]
FAS160	-3 h[c] +-2 nadph[c] +-1 ttdca[c] +-1 malcoa[c] -> 2 nadp[c]+1 h2o[c]+1 co2[c]+1 coa[c]+1 hdca[c]
FAS180COA	-3 h[c] +-2 nadph[c] +-1 pmtcoa[c] +-1 malcoa[c] -> 2 nadp[c]+1 h2o[c]+1 co2[c]+1 coa[c]+1 stcoa[c]
FAS60COA_L	-6 h[c] +-4 nadph[c] +-1 accoa[c] +-2 malcoa[c] -> 4 nadp[c]+2 h2o[c]+2 co2[c]+2 coa[c]+1 hexacoa[c]
DHFRi	-1 h[c] +-1 nadph[c] +-1 dhf[c] -> 1 nadp[c]+1 thf[c]
DESAT18_2	-1 h[c] +-1 nadph[c] +-1 o2[c] +-1 ocdceacoa[c] -> 1 nadp[c]+2 h2o[c]+1 ocdcyacoa[c]
DESAT20_5	-5 h[c] +-5 nadph[c] +-5 o2[c] +-1 eicosacoa[c] -> 5 nadp[c]+10 h2o[c]+1 eicosapencoa[c]
DESAT18_3	-1 h[c] +-1 nadph[c] +-1 o2[c] +-1 ocdcyacoa[c] -> 1 nadp[c]+2 h2o[c]+1 ocdctacoa[c]

	Solution 1						Solution 2					
	NADPH Pt 1		NADPH Pt 3		NADPH Pt 3- Pt 1		NADPH Pt 1		NADPH Pt 3		NADPH Pt 3- Pt 1	
**Rxns**	**Produced**	**Consumed**	**Produced**	**Consumed**	**Produced**	**Consumed**	**Produced**	**Consumed**	**Produced**	**Consumed**	**Produced**	**Consumed**
G6PDH	1.797		0.905		-0.892		1.797		0.905		-0.892	
GND	1.797		0.905		-0.892		1.797		0.905		-0.892	
ALDDH1	0.307		0.270		-0.037		0.307		0.270		-0.037	
MTHFD	0.047		0.041		-0.006		0.047		0.041		-0.006	
PPND	0.012		0.010		-0.001		0.012		0.010		-0.001	
XYLR		2.410		0.461		-1.950		2.410		0.461		-1.950
GLUD2		0.781		0.686		-0.094		0.781		0.686		-0.094
P5CR		0.223		0.505		0.282		0.223		0.505		0.282
HMGCOAR		0.109		0.096		-0.013		0.109		0.096		-0.013
SULRy		0.050		0.044		-0.006		0.050		0.044		-0.006
SACCD1		0.041		0.036		-0.005		0.041		0.036		-0.005
FAS80COA_L		0.037		0.033		-0.005		0.037		0.033		-0.005
ASAD		0.037		0.033		-0.004		0.037		0.033		-0.004
SHK3D		0.033		0.029		-0.004		0.033		0.029		-0.004
C4STMO2		0.027		0.024		-0.003		0.027		0.024		-0.003
C4STMO1		0.027		0.024		-0.003		0.027		0.024		-0.003
LNS14DM		0.027		0.024		-0.003		0.027		0.024		-0.003
TRDR		0.023		0.020		-0.003		0.023		0.020		-0.003
MTHFR2		0.018		0.015		-0.002		0.018		0.015		-0.002
FAS100		0.012		0.011		-0.001		0.012		0.011		-0.001
FAS120		0.000		0.000		0.000		0.012		0.010		-0.001
FAS120COA		0.012		0.010		-0.001		0.000		0.000		0.000
FAS140COA		0.000		0.000		0.000		0.009		0.008		-0.001
FAS140		0.009		0.008		-0.001		0.000		0.000		0.000
SQLS		0.009		0.008		-0.001		0.009		0.008		-0.001
SQLE		0.009		0.008		-0.001		0.009		0.008		-0.001
C3STKR2		0.009		0.008		-0.001		0.009		0.008		-0.001
C3STKR1		0.009		0.008		-0.001		0.009		0.008		-0.001
C14STR		0.009		0.008		-0.001		0.009		0.008		-0.001

C24STR		0.009		0.008		-0.001		0.009		0.008		-0.001
C5STDS		0.009		0.008		-0.001		0.009		0.008		-0.001
FAS160COA		0.000		0.000		0.000		0.008		0.007		-0.001
FAS160		0.008		0.007		-0.001		0.000		0.000		0.000
FAS180COA		0.003		0.003		0.000		0.003		0.003		0.000
FAS60COA_L		0.002		0.002		0.000		0.002		0.002		0.000
DHFRi		0.002		0.002		0.000		0.002		0.002		0.000
DESAT18_2		0.001		0.001		0.000		0.001		0.001		0.000
DESAT20_5		0.001		0.000		0.000		0.001		0.000		0.000
DESAT18_3		0.000		0.000		0.000		0.000		0.000		0.000
	**Produced**	**3.958**	**Produced**	**2.131**	**Produced**	**-1.827**	**Produced**	**3.958**	**Produced**	**2.131**	**Produced**	**-1.827**
	**Consumed**	**3.958**	**Consumed**	**2.131**	**Consumed**	**-1.827**	**Consumed**	**3.958**	**Consumed**	**2.131**	**Consumed**	**-1.827**
