# Supplementary material for: Elucidating redox balance shift in Scheffersomyces stipitis’ fermentative metabolism using a modified genome-scale metabolic model
Source: Microb Cell Fact. 2018 Sep 5;17:140. doi: 10.1186/s12934-018-0983-y (PMC6126012; doi:10.1186/s12934-018-0983-y)
Supplement: Supplementary file 8 — Additional file 8: Table S8. Genome-wide comparison for Trial I. [file 12934_2018_983_MOESM8_ESM.pdf]

Table S8: Genome-wide comparison for Trial I

Rxn	Load	Flux Pt 1	Flux Pt 3	Abs Flux Diff	TPM Aero	TPM OL	TPM Change	Flux Change <10%?	TPM Change <10%	Protein Assocation?	Flux?	Active w/Protein?	Correct?
ARGSS	-0.0038	0.0285	0.0251	-0.0034	551.2	308.4	-242.8	0	0	1	1	1	1
ASPTAm	0.3034	0.2740	0.5499	0.2758	1690.8	701.6	-989.2	0	0	1	1	1	0
ARGSL	-0.0038	0.0285	0.0251	-0.0034	122.2	78.5	-43.7	0	0	1	1	1	1
ASPTA	-0.2802	-0.4493	-0.7040	0.2547	768.8	63.9	-704.9	0	0	1	1	1	0
ALATA_L	-0.3226	-0.1295	-0.4228	0.2933	64.2	21.6	-42.6	0	0	1	1	1	0
CBPS	-0.0066	0.0498	0.0438	-0.0060	75.5	48.5	-26.9	0	0	1	1	1	1
GLNS	-0.0306	0.2309	0.2030	-0.0278	1507.1	2947.8	1440.7	0	0	1	1	1	0
ASNS1	-0.0031	0.0237	0.0208	-0.0029	237.5	308.1	70.6	0	0	1	1	1	0
GLUPRT	-0.0021	0.0157	0.0138	-0.0019	83.1	201.3	118.2	0	0	1	1	1	0
CHTNS	-0.0094	0.0709	0.0624	-0.0086	203.7	162.3	-41.4	0	0	1	1	1	1
CYB5R	-0.0006	0.0049	0.0043	-0.0006	1012.4	546.0	-466.4	0	0	1	1	1	1
ACGAMPP	-0.0094	0.0709	0.0624	-0.0086	65.9	54.2	-11.7	0	0	1	1	1	1
GF6PTA	-0.0094	0.0709	0.0624	-0.0086	133.1	94.8	-38.2	0	0	1	1	1	1
ACGAM6PS	-0.0094	0.0709	0.0624	-0.0086	41.1	20.5	-20.6	0	0	1	1	1	1
ACGAMPM	-0.0094	0.0709	0.0624	-0.0086	46.9	27.0	-19.9	0	0	1	1	1	1
P5CR	0.3102	0.2231	0.5051	0.2820	141.3	77.5	-63.8	0	0	1	1	1	0
OCBT	-0.0038	0.0285	0.0251	-0.0034	74.9	23.4	-51.5	0	0	1	1	1	1
ACOTAm	-0.0038	0.0285	0.0251	-0.0034	67.8	40.8	-26.9	0	0	1	1	1	1
ACGKm	-0.0038	0.0285	0.0251	-0.0034	107.9	96.2	-11.7	0	0	1	1	1	1
AGPRm	-0.0038	0.0285	0.0251	-0.0034	107.9	96.2	-11.7	0	0	1	1	1	1
ORNTACim	-0.0038	0.0285	0.0251	-0.0034	67.2	29.4	-37.8	0	0	1	1	1	1
PRO1m	0.3135	0.1982	0.4832	0.2850	40.1	12.4	-27.7	0	0	1	1	1	0
SUCCDHpm	-0.0031	0.0233	0.0205	-0.0028	95.7	83.5	-12.2	0	0	1	1	1	1
MDHm	-0.0122	0.0920	0.0809	-0.0111	3314.2	868.7	-2445.5	0	0	1	1	1	1
ICDH1m	-0.0230	0.1730	0.1521	-0.0209	552.0	334.1	-217.9	0	0	1	1	1	1
ACONHm	-0.0260	0.1963	0.1726	-0.0237	376.2	312.6	-63.5	0	0	1	1	1	1
CITSm	-0.0260	0.1963	0.1726	-0.0237	1857.9	126.4	-1731.5	0	0	1	1	1	1
FUMm	-0.0122	0.0920	0.0809	-0.0111	564.7	255.4	-309.2	0	0	1	1	1	1
SHSL1	-0.0012	0.0088	0.0077	-0.0011	94.9	97.0	2.0	0	1	0	1	0	0
SHSL4	0.0012	-0.0088	-0.0077	-0.0011	94.9	97.0	2.0	0	1	0	1	0	0
HSD1	-0.0049	0.0371	0.0326	-0.0045	157.0	103.1	-53.9	0	0	1	1	1	1
METAT	-0.0012	0.0088	0.0077	-0.0011	752.4	1925.3	1172.9	0	0	1	1	1	0

ASPK	-0.0049	0.0371	0.0326	-0.0045	85.0	107.3	22.3	0	0	1	1	1	0
AHC	-0.0012	0.0088	0.0077	-0.0011	1443.7	1094.6	-349.1	0	0	1	1	1	1
METS	-0.0023	0.0175	0.0154	-0.0021	1762.6	875.5	-887.0	0	0	1	1	1	1
ASAD	-0.0049	0.0371	0.0326	-0.0045	269.9	451.3	181.4	0	0	1	1	1	0
SERAT	-0.0022	0.0168	0.0147	-0.0020	0.0	0.0	0.0	0	0	0	1	0	0
CYSS	-0.0022	0.0168	0.0147	-0.0020	296.5	144.5	-152.0	0	0	1	1	1	1
CYSTL	-0.0012	0.0088	0.0077	-0.0011	130.3	100.6	-29.7	0	0	1	1	1	1
EX_co2(e)	-0.2590	7.8617	7.6262	-0.2355	0.0	0.0	0.0	1	0	0	1	0	0
EX_etoh(e)	0.8201	5.3240	6.0695	0.7456	0.0	0.0	0.0	0	0	0	1	0	0
EX_for(e)	-0.0012	0.0091	0.0080	-0.0011	0.0	0.0	0.0	0	0	0	1	0	0
EX_h(e)	-2.1034	6.9499	5.0377	-1.9122	0.0	0.0	0.0	0	0	0	1	0	0
EX_h2o(e)	-1.2363	4.5070	3.3830	-1.1239	0.0	0.0	0.0	0	0	0	1	0	0
EX_nh4(e)	0.1345	-1.0141	-0.8918	-0.1223	0.0	0.0	0.0	0	0	0	1	0	0
EX_o2(e)	1.0000	-2.3333	-1.4242	-0.9091	0.0	0.0	0.0	0	0	0	1	0	0
EX_pi(e)	0.0055	-0.0416	-0.0366	-0.0050	0.0	0.0	0.0	0	0	0	1	0	0
EX_so4(e)	0.0022	-0.0168	-0.0147	-0.0020	0.0	0.0	0.0	0	0	0	1	0	0
EX_xyl-D(e)	0.0000	-5.0000	-5.0000	0.0000	0.0	0.0	0.0	1	0	0	1	0	0
EX_xylt(e)	-0.1237	0.1482	0.0358	-0.1125	0.0	0.0	0.0	0	0	0	1	0	0
Hexacoa_ex	0.0000	0.0002	0.0002	0.0000	0.0	0.0	0.0	0	0	0	1	0	0
FACOAL180	-0.0004	0.0028	0.0025	-0.0003	1246.0	166.5	-1079.5	0	0	1	1	1	1
FAS100ACPm	-0.0004	0.0030	0.0027	-0.0004	49.6	16.4	-33.2	0	0	1	1	1	1
FAS120ACPm	-0.0004	0.0030	0.0027	-0.0004	49.6	16.4	-33.2	0	0	1	1	1	1
FAS140ACPm	-0.0004	0.0030	0.0027	-0.0004	49.6	16.4	-33.2	0	0	1	1	1	1
FAS160ACPm	-0.0004	0.0030	0.0027	-0.0004	49.6	16.4	-33.2	0	0	1	1	1	1
FAS180ACPm	-0.0004	0.0030	0.0027	-0.0004	49.6	16.4	-33.2	0	0	1	1	1	1
FAS200ACPm	0.0000	0.0002	0.0002	0.0000	49.6	16.4	-33.2	0	0	1	1	1	1
FAS220ACPm	0.0000	0.0001	0.0001	0.0000	49.6	16.4	-33.2	0	0	1	1	1	1
FAS80ACPm_L	-0.0004	0.0030	0.0027	-0.0004	49.6	16.4	-33.2	0	0	1	1	1	1
MCOATAm	-0.0032	0.0244	0.0215	-0.0029	22.9	14.7	-8.2	0	0	1	1	1	1
DESAT16	-0.0001	0.0008	0.0007	-0.0001	623.4	2303.6	1680.1	0	0	1	1	1	0
DESAT18	-0.0005	0.0041	0.0036	-0.0005	623.4	2303.6	1680.1	0	0	1	1	1	0
ACOATAm	-0.0004	0.0030	0.0027	-0.0004	63.8	195.8	132.0	0	0	1	1	1	0
FA180ACPH	-0.0004	0.0028	0.0025	-0.0003	63.8	195.8	132.0	0	0	1	1	1	0
FA200ACPH	0.0000	0.0001	0.0001	0.0000	63.8	195.8	132.0	0	0	1	1	1	0

FA220ACPH	0.0000	0.0001	0.0001	0.0000	63.8	195.8	132.0	0	0	1	1	1	0
FAS100COA	-0.0008	0.0060	0.0053	-0.0007	63.8	195.8	132.0	0	0	1	1	1	0
FAS120COA	-0.0008	0.0058	0.0051	-0.0007	63.8	195.8	132.0	0	0	1	1	1	0
FAS140COA	-0.0006	0.0047	0.0041	-0.0006	63.8	195.8	132.0	0	0	1	1	1	0
FAS160COA	-0.0006	0.0041	0.0036	-0.0005	63.8	195.8	132.0	0	0	1	1	1	0
FAS180COA	-0.0002	0.0016	0.0014	-0.0002	63.8	195.8	132.0	0	0	1	1	1	0
FAS60COA_L	-0.0001	0.0005	0.0004	-0.0001	63.8	195.8	132.0	0	0	1	1	1	0
FAS80COA_L	-0.0008	0.0062	0.0055	-0.0008	63.8	195.8	132.0	0	0	1	1	1	0
DESAT18_3	0.0000	0.0001	0.0001	0.0000	0.0	0.0	0.0	0	0	0	1	0	0
DESAT20_5	0.0000	0.0001	0.0001	0.0000	0.0	0.0	0.0	0	0	0	1	0	0
DHFRi	-0.0002	0.0018	0.0015	-0.0002	99.0	59.2	-39.8	0	0	1	1	1	1
MTHFR2	-0.0023	0.0175	0.0154	-0.0021	130.2	83.9	-46.3	0	0	1	1	1	1
MTHFD	-0.0062	0.0471	0.0414	-0.0057	265.6	280.4	14.8	0	0	1	1	1	0
MTHFC	-0.0062	0.0471	0.0414	-0.0057	206.7	87.4	-119.3	0	0	1	1	1	1
MAN6PI	0.0149	-0.1121	-0.0986	-0.0135	118.9	87.4	-31.5	0	0	1	1	1	1
MAN1PGT	-0.0149	0.1121	0.0986	-0.0135	816.8	839.1	22.3	0	1	0	1	0	0
PMANM	0.0149	-0.1121	-0.0986	-0.0135	315.8	204.0	-111.8	0	0	1	1	1	1
DOLPMMer	-0.0149	0.1121	0.0986	-0.0135	8.5	5.2	-3.4	0	0	1	1	1	1
DOLPMTcer	-0.0149	0.1121	0.0986	-0.0135	0.0	0.0	0.0	0	0	0	1	0	0
GAT1	-0.0006	0.0049	0.0043	-0.0006	49.7	51.2	1.5	0	1	0	1	0	0
AGAT	-0.0006	0.0049	0.0043	-0.0006	205.0	141.1	-63.9	0	0	1	1	1	1
DAGPYP	0.0000	0.0002	0.0002	0.0000	87.1	28.8	-58.3	0	0	1	1	1	1
PLD	0.0000	0.0000	0.0000	0.0000	16.9	13.9	-3.0	0	0	1	1	1	1
G3PD1	-2.5242	3.4953	1.2006	-2.2947	169.4	107.6	-61.8	0	0	1	1	1	1
G3PDm	-2.5235	3.4904	1.1963	-2.2941	38.5	35.8	-2.7	0	0	1	1	1	1
PSERDm	-0.0001	0.0006	0.0006	-0.0001	224.4	106.8	-117.6	0	0	1	1	1	1
PSERT	-0.0165	0.1242	0.1092	-0.0150	91.2	58.9	-32.4	0	0	1	1	1	1
PSPL	-0.0165	0.1242	0.1092	-0.0150	91.2	57.2	-34.0	0	0	1	1	1	1
AGT	-0.0031	0.0233	0.0205	-0.0028	343.8	57.2	-286.6	0	0	1	1	1	1
THRS	-0.0049	0.0371	0.0326	-0.0045	124.0	146.0	21.9	0	0	1	1	1	0
THRD	-0.0012	0.0088	0.0077	-0.0011	41.4	61.9	20.6	0	0	1	1	1	0
HSK	-0.0049	0.0371	0.0326	-0.0045	76.2	153.6	77.4	0	0	1	1	1	0
PGCD	-0.0165	0.1242	0.1092	-0.0150	403.6	518.3	114.7	0	0	1	1	1	0
PGM	-0.6597	-6.5332	-7.1329	0.5997	1897.1	3367.3	1470.1	1	0	1	1	1	1

PFK	0.4617	2.2417	2.6614	0.4197	62.0	83.6	21.7	0	0	1	1	1	1
ENO	0.6597	6.5332	7.1329	0.5997	1789.5	2753.2	963.7	1	0	1	1	1	1
GAPD	0.6432	6.6574	7.2421	0.5847	4078.4	6138.2	2059.7	1	0	1	1	1	1
PGK	-0.6432	-6.6574	-7.2421	0.5847	1332.1	1983.2	651.1	1	0	1	1	1	1
PYK	0.6686	6.4663	7.0741	0.6078	565.8	747.2	181.4	1	0	1	1	1	1
G6PI	1.0023	-1.9586	-1.0474	-0.9112	872.7	529.9	-342.7	0	0	1	1	1	1
FBA	0.4617	2.2417	2.6614	0.4197	1131.1	878.0	-253.2	0	0	1	1	1	0
TPI	0.4623	2.2368	2.6571	0.4203	1369.3	1633.8	264.4	0	0	1	1	1	1
ICL	-0.0031	0.0233	0.0205	-0.0028	651.7	161.0	-490.7	0	0	1	1	1	1
HISTP	-0.0021	0.0157	0.0138	-0.0019	20.4	12.2	-8.2	0	0	1	1	1	1
PRMICI	-0.0021	0.0157	0.0138	-0.0019	40.1	57.3	17.3	0	0	1	1	1	0
IG3PS	-0.0021	0.0157	0.0138	-0.0019	58.8	66.2	7.4	0	0	1	1	1	0
ATPPRT	-0.0021	0.0157	0.0138	-0.0019	97.0	98.1	1.1	0	1	0	1	0	0
HISTD	-0.0021	0.0157	0.0138	-0.0019	78.4	63.1	-15.3	0	0	1	1	1	1
PRAMPC	-0.0021	0.0157	0.0138	-0.0019	78.4	63.1	-15.3	0	0	1	1	1	1
PRATPP	-0.0021	0.0157	0.0138	-0.0019	78.4	63.1	-15.3	0	0	1	1	1	1
IGPDH	-0.0021	0.0157	0.0138	-0.0019	0.0	0.0	0.0	0	0	0	1	0	0
HSTPT	-0.0021	0.0157	0.0138	-0.0019	56.7	37.3	-19.4	0	0	1	1	1	1
HICITDm	-0.0055	0.0411	0.0361	-0.0050	395.8	199.1	-196.7	0	0	1	1	1	1
AATA	-0.0055	0.0411	0.0361	-0.0050	211.6	74.4	-137.2	0	0	1	1	1	1
AASAD2	-0.0055	0.0411	0.0361	-0.0050	83.0	70.3	-12.7	0	0	1	1	1	1
SACCD1	-0.0055	0.0411	0.0361	-0.0050	358.7	541.1	182.4	0	0	1	1	1	0
SACCD2	-0.0055	0.0411	0.0361	-0.0050	509.7	684.5	174.8	0	0	1	1	1	0
OXAGm	-0.0055	0.0411	0.0361	-0.0050	0.0	0.0	0.0	0	0	0	1	0	0
RNTR1	-0.0002	0.0018	0.0015	-0.0002	1550.6	1142.0	-408.6	0	0	1	1	1	1
GSNK	-0.0087	0.0654	0.0575	-0.0079	0.0	0.0	0.0	0	0	0	1	0	0
GHMT	-0.0088	0.0664	0.0584	-0.0080	642.3	277.3	-365.0	0	0	1	1	1	1
CYOR_u6m	-1.9666	4.4147	2.6269	-1.7878	119.6	113.9	-5.7	0	1	0	1	0	0
NADH2-u6t	0.5600	0.9010	1.4101	0.5091	37.9	51.6	13.7	0	0	1	1	1	1
SUCCDH1m	-2.5266	3.5137	1.2168	-2.2970	95.7	83.5	-12.2	0	0	1	1	1	1
ATPSm	-1.9721	5.9601	4.1673	-1.7928	312.9	311.8	-1.1	0	1	0	1	0	0
CYOOm	-0.9833	2.2074	1.3135	-0.8939	39.8	50.4	10.6	0	0	1	1	1	0
PPA	-0.1141	0.8604	0.7567	-0.1038	727.0	463.3	-263.7	0	0	1	1	1	1
DHAD1m	-0.0127	0.0957	0.0841	-0.0115	40.3	132.8	92.5	0	0	1	1	1	0

KARA1m	-0.0127	0.0957	0.0841	-0.0115	1452.4	2713.9	1261.5	0	0	1	1	1	0
XYLK	0.1237	4.8518	4.9642	0.1125	639.5	1416.9	777.4	1	0	1	1	1	1
XYLUR	-0.1237	-4.8518	-4.9642	0.1125	6330.6	10531.5	4200.9	1	0	1	1	1	1
RPE	-0.6886	-0.4685	-1.0945	0.6260	195.6	103.4	-92.3	0	0	1	1	1	0
TKT1	-0.2802	2.1749	1.9202	-0.2548	1458.2	702.8	-755.4	0	0	1	1	1	1
TKT2	-0.2847	2.2084	1.9496	-0.2588	1458.2	702.8	-755.4	0	0	1	1	1	1
PGL	-0.9808	1.7966	0.9049	-0.8917	342.7	194.9	-147.8	0	0	1	1	1	1
GND	-0.9808	1.7966	0.9049	-0.8917	2551.5	775.0	-1776.5	0	0	1	1	1	1
TALA	-0.2847	2.2084	1.9496	-0.2588	2519.9	5673.1	3153.2	0	0	1	1	1	0
RPI	0.2922	-2.2650	-1.9994	-0.2656	425.9	226.9	-199.0	0	0	1	1	1	1
G6PDH	-0.9808	1.7966	0.9049	-0.8917	830.9	311.2	-519.7	0	0	1	1	1	1
PPM	0.0075	-0.0566	-0.0498	-0.0068	464.8	289.5	-175.3	0	0	1	1	1	1
FACOAL200	0.0000	0.0001	0.0001	0.0000	1246.0	166.5	-1079.5	0	0	1	1	1	1
FACOAL220	0.0000	0.0001	0.0001	0.0000	1246.0	166.5	-1079.5	0	0	1	1	1	1
DESAT18_2	-0.0001	0.0010	0.0009	-0.0001	172.3	1220.0	1047.7	0	0	1	1	1	0
PPNDH	-0.0024	0.0180	0.0158	-0.0022	42.8	35.0	-7.8	0	0	1	1	1	1
ANPRT	-0.0005	0.0039	0.0034	-0.0005	74.8	62.1	-12.7	0	0	1	1	1	1
PRAI	-0.0005	0.0039	0.0034	-0.0005	76.4	35.1	-41.3	0	0	1	1	1	1
TYRTAm	0.0015	-0.0116	-0.0102	-0.0014	1690.8	701.6	-989.2	0	0	1	1	1	1
PPND	-0.0015	0.0116	0.0102	-0.0014	35.2	25.1	-10.1	0	0	1	1	1	1
DHQT	-0.0044	0.0335	0.0294	-0.0040	251.1	92.2	-158.9	0	0	1	1	1	1
PHETA1	0.0024	-0.0180	-0.0158	-0.0022	240.5	77.5	-163.0	0	0	1	1	1	1
TRPS1	-0.0005	0.0039	0.0034	-0.0005	158.4	113.4	-45.0	0	0	1	1	1	1
CHORM	-0.0039	0.0296	0.0260	-0.0036	41.5	30.1	-11.4	0	0	1	1	1	1
ANS	-0.0005	0.0039	0.0034	-0.0005	15.7	12.1	-3.7	0	0	1	1	1	1
DDPA	-0.0044	0.0335	0.0294	-0.0040	391.1	429.5	38.4	0	0	1	1	1	0
DHQS	-0.0044	0.0335	0.0294	-0.0040	50.9	51.9	1.0	0	1	0	1	0	0
PSCVT	-0.0044	0.0335	0.0294	-0.0040	50.9	51.9	1.0	0	1	0	1	0	0
SHK3D	-0.0044	0.0335	0.0294	-0.0040	50.9	51.9	1.0	0	1	0	1	0	0
SHKK	-0.0044	0.0335	0.0294	-0.0040	50.9	51.9	1.0	0	1	0	1	0	0
CHORS	-0.0044	0.0335	0.0294	-0.0040	259.6	343.7	84.0	0	0	1	1	1	0
IGPS	-0.0005	0.0039	0.0034	-0.0005	103.7	100.7	-3.1	0	1	0	1	0	0
DASYN	-0.0006	0.0047	0.0041	-0.0006	48.2	39.8	-8.4	0	0	1	1	1	1
MMSAD1	-0.0030	0.0228	0.0200	-0.0027	115.3	7.2	-108.2	0	0	1	1	1	1

ADPT	-0.0012	0.0088	0.0077	-0.0011	366.4	379.9	13.5	0	1	0	1	0	0
RNDR2	-0.0002	0.0013	0.0012	-0.0002	17.6	13.1	-4.5	0	0	1	1	1	1
GARFT	-0.0021	0.0157	0.0138	-0.0019	73.2	79.5	6.3	0	0	1	1	1	0
PRASCS	-0.0021	0.0157	0.0138	-0.0019	109.7	69.7	-40.0	0	0	1	1	1	1
ADK1	-0.0623	0.4697	0.4130	-0.0566	756.4	336.8	-419.6	0	0	1	1	1	1
IMPD	-0.0009	0.0070	0.0061	-0.0008	239.4	946.1	706.7	0	0	1	1	1	0
NDPK1	-0.0254	0.1913	0.1682	-0.0231	1227.9	1228.1	0.2	0	1	0	1	0	0
AICART	-0.0042	0.0314	0.0276	-0.0038	508.5	246.6	-261.9	0	0	1	1	1	1
IMPC	0.0042	-0.0314	-0.0276	-0.0038	508.5	246.6	-261.9	0	0	1	1	1	1
GMPS	-0.0009	0.0070	0.0061	-0.0008	104.1	502.7	398.6	0	0	1	1	1	0
GK3	0.0002	-0.0013	-0.0012	-0.0002	170.2	234.5	64.2	0	0	1	1	1	0
PRFGS	-0.0021	0.0157	0.0138	-0.0019	81.4	82.4	1.0	0	1	0	1	0	0
PRAGS	-0.0021	0.0157	0.0138	-0.0019	80.2	148.5	68.3	0	0	1	1	1	0
PRAIS	-0.0021	0.0157	0.0138	-0.0019	80.2	148.5	68.3	0	0	1	1	1	0
AIRC	-0.0021	0.0157	0.0138	-0.0019	52.5	110.3	57.8	0	0	1	1	1	0
ADSL1	-0.0032	0.0245	0.0215	-0.0029	188.0	235.0	47.0	0	0	1	1	1	0
ADSL2	-0.0021	0.0157	0.0138	-0.0019	188.0	235.0	47.0	0	0	1	1	1	0
ADSS	-0.0032	0.0245	0.0215	-0.0029	437.1	458.0	20.8	0	1	0	1	0	0
ADSK	-0.0022	0.0168	0.0147	-0.0020	135.1	249.3	114.2	0	0	1	1	1	0
DADK	0.0002	-0.0018	-0.0015	-0.0002	0.0	0.0	0.0	0	0	0	1	0	0
PUNP1	-0.0012	0.0088	0.0077	-0.0011	0.0	0.0	0.0	0	0	0	1	0	0
PUNP3	0.0087	-0.0654	-0.0575	-0.0079	0.0	0.0	0.0	0	0	0	1	0	0
RNDR4	-0.0004	0.0031	0.0027	-0.0004	17.6	13.1	-4.5	0	0	1	1	1	1
DHORTS	0.0028	-0.0213	-0.0187	-0.0026	60.1	87.9	27.8	0	0	1	1	1	0
DCMPDA	0.0002	-0.0013	-0.0012	-0.0002	34.1	13.6	-20.4	0	0	1	1	1	1
TMDS	-0.0002	0.0018	0.0015	-0.0002	329.6	118.3	-211.3	0	0	1	1	1	1
OMPDC	-0.0028	0.0213	0.0187	-0.0026	142.9	146.6	3.8	0	1	0	1	0	0
DHORD	-0.0028	0.0213	0.0187	-0.0026	115.0	147.1	32.1	0	0	1	1	1	0
TRDR	-0.0030	0.0229	0.0201	-0.0028	459.4	2219.2	1759.8	0	0	1	1	1	0
CTPS1	-0.0013	0.0101	0.0089	-0.0012	65.4	250.2	184.7	0	0	1	1	1	0
ASPCT	-0.0028	0.0213	0.0187	-0.0026	75.5	48.5	-26.9	0	0	1	1	1	1
CYTK1	0.0007	-0.0054	-0.0047	-0.0007	147.6	91.2	-56.5	0	0	1	1	1	1
UMPK	-0.0017	0.0131	0.0116	-0.0016	147.6	91.2	-56.5	0	0	1	1	1	1
URIDK	0.0004	-0.0031	-0.0027	-0.0004	147.6	91.2	-56.5	0	0	1	1	1	1

ORPT	0.0028	-0.0213	-0.0187	-0.0026	127.6	137.1	9.5	0	0	1	1	1	0
NDPK2	-0.0307	0.2311	0.2032	-0.0279	1227.9	1228.1	0.2	0	1	0	1	0	0
NDPK3	0.0007	-0.0054	-0.0047	-0.0007	1227.9	1228.1	0.2	0	1	0	1	0	0
ALCDH	-0.8201	-5.3240	-6.0695	0.7456	1867.6	4924.1	3056.5	0	0	1	1	1	1
HCITSm	-0.0055	0.0411	0.0361	-0.0050	707.8	522.3	-185.5	0	0	1	1	1	1
ACACT1	-0.0073	0.0547	0.0481	-0.0066	456.1	409.7	-46.4	0	0	1	1	1	1
ACLSm	-0.0127	0.0957	0.0841	-0.0115	282.5	232.5	-50.1	0	0	1	1	1	1
ALDDH1	-0.0407	0.3066	0.2696	-0.0370	199.6	37.5	-162.1	0	0	1	1	1	1
PYRDC	0.7794	5.6306	6.3391	0.7086	577.4	2097.6	1520.3	0	0	1	1	1	1
IPPS	-0.0051	0.0384	0.0338	-0.0046	133.8	158.6	24.8	0	0	1	1	1	0
ACS1	-0.0429	0.3234	0.2844	-0.0390	1952.8	236.5	-1716.3	0	0	1	1	1	1
PDHm	-0.0402	0.3032	0.2667	-0.0366	593.0	327.8	-265.2	0	0	1	1	1	1
ACCOAC	-0.0056	0.0420	0.0370	-0.0051	70.5	156.1	85.6	0	0	1	1	1	0
ACCOACrm	-0.0032	0.0244	0.0215	-0.0029	70.5	156.1	85.6	0	0	1	1	1	0
PC	-0.0371	0.2796	0.2459	-0.0337	199.4	202.9	3.5	0	1	0	1	0	0
IPC224PLC	0.0000	0.0000	0.0000	0.0000	30.7	14.1	-16.6	0	0	1	1	1	1
IPCS224	0.0000	0.0000	0.0000	0.0000	111.1	323.1	212.1	0	0	1	1	1	0
PGMT	0.0199	-0.1501	-0.1320	-0.0181	464.8	289.5	-175.3	0	0	1	1	1	1
GALU	-0.0199	0.1501	0.1320	-0.0181	623.6	913.6	290.0	0	0	1	1	1	0
C5STDS	-0.0012	0.0088	0.0077	-0.0011	541.7	739.8	198.1	0	0	1	1	1	0
C24STR	-0.0012	0.0088	0.0077	-0.0011	50.0	55.7	5.6	0	0	1	1	1	0
C14STR	-0.0012	0.0091	0.0080	-0.0011	85.2	64.2	-20.9	0	0	1	1	1	1
C3STKR1	-0.0012	0.0091	0.0080	-0.0011	48.6	44.9	-3.8	0	0	1	1	1	1
C3STKR2	-0.0012	0.0091	0.0080	-0.0011	48.6	44.9	-3.8	0	0	1	1	1	1
LNS14DM	-0.0012	0.0091	0.0080	-0.0011	134.1	162.3	28.2	0	0	1	1	1	0
LNSTLS	-0.0012	0.0091	0.0080	-0.0011	134.1	162.3	28.2	0	0	1	1	1	0
C3STDH1	-0.0012	0.0091	0.0080	-0.0011	212.7	202.1	-10.6	0	1	0	1	0	0
C3STDH2	-0.0012	0.0091	0.0080	-0.0011	212.7	202.1	-10.6	0	1	0	1	0	0
C22STDS1	-0.0012	0.0088	0.0077	-0.0011	163.4	174.0	10.6	0	0	1	1	1	0
C8STI	-0.0012	0.0088	0.0077	-0.0011	152.3	154.1	1.8	0	1	0	1	0	0
C4STMO1	-0.0012	0.0091	0.0080	-0.0011	2.0	993.0	991.1	0	0	1	1	1	0
C4STMO2	-0.0012	0.0091	0.0080	-0.0011	2.0	993.0	991.1	0	0	1	1	1	0
SQLE	-0.0012	0.0091	0.0080	-0.0011	70.3	118.1	47.8	0	0	1	1	1	0
SAM24MT	-0.0012	0.0088	0.0077	-0.0011	642.8	811.7	168.9	0	0	1	1	1	0

SQLS	-0.0012	0.0091	0.0080	-0.0011	263.2	170.7	-92.6	0	0	1	1	1	1
13GS	-0.0127	0.0957	0.0841	-0.0115	12.8	10.0	-2.8	0	0	1	1	1	1
TRE6PS	-0.0016	0.0119	0.0105	-0.0014	32.4	36.6	4.3	0	0	1	1	1	0
TRE6PP	-0.0016	0.0119	0.0105	-0.0014	81.8	55.9	-25.9	0	0	1	1	1	1
GLYGS	-0.0056	0.0425	0.0374	-0.0051	624.3	542.4	-81.9	0	0	1	1	1	1
14GBEZ	-0.0056	0.0425	0.0374	-0.0051	183.5	140.6	-42.9	0	0	1	1	1	1
BPNT	-0.0022	0.0168	0.0147	-0.0020	118.8	57.3	-61.5	0	0	1	1	1	1
PAPSR	-0.0022	0.0168	0.0147	-0.0020	69.6	73.4	3.9	0	0	1	1	1	0
SADT	-0.0022	0.0168	0.0147	-0.0020	381.9	274.7	-107.2	0	0	1	1	1	1
SULRy	0.0022	-0.0168	-0.0147	-0.0020	128.0	144.8	16.8	0	0	1	1	1	0
HMGCOAS	0.0073	-0.0547	-0.0481	-0.0066	254.4	413.7	159.3	0	0	1	1	1	0
MEVK3	-0.0073	0.0547	0.0481	-0.0066	18.2	36.1	17.9	0	0	1	1	1	0
DMATT	-0.0024	0.0182	0.0160	-0.0022	286.5	238.7	-47.8	0	0	1	1	1	1
GRTT	-0.0024	0.0182	0.0160	-0.0022	286.5	238.7	-47.8	0	0	1	1	1	1
PMEVK	-0.0073	0.0547	0.0481	-0.0066	29.9	18.9	-11.1	0	0	1	1	1	1
IPDDI	-0.0024	0.0182	0.0160	-0.0022	145.0	177.6	32.5	0	0	1	1	1	0
HMGCOAR	0.0073	-0.0547	-0.0481	-0.0066	31.1	43.7	12.6	0	0	1	1	1	0
DPMVD	-0.0073	0.0547	0.0481	-0.0066	158.7	146.9	-11.8	0	0	1	1	1	1
CITtam	0.0031	-0.0233	-0.0205	-0.0028	120.0	84.9	-35.1	0	0	1	1	1	1
CITtcm	-0.0031	0.0233	0.0205	-0.0028	120.0	84.9	-35.1	0	0	1	1	1	1
ATPtm-H	-1.9651	5.9072	4.1207	-1.7864	4160.0	9544.2	5384.2	0	0	1	1	1	0
SFC1	0.0091	-0.0687	-0.0604	-0.0083	175.5	72.4	-103.1	0	0	1	1	1	1
ASPGLU2m	-0.3034	-0.2740	-0.5499	0.2758	200.5	115.0	-85.4	0	0	1	1	1	0
34HPPt2m	-0.0015	0.0116	0.0102	-0.0014	0.0	0.0	0.0	0	0	0	1	0	0
3C3HMPtm	0.0051	-0.0384	-0.0338	-0.0046	0.0	0.0	0.0	0	0	0	1	0	0
3MOBtm	-0.0046	0.0345	0.0303	-0.0042	0.0	0.0	0.0	0	0	0	1	0	0
ACPtm	0.0004	-0.0030	-0.0027	-0.0004	0.0	0.0	0.0	0	0	0	1	0	0
ALAtm	0.3226	0.1295	0.4228	0.2933	0.0	0.0	0.0	0	0	0	1	0	0
CO2tm	0.0844	-0.6364	-0.5597	-0.0768	0.0	0.0	0.0	0	0	0	1	0	0
COAtm	-0.0060	0.0456	0.0401	-0.0055	0.0	0.0	0.0	0	0	0	1	0	0
DHAPtm	-2.5235	3.4904	1.1963	-2.2941	0.0	0.0	0.0	0	0	0	1	0	0
FA180ACPtm	-0.0004	0.0028	0.0025	-0.0003	0.0	0.0	0.0	0	0	0	1	0	0
FA200ACPtm	0.0000	0.0001	0.0001	0.0000	0.0	0.0	0.0	0	0	0	1	0	0
FA220ACPtm	0.0000	0.0001	0.0001	0.0000	0.0	0.0	0.0	0	0	0	1	0	0

GLYC3Ptm	-2.5235	3.4904	1.1963	-2.2941	0.0	0.0	0.0	0	0	0	1	0	0
H2Otm	2.5645	-3.7992	-1.4678	-2.3314	0.0	0.0	0.0	0	0	0	1	0	0
HIBUTtm	0.0030	-0.0228	-0.0200	-0.0027	0.0	0.0	0.0	0	0	0	1	0	0
IBCOAtm	-0.0030	0.0228	0.0200	-0.0027	0.0	0.0	0.0	0	0	0	1	0	0
O2tm	-0.9833	2.2074	1.3135	-0.8939	0.0	0.0	0.0	0	0	0	1	0	0
OAAtm	-0.3173	-0.1697	-0.4581	0.2884	0.0	0.0	0.0	0	0	0	1	0	0
ORNtm	-0.0038	0.0285	0.0251	-0.0034	0.0	0.0	0.0	0	0	0	1	0	0
Petm	0.0000	0.0000	0.0000	0.0000	0.0	0.0	0.0	0	0	0	1	0	0
PPCOAM	-0.0030	0.0228	0.0200	-0.0027	0.0	0.0	0.0	0	0	0	1	0	0
PROtm	0.3135	0.1982	0.4832	0.2850	0.0	0.0	0.0	0	0	0	1	0	0
PStm	0.0000	0.0000	0.0000	0.0000	0.0	0.0	0.0	0	0	0	1	0	0
PYRtm	0.2570	0.6241	0.8577	0.2336	0.0	0.0	0.0	0	0	0	1	0	0
TYRt2m	0.0015	-0.0116	-0.0102	-0.0014	0.0	0.0	0.0	0	0	0	1	0	0
XYLt	0.0000	5.0000	5.0000	0.0000	61.5	28.0	-33.6	1	0	1	1	1	0
PIt2r	-0.0055	0.0416	0.0366	-0.0050	222.2	758.1	535.9	0	0	1	1	1	0
NH4t	-0.1345	1.0141	0.8918	-0.1223	798.3	701.8	-96.6	0	0	1	1	1	1
SO4t	-0.0022	0.0168	0.0147	-0.0020	242.3	353.2	110.9	0	0	1	1	1	0
CO2t	0.2590	-7.8617	-7.6262	-0.2355	0.0	0.0	0.0	1	0	0	1	0	0
ETOHt	-0.8201	-5.3240	-6.0695	0.7456	0.0	0.0	0.0	0	0	0	1	0	0
FORt	0.0012	-0.0091	-0.0080	-0.0011	0.0	0.0	0.0	0	0	0	1	0	0
H2Ot	1.2363	-4.5070	-3.3830	-1.1239	0.0	0.0	0.0	0	0	0	1	0	0
HCO3E	-0.0493	0.3715	0.3267	-0.0448	0.0	0.0	0.0	0	0	0	1	0	0
HCO3Em	-0.0032	0.0244	0.0215	-0.0029	0.0	0.0	0.0	0	0	0	1	0	0
HEXCOAt	0.0000	0.0002	0.0002	0.0000	0.0	0.0	0.0	0	0	0	1	0	0
O2t	-1.0000	2.3333	1.4242	-0.9091	0.0	0.0	0.0	0	0	0	1	0	0
XYLTt	0.1237	-0.1482	-0.0358	-0.1125	0.0	0.0	0.0	0	0	0	1	0	0
CAT	-0.0014	0.0107	0.0094	-0.0013	496.1	112.7	-383.4	0	0	1	1	1	1
IPMD	-0.0051	0.0384	0.0338	-0.0046	305.6	212.1	-93.5	0	0	1	1	1	1
IPPMIa	0.0051	-0.0384	-0.0338	-0.0046	364.6	211.2	-153.4	0	0	1	1	1	1
IPPMIb	0.0051	-0.0384	-0.0338	-0.0046	364.6	211.2	-153.4	0	0	1	1	1	1
HBUTHYD	-0.0030	0.0228	0.0200	-0.0027	184.4	55.9	-128.5	0	0	1	1	1	1
MACRYLCOAH	-0.0030	0.0228	0.0200	-0.0027	184.4	55.9	-128.5	0	0	1	1	1	1
MBUT2COAH	0.0030	-0.0228	-0.0200	-0.0027	184.4	55.9	-128.5	0	0	1	1	1	1
HACD8	0.0030	-0.0228	-0.0200	-0.0027	12.7	14.1	1.3	0	0	1	1	1	0

ACACT2	0.0030	-0.0228	-0.0200	-0.0027	456.1	409.7	-46.4	0	0	1	1	1	1
BACDH	-0.0030	0.0228	0.0200	-0.0027	211.4	7.8	-203.6	0	0	1	1	1	1
MBUTCOAH	0.0030	-0.0228	-0.0200	-0.0027	211.4	7.8	-203.6	0	0	1	1	1	1
HIBUTDH	-0.0030	0.0228	0.0200	-0.0027	435.4	9.4	-426.0	0	0	1	1	1	1
OIVALDH	0.0030	-0.0228	-0.0200	-0.0027	19.7	25.5	5.8	0	0	1	1	1	0
OVALDH	-0.0030	0.0228	0.0200	-0.0027	159.9	122.6	-37.3	0	0	1	1	1	1
ILETA	0.0030	-0.0228	-0.0200	-0.0027	324.1	285.3	-38.8	0	0	1	1	1	1
LEUTA	0.0051	-0.0384	-0.0338	-0.0046	324.1	285.3	-38.8	0	0	1	1	1	1
OMCDC	-0.0051	0.0384	0.0338	-0.0046	324.1	285.3	-38.8	0	0	1	1	1	1
VALTA	0.0046	-0.0345	-0.0303	-0.0042	324.1	285.3	-38.8	0	0	1	1	1	1
Biomass	-0.0208	0.1567	0.1378	-0.0189	0.0	0.0	0.0	0	0	0	1	0	0
GMPPRPP	0.0087	-0.0654	-0.0575	-0.0079	133.5	270.6	137.1	0	0	1	1	1	0
PIPLC	0.0000	0.0000	0.0000	0.0000	626.2	206.0	-420.2	0	0	1	1	1	1
ALT	0.3355	0.0321	0.3371	0.3050	64.2	21.6	-42.6	0	0	1	1	1	0
PD	0.0000	0.0000	0.0000	0.0000	16.9	13.9	-3.0	0	0	1	1	1	1
CDPSPT	-0.0001	0.0006	0.0006	-0.0001	183.2	138.5	-44.7	0	0	1	1	1	1
MAKG	-0.0091	0.0687	0.0604	-0.0083	0.0	0.0	0.0	0	0	0	1	0	0
Ht	-2.1089	11.9916	10.0743	-1.9172	0.0	0.0	0.0	0	0	0	1	0	0
PYRC	0.3102	0.2231	0.5051	0.2820	72.8	28.6	-44.1	0	0	1	1	1	0
PYRCm	-0.3135	-0.1982	-0.4832	0.2850	72.8	28.6	-44.1	0	0	1	1	1	0
MOC	-0.0055	0.0411	0.0361	-0.0050	623.4	2303.6	1680.1	0	0	1	1	1	0
GLUD2	0.1035	-0.7806	-0.6864	-0.0941	11201.5	8097.0	-3104.5	0	0	1	1	1	1
