# Supplementary material for: Elucidating redox balance shift in Scheffersomyces stipitis’ fermentative metabolism using a modified genome-scale metabolic model
Source: Microb Cell Fact. 2018 Sep 5;17:140. doi: 10.1186/s12934-018-0983-y (PMC6126012; doi:10.1186/s12934-018-0983-y)
Supplement: Supplementary file 9 — Additional file 9: Table S9. Genome-wide comparison for Trial II. [file 12934_2018_983_MOESM9_ESM.pdf]

Table S9: Genome-wide comparison for Trial II

Rxn	Load	Flux Pt 1	Flux Pt 3	Abs Flux Diff	TPM Aero	TPM OL	TPM Change	Flux Change <10%?	TPM Change <10%	Protein Assocation?	Flux?	Active w/Protein?	Correct?
ARGSS	-0.004	0.029	0.025	-0.003	332.3	56.2	-276.1	0	0	1	1	1	1
ASPTAm	0.303	0.274	0.550	0.276	1615.8	697.8	-918.0	0	0	1	1	1	0
ARGSL	-0.004	0.029	0.025	-0.003	102.0	45.8	-56.2	0	0	1	1	1	1
ASPTA	-0.280	-0.449	-0.704	0.255	495.4	81.5	-414.0	0	0	1	1	1	0
ALATA_L	-0.323	-0.130	-0.423	0.293	88.4	16.5	-71.9	0	0	1	1	1	0
CBPS	-0.007	0.050	0.044	-0.006	80.1	10.0	-70.2	0	0	1	1	1	1
GLNS	-0.031	0.231	0.203	-0.028	1517.7	2400.0	882.3	0	0	1	1	1	0
ASNS1	-0.003	0.024	0.021	-0.003	260.5	226.9	-33.7	0	0	1	1	1	1
GLUPRT	-0.002	0.016	0.014	-0.002	50.9	166.5	115.6	0	0	1	1	1	0
CHTNS	-0.009	0.071	0.062	-0.009	375.9	150.5	-225.4	0	0	1	1	1	1
CYB5R	-0.001	0.005	0.004	-0.001	912.4	575.1	-337.3	0	0	1	1	1	1
ACGAMPP	-0.009	0.071	0.062	-0.009	175.6	76.0	-99.6	0	0	1	1	1	1
GF6PTA	-0.009	0.071	0.062	-0.009	268.1	112.7	-155.4	0	0	1	1	1	1
ACGAM6PS	-0.009	0.071	0.062	-0.009	53.4	18.5	-34.8	0	0	1	1	1	1
ACGAMPM	-0.009	0.071	0.062	-0.009	81.9	33.2	-48.7	0	0	1	1	1	1
P5CR	0.310	0.223	0.505	0.282	147.7	121.1	-26.6	0	0	1	1	1	0
OCBT	-0.004	0.029	0.025	-0.003	84.2	3.0	-81.2	0	0	1	1	1	1
ACOTAm	-0.004	0.029	0.025	-0.003	81.2	13.7	-67.5	0	0	1	1	1	1
ACGKm	-0.004	0.029	0.025	-0.003	85.1	28.5	-56.5	0	0	1	1	1	1
AGPRm	-0.004	0.029	0.025	-0.003	85.1	28.5	-56.5	0	0	1	1	1	1
ORNTACim	-0.004	0.029	0.025	-0.003	56.6	13.5	-43.1	0	0	1	1	1	1
PRO1m	0.313	0.198	0.483	0.285	70.4	31.3	-39.0	0	0	1	1	1	0
SUCCDHpm	-0.003	0.023	0.020	-0.003	140.2	89.5	-50.6	0	0	1	1	1	1
MDHm	-0.012	0.092	0.081	-0.011	2466.3	706.5	-1759.8	0	0	1	1	1	1
ICDH1m	-0.023	0.173	0.152	-0.021	564.6	282.7	-281.9	0	0	1	1	1	1
ACONHm	-0.026	0.196	0.173	-0.024	508.4	284.3	-224.1	0	0	1	1	1	1
CITSm	-0.026	0.196	0.173	-0.024	1960.0	67.3	-1892.7	0	0	1	1	1	1
FUMm	-0.012	0.092	0.081	-0.011	612.2	317.0	-295.2	0	0	1	1	1	1
SHSL1	-0.001	0.009	0.008	-0.001	126.4	70.1	-56.3	0	0	1	1	1	1
SHSL4	0.001	-0.009	-0.008	-0.001	126.4	70.1	-56.3	0	0	1	1	1	1
HSD1	-0.005	0.037	0.033	-0.004	196.6	104.4	-92.2	0	0	1	1	1	1
METAT	-0.001	0.009	0.008	-0.001	682.0	1843.2	1161.1	0	0	1	1	1	0

ASPK	-0.005	0.037	0.033	-0.004	132.5	63.6	-68.9	0	0	1	1	1	1
AHC	-0.001	0.009	0.008	-0.001	590.6	679.0	88.4	0	0	1	1	1	0
METS	-0.002	0.018	0.015	-0.002	750.6	559.9	-190.7	0	0	1	1	1	1
ASAD	-0.005	0.037	0.033	-0.004	295.8	343.7	47.9	0	0	1	1	1	0
SERAT	-0.002	0.017	0.015	-0.002	0.0	0.0	0.0	0	0	0	1	0	0
CYSS	-0.002	0.017	0.015	-0.002	215.1	101.8	-113.3	0	0	1	1	1	1
CYSTL	-0.001	0.009	0.008	-0.001	194.3	53.8	-140.5	0	0	1	1	1	1
EX_co2(e)	-0.259	7.862	7.626	-0.235	0.0	0.0	0.0	1	0	0	1	0	0
EX_etoh(e)	0.820	5.324	6.070	0.746	0.0	0.0	0.0	0	0	0	1	0	0
EX_for(e)	-0.001	0.009	0.008	-0.001	0.0	0.0	0.0	0	0	0	1	0	0
EX_h(e)	-2.103	6.950	5.038	-1.912	0.0	0.0	0.0	0	0	0	1	0	0
EX_h2o(e)	-1.236	4.507	3.383	-1.124	0.0	0.0	0.0	0	0	0	1	0	0
EX_nh4(e)	0.135	-1.014	-0.892	-0.122	0.0	0.0	0.0	0	0	0	1	0	0
EX_o2(e)	1.000	-2.333	-1.424	-0.909	0.0	0.0	0.0	0	0	0	1	0	0
EX_pi(e)	0.006	-0.042	-0.037	-0.005	0.0	0.0	0.0	0	0	0	1	0	0
EX_so4(e)	0.002	-0.017	-0.015	-0.002	0.0	0.0	0.0	0	0	0	1	0	0
EX_xyl-D(e)	0.000	-5.000	-5.000	0.000	0.0	0.0	0.0	1	0	0	1	0	0
EX_xylt(e)	-0.124	0.148	0.036	-0.112	0.0	0.0	0.0	0	0	0	1	0	0
Hexacoa_ex	0.000	0.000	0.000	0.000	0.0	0.0	0.0	0	0	0	1	0	0
FACOAL180	0.000	0.003	0.002	0.000	1509.7	211.2	-1298.5	0	0	1	1	1	1
FAS100ACPm	0.000	0.003	0.003	0.000	36.6	16.3	-20.3	0	0	1	1	1	1
FAS120ACPm	0.000	0.003	0.003	0.000	36.6	16.3	-20.3	0	0	1	1	1	1
FAS140ACPm	0.000	0.003	0.003	0.000	36.6	16.3	-20.3	0	0	1	1	1	1
FAS160ACPm	0.000	0.003	0.003	0.000	36.6	16.3	-20.3	0	0	1	1	1	1
FAS180ACPm	0.000	0.003	0.003	0.000	36.6	16.3	-20.3	0	0	1	1	1	1
FAS200ACPm	0.000	0.000	0.000	0.000	36.6	16.3	-20.3	0	0	1	1	1	1
FAS220ACPm	0.000	0.000	0.000	0.000	36.6	16.3	-20.3	0	0	1	1	1	1
FAS80ACPm_L	0.000	0.003	0.003	0.000	36.6	16.3	-20.3	0	0	1	1	1	1
MCOATAm	-0.003	0.024	0.021	-0.003	23.4	14.4	-9.0	0	0	1	1	1	1
DESAT16	0.000	0.001	0.001	0.000	786.2	2739.5	1953.3	0	0	1	1	1	0
DESAT18	-0.001	0.004	0.004	0.000	786.2	2739.5	1953.3	0	0	1	1	1	0
ACOATAm	0.000	0.003	0.003	0.000	114.6	273.0	158.3	0	0	1	1	1	0
FA180ACPH	0.000	0.003	0.002	0.000	114.6	273.0	158.3	0	0	1	1	1	0
FA200ACPH	0.000	0.000	0.000	0.000	114.6	273.0	158.3	0	0	1	1	1	0

FA220ACPH	0.000	0.000	0.000	0.000	114.6	273.0	158.3	0	0	1	1	1	0
FAS100COA	-0.001	0.006	0.005	-0.001	114.6	273.0	158.3	0	0	1	1	1	0
FAS120COA	-0.001	0.006	0.005	-0.001	114.6	273.0	158.3	0	0	1	1	1	0
FAS140COA	-0.001	0.005	0.004	-0.001	114.6	273.0	158.3	0	0	1	1	1	0
FAS160COA	-0.001	0.004	0.004	-0.001	114.6	273.0	158.3	0	0	1	1	1	0
FAS180COA	0.000	0.002	0.001	0.000	114.6	273.0	158.3	0	0	1	1	1	0
FAS60COA_L	0.000	0.000	0.000	0.000	114.6	273.0	158.3	0	0	1	1	1	0
FAS80COA_L	-0.001	0.006	0.005	-0.001	114.6	273.0	158.3	0	0	1	1	1	0
DESAT18_3	0.000	0.000	0.000	0.000	0.0	0.0	0.0	0	0	0	1	0	0
DESAT20_5	0.000	0.000	0.000	0.000	0.0	0.0	0.0	0	0	0	1	0	0
DHFRi	0.000	0.002	0.002	0.000	134.7	61.5	-73.2	0	0	1	1	1	1
MTHFR2	-0.002	0.018	0.015	-0.002	134.8	69.3	-65.6	0	0	1	1	1	1
MTHFD	-0.006	0.047	0.041	-0.006	204.0	204.3	0.3	0	1	0	1	0	0
MTHFC	-0.006	0.047	0.041	-0.006	109.3	72.0	-37.3	0	0	1	1	1	1
MAN6PI	0.015	-0.112	-0.099	-0.014	102.5	72.3	-30.2	0	0	1	1	1	1
MAN1PGT	-0.015	0.112	0.099	-0.014	1271.7	992.2	-279.5	0	0	1	1	1	1
PMANM	0.015	-0.112	-0.099	-0.014	395.6	193.0	-202.6	0	0	1	1	1	1
DOLPMMer	-0.015	0.112	0.099	-0.014	21.5	8.5	-13.0	0	0	1	1	1	1
DOLPMTcer	-0.015	0.112	0.099	-0.014	0.0	0.0	0.0	0	0	0	1	0	0
GAT1	-0.001	0.005	0.004	-0.001	123.0	88.1	-34.9	0	0	1	1	1	1
AGAT	-0.001	0.005	0.004	-0.001	217.3	155.4	-61.9	0	0	1	1	1	1
DAGPYP	0.000	0.000	0.000	0.000	114.6	37.4	-77.2	0	0	1	1	1	1
PLD	0.000	0.000	0.000	0.000	21.0	12.5	-8.5	0	0	1	1	1	1
G3PD1	-2.524	3.495	1.201	-2.295	160.0	138.6	-21.5	0	0	1	1	1	1
G3PDm	-2.524	3.490	1.196	-2.294	34.9	26.1	-8.8	0	0	1	1	1	1
PSERDm	0.000	0.001	0.001	0.000	178.1	92.2	-85.9	0	0	1	1	1	1
PSERT	-0.016	0.124	0.109	-0.015	112.9	42.9	-70.0	0	0	1	1	1	1
PSPL	-0.016	0.124	0.109	-0.015	72.8	57.4	-15.4	0	0	1	1	1	1
AGT	-0.003	0.023	0.020	-0.003	250.2	54.0	-196.3	0	0	1	1	1	1
THRS	-0.005	0.037	0.033	-0.004	189.8	147.5	-42.3	0	0	1	1	1	1
THRD	-0.001	0.009	0.008	-0.001	58.2	58.2	0.0	0	1	0	1	0	0
HSK	-0.005	0.037	0.033	-0.004	65.7	121.9	56.2	0	0	1	1	1	0
PGCD	-0.016	0.124	0.109	-0.015	390.5	355.2	-35.3	0	1	0	1	0	0
PGM	-0.660	-6.533	-7.133	0.600	2118.8	4111.6	1992.8	1	0	1	1	1	1

PFK	0.462	2.242	2.661	0.420	93.3	139.5	46.3	0	0	1	1	1	1
ENO	0.660	6.533	7.133	0.600	2217.3	4326.0	2108.7	1	0	1	1	1	1
GAPD	0.643	6.657	7.242	0.585	4339.5	9114.0	4774.4	1	0	1	1	1	1
PGK	-0.643	-6.657	-7.242	0.585	1550.2	2543.9	993.7	1	0	1	1	1	1
PYK	0.669	6.466	7.074	0.608	502.9	1010.3	507.4	1	0	1	1	1	1
G6PI	1.002	-1.959	-1.047	-0.911	1226.0	784.0	-441.9	0	0	1	1	1	1
FBA	0.462	2.242	2.661	0.420	828.4	1107.6	279.2	0	0	1	1	1	1
TPI	0.462	2.237	2.657	0.420	1105.1	1431.4	326.3	0	0	1	1	1	1
ICL	-0.003	0.023	0.020	-0.003	697.7	89.0	-608.7	0	0	1	1	1	1
HISTP	-0.002	0.016	0.014	-0.002	23.9	12.5	-11.3	0	0	1	1	1	1
PRMICI	-0.002	0.016	0.014	-0.002	33.5	55.9	22.4	0	0	1	1	1	0
IG3PS	-0.002	0.016	0.014	-0.002	86.1	55.6	-30.5	0	0	1	1	1	1
ATPPRT	-0.002	0.016	0.014	-0.002	81.1	56.5	-24.5	0	0	1	1	1	1
HISTD	-0.002	0.016	0.014	-0.002	85.5	41.7	-43.8	0	0	1	1	1	1
PRAMPC	-0.002	0.016	0.014	-0.002	85.5	41.7	-43.8	0	0	1	1	1	1
PRATPP	-0.002	0.016	0.014	-0.002	85.5	41.7	-43.8	0	0	1	1	1	1
IGPDH	-0.002	0.016	0.014	-0.002	0.0	0.0	0.0	0	0	0	1	0	0
HSTPT	-0.002	0.016	0.014	-0.002	63.0	31.9	-31.1	0	0	1	1	1	1
HICITDm	-0.005	0.041	0.036	-0.005	365.4	150.8	-214.6	0	0	1	1	1	1
AATA	-0.005	0.041	0.036	-0.005	238.7	59.9	-178.8	0	0	1	1	1	1
AASAD2	-0.005	0.041	0.036	-0.005	88.4	37.2	-51.2	0	0	1	1	1	1
SACCD1	-0.005	0.041	0.036	-0.005	324.2	687.0	362.8	0	0	1	1	1	0
SACCD2	-0.005	0.041	0.036	-0.005	324.9	618.7	293.9	0	0	1	1	1	0
OXAGm	-0.005	0.041	0.036	-0.005	0.0	0.0	0.0	0	0	0	1	0	0
RNTR1	0.000	0.002	0.002	0.000	1477.8	1030.2	-447.6	0	0	1	1	1	1
GSNK	-0.009	0.065	0.058	-0.008	0.0	0.0	0.0	0	0	0	1	0	0
GHMT	-0.009	0.066	0.058	-0.008	357.9	290.4	-67.5	0	0	1	1	1	1
CYOR_u6m	-1.967	4.415	2.627	-1.788	111.1	92.0	-19.2	0	0	1	1	1	1
NADH2-u6t	0.560	0.901	1.410	0.509	40.1	38.8	-1.3	0	1	0	1	0	0
SUCCDH1m	-2.527	3.514	1.217	-2.297	140.2	89.5	-50.6	0	0	1	1	1	1
ATPSm	-1.972	5.960	4.167	-1.793	384.5	259.0	-125.5	0	0	1	1	1	1
CYOOm	-0.983	2.207	1.313	-0.894	31.4	37.0	5.6	0	0	1	1	1	0
PPA	-0.114	0.860	0.757	-0.104	773.9	497.9	-276.0	0	0	1	1	1	1
DHAD1m	-0.013	0.096	0.084	-0.012	43.0	74.8	31.8	0	0	1	1	1	0

KARA1m	-0.013	0.096	0.084	-0.012	1251.2	1718.0	466.8	0	0	1	1	1	0
XYLK	0.124	4.852	4.964	0.112	464.6	1351.5	886.9	1	0	1	1	1	1
XYLUR	-0.124	-4.852	-4.964	0.112	4420.7	11322.4	6901.7	1	0	1	1	1	1
RPE	-0.689	-0.468	-1.094	0.626	194.5	128.0	-66.5	0	0	1	1	1	0
TKT1	-0.280	2.175	1.920	-0.255	1186.3	799.5	-386.8	0	0	1	1	1	1
TKT2	-0.285	2.208	1.950	-0.259	1186.3	799.5	-386.8	0	0	1	1	1	1
PGL	-0.981	1.797	0.905	-0.892	384.4	228.9	-155.5	0	0	1	1	1	1
GND	-0.981	1.797	0.905	-0.892	2249.5	992.0	-1257.5	0	0	1	1	1	1
TALA	-0.285	2.208	1.950	-0.259	2567.7	5019.5	2451.8	0	0	1	1	1	0
RPI	0.292	-2.265	-1.999	-0.266	592.8	225.6	-367.1	0	0	1	1	1	1
G6PDH	-0.981	1.797	0.905	-0.892	763.9	382.0	-381.9	0	0	1	1	1	1
PPM	0.008	-0.057	-0.050	-0.007	604.9	306.3	-298.6	0	0	1	1	1	1
FACOAL200	0.000	0.000	0.000	0.000	1509.7	211.2	-1298.5	0	0	1	1	1	1
FACOAL220	0.000	0.000	0.000	0.000	1509.7	211.2	-1298.5	0	0	1	1	1	1
DESAT18_2	0.000	0.001	0.001	0.000	153.7	987.4	833.7	0	0	1	1	1	0
PPNDH	-0.002	0.018	0.016	-0.002	60.6	19.6	-41.0	0	0	1	1	1	1
ANPRT	-0.001	0.004	0.003	0.000	93.0	39.9	-53.1	0	0	1	1	1	1
PRAI	-0.001	0.004	0.003	0.000	45.4	22.6	-22.8	0	0	1	1	1	1
TYRTAm	0.002	-0.012	-0.010	-0.001	1615.8	697.8	-918.0	0	0	1	1	1	1
PPND	-0.002	0.012	0.010	-0.001	51.7	36.3	-15.4	0	0	1	1	1	1
DHQT	-0.004	0.033	0.029	-0.004	195.8	61.4	-134.4	0	0	1	1	1	1
PHETA1	0.002	-0.018	-0.016	-0.002	289.0	62.6	-226.4	0	0	1	1	1	1
TRPS1	-0.001	0.004	0.003	0.000	157.1	86.1	-71.0	0	0	1	1	1	1
CHORM	-0.004	0.030	0.026	-0.004	40.3	19.8	-20.5	0	0	1	1	1	1
ANS	-0.001	0.004	0.003	0.000	9.7	10.2	0.5	0	1	0	1	0	0
DDPA	-0.004	0.033	0.029	-0.004	295.7	276.9	-18.8	0	1	0	1	0	0
DHQS	-0.004	0.033	0.029	-0.004	58.7	38.0	-20.8	0	0	1	1	1	1
PSCVT	-0.004	0.033	0.029	-0.004	58.7	38.0	-20.8	0	0	1	1	1	1
SHK3D	-0.004	0.033	0.029	-0.004	58.7	38.0	-20.8	0	0	1	1	1	1
SHKK	-0.004	0.033	0.029	-0.004	58.7	38.0	-20.8	0	0	1	1	1	1
CHORS	-0.004	0.033	0.029	-0.004	240.1	261.8	21.7	0	1	0	1	0	0
IGPS	-0.001	0.004	0.003	0.000	128.7	91.2	-37.5	0	0	1	1	1	1
DASYN	-0.001	0.005	0.004	-0.001	74.5	45.0	-29.5	0	0	1	1	1	1
MMSAD1	-0.003	0.023	0.020	-0.003	70.9	3.0	-67.9	0	0	1	1	1	1

ADPT	-0.001	0.009	0.008	-0.001	270.7	308.1	37.5	0	0	1	1	1	0
RNDR2	0.000	0.001	0.001	0.000	20.9	12.4	-8.5	0	0	1	1	1	1
GARFT	-0.002	0.016	0.014	-0.002	40.3	85.4	45.1	0	0	1	1	1	0
PRASCS	-0.002	0.016	0.014	-0.002	157.2	105.0	-52.2	0	0	1	1	1	1
ADK1	-0.062	0.470	0.413	-0.057	482.9	310.6	-172.3	0	0	1	1	1	1
IMPD	-0.001	0.007	0.006	-0.001	135.4	783.1	647.8	0	0	1	1	1	0
NDPK1	-0.025	0.191	0.168	-0.023	685.5	1343.2	657.7	0	0	1	1	1	0
AICART	-0.004	0.031	0.028	-0.004	327.8	244.0	-83.8	0	0	1	1	1	1
IMPC	0.004	-0.031	-0.028	-0.004	327.8	244.0	-83.8	0	0	1	1	1	1
GMPS	-0.001	0.007	0.006	-0.001	67.9	539.8	471.9	0	0	1	1	1	0
GK3	0.000	-0.001	-0.001	0.000	245.1	185.6	-59.4	0	0	1	1	1	1
PRFGS	-0.002	0.016	0.014	-0.002	43.4	75.3	31.9	0	0	1	1	1	0
PRAGS	-0.002	0.016	0.014	-0.002	62.4	145.4	83.0	0	0	1	1	1	0
PRAIS	-0.002	0.016	0.014	-0.002	62.4	145.4	83.0	0	0	1	1	1	0
AIRC	-0.002	0.016	0.014	-0.002	37.7	125.6	87.9	0	0	1	1	1	0
ADSL1	-0.003	0.024	0.022	-0.003	110.6	250.7	140.0	0	0	1	1	1	0
ADSL2	-0.002	0.016	0.014	-0.002	110.6	250.7	140.0	0	0	1	1	1	0
ADSS	-0.003	0.024	0.022	-0.003	351.2	526.6	175.4	0	0	1	1	1	0
ADSK	-0.002	0.017	0.015	-0.002	94.9	77.8	-17.1	0	0	1	1	1	1
DADK	0.000	-0.002	-0.002	0.000	0.0	0.0	0.0	0	0	0	1	0	0
PUNP1	-0.001	0.009	0.008	-0.001	0.0	0.0	0.0	0	0	0	1	0	0
PUNP3	0.009	-0.065	-0.058	-0.008	0.0	0.0	0.0	0	0	0	1	0	0
RNDR4	0.000	0.003	0.003	0.000	20.9	12.4	-8.5	0	0	1	1	1	1
DHORTS	0.003	-0.021	-0.019	-0.003	91.9	107.2	15.4	0	0	1	1	1	0
DCMPDA	0.000	-0.001	-0.001	0.000	33.5	12.0	-21.5	0	0	1	1	1	1
TMDS	0.000	0.002	0.002	0.000	341.2	37.6	-303.6	0	0	1	1	1	1
OMPDC	-0.003	0.021	0.019	-0.003	102.4	125.3	22.8	0	0	1	1	1	0
DHORD	-0.003	0.021	0.019	-0.003	86.3	138.1	51.8	0	0	1	1	1	0
TRDR	-0.003	0.023	0.020	-0.003	494.3	3608.3	3114.0	0	0	1	1	1	0
CTPS1	-0.001	0.010	0.009	-0.001	54.6	268.7	214.1	0	0	1	1	1	0
ASPCT	-0.003	0.021	0.019	-0.003	80.1	10.0	-70.2	0	0	1	1	1	1
CYTK1	0.001	-0.005	-0.005	-0.001	136.7	88.6	-48.0	0	0	1	1	1	1
UMPK	-0.002	0.013	0.012	-0.002	136.7	88.6	-48.0	0	0	1	1	1	1
URIDK	0.000	-0.003	-0.003	0.000	136.7	88.6	-48.0	0	0	1	1	1	1

ORPT	0.003	-0.021	-0.019	-0.003	109.1	136.7	27.6	0	0	1	1	1	0
NDPK2	-0.031	0.231	0.203	-0.028	685.5	1343.2	657.7	0	0	1	1	1	0
NDPK3	0.001	-0.005	-0.005	-0.001	685.5	1343.2	657.7	0	0	1	1	1	0
ALCDH	-0.820	-5.324	-6.070	0.746	1635.4	7238.6	5603.2	0	0	1	1	1	1
HCITSm	-0.005	0.041	0.036	-0.005	636.1	203.2	-432.9	0	0	1	1	1	1
ACACT1	-0.007	0.055	0.048	-0.007	433.5	332.0	-101.5	0	0	1	1	1	1
ACLSm	-0.013	0.096	0.084	-0.012	271.3	141.8	-129.5	0	0	1	1	1	1
ALDDH1	-0.041	0.307	0.270	-0.037	193.2	41.1	-152.1	0	0	1	1	1	1
PYRDC	0.779	5.631	6.339	0.709	662.8	3448.7	2785.8	0	0	1	1	1	1
IPPS	-0.005	0.038	0.034	-0.005	44.8	76.6	31.8	0	0	1	1	1	0
ACS1	-0.043	0.323	0.284	-0.039	2254.4	314.4	-1939.9	0	0	1	1	1	1
PDHm	-0.040	0.303	0.267	-0.037	555.0	353.4	-201.6	0	0	1	1	1	1
ACCOAC	-0.006	0.042	0.037	-0.005	142.6	207.4	64.8	0	0	1	1	1	0
ACCOACrm	-0.003	0.024	0.021	-0.003	142.6	207.4	64.8	0	0	1	1	1	0
PC	-0.037	0.280	0.246	-0.034	280.0	118.2	-161.8	0	0	1	1	1	1
IPC224PLC	0.000	0.000	0.000	0.000	61.6	16.1	-45.5	0	0	1	1	1	1
IPCS224	0.000	0.000	0.000	0.000	177.5	516.3	338.8	0	0	1	1	1	0
PGMT	0.020	-0.150	-0.132	-0.018	604.9	306.3	-298.6	0	0	1	1	1	1
GALU	-0.020	0.150	0.132	-0.018	569.9	619.3	49.4	0	1	0	1	0	0
C5STDS	-0.001	0.009	0.008	-0.001	1035.0	920.1	-114.9	0	0	1	1	1	1
C24STR	-0.001	0.009	0.008	-0.001	61.2	29.2	-32.0	0	0	1	1	1	1
C14STR	-0.001	0.009	0.008	-0.001	70.1	49.4	-20.8	0	0	1	1	1	1
C3STKR1	-0.001	0.009	0.008	-0.001	78.9	54.1	-24.8	0	0	1	1	1	1
C3STKR2	-0.001	0.009	0.008	-0.001	78.9	54.1	-24.8	0	0	1	1	1	1
LNS14DM	-0.001	0.009	0.008	-0.001	145.3	130.3	-15.0	0	0	1	1	1	1
LNSTLS	-0.001	0.009	0.008	-0.001	145.3	130.3	-15.0	0	0	1	1	1	1
C3STDH1	-0.001	0.009	0.008	-0.001	337.5	229.0	-108.4	0	0	1	1	1	1
C3STDH2	-0.001	0.009	0.008	-0.001	337.5	229.0	-108.4	0	0	1	1	1	1
C22STDS1	-0.001	0.009	0.008	-0.001	164.7	119.4	-45.3	0	0	1	1	1	1
C8STI	-0.001	0.009	0.008	-0.001	141.7	104.4	-37.3	0	0	1	1	1	1
C4STMO1	-0.001	0.009	0.008	-0.001	1.2	671.6	670.4	0	0	1	1	1	0
C4STMO2	-0.001	0.009	0.008	-0.001	1.2	671.6	670.4	0	0	1	1	1	0
SQLE	-0.001	0.009	0.008	-0.001	79.6	101.5	21.9	0	0	1	1	1	0
SAM24MT	-0.001	0.009	0.008	-0.001	681.4	595.2	-86.3	0	0	1	1	1	1

SQLS	-0.001	0.009	0.008	-0.001	291.9	173.2	-118.7	0	0	1	1	1	1
13GS	-0.013	0.096	0.084	-0.012	38.4	16.2	-22.2	0	0	1	1	1	1
TRE6PS	-0.002	0.012	0.010	-0.001	75.2	54.1	-21.1	0	0	1	1	1	1
TRE6PP	-0.002	0.012	0.010	-0.001	107.9	62.9	-45.0	0	0	1	1	1	1
GLYGS	-0.006	0.043	0.037	-0.005	658.9	510.7	-148.2	0	0	1	1	1	1
14GBEZ	-0.006	0.043	0.037	-0.005	280.9	175.1	-105.8	0	0	1	1	1	1
BPNT	-0.002	0.017	0.015	-0.002	127.5	68.9	-58.6	0	0	1	1	1	1
PAPSR	-0.002	0.017	0.015	-0.002	51.1	31.7	-19.3	0	0	1	1	1	1
SADT	-0.002	0.017	0.015	-0.002	307.1	86.9	-220.2	0	0	1	1	1	1
SULRy	0.002	-0.017	-0.015	-0.002	127.8	70.1	-57.7	0	0	1	1	1	1
HMGCOAS	0.007	-0.055	-0.048	-0.007	276.6	274.8	-1.7	0	1	0	1	0	0
MEVK3	-0.007	0.055	0.048	-0.007	27.7	46.7	18.9	0	0	1	1	1	0
DMATT	-0.002	0.018	0.016	-0.002	285.3	234.1	-51.1	0	0	1	1	1	1
GRTT	-0.002	0.018	0.016	-0.002	285.3	234.1	-51.1	0	0	1	1	1	1
PMEVK	-0.007	0.055	0.048	-0.007	68.8	29.1	-39.7	0	0	1	1	1	1
IPDDI	-0.002	0.018	0.016	-0.002	128.2	106.9	-21.3	0	0	1	1	1	1
HMGCOAR	0.007	-0.055	-0.048	-0.007	62.2	39.7	-22.5	0	0	1	1	1	1
DPMVD	-0.007	0.055	0.048	-0.007	149.5	139.5	-10.0	0	1	0	1	0	0
CITtam	0.003	-0.023	-0.020	-0.003	103.8	63.5	-40.3	0	0	1	1	1	1
CITtcm	-0.003	0.023	0.020	-0.003	103.8	63.5	-40.3	0	0	1	1	1	1
ATPtm-H	-1.965	5.907	4.121	-1.786	3253.9	9066.1	5812.2	0	0	1	1	1	0
SFC1	0.009	-0.069	-0.060	-0.008	171.7	21.5	-150.2	0	0	1	1	1	1
ASPGLU2m	-0.303	-0.274	-0.550	0.276	209.7	116.0	-93.7	0	0	1	1	1	0
34HPPt2m	-0.002	0.012	0.010	-0.001	0.0	0.0	0.0	0	0	0	1	0	0
3C3HMPtm	0.005	-0.038	-0.034	-0.005	0.0	0.0	0.0	0	0	0	1	0	0
3MOBtm	-0.005	0.034	0.030	-0.004	0.0	0.0	0.0	0	0	0	1	0	0
ACPtm	0.000	-0.003	-0.003	0.000	0.0	0.0	0.0	0	0	0	1	0	0
ALAtm	0.323	0.130	0.423	0.293	0.0	0.0	0.0	0	0	0	1	0	0
CO2tm	0.084	-0.636	-0.560	-0.077	0.0	0.0	0.0	0	0	0	1	0	0
COAtm	-0.006	0.046	0.040	-0.005	0.0	0.0	0.0	0	0	0	1	0	0
DHAPtm	-2.524	3.490	1.196	-2.294	0.0	0.0	0.0	0	0	0	1	0	0
FA180ACPtm	0.000	0.003	0.002	0.000	0.0	0.0	0.0	0	0	0	1	0	0
FA200ACPtm	0.000	0.000	0.000	0.000	0.0	0.0	0.0	0	0	0	1	0	0
FA220ACPtm	0.000	0.000	0.000	0.000	0.0	0.0	0.0	0	0	0	1	0	0

GLYC3Ptm	-2.524	3.490	1.196	-2.294	0.0	0.0	0.0	0	0	0	1	0	0
H2Otm	2.564	-3.799	-1.468	-2.331	0.0	0.0	0.0	0	0	0	1	0	0
HIBUTtm	0.003	-0.023	-0.020	-0.003	0.0	0.0	0.0	0	0	0	1	0	0
IBCOAtm	-0.003	0.023	0.020	-0.003	0.0	0.0	0.0	0	0	0	1	0	0
O2tm	-0.983	2.207	1.313	-0.894	0.0	0.0	0.0	0	0	0	1	0	0
OAAtm	-0.317	-0.170	-0.458	0.288	0.0	0.0	0.0	0	0	0	1	0	0
ORNtm	-0.004	0.029	0.025	-0.003	0.0	0.0	0.0	0	0	0	1	0	0
Petm	0.000	0.000	0.000	0.000	0.0	0.0	0.0	0	0	0	1	0	0
PPCOAM	-0.003	0.023	0.020	-0.003	0.0	0.0	0.0	0	0	0	1	0	0
PROtm	0.313	0.198	0.483	0.285	0.0	0.0	0.0	0	0	0	1	0	0
PStm	0.000	0.000	0.000	0.000	0.0	0.0	0.0	0	0	0	1	0	0
PYRtm	0.257	0.624	0.858	0.234	0.0	0.0	0.0	0	0	0	1	0	0
TYRt2m	0.002	-0.012	-0.010	-0.001	0.0	0.0	0.0	0	0	0	1	0	0
XYLt	0.000	5.000	5.000	0.000	129.1	29.8	-99.3	1	0	1	1	1	1
PIt2r	-0.006	0.042	0.037	-0.005	256.8	587.4	330.6	0	0	1	1	1	0
NH4t	-0.135	1.014	0.892	-0.122	1660.6	381.5	-1279.2	0	0	1	1	1	1
SO4t	-0.002	0.017	0.015	-0.002	217.9	133.1	-84.8	0	0	1	1	1	1
CO2t	0.259	-7.862	-7.626	-0.235	0.0	0.0	0.0	1	0	0	1	0	0
ETOHt	-0.820	-5.324	-6.070	0.746	0.0	0.0	0.0	0	0	0	1	0	0
FORt	0.001	-0.009	-0.008	-0.001	0.0	0.0	0.0	0	0	0	1	0	0
H2Ot	1.236	-4.507	-3.383	-1.124	0.0	0.0	0.0	0	0	0	1	0	0
HCO3E	-0.049	0.372	0.327	-0.045	0.0	0.0	0.0	0	0	0	1	0	0
HCO3Em	-0.003	0.024	0.021	-0.003	0.0	0.0	0.0	0	0	0	1	0	0
HEXCOAt	0.000	0.000	0.000	0.000	0.0	0.0	0.0	0	0	0	1	0	0
O2t	-1.000	2.333	1.424	-0.909	0.0	0.0	0.0	0	0	0	1	0	0
XYLTt	0.124	-0.148	-0.036	-0.112	0.0	0.0	0.0	0	0	0	1	0	0
CAT	-0.001	0.011	0.009	-0.001	437.4	124.6	-312.9	0	0	1	1	1	1
IPMD	-0.005	0.038	0.034	-0.005	263.5	125.0	-138.5	0	0	1	1	1	1
IPPMIa	0.005	-0.038	-0.034	-0.005	425.2	170.6	-254.6	0	0	1	1	1	1
IPPMIb	0.005	-0.038	-0.034	-0.005	425.2	170.6	-254.6	0	0	1	1	1	1
HBUTHYD	-0.003	0.023	0.020	-0.003	185.3	60.8	-124.5	0	0	1	1	1	1
MACRYLCOAH	-0.003	0.023	0.020	-0.003	185.3	60.8	-124.5	0	0	1	1	1	1
MBUT2COAH	0.003	-0.023	-0.020	-0.003	185.3	60.8	-124.5	0	0	1	1	1	1
HACD8	0.003	-0.023	-0.020	-0.003	11.9	14.0	2.1	0	0	1	1	1	0

ACACT2	0.003	-0.023	-0.020	-0.003	433.5	332.0	-101.5	0	0	1	1	1	1
BACDH	-0.003	0.023	0.020	-0.003	156.7	6.0	-150.8	0	0	1	1	1	1
MBUTCOAH	0.003	-0.023	-0.020	-0.003	156.7	6.0	-150.8	0	0	1	1	1	1
HIBUTDH	-0.003	0.023	0.020	-0.003	493.5	6.7	-486.8	0	0	1	1	1	1
OIVALDH	0.003	-0.023	-0.020	-0.003	26.9	33.5	6.6	0	0	1	1	1	0
OVALDH	-0.003	0.023	0.020	-0.003	105.5	98.3	-7.3	0	1	0	1	0	0
ILETA	0.003	-0.023	-0.020	-0.003	301.3	219.9	-81.5	0	0	1	1	1	1
LEUTA	0.005	-0.038	-0.034	-0.005	301.3	219.9	-81.5	0	0	1	1	1	1
OMCDC	-0.005	0.038	0.034	-0.005	301.3	219.9	-81.5	0	0	1	1	1	1
VALTA	0.005	-0.034	-0.030	-0.004	301.3	219.9	-81.5	0	0	1	1	1	1
Biomass	-0.021	0.157	0.138	-0.019	0.0	0.0	0.0	0	0	0	1	0	0
GMPPRPP	0.009	-0.065	-0.058	-0.008	109.2	215.5	106.4	0	0	1	1	1	0
PIPLC	0.000	0.000	0.000	0.000	596.9	160.0	-436.9	0	0	1	1	1	1
ALT	0.336	0.032	0.337	0.305	88.4	16.5	-71.9	0	0	1	1	1	0
PD	0.000	0.000	0.000	0.000	21.0	12.5	-8.5	0	0	1	1	1	1
CDPSPT	0.000	0.001	0.001	0.000	195.2	150.3	-44.9	0	0	1	1	1	1
MAKG	-0.009	0.069	0.060	-0.008	0.0	0.0	0.0	0	0	0	1	0	0
Ht	-2.109	11.992	10.074	-1.917	0.0	0.0	0.0	0	0	0	1	0	0
PYRC	0.310	0.223	0.505	0.282	87.9	38.9	-49.0	0	0	1	1	1	0
PYRCm	-0.313	-0.198	-0.483	0.285	87.9	38.9	-49.0	0	0	1	1	1	0
MOC	-0.005	0.041	0.036	-0.005	786.2	2739.5	1953.3	0	0	1	1	1	0
GLUD2	0.104	-0.781	-0.686	-0.094	14076.0	6061.5	-8014.5	0	0	1	1	1	1
